# Supplementary material for: The TrkC-PTPσ complex governs synapse maturation and anxiogenic avoidance via synaptic protein phosphorylation
Source: EMBO J. 2024 Sep 27;43(22):5690–717. doi: 10.1038/s44318-024-00252-9 (PMC11574141; doi:10.1038/s44318-024-00252-9)
Supplement: Supplementary file 1 — Appendix [file 44318_2024_252_MOESM1_ESM.pdf]

APPENDIX

The TrkC-PTPσ complex governs synapse maturation and anxiogenic avoidance via synaptic protein phosphorylation

Husam Khaled *et al.*

Lead contact: Hideto.Takahashi@ircm.qc.ca

This file includes:

Appendix Figures S1 to S10
Appendix Tables S1 to S4

Appendix Figures

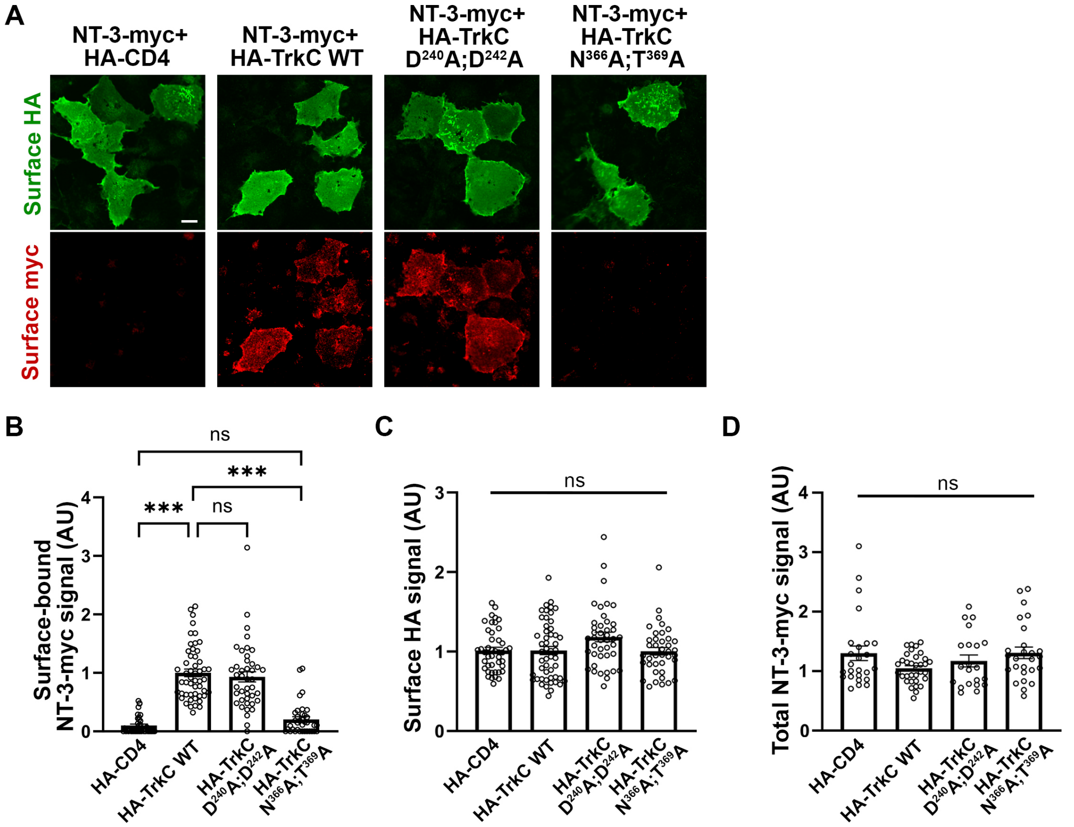

Appendix Figure S1. TrkC D240A;D242A substitution has no effect on neurotrophin-3 binding to TrkC

(**A**) Double immunolabeling for cell surface-bound neurotrophin-3 (NT-3)-myc and surface-expressed HA-TrkC wild-type (WT), HA-TrkC D240A;D242A (PTPσ-binding dead mutant), HA-TrkC N366A;T369A (NT-3-binding dead mutant, a negative control) or HA-CD4 (another negative control) on COS-7 cells. Cells expressing HA-TrkC WT and HA-TrkC D240A;D242A show a comparable bound NT-3-myc signal, while cells expressing HA-CD4 and HA-TrkC N366A;T369A show no significant signal for bound NT-3-myc. Scale bar: 20 µm.

(**B**) Quantification of surface-bound NT-3-myc on COS-7 cells expressing the indicated HA-tagged proteins. n > 35 cells from three independent experiments.

(**C**) Quantification of the surface HA intensity on the COS-7 cells shows no significant difference in surface intensity for transfections with the different HA-TrkC constructs. n > 35 cells from three independent experiments.

(**D**) Quantification of total NT-3-myc expression in COS-7 cells expressing the indicated HA-tagged proteins. All conditions show a comparable total NT-3-myc signal level, suggesting that the lack of significant signal corresponding to bound NT-3 on COS-7 cells expressing HA-TrkC N366A;T369A and HA-CD4 is not due to poor transfection of the NT-3-myc plasmid, but rather due to the inability of NT-3 to bind TrkC N366A;T369A and CD4. n ≥ 20 cells from two independent experiments.

Statistical significance was examined by a Kruskal-Wallis test with Dunn's post hoc analysis. ***p < 0.001, ns: not significant. Data are presented as mean ± SEM.

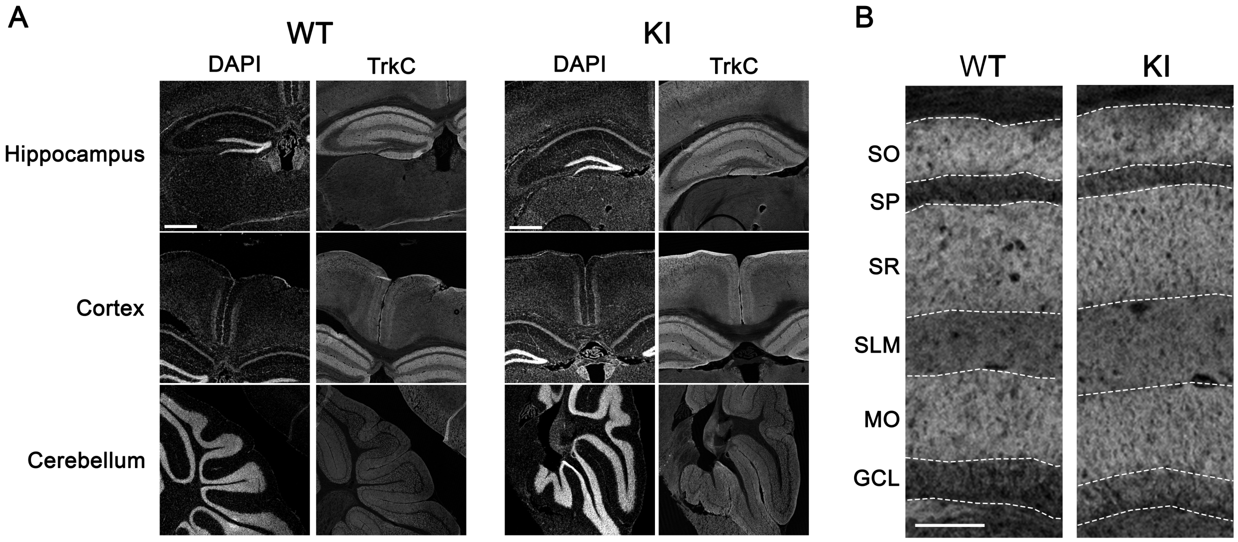

Appendix Figure S2. TrkC proteins are highly expressed in the hippocampus
(**A**) Immunolabeling for TrkC with nuclear DAPI staining in the hippocampus (upper), the cortex (middle) and the cerebellum (lower) in TrkC KI and WT mice.
(**B**) High magnification images showing TrkC immunosignals in the hippocampal CA1 region in TrkC KI mice and WT mice. TrkC is highly expressed in the stratum oriens (SO) and the stratum radiatum (SR). SP: stratum pyramidale, SLM: stratum lacunosum-moleculare, MO: dentate gyrus molecular layer, GCL: dentate gyrus granule cell layer.
Scale bars: 500 μm in (**A**) and 100 μm in (**B**)

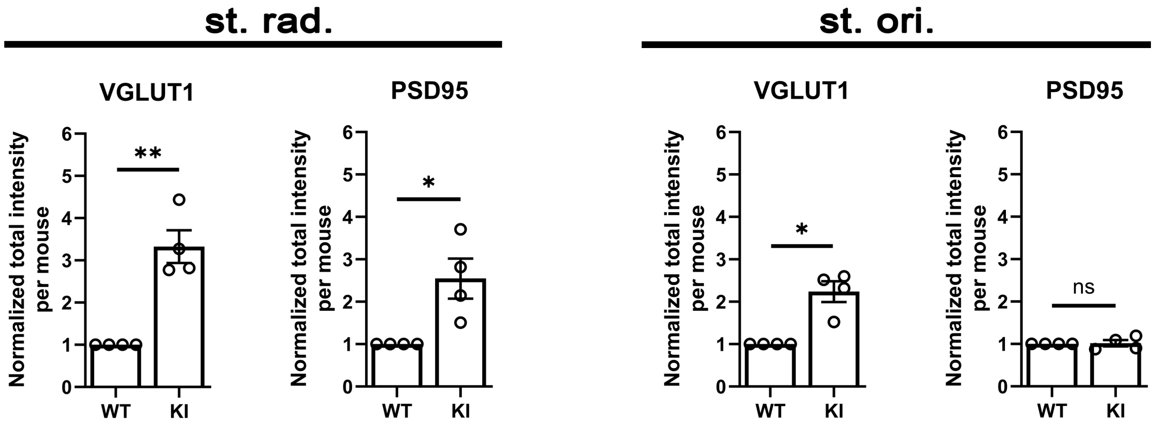

Appendix Figure S3. Total intensity of VGLUT1 and PSD-95 per mice

Quantification of total intensity of VGLUT1 and PSD-95 puncta in the st. rad. and st. ori. per mouse (related to **Fig. 2B,C**). n = 4 mice for each genotype. One sample t-tests, **p = 0.0093 for VGLUT1 and *p = 0.046 for PSD-95 in the st. rad., and *p = 0.0151 for VGLUT1 and p = 0.84 for PSD-95 in the st. ori..

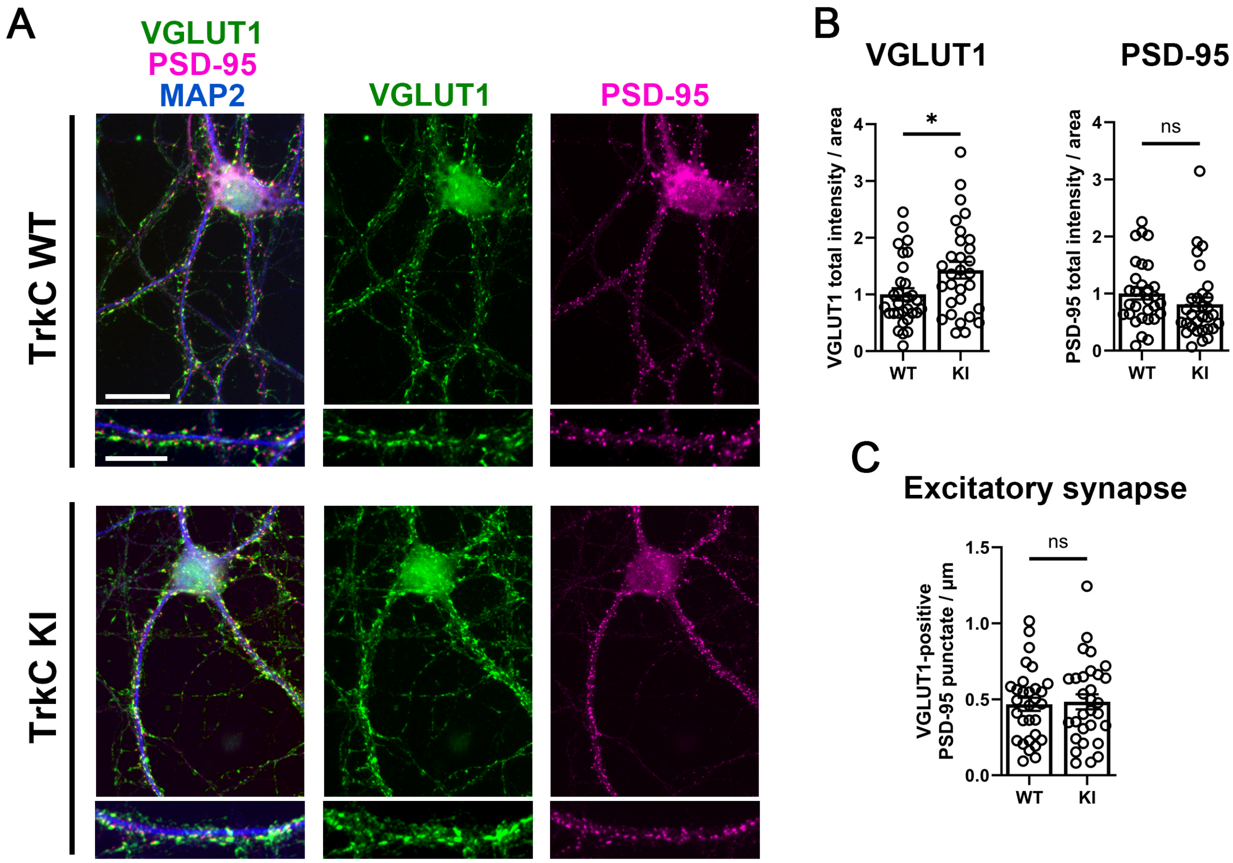

Appendix Figure S4. Cultured hippocampal neurons derived from TrkC KI mice show increased VGLUT1 puncta intensity and no change in excitatory synapse number

(**A**) Triple immunolabeling of VGLUT1, PSD-95 and MAP2 in cultured hippocampal neurons at 21 days *in vitro* from TrkC KI and WT littermate pups. Scale bars: 20 µm and 10 µm for lower and higher magnification images, respectively.

(**B**) Quantification of total intensity of all VGLUT1 and PSD-95 puncta per dendrite segment area. VGLUT1 intensity was significantly higher in TrkC KI than in WT cultured neurons. Student's t-tests, *p = 0.023 for VGLUT1 and p = 0.23 for PSD-95.

(**C**) Quantification of the number of VGLUT1-positive PSD-95 puncta per dendrite length. Excitatory synapse number was comparable in TrkC KI and WT neurons. Student's t-test, p = 0.80.

Data are presented as mean ± SEM. n = 30 neurons per each condition. ns: not significant.

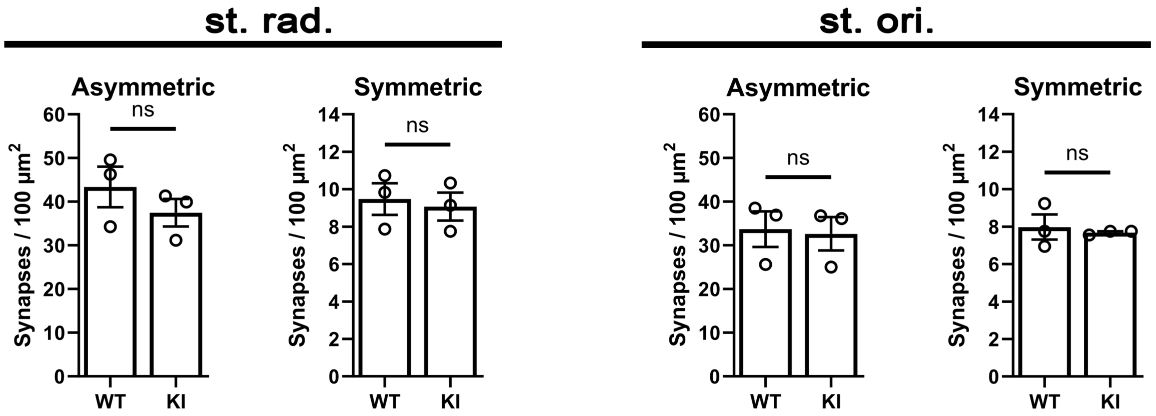

Appendix Figure S5. Number of asymmetric and symmetric synapses per mice
Quantification of the number of asymmetric and symmetric synapses in the st. rad. and the st. ori. per mouse (related to **Fig. 3B**). n = 3 mice for each genotype. Student's t-test, p = 0.35 and p = 0.74 for asymmetric and symmetric synapses in the st. rad., and p = 0.86 and p = 0.68 for asymmetric and symmetric synapses in the st. ori., respectively.

A

Phosphorylation site	Isolated phospho-peptide sequence	P-value	Ratio (KI/WT)
S20	[R].GAGPCSPGLER.[A]	**0.0076**	2.200
S30	[R].SVGELR.[L]	**0.0080**	1.412
S195	[R].YSWVQLAGHTGSFK.[A]	**0.0413**	1.224
T83	[R].AAPAPVIPQLTVTSEEDVTPASPGPPDQEGNWLPAAGSHLQQPR.[R]	0.0723	1.218
S30	[R].RSVGELR.[L]	0.2213	1.194
S135	[R].LSTSSLSSTGSSSLLEDSEDDLLSDSESR.[S]	0.2483	1.128
S119;S135	[R].RLSTSSLSSTGSSSLLEDSEDDLLSDSESR.[S]	0.1168	1.125
S185	[K].RYSWVQLAGHTGSFK.[A]	0.4112	1.093
S94	[R].AAPAPVIPQLTVTSEEDVTPASPGPPDQEGNWLPAAGSHLQQPR.[R]	0.5269	1.074
S119	[R].RLSTSSLSSTGSSSLLEDSEDDLLSDSESR.[S]	0.3250	1.047

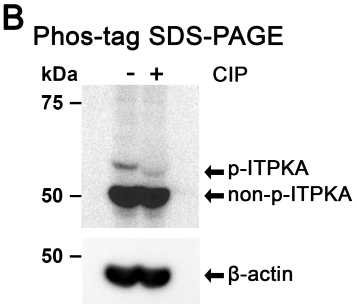

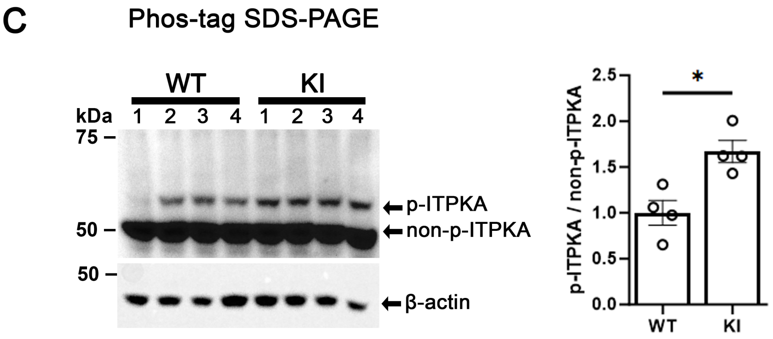

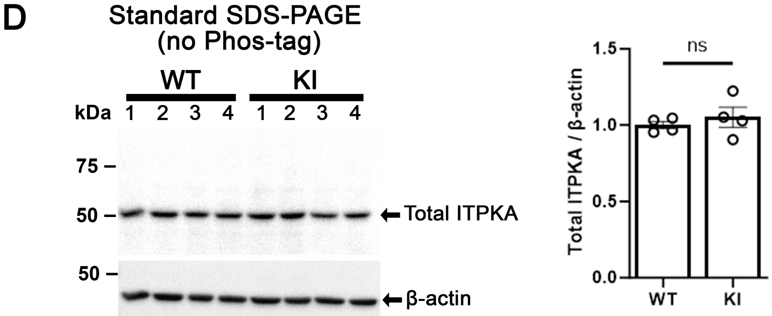

Appendix Figure S6. TrkC KI mice show an increase in phosphorylated-ITPKA expression
(**A**) Hippocampal phosphoproteomic analysis in Figure 5 identified 10 phosphorylation sites in ITPKA with increased phosphorylation in TrkC KI mice.
(**B**) Phosphorylated-ITPKA (p-ITPKA) and non-phosphorylated-ITPKA (non-p-ITPKA) migrate as two distinct bands when run on Phos-Tag SDS-PAGE. Treating the same tissue lysate with calf-intestinal alkaline phosphatase (CIP) diminishes the phosphorylated-ITPKA band.
(**C**) Representative immunoblots and quantifications for the ratio of p-ITPKA to non-p-ITPKA in hippocampal total lysate samples using Phos-Tag SDS-PAGE. Non-p-ITPKA quantifications were carried out using membranes captured at lower exposure to avoid saturation of bands (blot provided in source data). Student's t-tests, * $p = 0.01$.
(**D**) Representative immunoblots and quantifications for total ITPKA expression in hippocampal total lysate samples using standard SDS-PAGE. Student's t-tests, $p = 0.48$.
All quantifications were normalized to β-actin loading controls and average of WT samples. $n = 4$ total hippocampal lysate samples per genotype. Data are presented as mean ± SEM. ns: not significant.

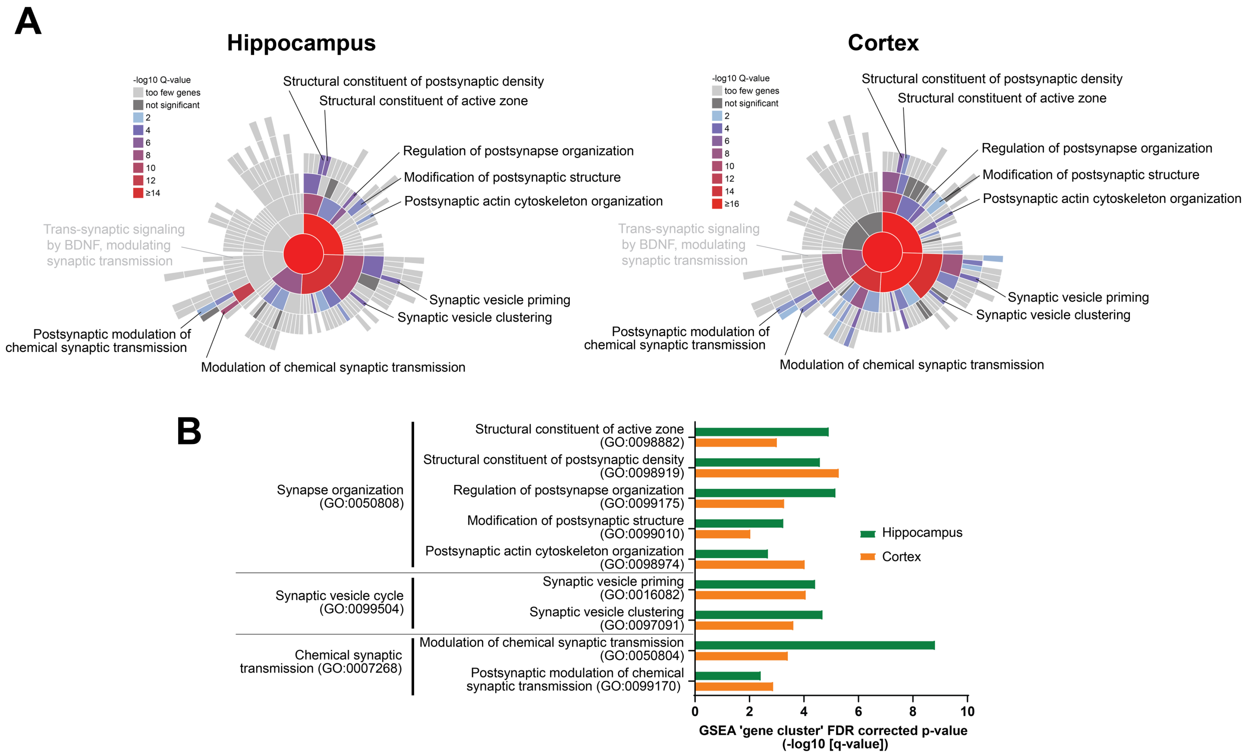

Appendix Figure S7. SynGO analysis of hippocampal and cortical phosphoproteomic data
(**A**) SynGO analysis of the phosphorylated molecules altered in the hippocampus (left) and the cortex (right) in TrkC KI mice for Biological Process. The SynGO term "trans-synaptic signaling by BDNF, modulating synaptic transmission (GO:0099183)" (in gray) was not detected as a significantly enriched SynGO term.
(**B**) The significantly enriched SynGO terms common between the hippocampus and the cortex in the above SynGO analysis.

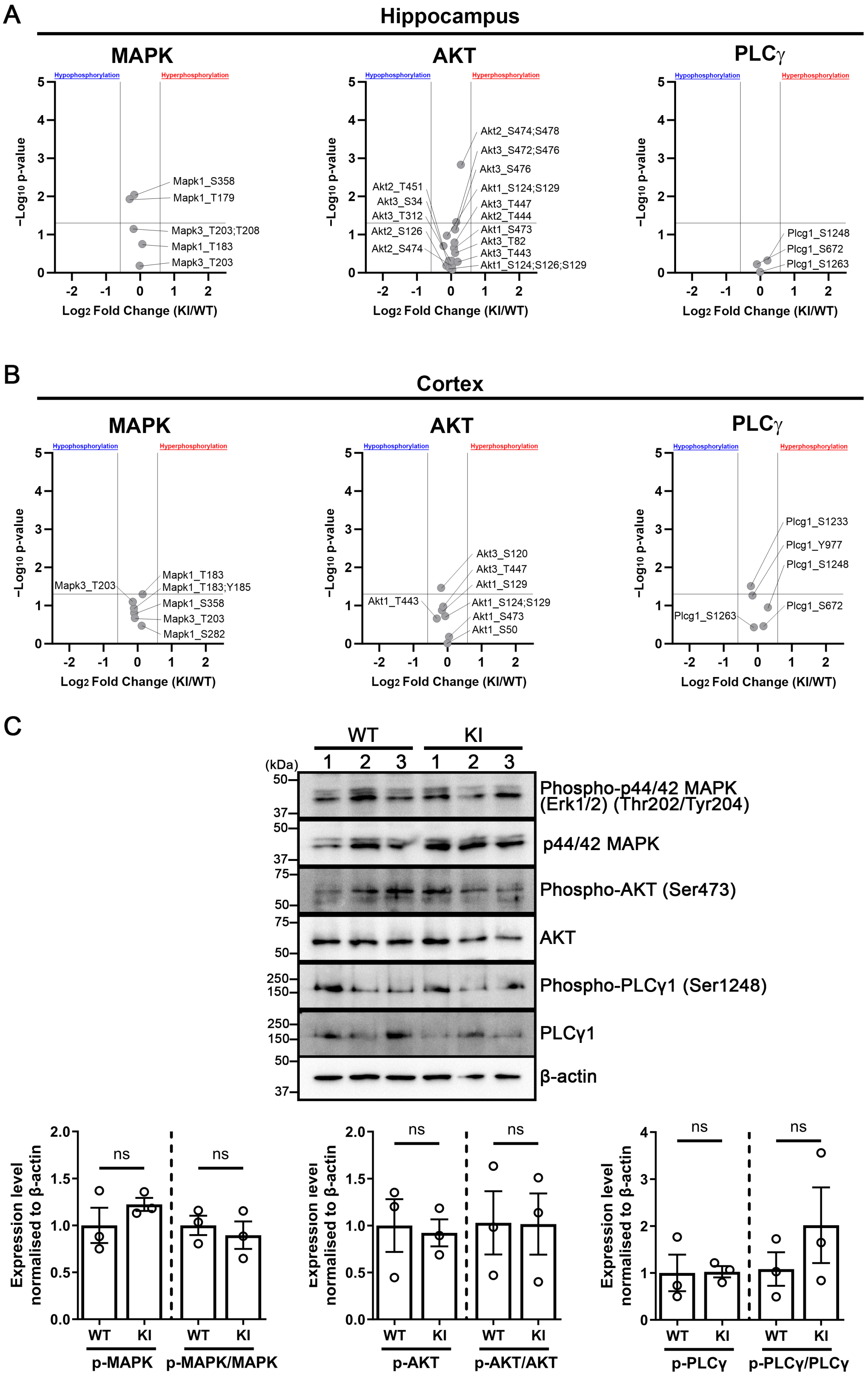
A
Hippocampus
MAPK
AKT
PLCγ
Hypophosphorylation
Hyperphosphorylation
−Log10 p-value
Log2 Fold Change (KI/WT)
Mapk1_S358
Mapk1_T179
Mapk3_T203;T208
Mapk1_T183
Mapk3_T203
Akt2_S474;S478
Akt3_S472;S476
Akt3_S476
Akt1_S124;S129
Akt3_T447
Akt2_T444
Akt1_S473
Akt3_T82
Akt3_T443
Akt1_S124;S126;S129
Akt2_T451
Akt3_S34
Akt3_T312
Akt2_S126
Akt2_S474
Plcg1_S1248
Plcg1_S672
Plcg1_S1263
B
Cortex
Mapk3_T203
Mapk1_T183
Mapk1_T183;Y185
Mapk1_S358
Mapk3_T203
Mapk1_S282
Akt3_S120
Akt3_T447
Akt1_S129
Akt1_T443
Akt1_S124;S129
Akt1_S473
Akt1_S50
Plcg1_S1233
Plcg1_Y977
Plcg1_S1248
Plcg1_S1263
Plcg1_S672
C
WT
KI
(kDa)
Phospho-p44/42 MAPK (Erk1/2) (Thr202/Tyr204)
p44/42 MAPK
Phospho-AKT (Ser473)
AKT
Phospho-PLCγ1 (Ser1248)
PLCγ1
β-actin
Expression level normalised to β-actin
ns
p-MAPK
p-MAPK/MAPK
p-AKT
p-AKT/AKT
p-PLCγ
p-PLCγ/PLCγ

Appendix Figure S8. Volcano plots of hippocampal and cortical phosphoproteomic data showing MAPK, AKT and PLCγ

(**A**, **B**) The phosphorylation of molecules in neurotrophin signaling pathways such as Mapk1/Erk2, Mapk3/Erk1, AKT1,2,3 and PLCγ (Plcg1) was not significantly altered in the TrkC KI hippocampus (**A**) or cortex (**B**). Differential protein expression was determined by a criteria of absolute fold change (|FC|) greater than 1.5 and p-value less than 0.05.

(**C**) Immunoblots and quantifications for some of the phosphorylation sites and total expression of p-44/42 MAPK (Mapk1/3), AKT and PLCγ in hippocampal total lysates. Quantification bar graphs show protein phosphorylation (left) and relative phosphorylation ratio to total expression (right) normalized to β-actin loading controls and average of WT samples. n = 3 total hippocampal lysate samples per genotype. Student's t-tests, p = 0.32 for p-MAPK, p = 0.59 for p-MAPK/MAPK, p = 0.82 for p-AKT, p = 0.98 for p-AKT/AKT, p = 0.95 for p-PLCγ and p = 0.35 for p-PLCγ/PLCγ. Data are presented as mean ± SEM. ns: not significant.

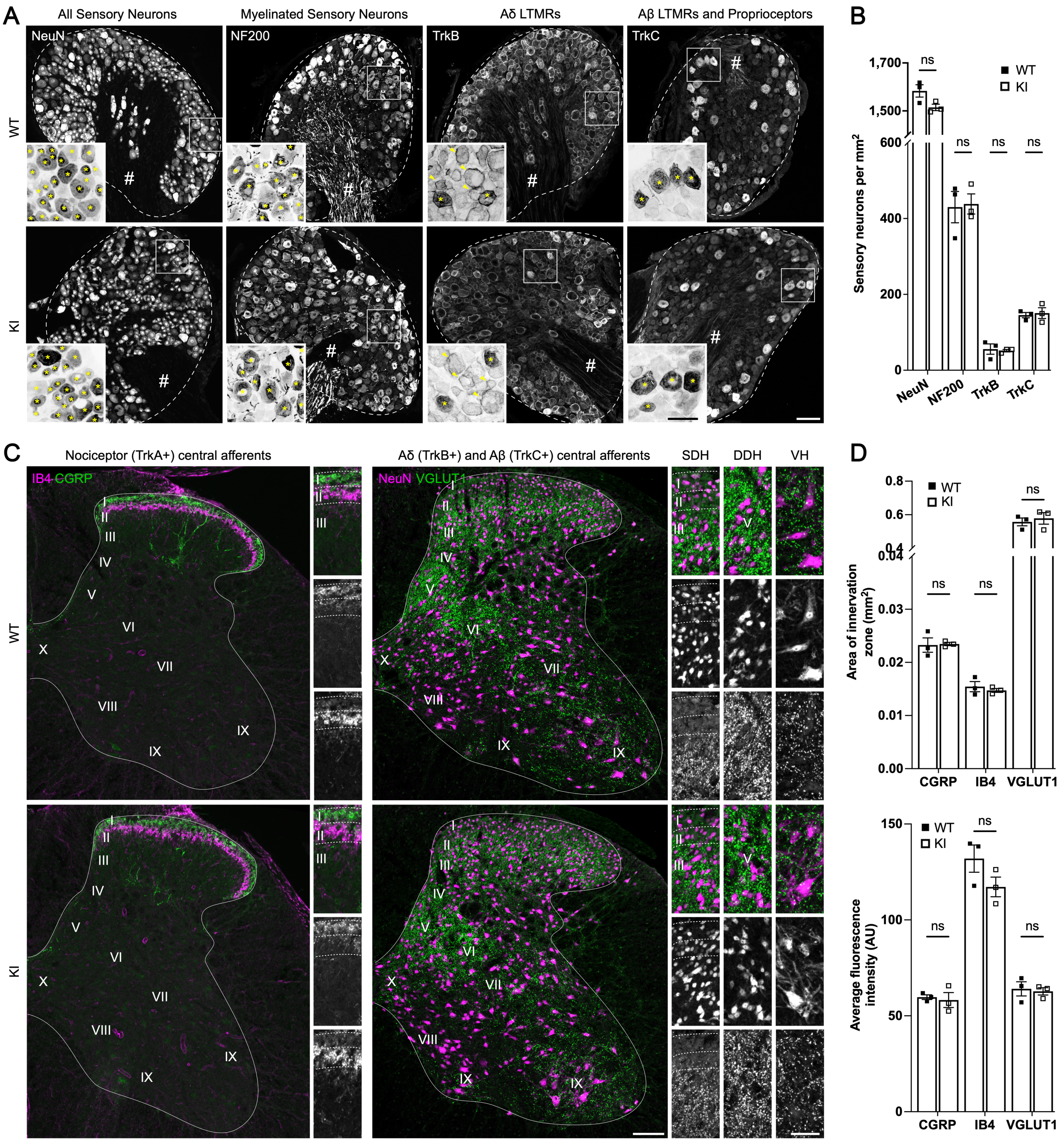

Appendix Figure S9. Staining for neuronal markers in TrkC KI mice show normal sensory neuron morphology in both DRG and spinal cord slices

(**A**) Immunohistochemical labeling of lumbar dorsal root ganglia (DRG) (L3 to L6) for all sensory neurons (pan-neuronal marker NeuN), myelinated sensory neurons (NF200), Aδ low threshold mechanoreceptors (LTMR) (TrkB), and Aβ LTMRs and proprioceptors (TrkC). Scale bars: 50 μm and 100 μm for higher and lower magnification images, respectively.

(**B**) Quantification of the number of sensory neurons in DRG with immunoreactivity for NeuN, NF200, TrkB and TrkC. Each data point represents the total obtained from 5 non-consecutive sections for each mouse, for a total of n = 3 mice per genotype.

(**C**) Immunoreactivity for central afferent markers detectible in the adult spinal cord. Nociceptor central afferents (TrkA+) were labeled by staining for CGRP (Calcitonin-Gene Related Peptide) and IB4 (Isolectin B4), and synaptic terminals of highly myelinated peripheral neurons were labeled by staining for VGLUT1. Key: SDH = Superficial Dorsal Horn, DDH = Deep Dorsal Horn, VH = Ventral horn. Scale bars: 50 µm and 100 µm for higher and lower magnification images, respectively.
(**D**) Quantification of the central afferent markers as detected by CGRP, IB4 and VGLUT1. Each data point represents the average for 5 non-consecutive sections obtained for each mouse, for a total of n = 3 mice per genotype.
Data were analyzed using Student's t-tests comparing WT and KI with the same staining conditions and are presented as mean ± SEM. ns: not significant.

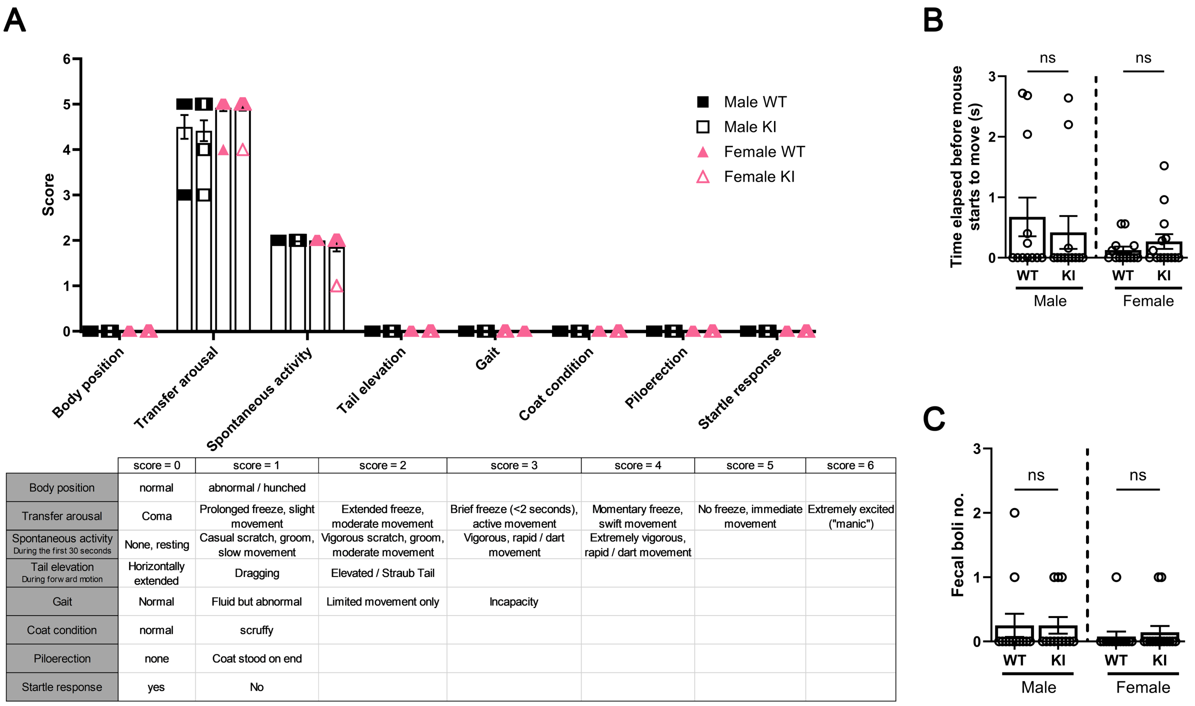

	score = 0	score = 1	score = 2	score = 3	score = 4	score = 5	score = 6
Body position	normal	abnormal / hunched					
Transfer arousal	Coma	Prolonged freeze, slight movement	Extended freeze, moderate movement	Brief freeze (<2 seconds), active movement	Momentary freeze, swift movement	No freeze, immediate movement	Extremely excited ("manic")
Spontaneous activity During the first 30 seconds	None, resting	Casual scratch, groom, slow movement	Vigorous scratch, groom, moderate movement	Vigorous, rapid / dart movement	Extremely vigorous, rapid / dart movement		
Tail elevation During forw ard motion	Horizontally extended	Dragging	Elevated / Straub Tail				
Gait	Normal	Fluid but abnormal	Limited movement only	Incapacity			
Coat condition	normal	scruffy					
Piloerection	none	Coat stood on end					
Startle response	yes	No					

Appendix Figure S10. General mouse observation and transfer behaviors

(**A**) Graph and score table for the general mouse screening for transfer behaviors not shown in Fig. 6 and Fig. 7. All "transfer behavior" tests were carried out by analyzing the initial transfer of mice from their home cage into an open field arena, except for the startle response, which was carried out using the fear-conditioning chamber. No significant differences were observed between WT and KI mice of the same sex for all tests, except for the jumping behavior reported in **Fig.7**. One-way ANOVA with post hoc Šídák's multiple comparison test.

(**B**) Time elapsed before mice started to move once placed in the open field arena during the transfer behaviors screening. Student's t-tests, p(male) = 0.55 and p(female) = 0.31.

(**C**) Number of fecal boli after 1 min in the open field arena during the screening. Student's t-tests, p(male) > 0.99 and p(female) = 0.60.

All data are presented as mean ± SEM. n = 12 WT male, 12 KI male, 13 WT female and 14 KI female littermate mice.

Appendix Tables

	GO.ID	pathway	enrichment	FDR	count
CC	**GO:0045202**	**synapse**	**14.74378686**	**1.80E-15**	**24**
	**GO:0098794**	**postsynapse**	**14.13913061**	**7.26E-15**	**19**
	**GO:0030054**	**cell junction**	**13.94916984**	**1.12E-14**	**26**
	**GO:0099572**	**postsynaptic specialization**	**9.99253804**	**1.02E-10**	**13**
	**GO:0098978**	**glutamatergic synapse**	**8.571060578**	**2.68E-09**	**13**
	**GO:0014069**	**postsynaptic density**	**7.73320616**	**1.85E-08**	**11**
	**GO:0032279**	**asymmetric synapse**	**7.543619027**	**2.86E-08**	**11**
	**GO:0098984**	**neuron to neuron synapse**	**7.178158608**	**6.64E-08**	**11**
	GO:0042995	cell projection	6.55480197	2.79E-07	20
	GO:0043005	neuron projection	6.144069754	7.18E-07	16
BP	**GO:0007267**	**cell-cell signaling**	**5.581844171**	**2.62E-06**	**17**
	**GO:0099536**	**synaptic signaling**	**5.229735021**	**5.89E-06**	**13**
	**GO:0007268**	**chemical synaptic transmission**	**4.431311815**	**3.7E-05**	**12**
	**GO:0098916**	**anterograde trans-synaptic signaling**	**4.431311815**	**3.7E-05**	**12**
	**GO:0099537**	**trans-synaptic signaling**	**4.394054503**	**4.04E-05**	**12**
	GO:0065008	regulation of biological quality	3.841435977	0.000144	20
	GO:0042391	regulation of membrane potential	3.778494456	0.000167	9
	GO:0007010	cytoskeleton organization	3.777467487	0.000167	14
	GO:0051234	establishment of localization	3.671308746	0.000213	24
	GO:0006996	organelle organization	3.277181094	0.000528	20
MF	GO:0008092	cytoskeletal protein binding	7.492133675	3.22E-08	15
	GO:0005516	calmodulin binding	6.303635954	4.97E-07	8
	GO:0019899	enzyme binding	4.256561978	5.54E-05	17
	GO:0019901	protein kinase binding	3.53997919	0.000288	10
	GO:0003779	actin binding	3.375068684	0.000422	8
	GO:0005515	protein binding	3.176286183	0.000666	34
	GO:0019900	kinase binding	3.11039851	0.000776	10
	GO:0005200	structural constituent of cytoskeleton	2.413420973	0.00386	4
	GO:0004683	calmodulin-dependent protein kinase activity	2.398655891	0.003993	3
	GO:0044877	protein-containing complex binding	2.380916106	0.00416	12

Appendix Table S1. GO enrichment analysis using phosphorylated molecules altered in both the hippocampus and cortex of TrkC KI mice

Hippocampus

	ID	Description	FDR
GO_BP	GO:0099536	synaptic signaling	4.15E-26
	GO:0007268	chemical synaptic transmission	6.31E-26
	GO:0098916	anterograde trans-synaptic signaling	6.31E-26
	GO:0099537	trans-synaptic signaling	8.71E-26
	GO:0007399	nervous system development	4.50E-24
	GO:0065008	regulation of biological quality	5.18E-23
	GO:0007267	cell-cell signaling	6.18E-23
	GO:0050808	synapse organization	6.69E-23
	GO:0051179	localization	1.89E-22
	GO:0034330	cell junction organization	2.34E-21
	**GO:0038179**	**neurotrophin signaling pathway**	**1**
	**GO:0000165**	**MAPK cascade**	**1**
KEGG	KEGG:04724	Glutamatergic synapse	0.001052275
	KEGG:04720	Long-term potentiation	0.007582829
	KEGG:04020	Calcium signaling pathway	0.020704352
	KEGG:04024	cAMP signaling pathway	0.073633719
	KEGG:04727	GABAergic synapse	0.279281961
	KEGG:04015	Rap1 signaling pathway	0.305403732
	KEGG:04934	Cushing syndrome	0.442398875
	KEGG:04144	Endocytosis	0.846279055
	**KEGG:04722**	**Neurotrophin signaling pathway**	**1**
	**KEGG:04151**	**PI3K-Akt signaling pathway**	**1**
	**KEGG:04010**	**MAPK signaling pathway**	**1**

Cortex

	ID	Description	FDR
GO_BP	GO:0051641	cellular localization	5.81E-50
	GO:0051179	localization	1.03E-48
	GO:0051234	establishment of localization	6.34E-45
	GO:0006810	transport	4.56E-44
	GO:0007399	nervous system development	9.07E-44
	GO:0050794	regulation of cellular process	1.77E-41
	GO:0034330	cell junction organization	3.52E-40
	GO:0032879	regulation of localization	2.23E-39
	GO:0051128	regulation of cellular component organization	1.23E-38
	GO:0050789	regulation of biological process	3.37E-37
	**GO:0000165**	**MAPK cascade**	**0.637265153**
	**GO:0038179**	**neurotrophin signaling pathway**	**1**
KEGG	KEGG:04144	Endocytosis	1.58E-07
	KEGG:04724	Glutamatergic synapse	2.62334E-05
	KEGG:04727	GABAergic synapse	5.45389E-05
	KEGG:04721	Synaptic vesicle cycle	6.33787E-05
	KEGG:04015	Rap1 signaling pathway	0.000378992
	KEGG:05033	Nicotine addiction	0.000795578
	KEGG:04012	ErbB signaling pathway	0.001216435
	KEGG:04072	Phospholipase D signaling pathway	0.00392544
	KEGG:04720	Long-term potentiation	0.005483843
	KEGG:03040	Spliceosome	0.006987117
	**KEGG:04010**	**MAPK signaling pathway**	**0.504724309**
	**KEGG:04151**	**PI3K-Akt signaling pathway**	**1**
	**KEGG:04722**	**Neurotrophin signaling pathway**	**1**

Appendix Table S2. GO enrichment analysis and KEGG analysis using phosphorylated molecules altered in the TrkC KI hippocampus and cortex separately

Common molecules in Fig. 6d	SFARI GENE SCORE	EAGLE SCORE	Associated Diseases	Associated syndroms
Shank3	1S	74.85	DD/NDD, BPD, ID, EPS, ASD	Phelan-McDermid syndrome, Rett syndrome-like phenotype, Pediatric Acute-Onset Neuropsychiatric Syndrome, Phelan-McDermid syndrome, ASD
Ddx3x	1S		DD/NDD, ADHD, ID, EP, EPS, ASD	
Kcnb1	1S		DD/NDD, ADHD, ASD	
Grin2b	1	29.65	DD/NDD, ADHD, ID, EP, EPS, ASD	
Ank2	1	10.8	ID	
Map1a	1			
Sptbn1	2S		ADHD, ASD, EPS	
Ksr2	2S		ADHD, ID, EPS, ASD	
Pclo	2			
Hecw2	2		DD/NDD, ADHD, ID, EPS, ASD	
Psd3	2		ID	
Top2b	2			
Cadps	2			
Gabrg2	3			
Rph3a	3			Angelman syndrome
Map2				
Sgsm2				
Gys1				
Slc4a4				
Oxr1				
Dnm1				
Phactr1				
Adcy2				
Map7d2				
Kiaa1109				
Ccny				
Stim1				
Limd2				
Prrc2c				
Map4				
Abcf2				
Dapk1				
Gsk3b				
Camkv				
Usp47				
Eef2				
Plppr2				
Tln2				
Fam171b				
Tnks1bp1				
Agfg1				
Scn3a				
U2surp				
Slc8a1				
Add2				
Itpka				
Slc7a2				
Myo18a				

DD/NDD, developmental delay/neurodevelopmental disorder; BPD, bipolar disorder; ID, intellectual disability; EPS, extrapyramidal symptoms; ASD, autism spectrum disorder; EP, epilepsy; ADHD, attention deficit with hyperactivity disorder

Appendix Table S3. SFARI score list of molecules with altered phosphorylation in both the hippocampus and cortex of TrkC KI mice

ID	phenotype	enrichment	FDR	count
HP:0002521	Hypsarrhythmia	2.880610228	0.001316	7
HP:0200134	Epileptic encephalopathy	2.669427748	0.002141	6
HP:0011198	EEG with generalized epileptiform discharges	1.730650647	0.018593	7
HP:0010844	EEG with multifocal slow activity	1.48096537	0.03304	4
HP:0100660	Dyskinesia	1.316390048	0.048263	6
**HP:0000729**	**Autistic behavior**	**1.306058343**	**0.049424**	**9**

Appendix Table S4. HPO analysis using phosphorylated molecules altered in both the hippocampus and cortex of TrkC KI mice
