# Supplementary material for: The TrkC-PTPσ complex governs synapse maturation and anxiogenic avoidance via synaptic protein phosphorylation
Source: EMBO J. 2024 Sep 27;43(22):5690–717. doi: 10.1038/s44318-024-00252-9 (PMC11574141; doi:10.1038/s44318-024-00252-9)
Supplement: Supplementary file 3 — Source data Fig. 1 [file 44318_2024_252_MOESM3_ESM.zip › Figure 1/1D/PCR sequences.docx]

PCR sequences for confirmation of point mutations transmission

Key:

**Bold and underlined** = fragments shown in Fig. 1D

Yellow highlight = point mutation sites

A = Adenine

C = Cytosine

G = Guanine

T = Thymine

M = Adenine / Cytosine

TrkC WT (WT/WT) PCR sequence

AYYGTCGTWTCACTCTGTCCTCCTAGATCTCCCAGAGATCAGTGTGAGCCACGTCAACCTGACTGTCCGAGAAGGAGACAATGCCGTGATCACTTGCAAT**GGCTCTGGCTCTCCTTTGCCTGATGTGGACTGGATAGTCACTGGGCTG**CAGTCCATCAACACCCACCAGGTAGGTATCCTATAACCAGGCCCATCAAGAGTTAAGGAATGAATATCCCTTAATTATAAGGAAGAGGGAGAGATTGTGATCATGTTCCTCTGGGAAGAAATGTATAGACCCAGATTCTGTGGAGATGTAAATTTCTGGTCATTTTGATCTTTGTTGTGGTGCTCAACCTTCTGTGCATTGGTTTCCTGTTTTTTAAAAAAATCACCCAGTTTTACCATTTACCATCCAATATTAAATTGAGGTTTACCTGAATTCCAAAGAATCTGGAGCACGGTCTGCGTGTTTTCAGTAGCTAGACTTCATTCAGCTTTTACTTCTTTTTTGGAAGTGTTAGGGGTTAGAAAAGGAAGGCATCCTTGTGTAAATAGTTGGTTATTGTCTTCTGATTGGGGTTTTCAGACCAATCTGAACTGGACCAATGTACATGCCATCAACAACCCCCYGKGGRRRAAA

TrkC Het (KI^(neo-)^/WT) PCR sequence

AYGYGTCGTWTCACTCTGTCCTCCTAGATCTCCCAGAGATCAGTGTGAGCCACGTCAACCTGACTGTCCGAGAAGGAGACAATGCCGTGATCACTTGCAAT**GGCTCTGGCTCTCCTTTGCCTGMTGTGGMCTGGATAGTCACTGGGCTG**CAGTCCATCAACACCCACCAGGTAGGTATCCTATAACCAGGCCCATCAAGAGTTAAGGAATGAATATCCCTTAATTATAAGGAAGAGGGAGAGATTGTGATCATGTTCCTCTGGGAAGAAATGTATAGACCCAGATTCTGTGGAGATGTAAATTTCTGGTCATTTTGATCTTTGTTGTGGTGCTCAACCTTCTGTGCATTGGTTTCCTGTTTTTTAAAAAAATCACCCAGTTTTACCATTTACCATCCAATATTAAATTGAGGTTTACCTGAATTCCAAAGAATCTGGAGCACGGTCTGCGTGTTTTCAGTAGCTAGACTTCATTCAGCTTTTACTTCTTTTTTGGAAGTGTTAGGGGTTAGAAAAGGAAGGCATCCTTGTGTAAATAGTTGGTTATTGTCTTCTGATTGGGGTTTTCAGACCAATCTGAACTGGACCAATGTACATGCCATCAACAMCCCCCYKGGGRRRAAATA

TrkC KI (KI^(neo-)^/ KI^(neo-)^) PCR sequence

AYYGTCGTWTCACTCTGTCCTCCTAGATCTCCCAGAGATCAGTGTGAGCCACGTCAACCTGACTGTCCGAGAAGGAGACAATGCCGTGATCACTTGCAAT**GGCTCTGGCTCTCCTTTGCCTGCTGTGGCCTGGATAGTCACTGGGCTG**CAGTCCATCAACACCCACCAGGTAGGTATCCTATAACCAGGCCCATCAAGAGTTAAGGAATGAATATCCCTTAATTATAAGGAAGAGGGAGAGATTGTGATCATGTTCCTCTGGGAAGAAATGTATAGACCCAGATTCTGTGGAGATGTAAATTTCTGGTCATTTTGATCTTTGTTGTGGTGCTCAACCTTCTGTGCATTGGTTTCCTGTTTTTTAAAAAAATCACCCAGTTTTACCATTTACCATCCAATATTAAATTGAGGTTTACCTGAATTCCAAAGAATCTGGAGCACGGTCTGCGTGTTTTCAGTAGCTAGACTTCATTCAGCTTTTACTTCTTTTTTGGAAGTGTTAGGGGTTAGAAAAGGAAGGCATCCTTGTGTAAATAGTTGGTTATTGTCTTCTGATTGGGGTTTTCAGACCAATCTGAACTGGACCAATGTACATGCCATCAACTAMCCCCYYKGKKRRAAAA
