# Supplementary figures and images for: The TrkC-PTPσ complex governs synapse maturation and anxiogenic avoidance via synaptic protein phosphorylation (part 1 of 2)
Source: EMBO J. 2024 Sep 27;43(22):5690–717. doi: 10.1038/s44318-024-00252-9 (PMC11574141; doi:10.1038/s44318-024-00252-9)

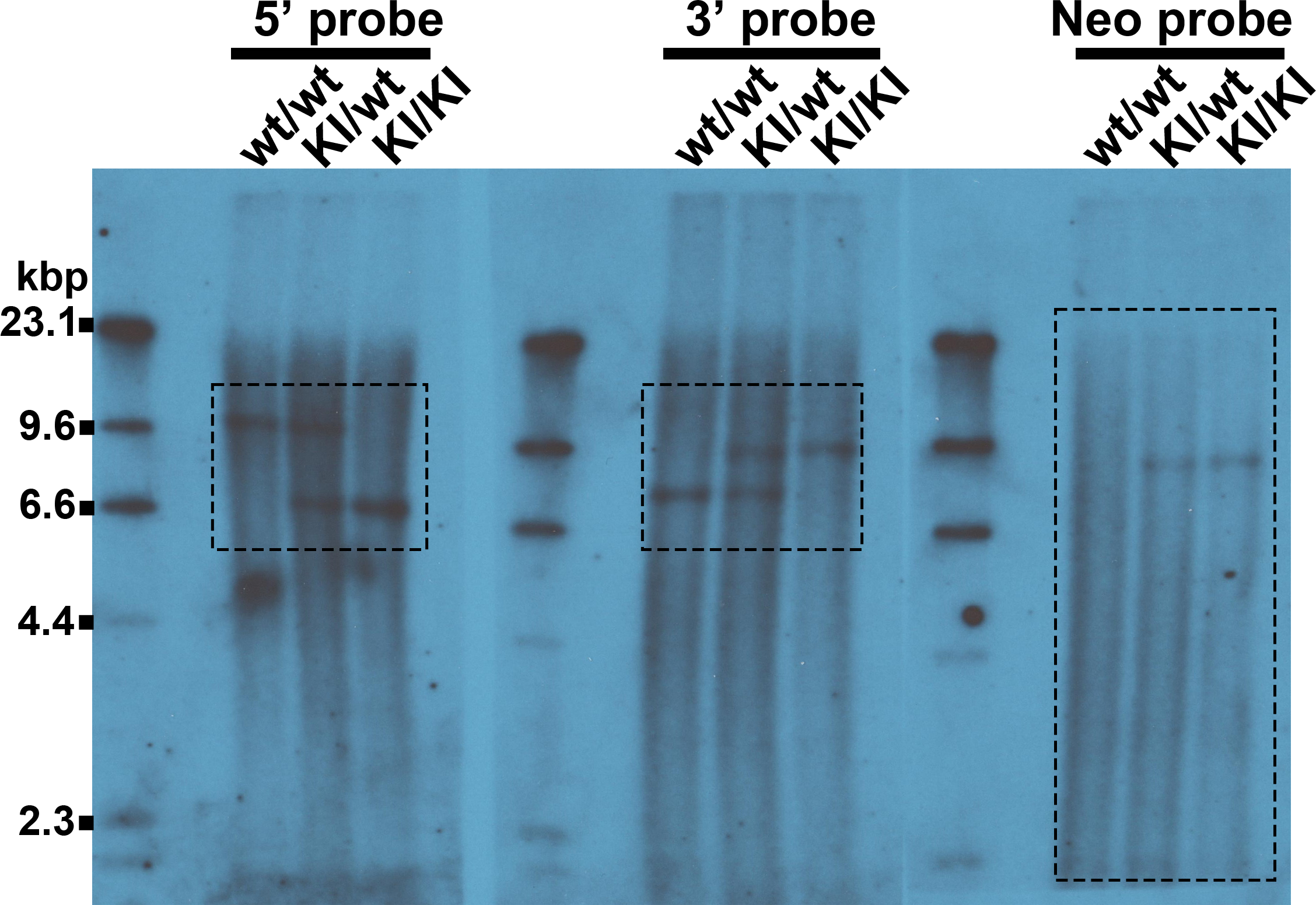

Supplement: Supplementary file 3 — Source data Fig. 1 [file 44318_2024_252_MOESM3_ESM.zip › Figure 1/1B/Southern blot annotated.png]

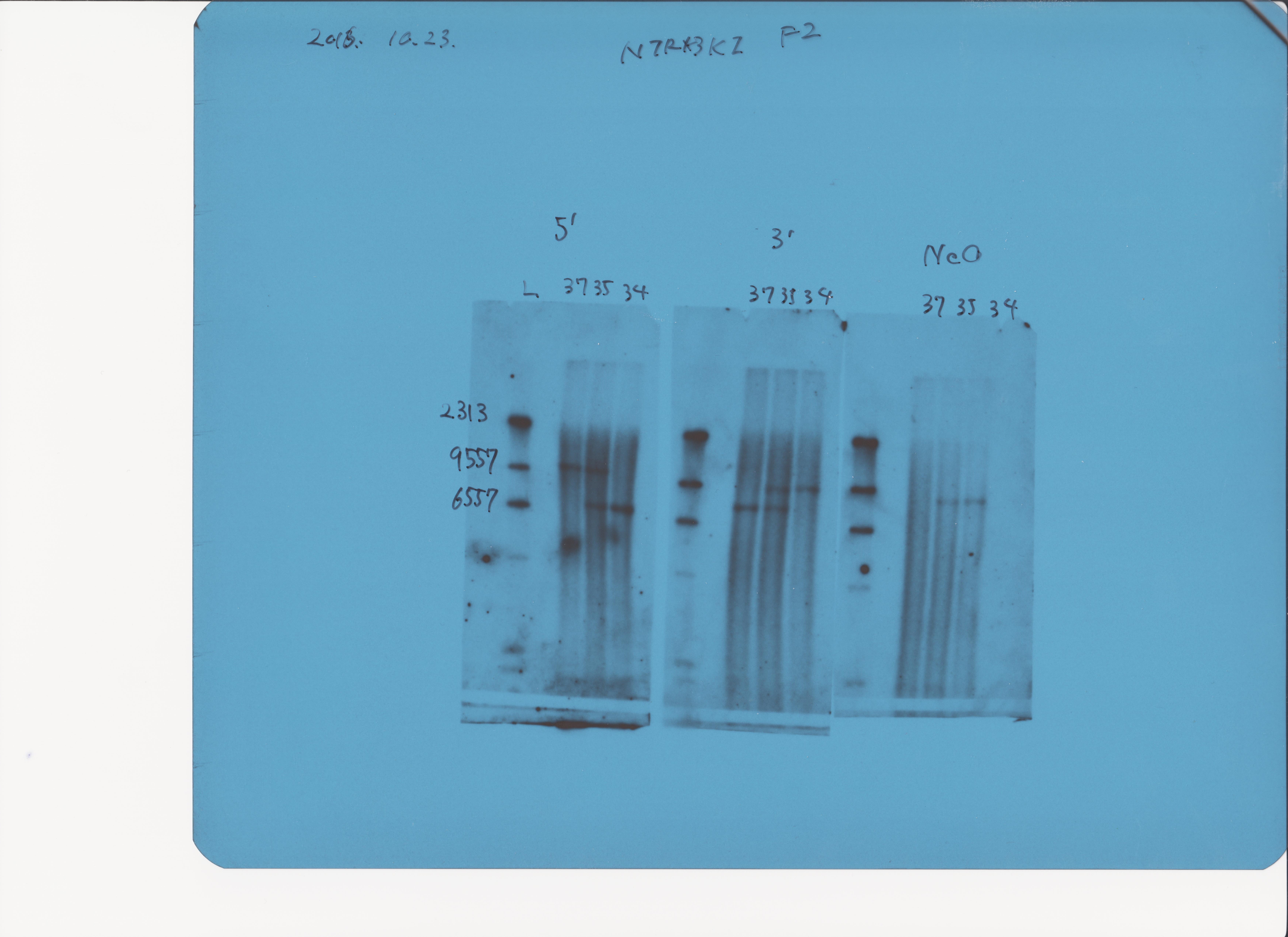

Supplement: Supplementary file 3 — Source data Fig. 1 [file 44318_2024_252_MOESM3_ESM.zip › Figure 1/1B/Southern blot.jpg]

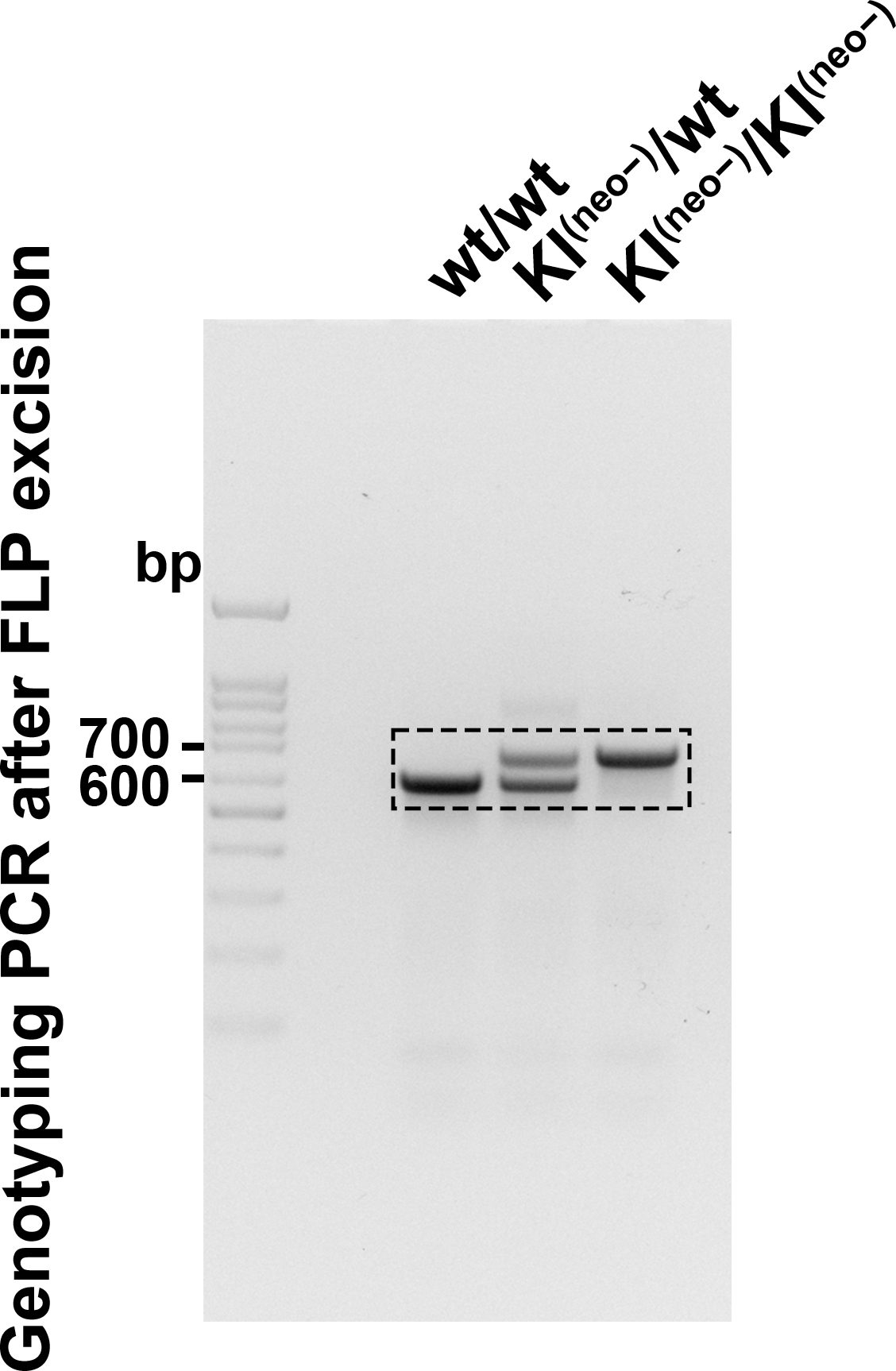

Supplement: Supplementary file 3 — Source data Fig. 1 [file 44318_2024_252_MOESM3_ESM.zip › Figure 1/1C/genotyping after FLP annotated.png]

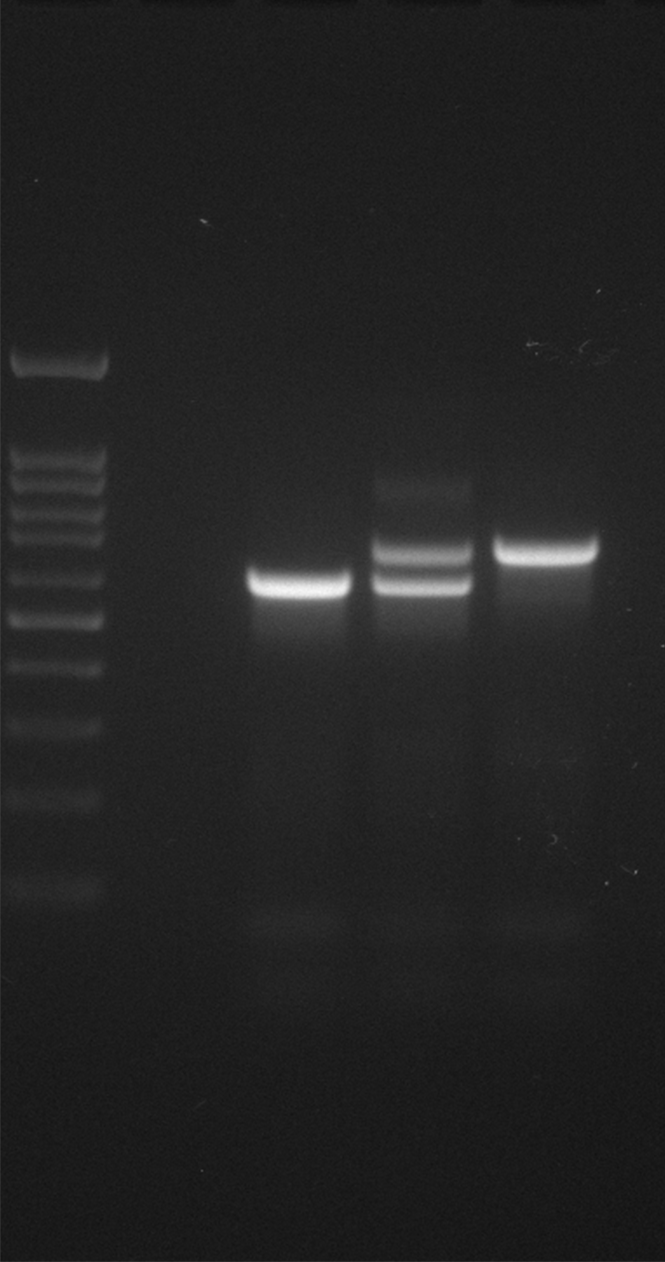

Supplement: Supplementary file 3 — Source data Fig. 1 [file 44318_2024_252_MOESM3_ESM.zip › Figure 1/1C/genotyping after FLP.tif]

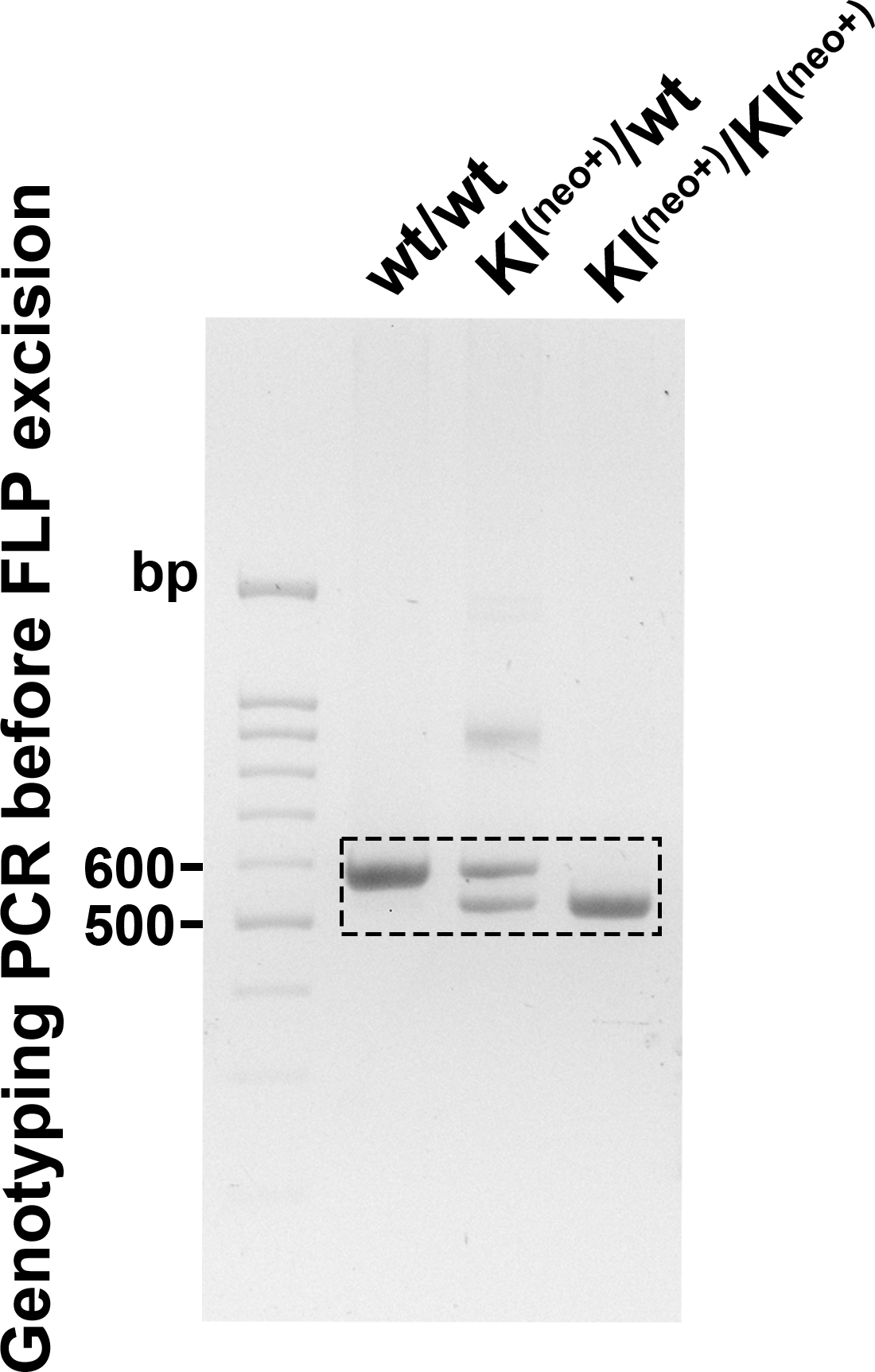

Supplement: Supplementary file 3 — Source data Fig. 1 [file 44318_2024_252_MOESM3_ESM.zip › Figure 1/1C/genotyping before FLP annotated.png]

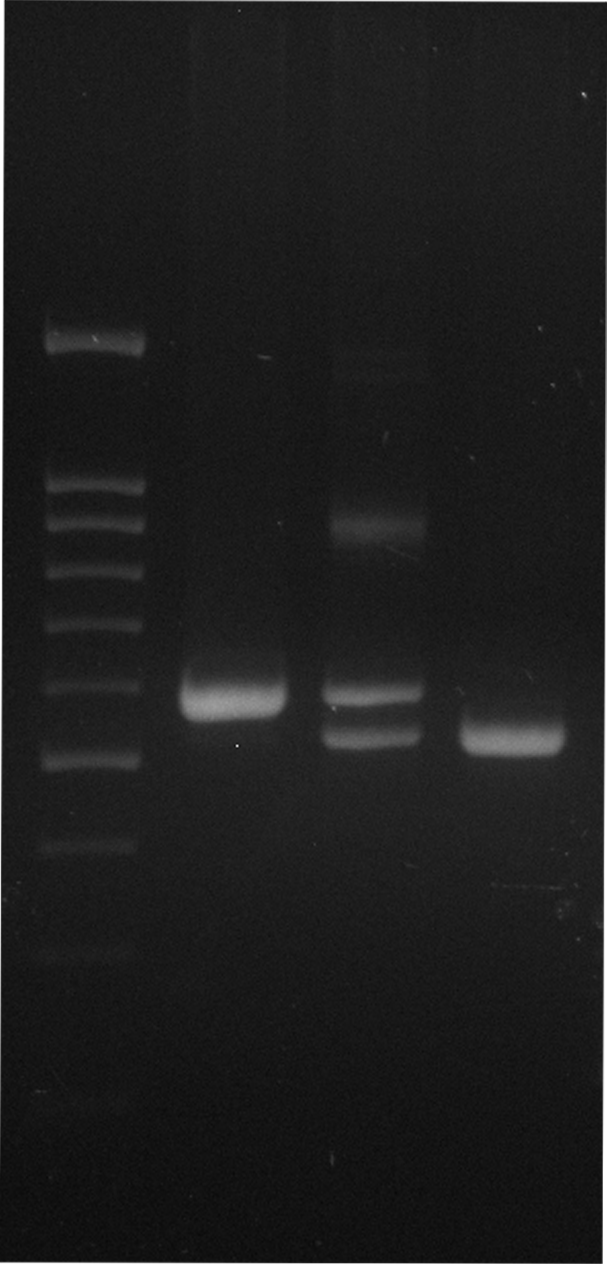

Supplement: Supplementary file 3 — Source data Fig. 1 [file 44318_2024_252_MOESM3_ESM.zip › Figure 1/1C/genotyping before FLP.tif]

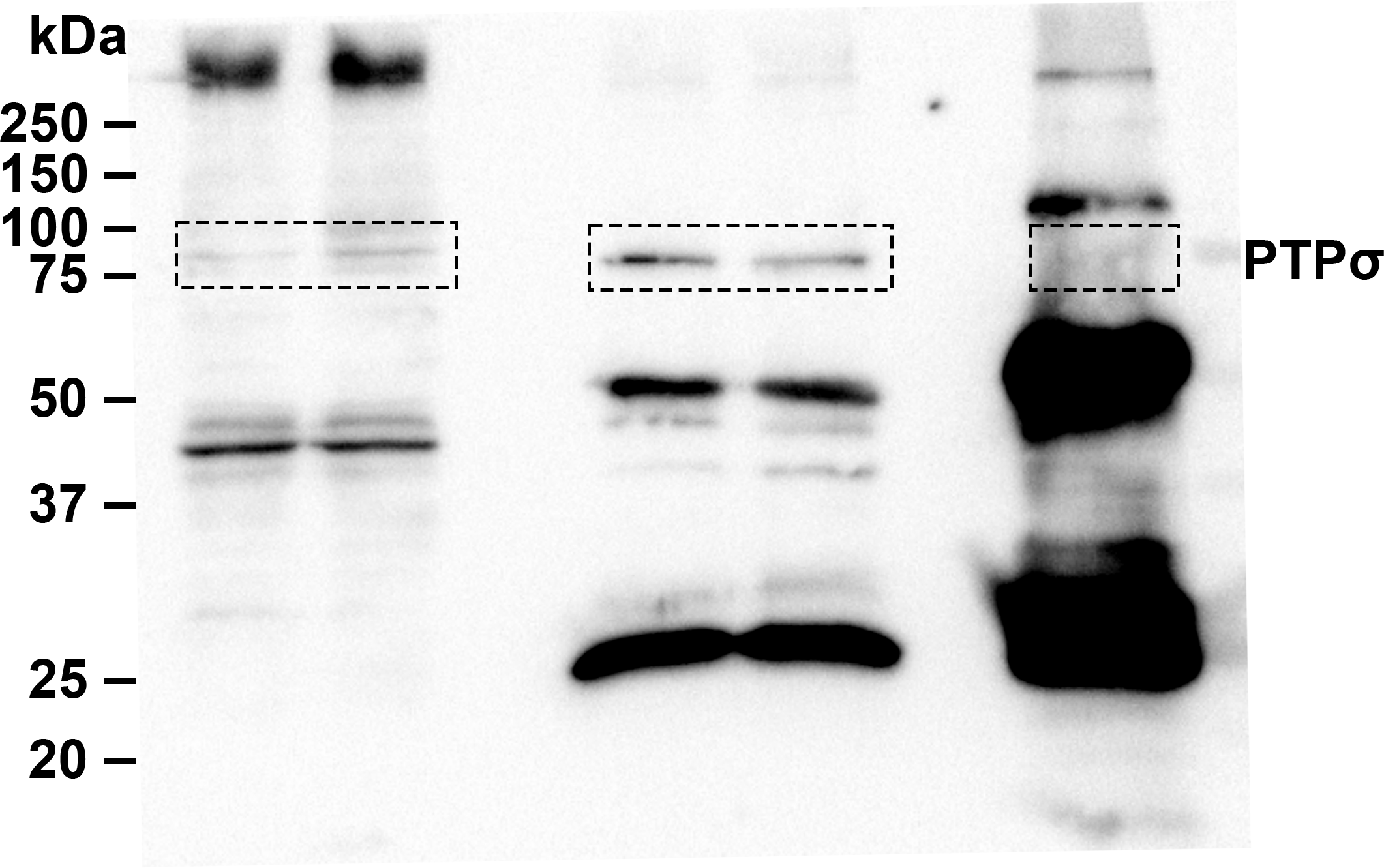

Supplement: Supplementary file 3 — Source data Fig. 1 [file 44318_2024_252_MOESM3_ESM.zip › Figure 1/1E/Co-IP PTPsigma WB annotated.png]

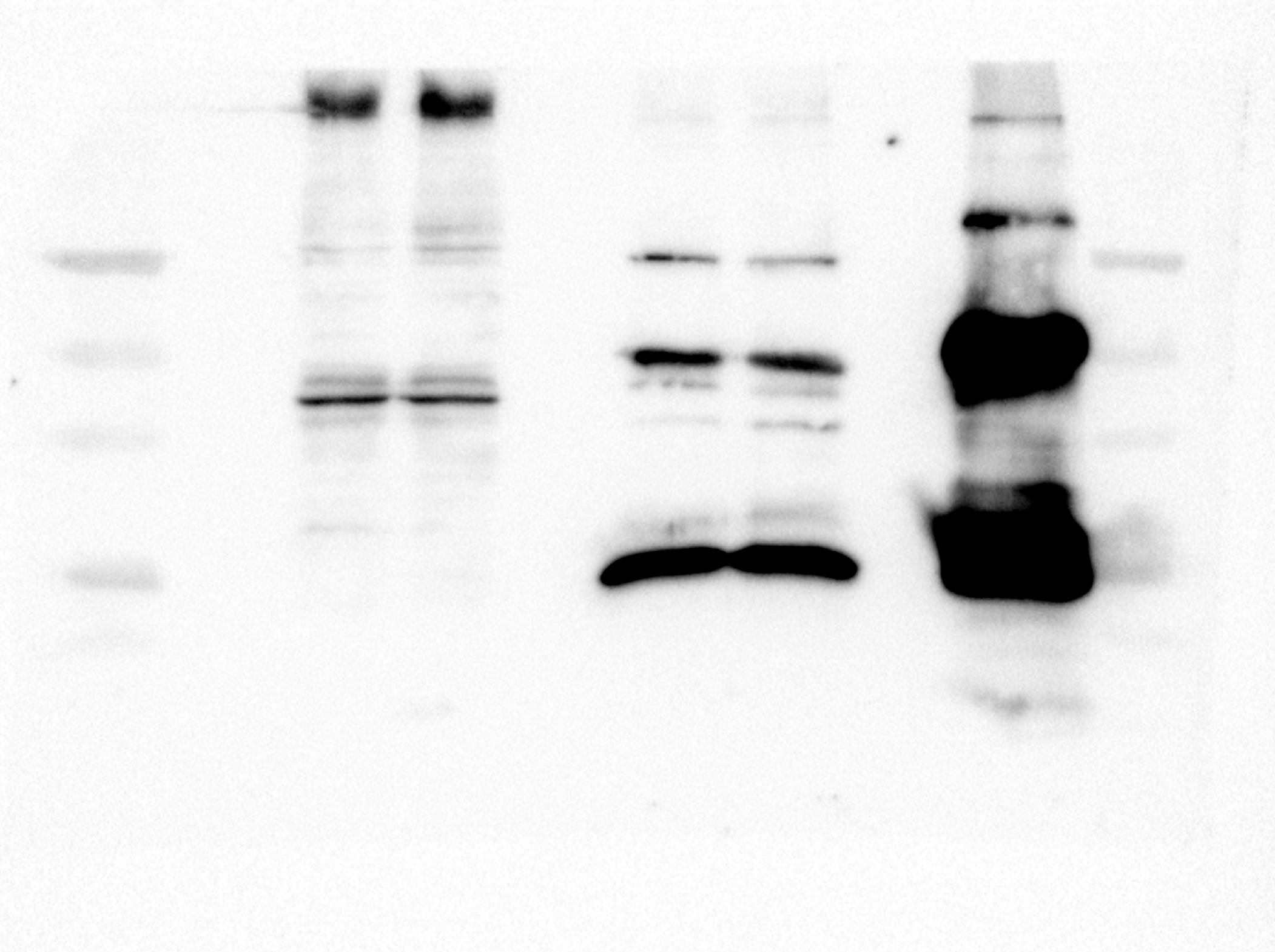

Supplement: Supplementary file 3 — Source data Fig. 1 [file 44318_2024_252_MOESM3_ESM.zip › Figure 1/1E/Co-IP PTPsigma WB.tif]

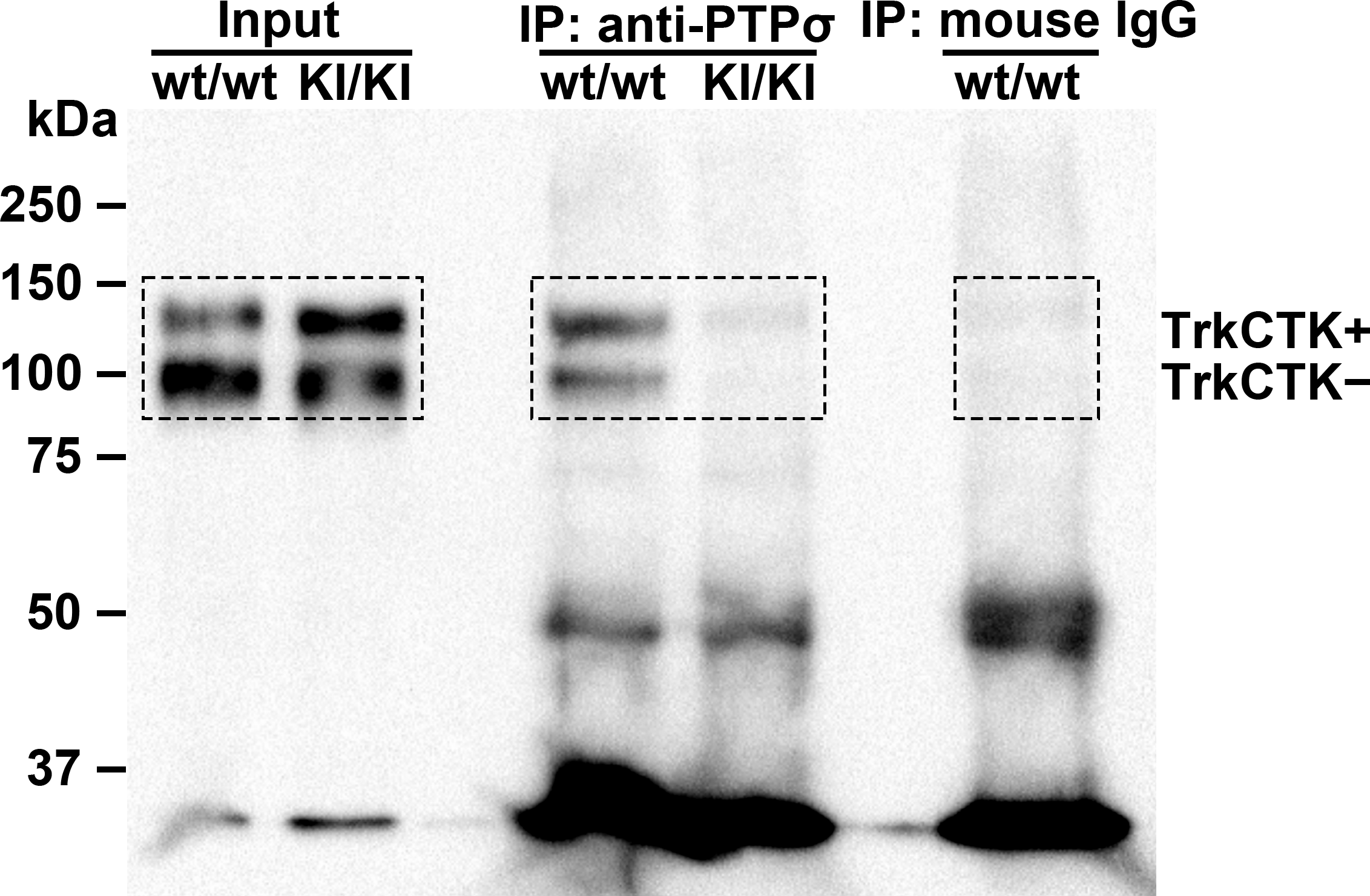

Supplement: Supplementary file 3 — Source data Fig. 1 [file 44318_2024_252_MOESM3_ESM.zip › Figure 1/1E/Co-IP TrkC WB annotated.png]

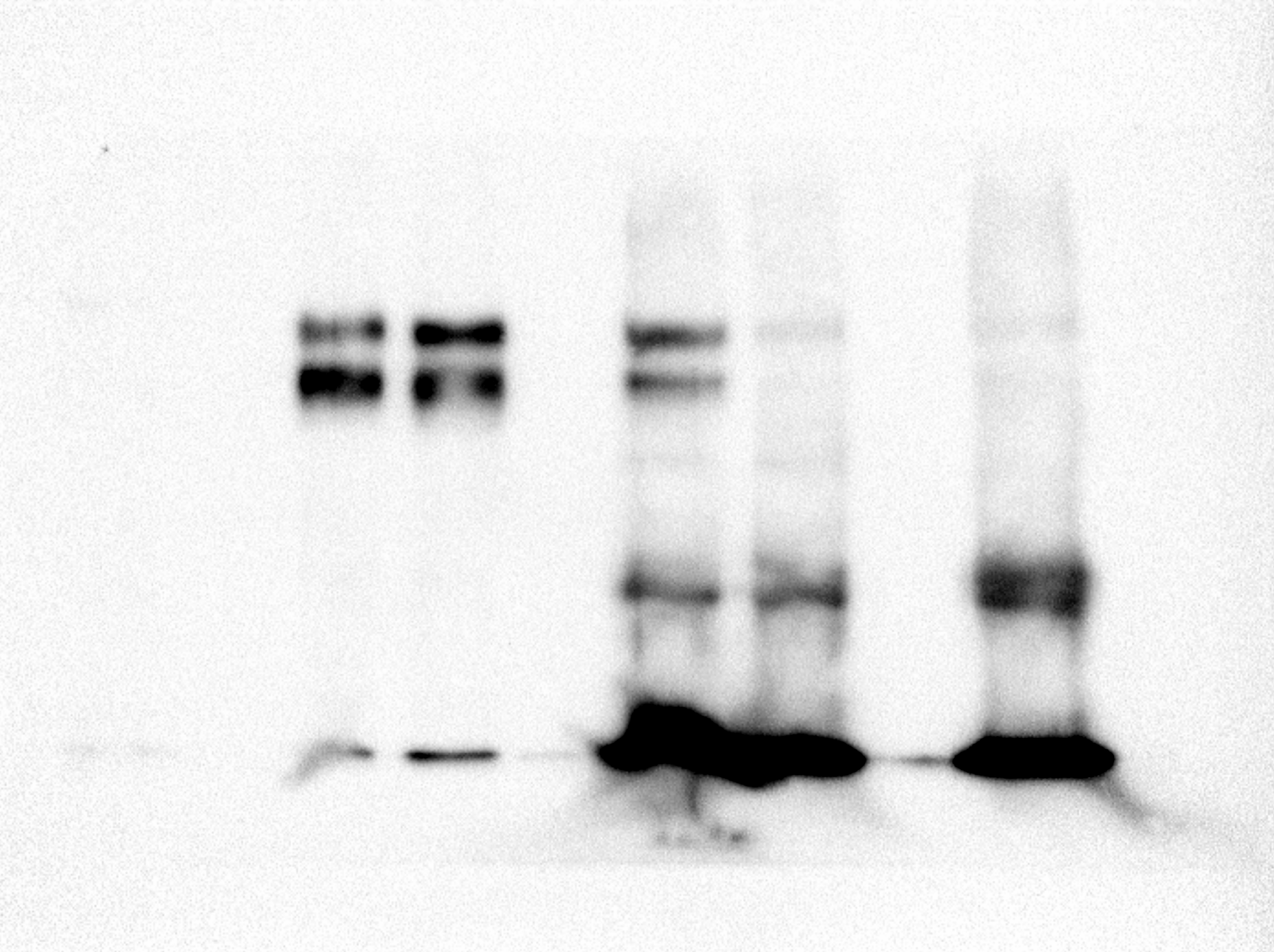

Supplement: Supplementary file 3 — Source data Fig. 1 [file 44318_2024_252_MOESM3_ESM.zip › Figure 1/1E/Co-IP TrkC WB.tif]

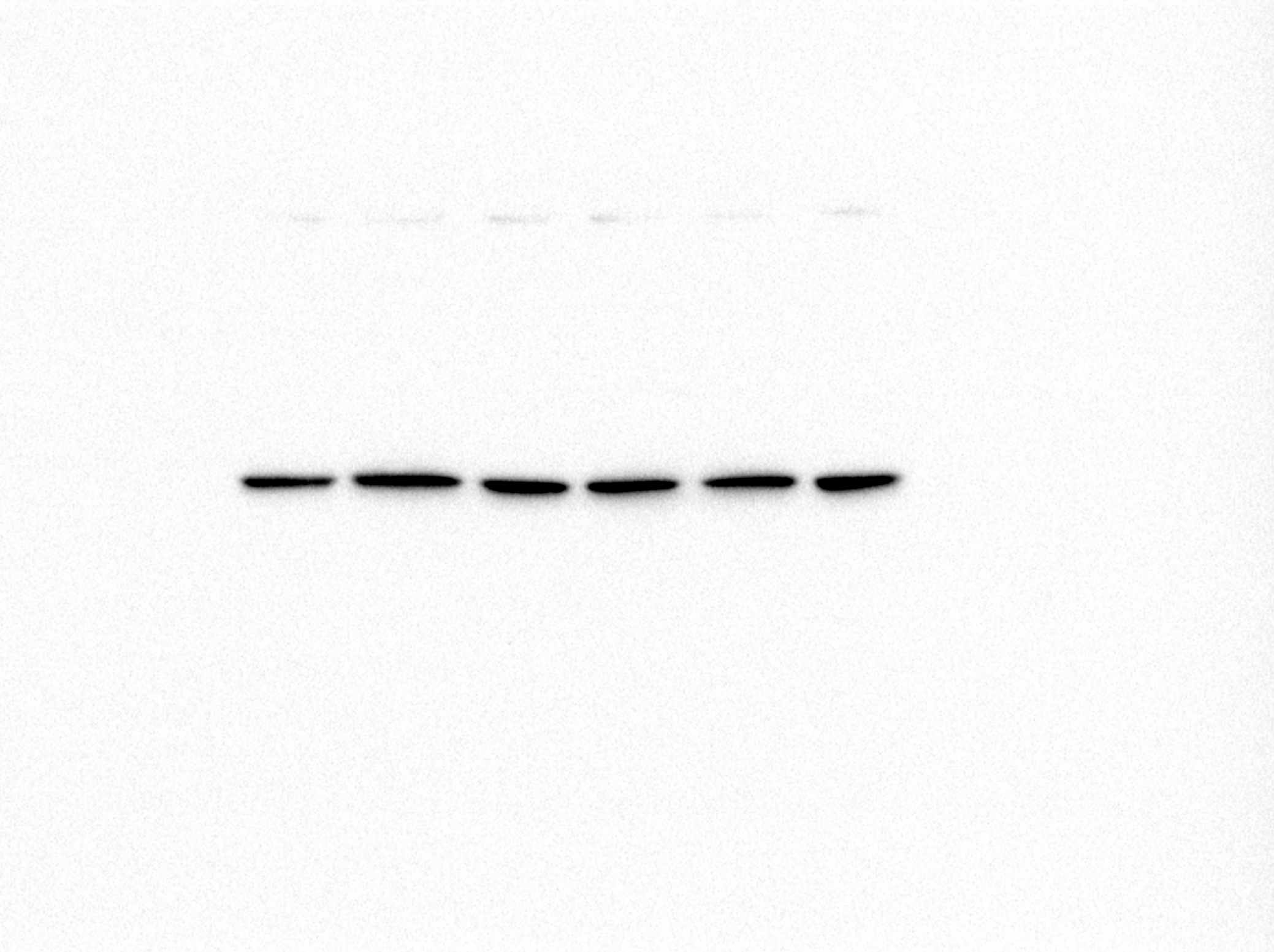

Supplement: Supplementary file 3 — Source data Fig. 1 [file 44318_2024_252_MOESM3_ESM.zip › Figure 1/1F/Figure1F_B-actin.tif]

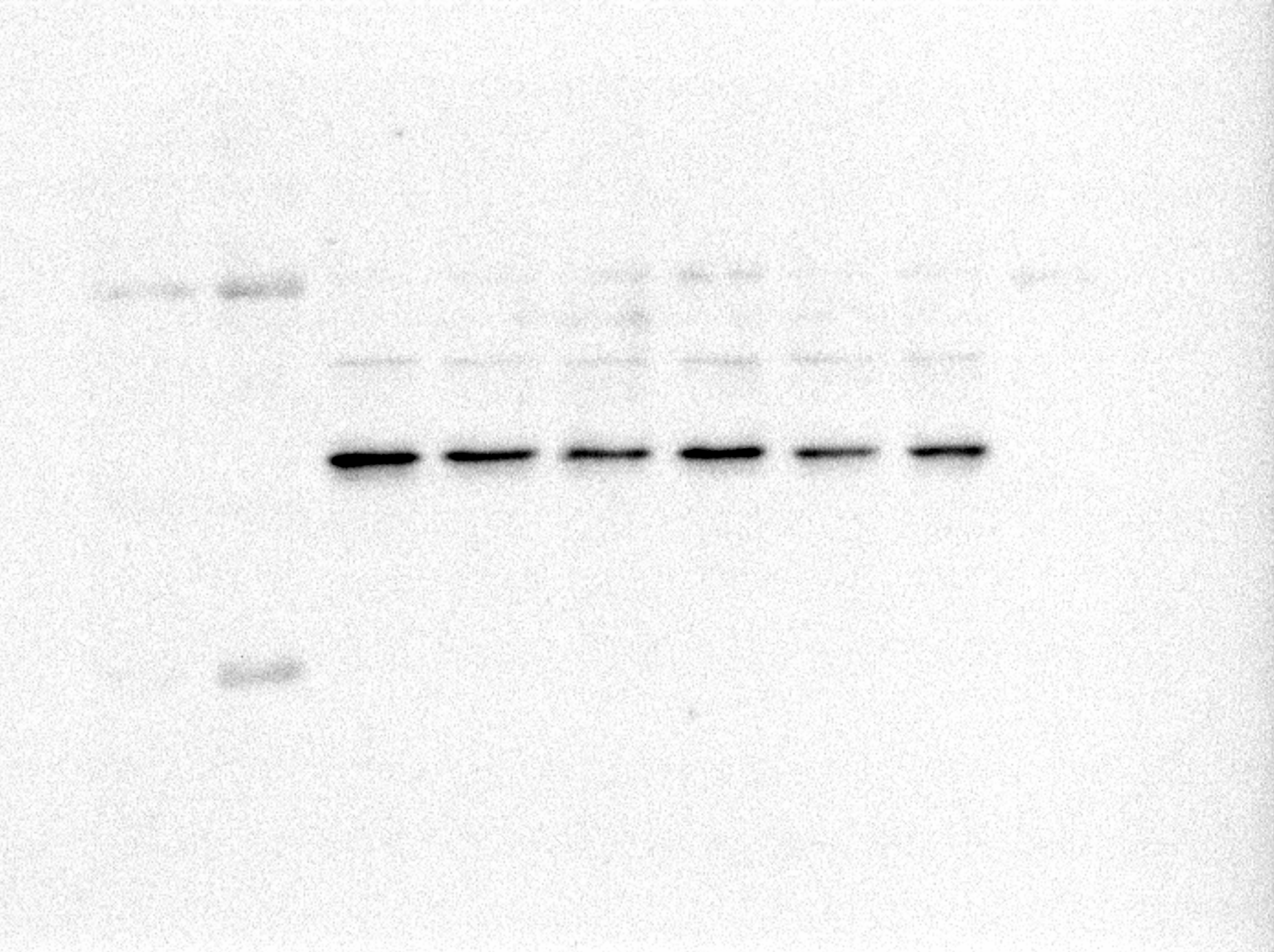

Supplement: Supplementary file 3 — Source data Fig. 1 [file 44318_2024_252_MOESM3_ESM.zip › Figure 1/1F/Figure1F_NT-3.tif]

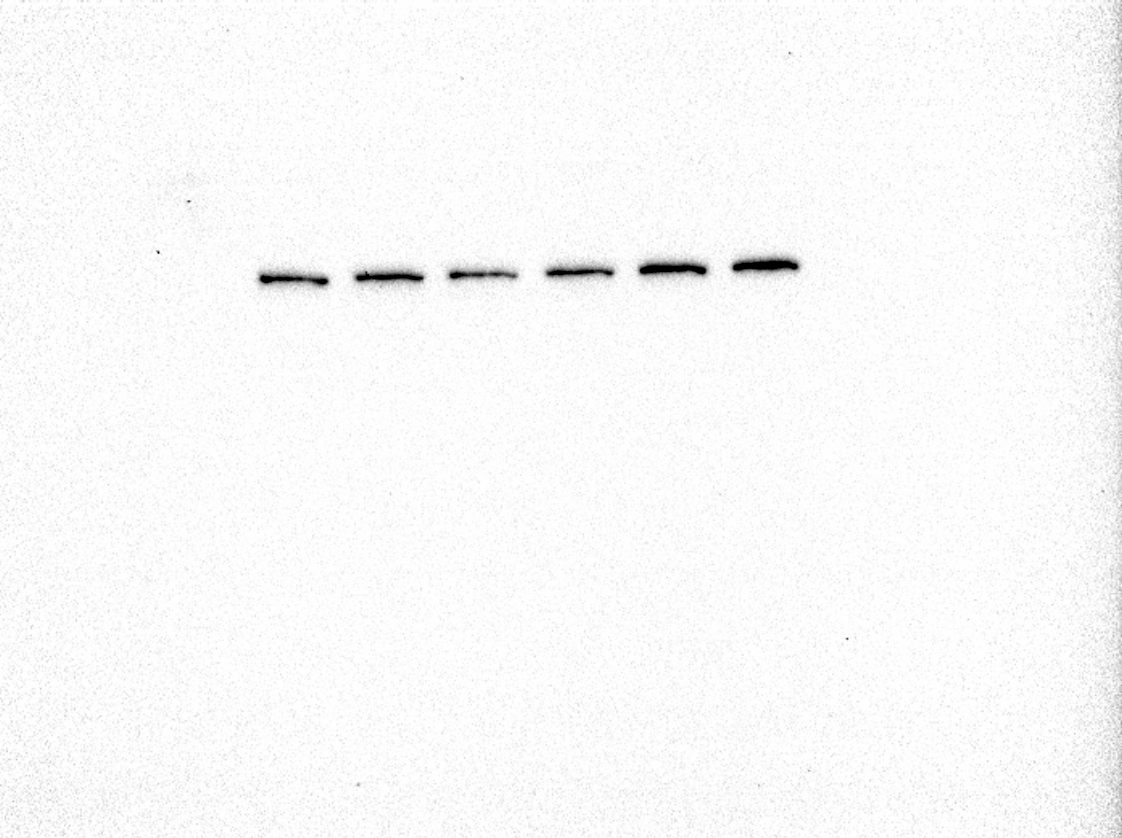

Supplement: Supplementary file 3 — Source data Fig. 1 [file 44318_2024_252_MOESM3_ESM.zip › Figure 1/1F/Figure1F_PTPsigma.tif]

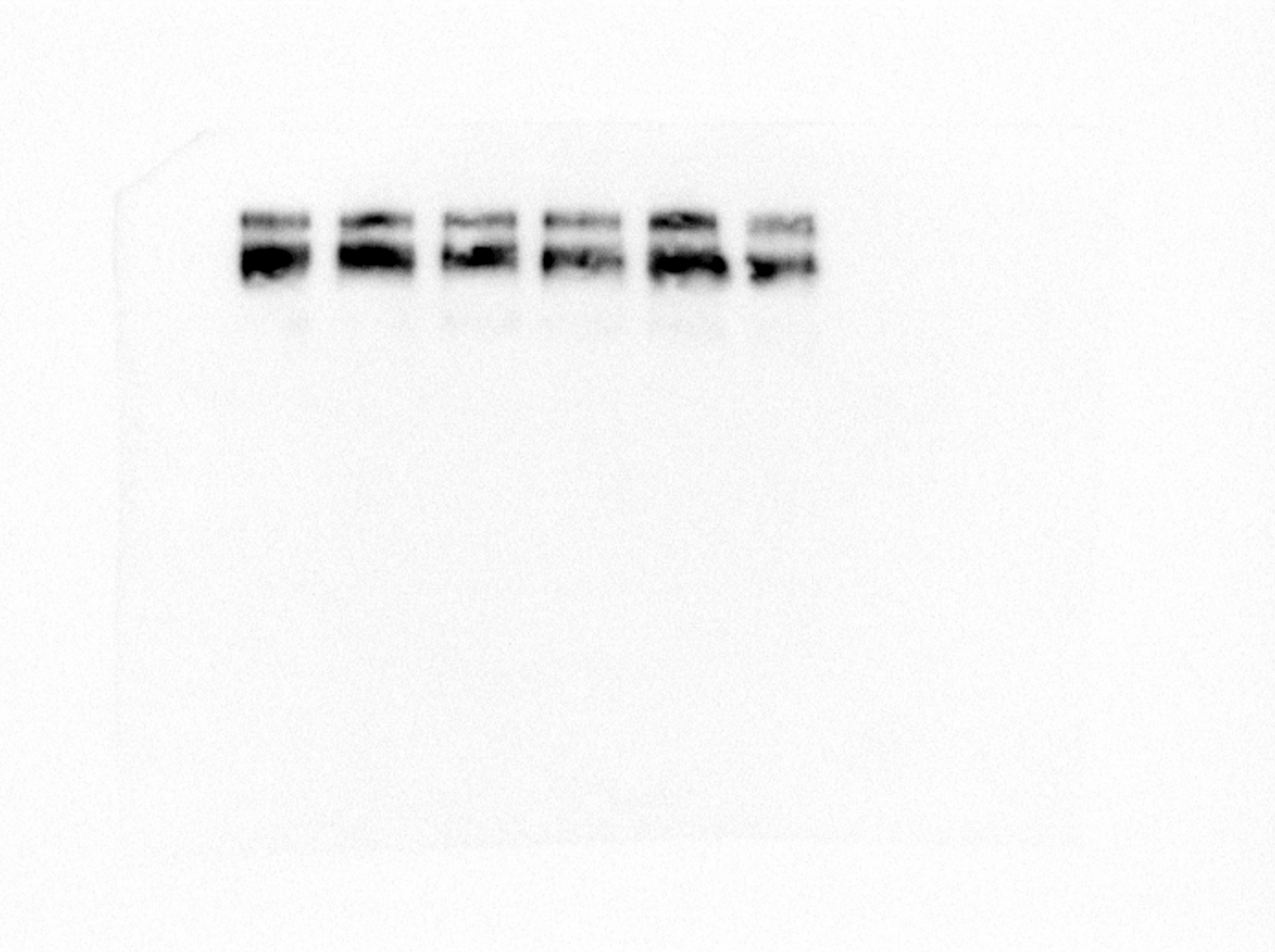

Supplement: Supplementary file 3 — Source data Fig. 1 [file 44318_2024_252_MOESM3_ESM.zip › Figure 1/1F/Figure1F_TrkC.tif]

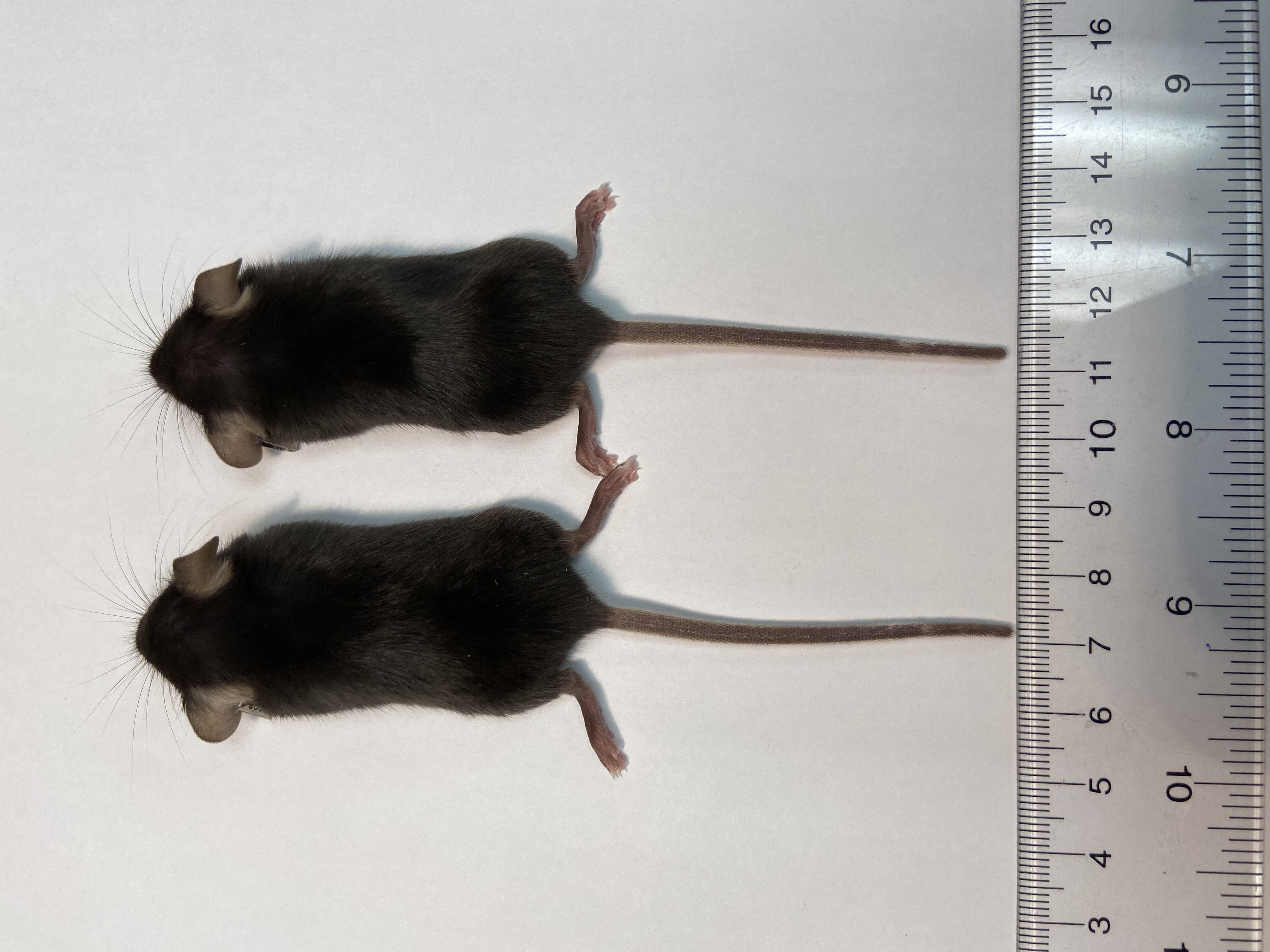

Supplement: Supplementary file 3 — Source data Fig. 1 [file 44318_2024_252_MOESM3_ESM.zip › Figure 1/1H/Figure 1H.jpg]

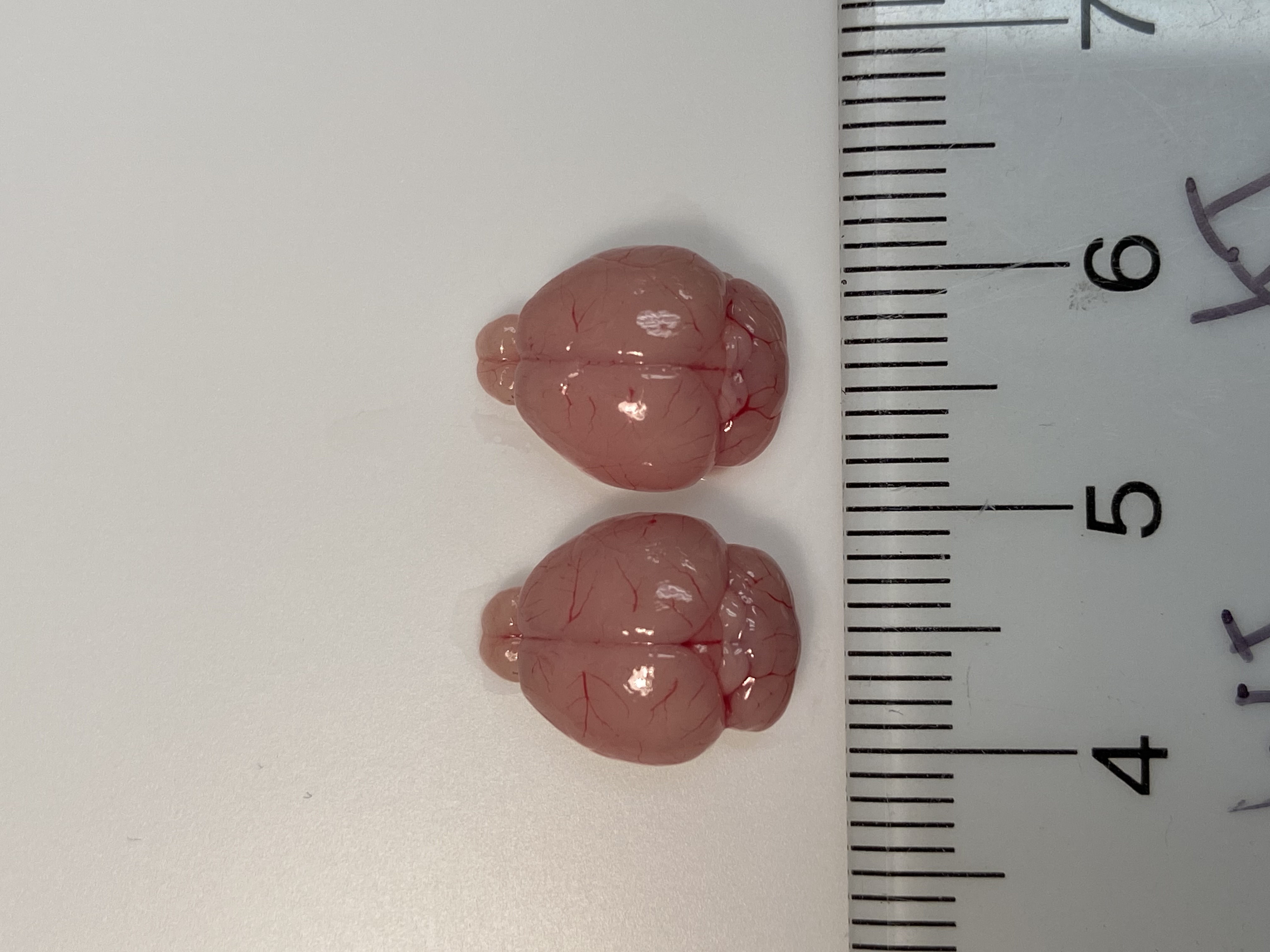

Supplement: Supplementary file 3 — Source data Fig. 1 [file 44318_2024_252_MOESM3_ESM.zip › Figure 1/1I/Figure 1I.jpg]

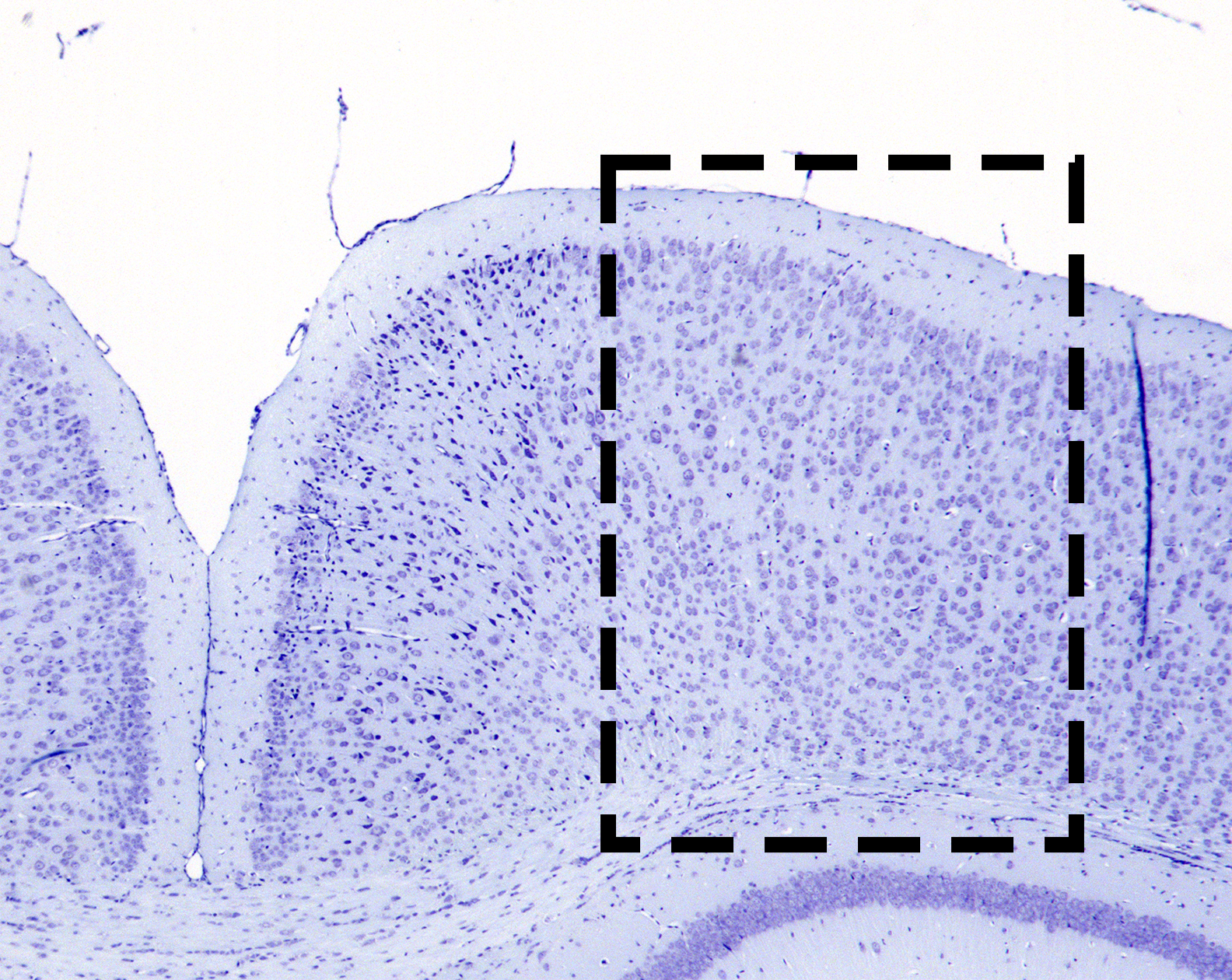

Supplement: Supplementary file 3 — Source data Fig. 1 [file 44318_2024_252_MOESM3_ESM.zip › Figure 1/1J/KI cortex annotated.png]

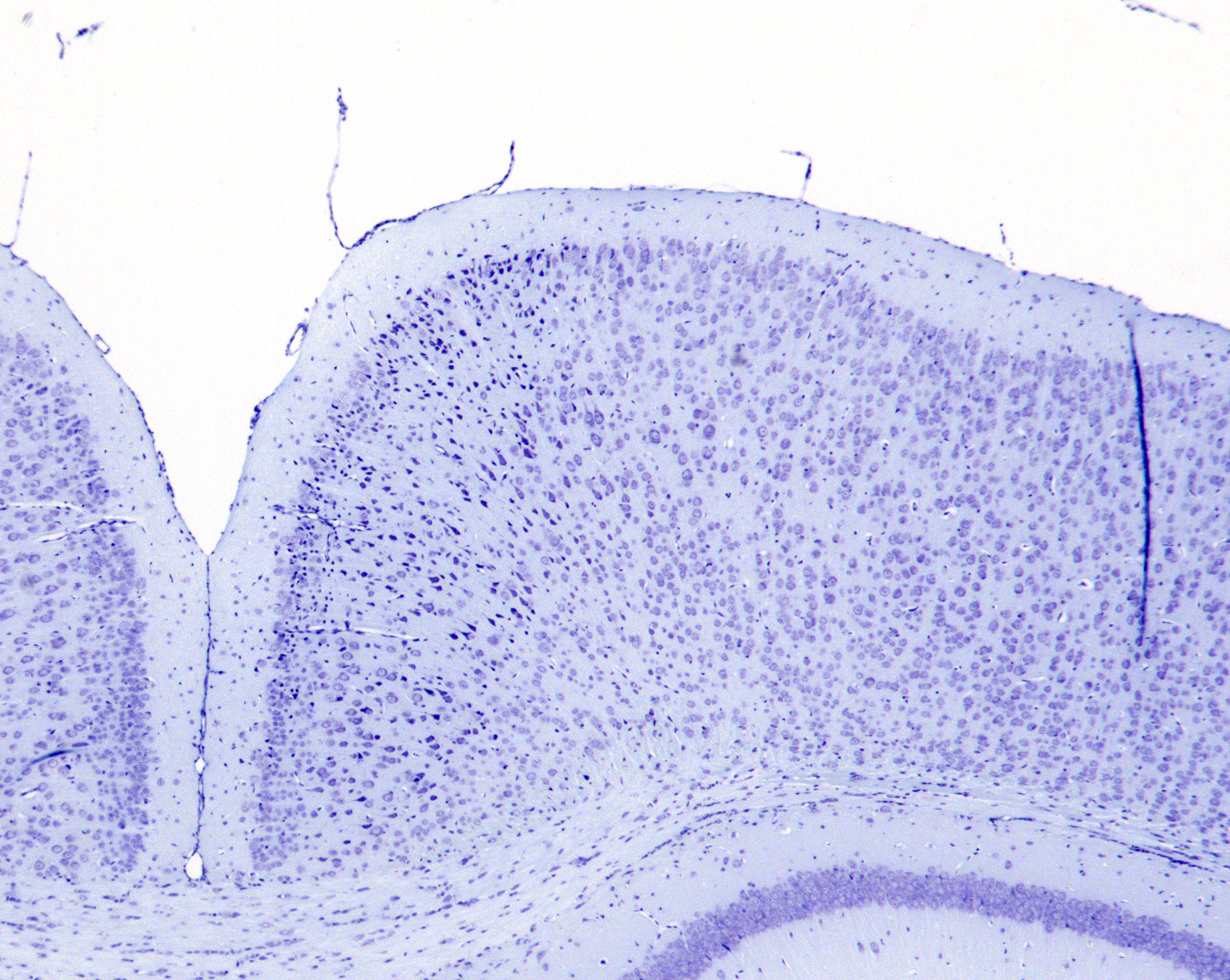

Supplement: Supplementary file 3 — Source data Fig. 1 [file 44318_2024_252_MOESM3_ESM.zip › Figure 1/1J/KI cortex.tif]

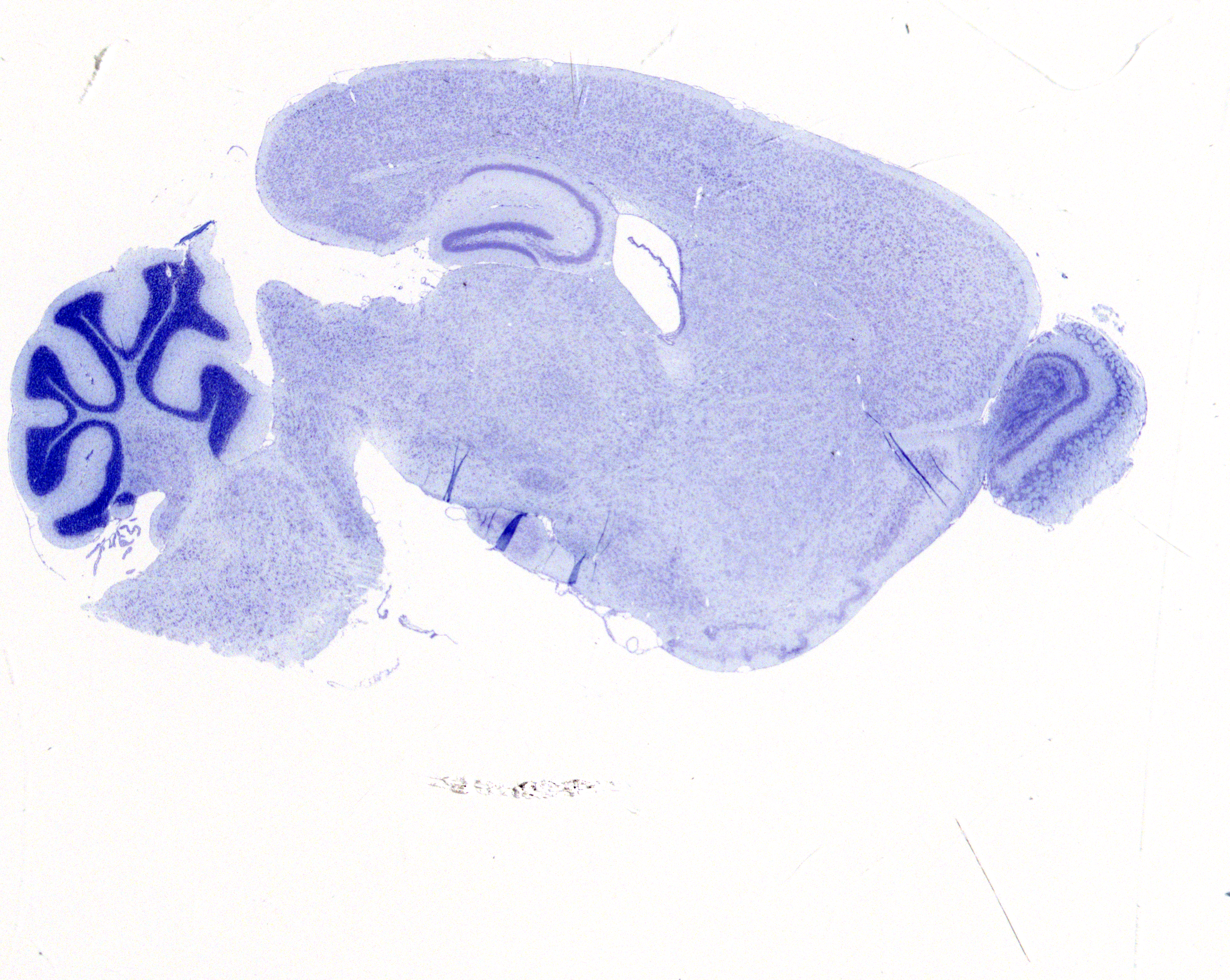

Supplement: Supplementary file 3 — Source data Fig. 1 [file 44318_2024_252_MOESM3_ESM.zip › Figure 1/1J/KI full brain.tif]

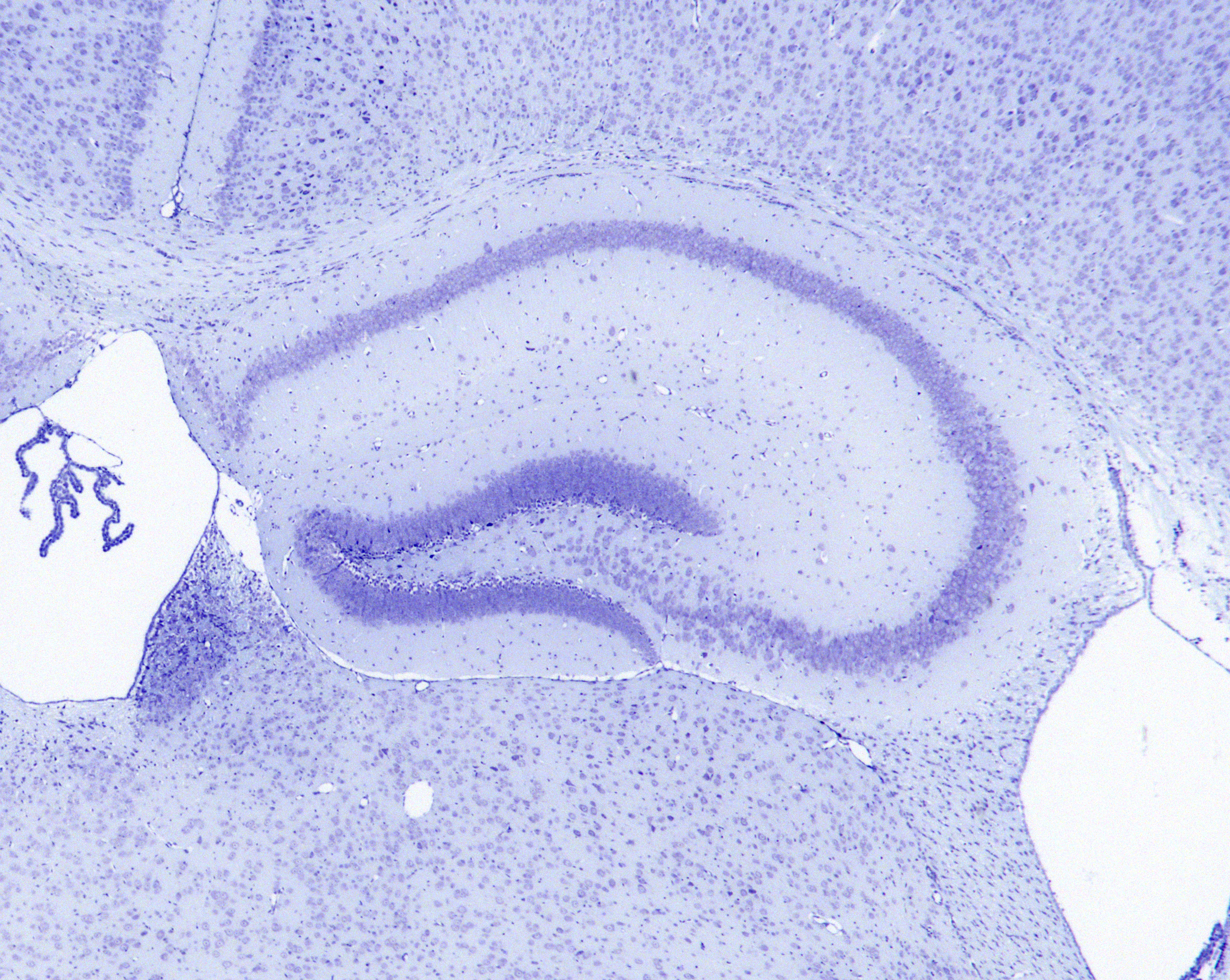

Supplement: Supplementary file 3 — Source data Fig. 1 [file 44318_2024_252_MOESM3_ESM.zip › Figure 1/1J/KI hippocampus.tif]

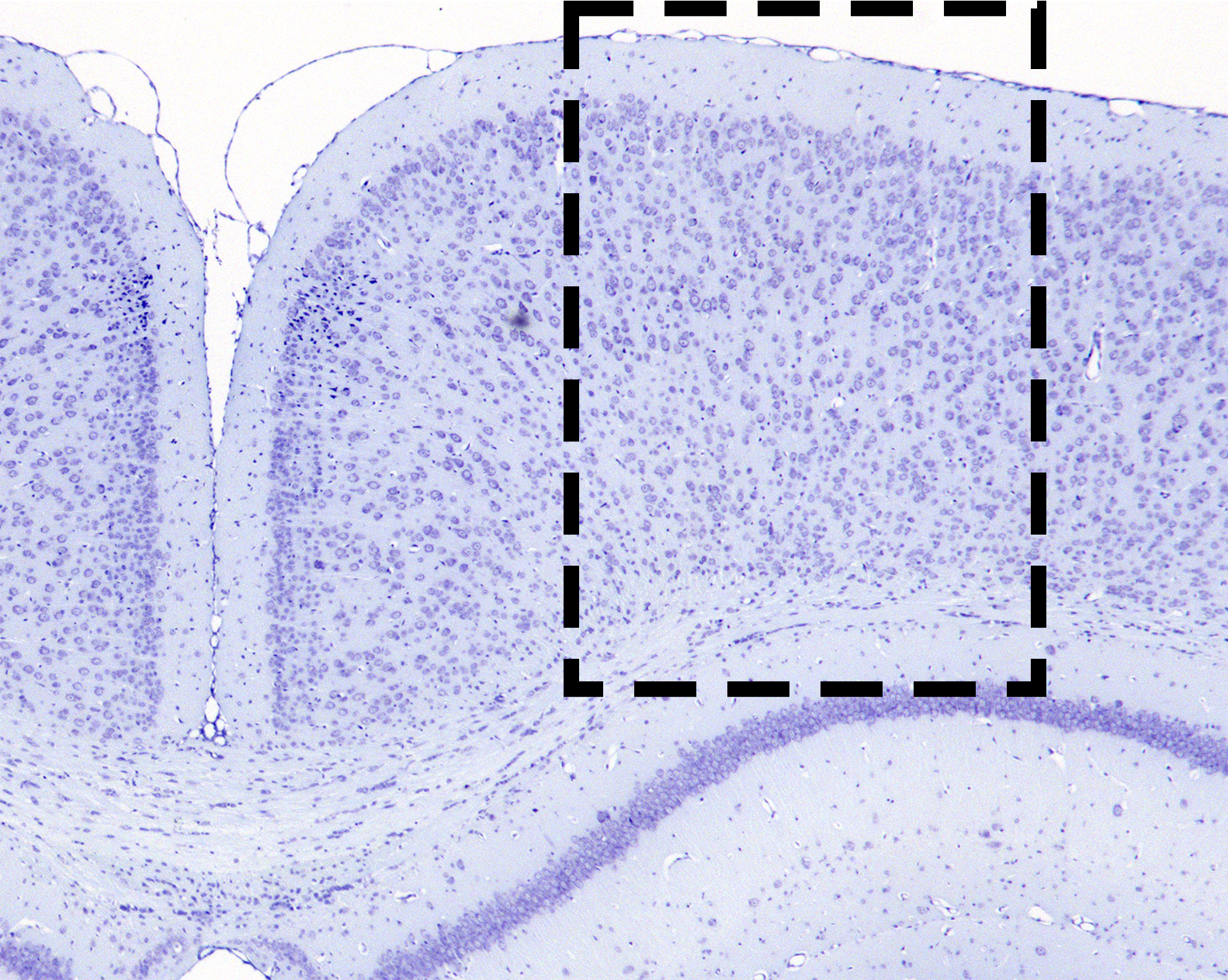

Supplement: Supplementary file 3 — Source data Fig. 1 [file 44318_2024_252_MOESM3_ESM.zip › Figure 1/1J/WT cortex annotated.png]

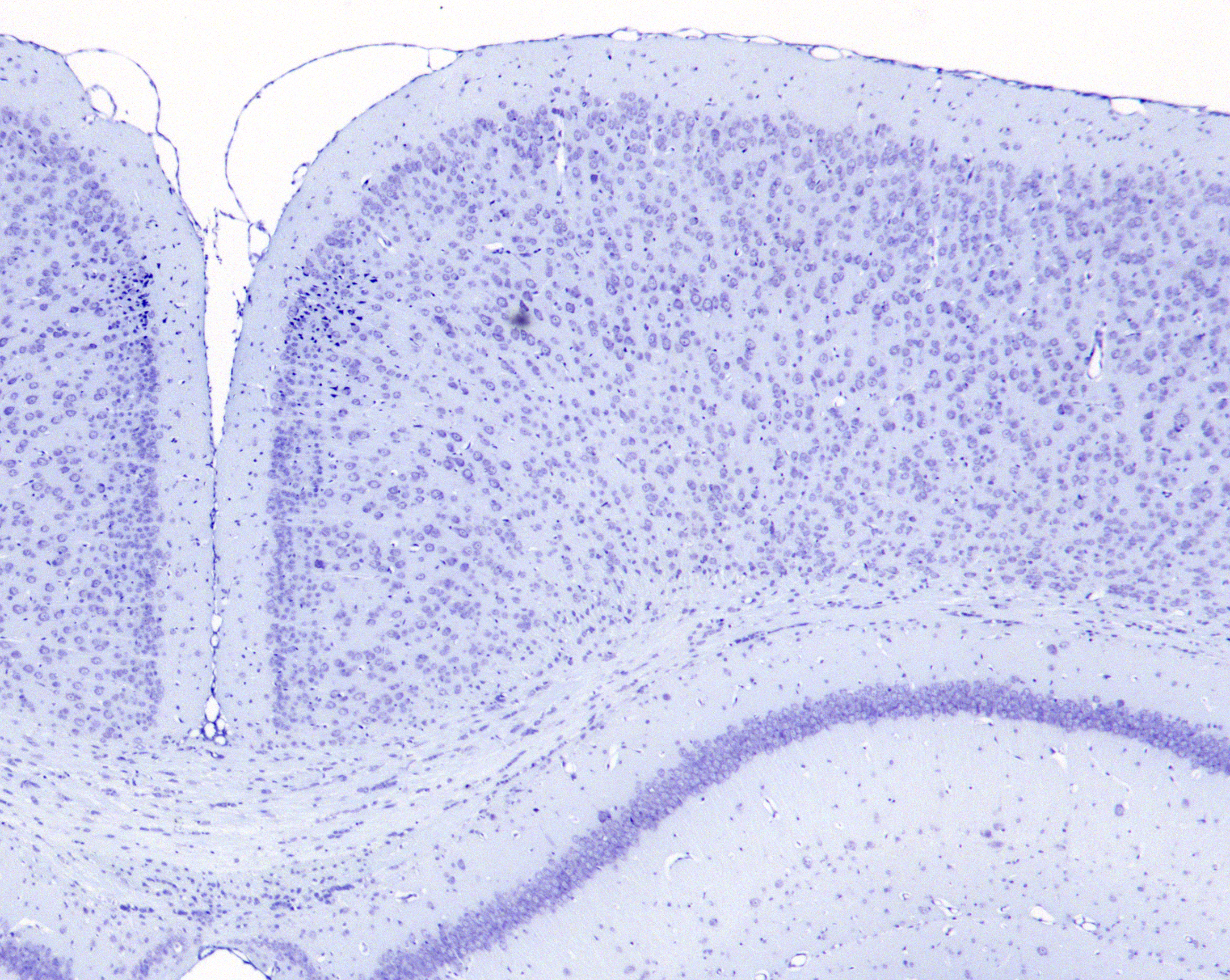

Supplement: Supplementary file 3 — Source data Fig. 1 [file 44318_2024_252_MOESM3_ESM.zip › Figure 1/1J/WT cortex.tif]

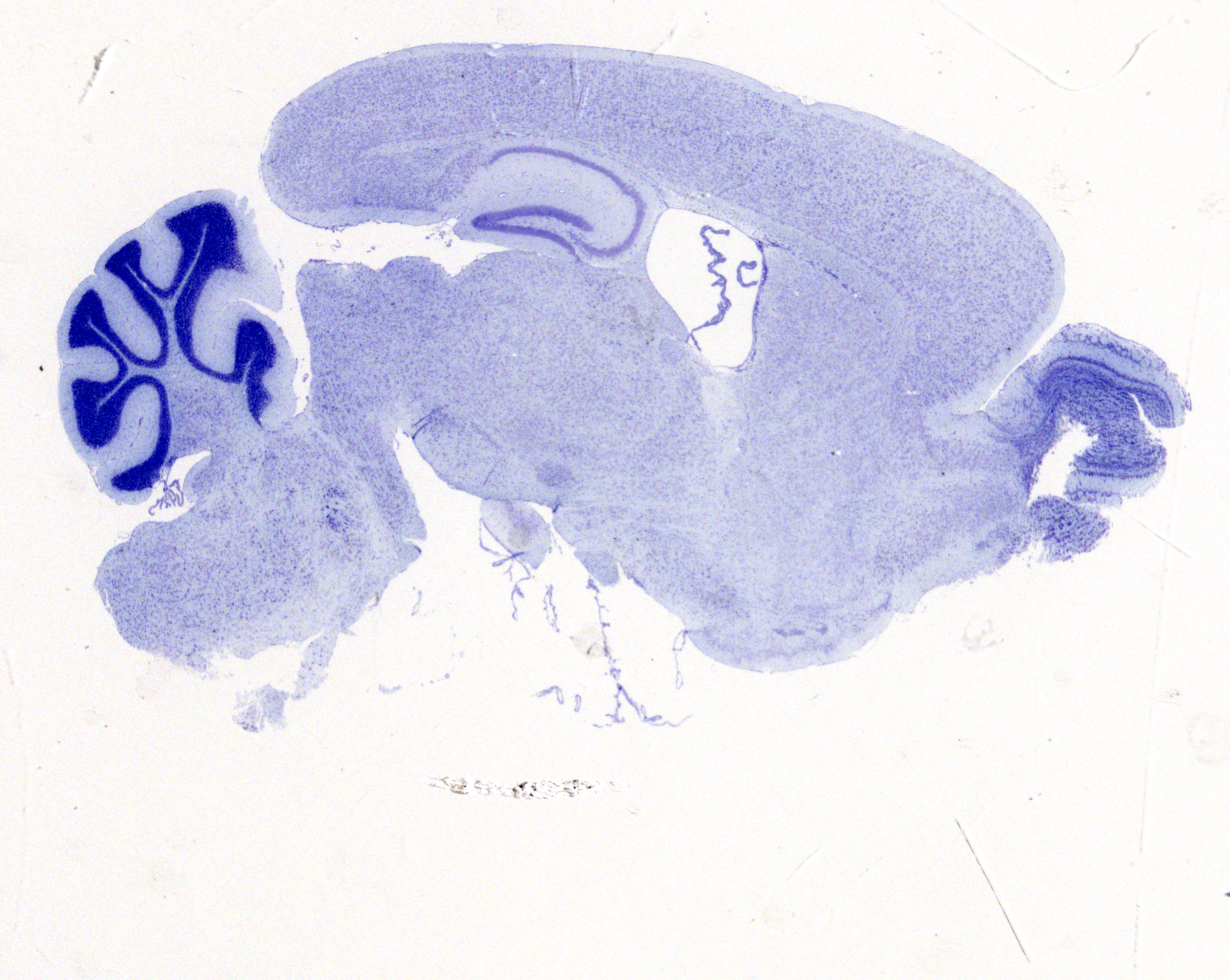

Supplement: Supplementary file 3 — Source data Fig. 1 [file 44318_2024_252_MOESM3_ESM.zip › Figure 1/1J/WT full brain.tif]

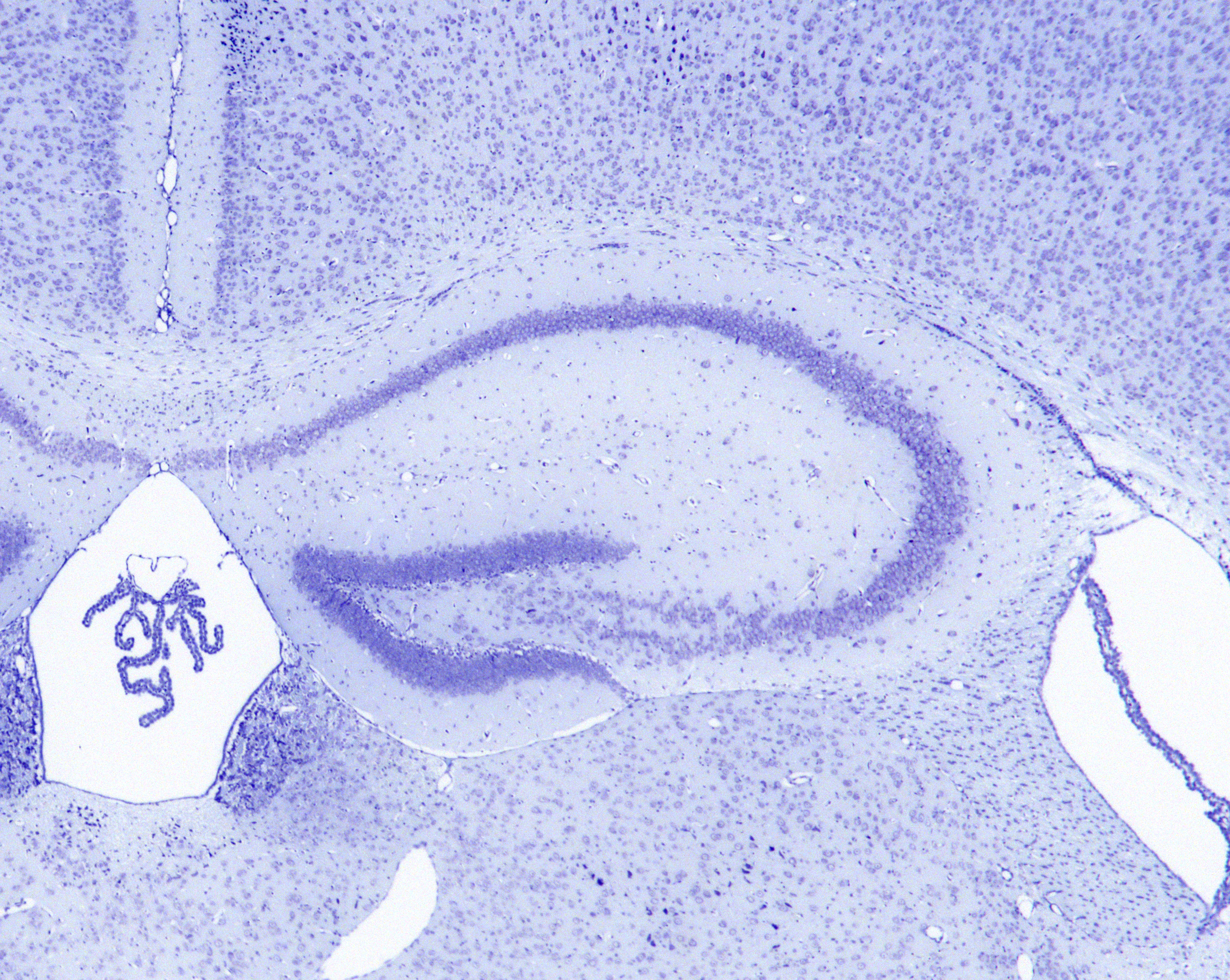

Supplement: Supplementary file 3 — Source data Fig. 1 [file 44318_2024_252_MOESM3_ESM.zip › Figure 1/1J/WT hippocampus.tif]

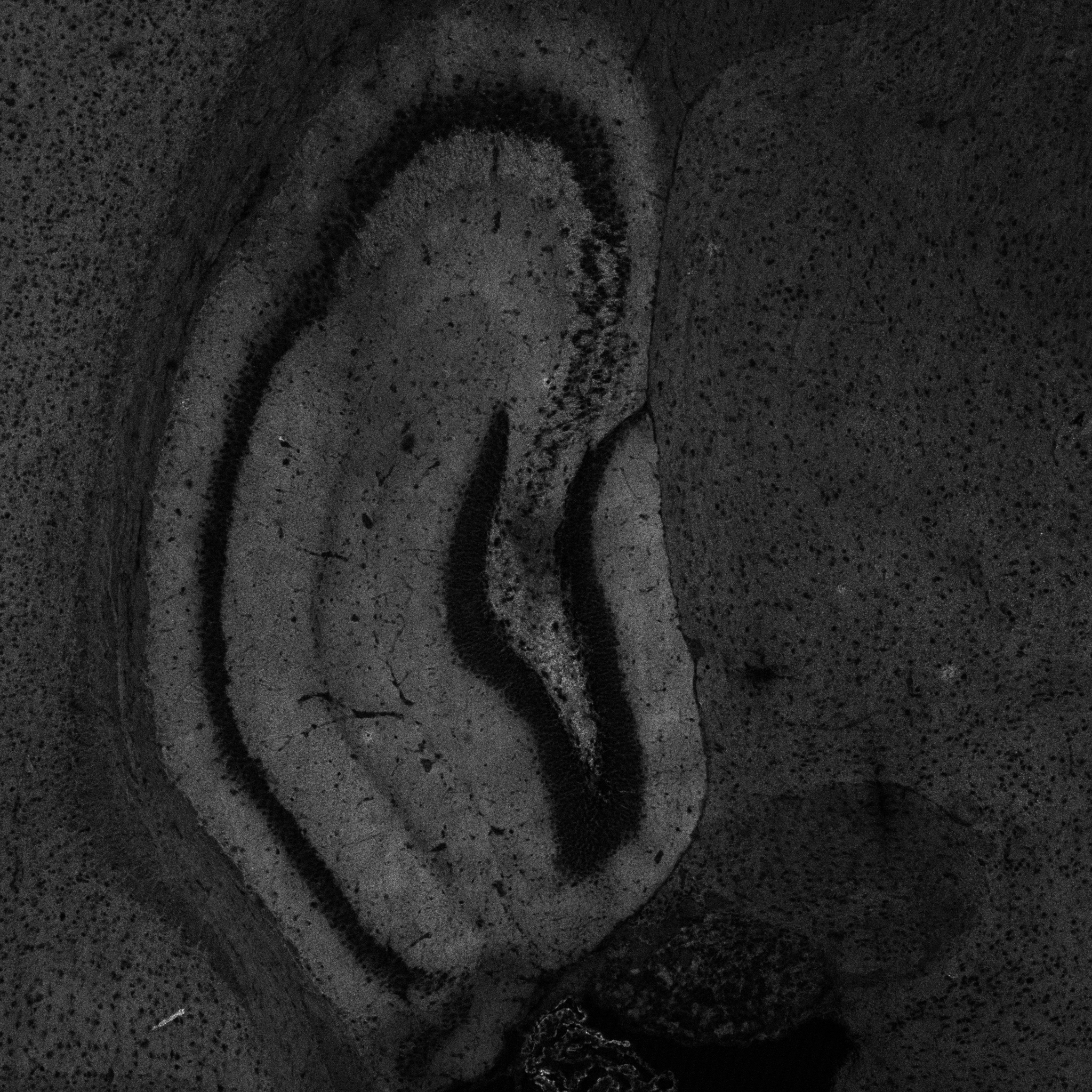

Supplement: Supplementary file 4 — Source data Fig. 2 [file 44318_2024_252_MOESM4_ESM.zip › Figure 2/2A/Fig2A_KI_PSD95.tif]

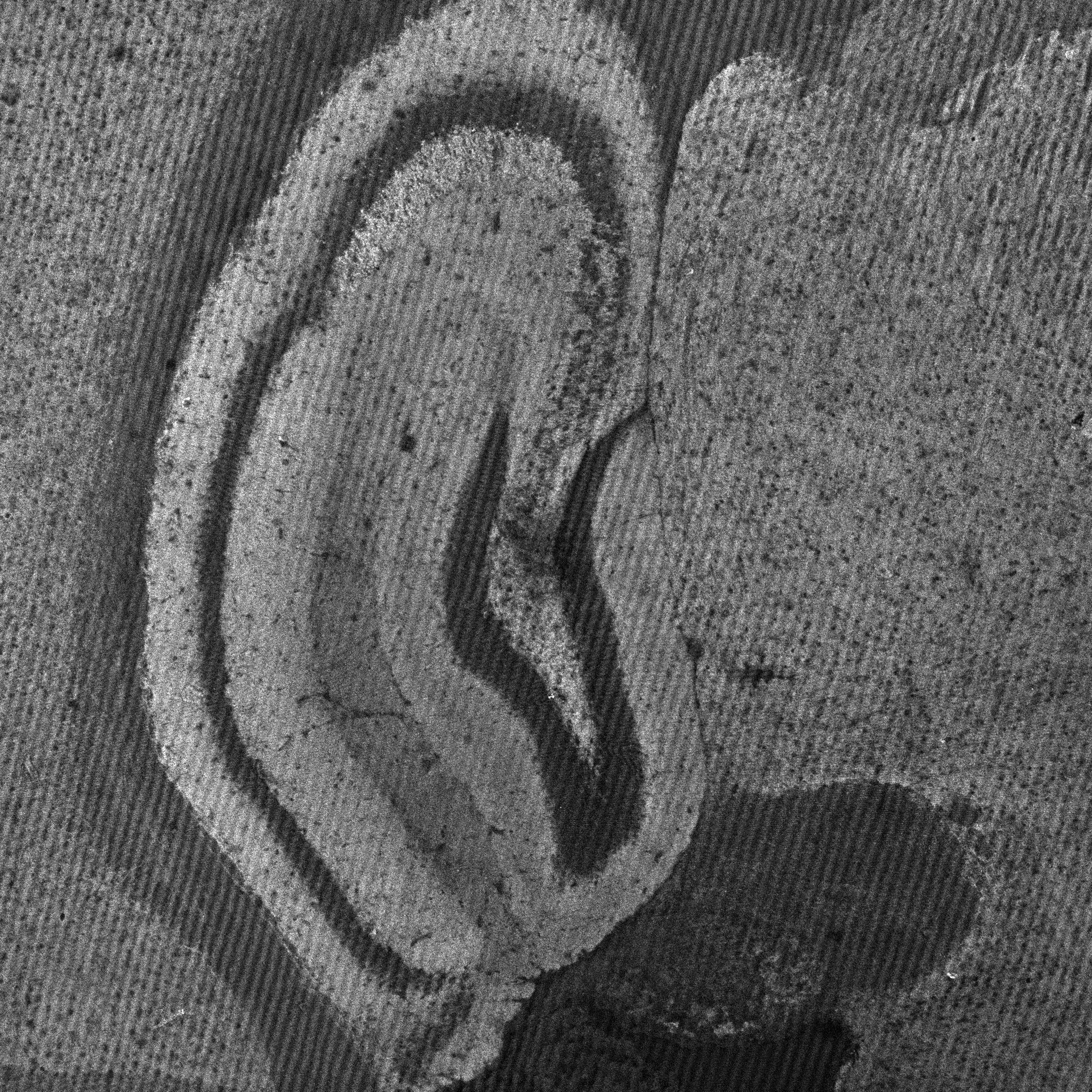

Supplement: Supplementary file 4 — Source data Fig. 2 [file 44318_2024_252_MOESM4_ESM.zip › Figure 2/2A/Fig2A_KI_VGLUT1.tif]

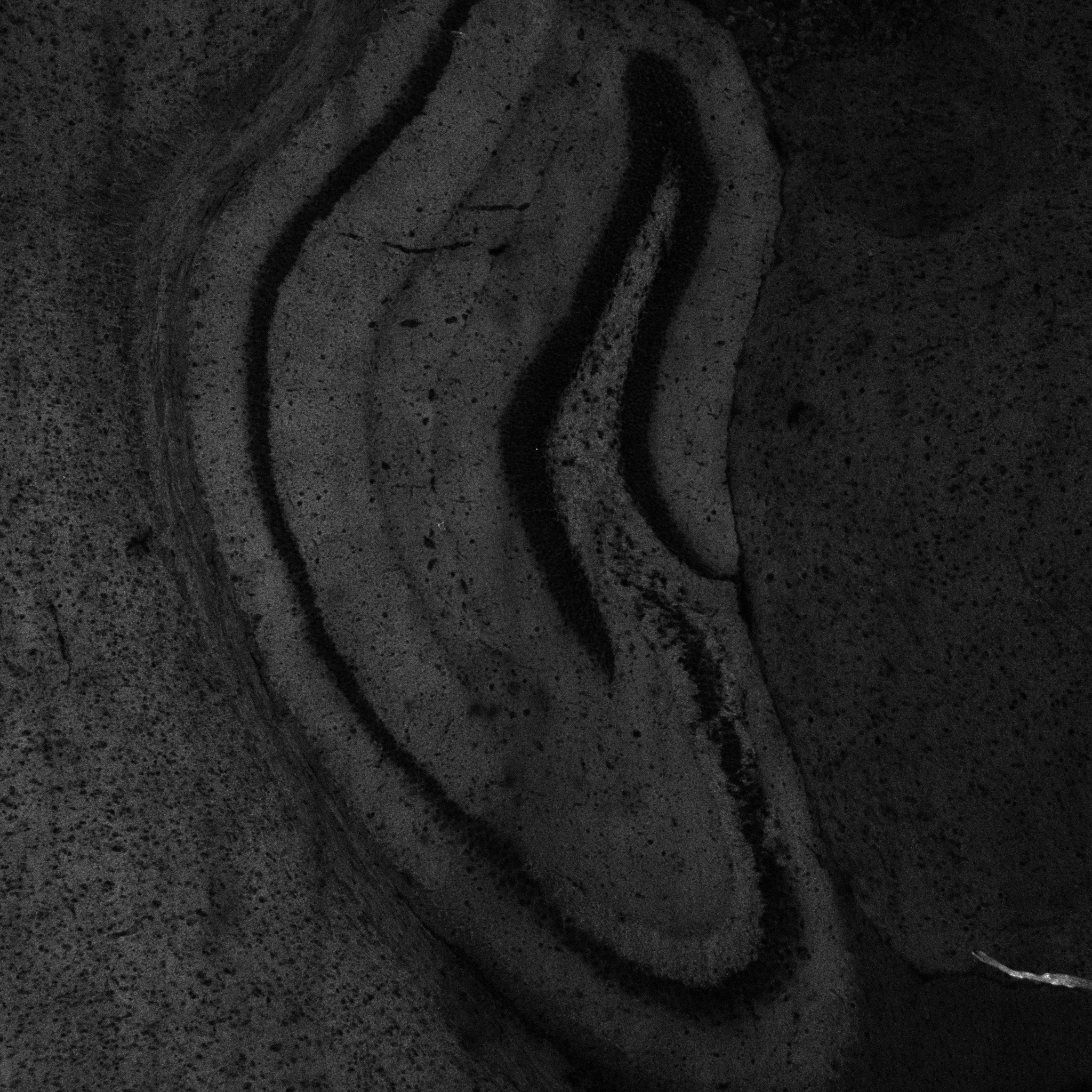

Supplement: Supplementary file 4 — Source data Fig. 2 [file 44318_2024_252_MOESM4_ESM.zip › Figure 2/2A/Fig2A_WT_PSD95.tif]

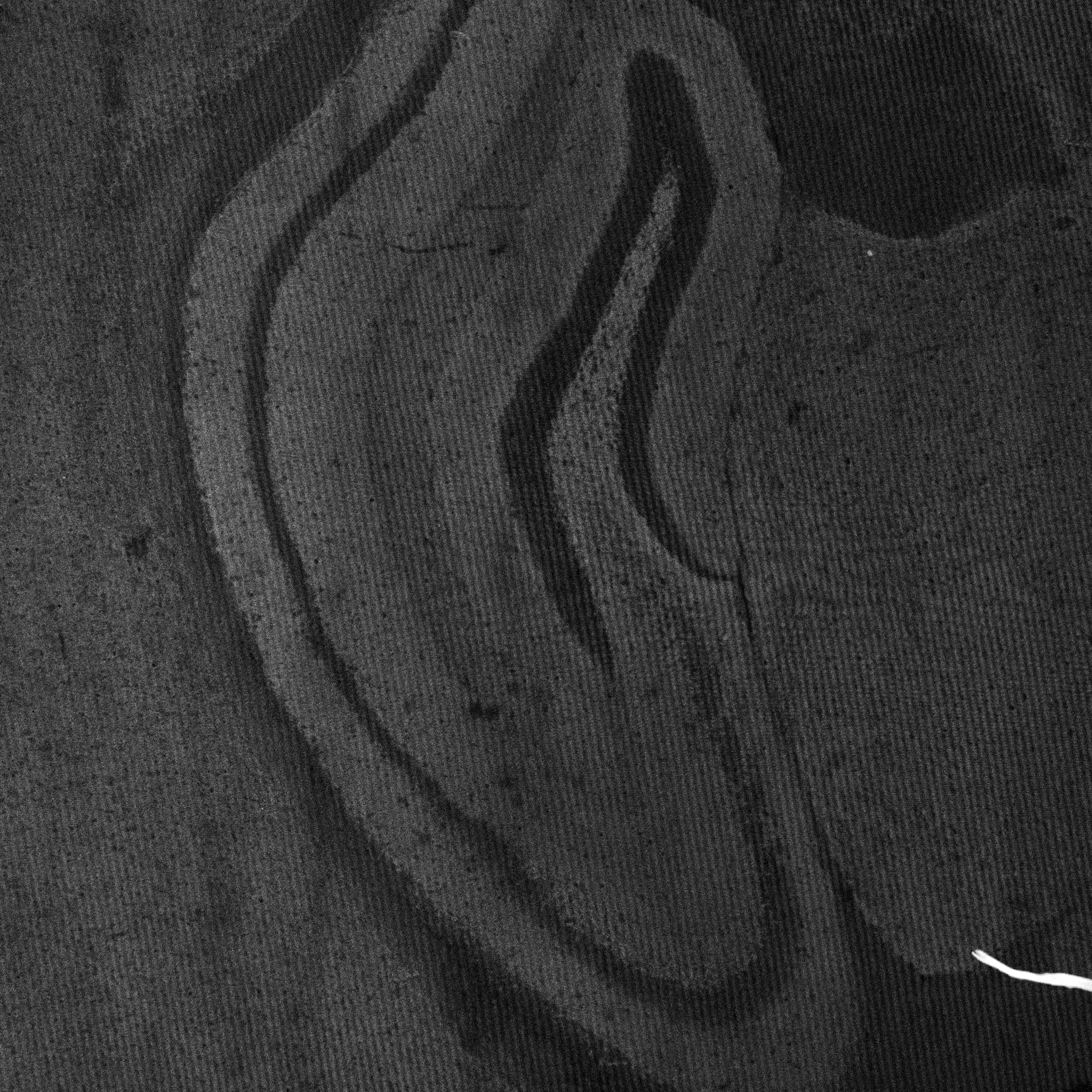

Supplement: Supplementary file 4 — Source data Fig. 2 [file 44318_2024_252_MOESM4_ESM.zip › Figure 2/2A/Fig2A_WT_VGLUT1.tif]

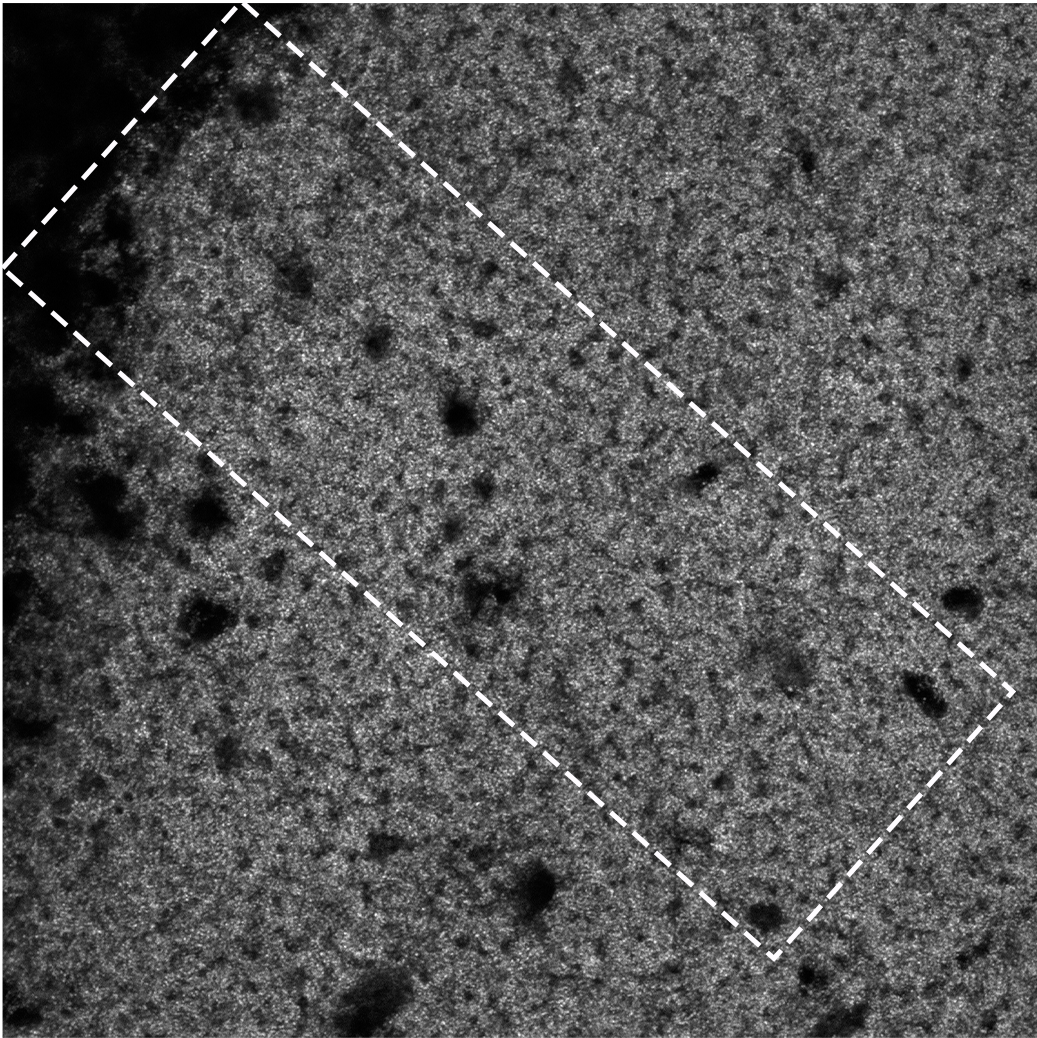

Supplement: Supplementary file 4 — Source data Fig. 2 [file 44318_2024_252_MOESM4_ESM.zip › Figure 2/2B/Figure2B_S.R_KI_PSD95 annotated.png]

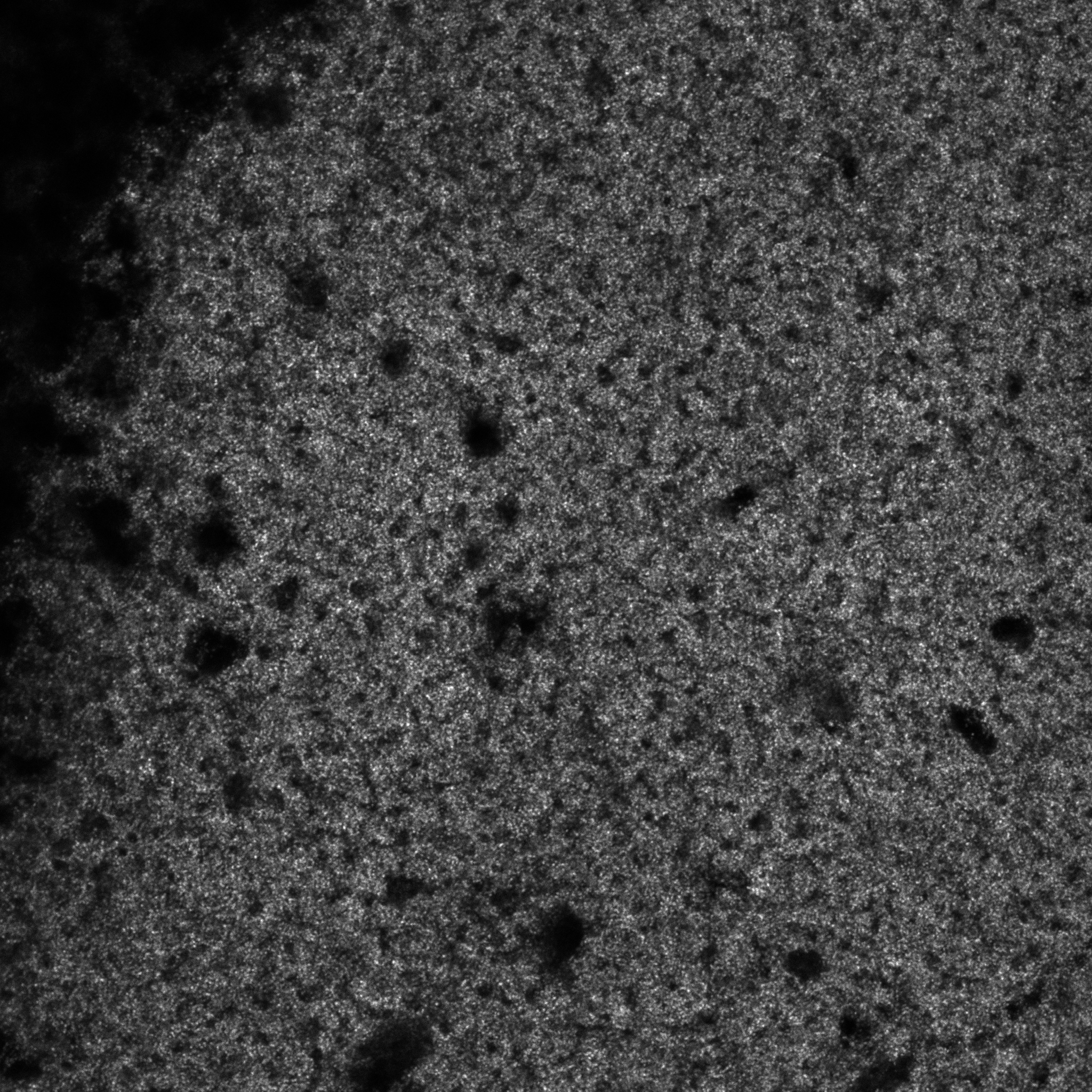

Supplement: Supplementary file 4 — Source data Fig. 2 [file 44318_2024_252_MOESM4_ESM.zip › Figure 2/2B/Figure2B_S.R_KI_PSD95.tif]

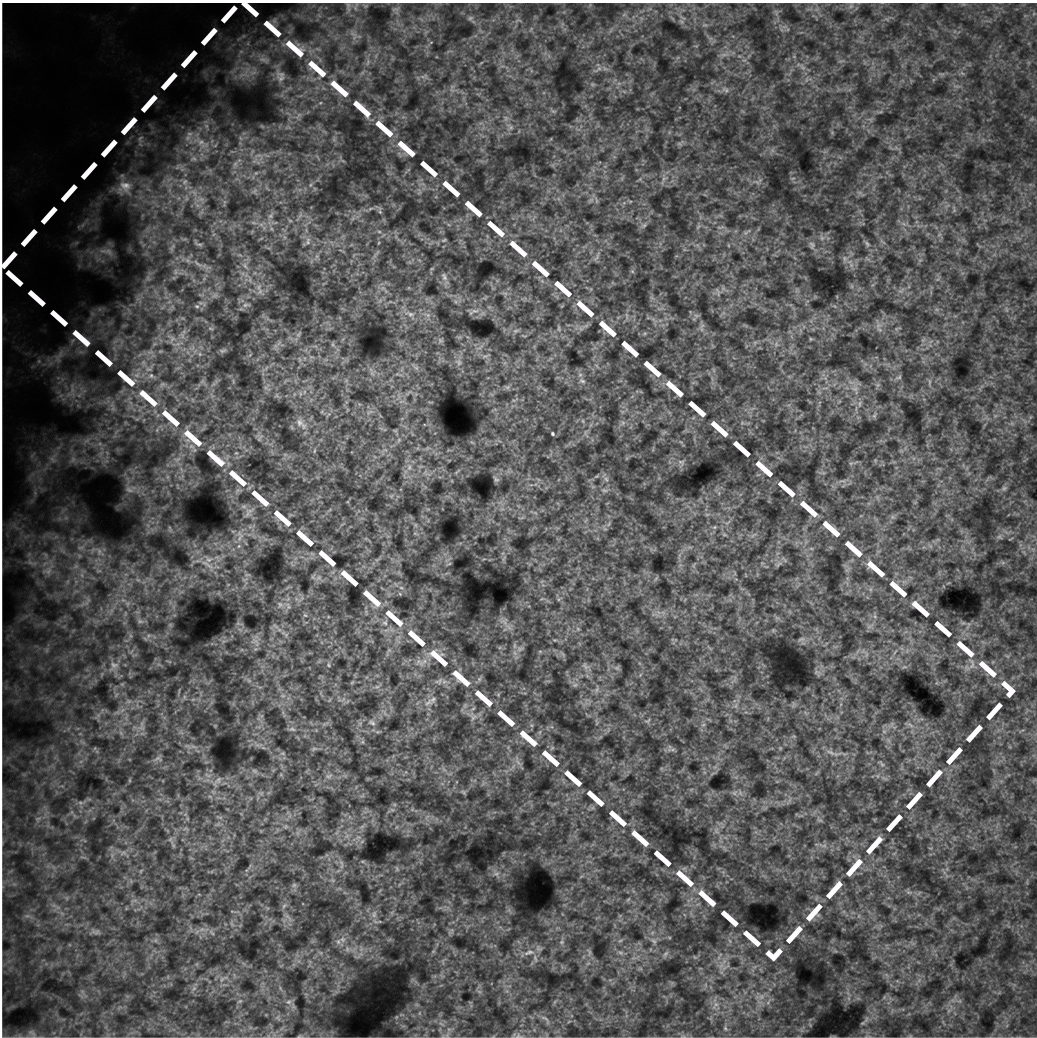

Supplement: Supplementary file 4 — Source data Fig. 2 [file 44318_2024_252_MOESM4_ESM.zip › Figure 2/2B/Figure2B_S.R_KI_VGLUT1 annotated.png]

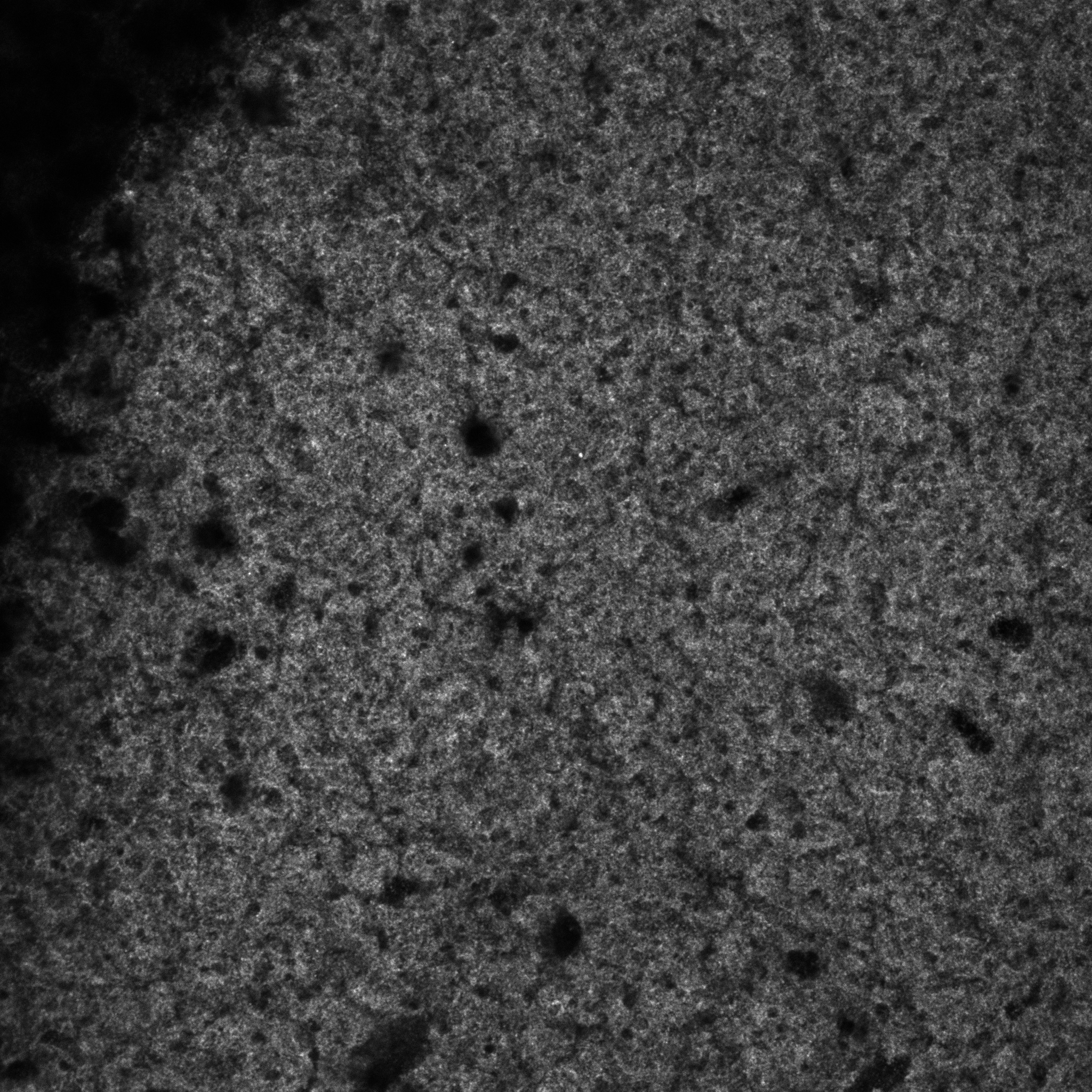

Supplement: Supplementary file 4 — Source data Fig. 2 [file 44318_2024_252_MOESM4_ESM.zip › Figure 2/2B/Figure2B_S.R_KI_VGLUT1.tif]

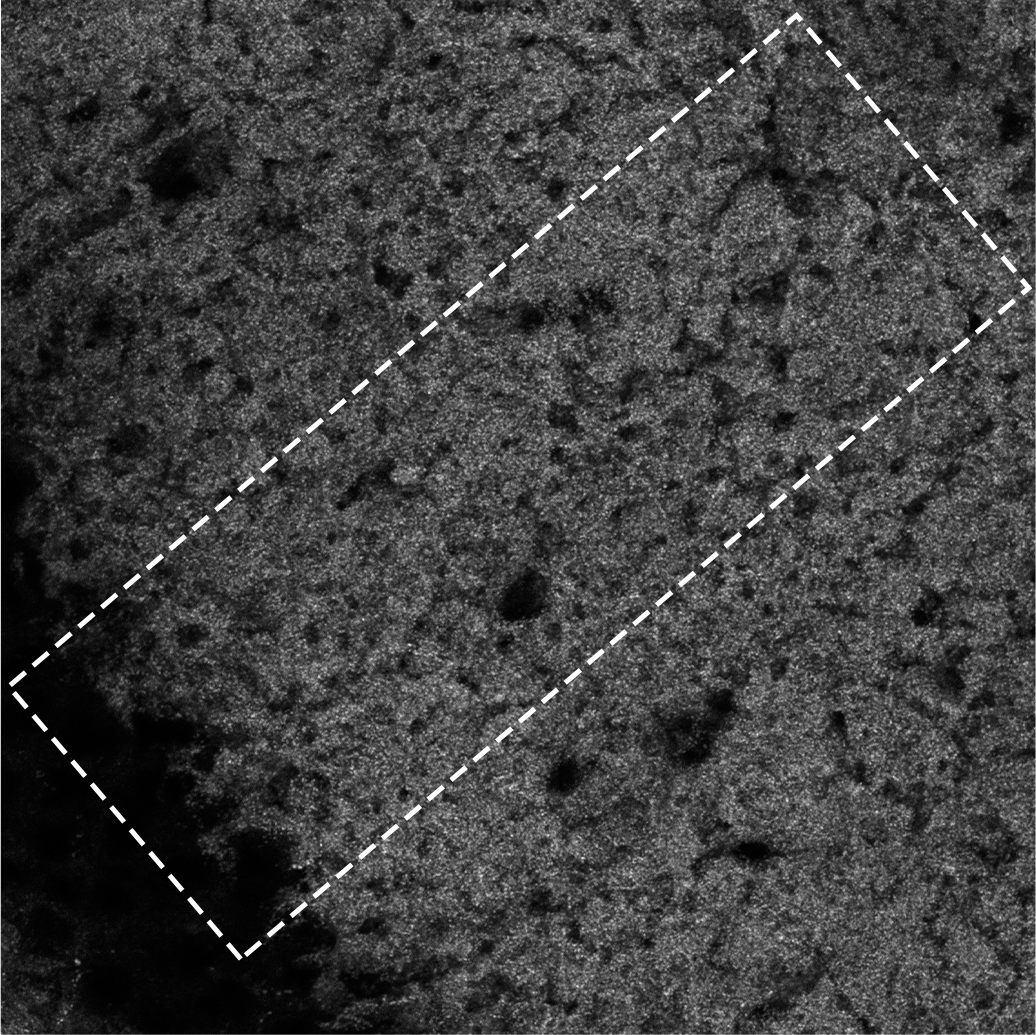

Supplement: Supplementary file 4 — Source data Fig. 2 [file 44318_2024_252_MOESM4_ESM.zip › Figure 2/2B/Figure2B_S.R_WT_PSD95 annotated.png]

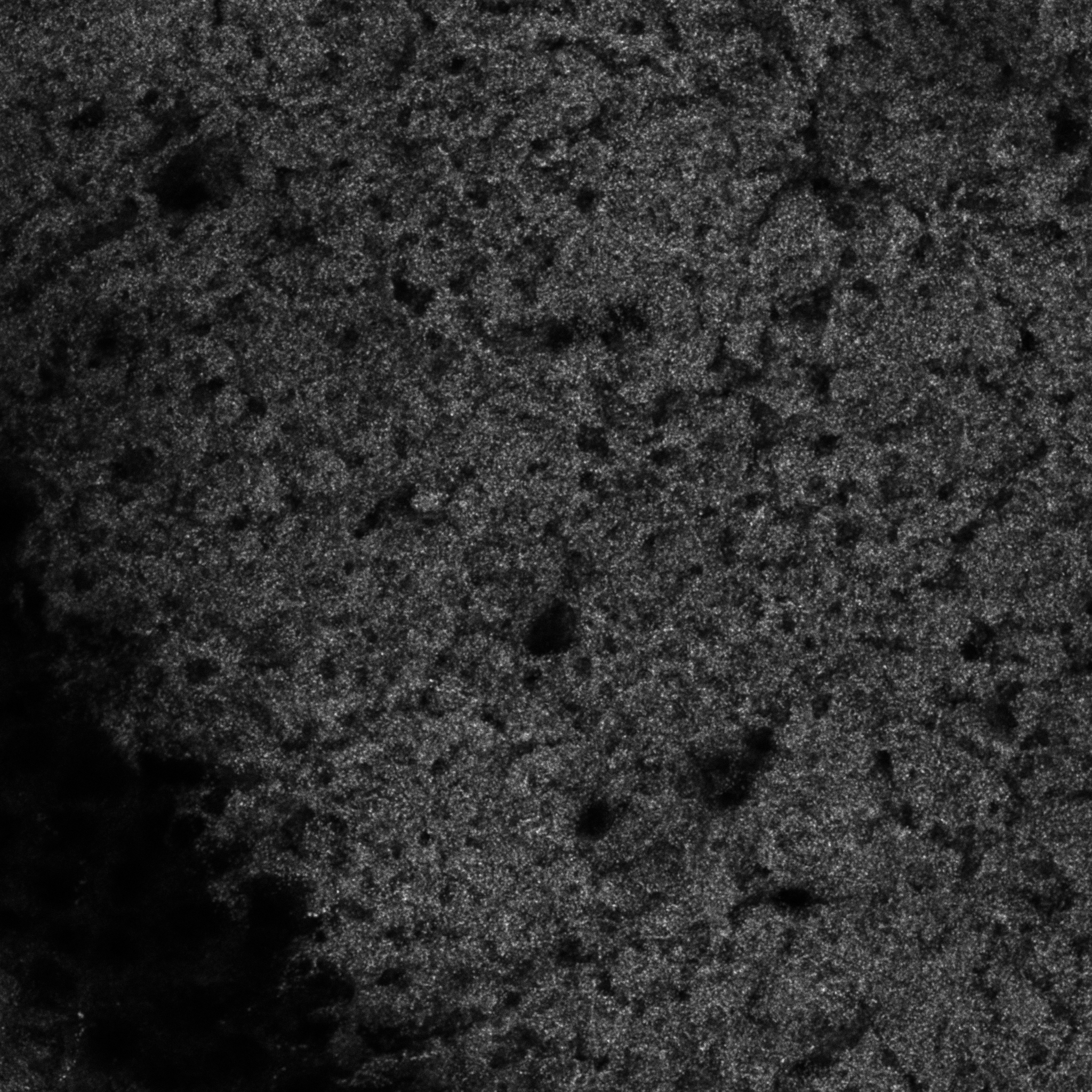

Supplement: Supplementary file 4 — Source data Fig. 2 [file 44318_2024_252_MOESM4_ESM.zip › Figure 2/2B/Figure2B_S.R_WT_PSD95.tif]

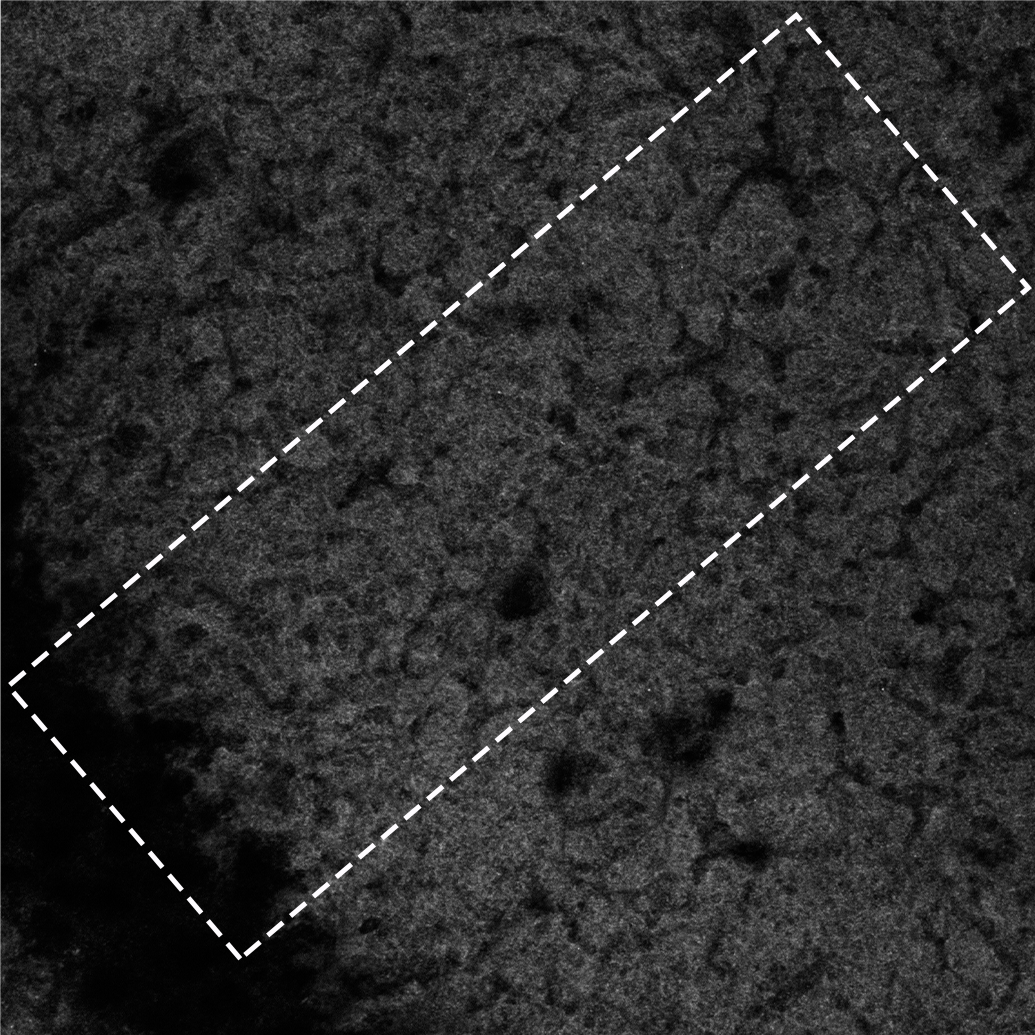

Supplement: Supplementary file 4 — Source data Fig. 2 [file 44318_2024_252_MOESM4_ESM.zip › Figure 2/2B/Figure2B_S.R_WT_VGLUT1 annotated.png]

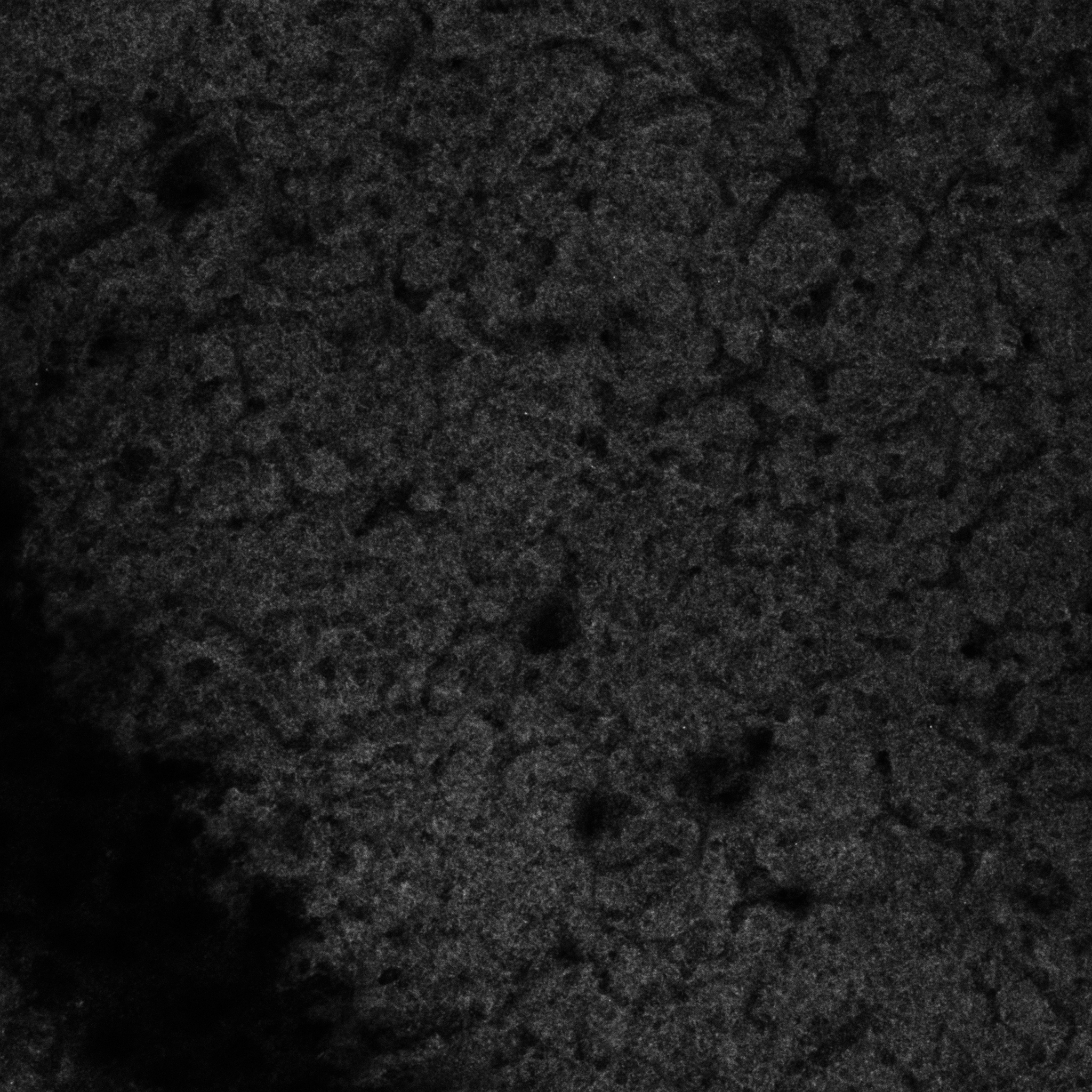

Supplement: Supplementary file 4 — Source data Fig. 2 [file 44318_2024_252_MOESM4_ESM.zip › Figure 2/2B/Figure2B_S.R_WT_VGLUT1.tif]

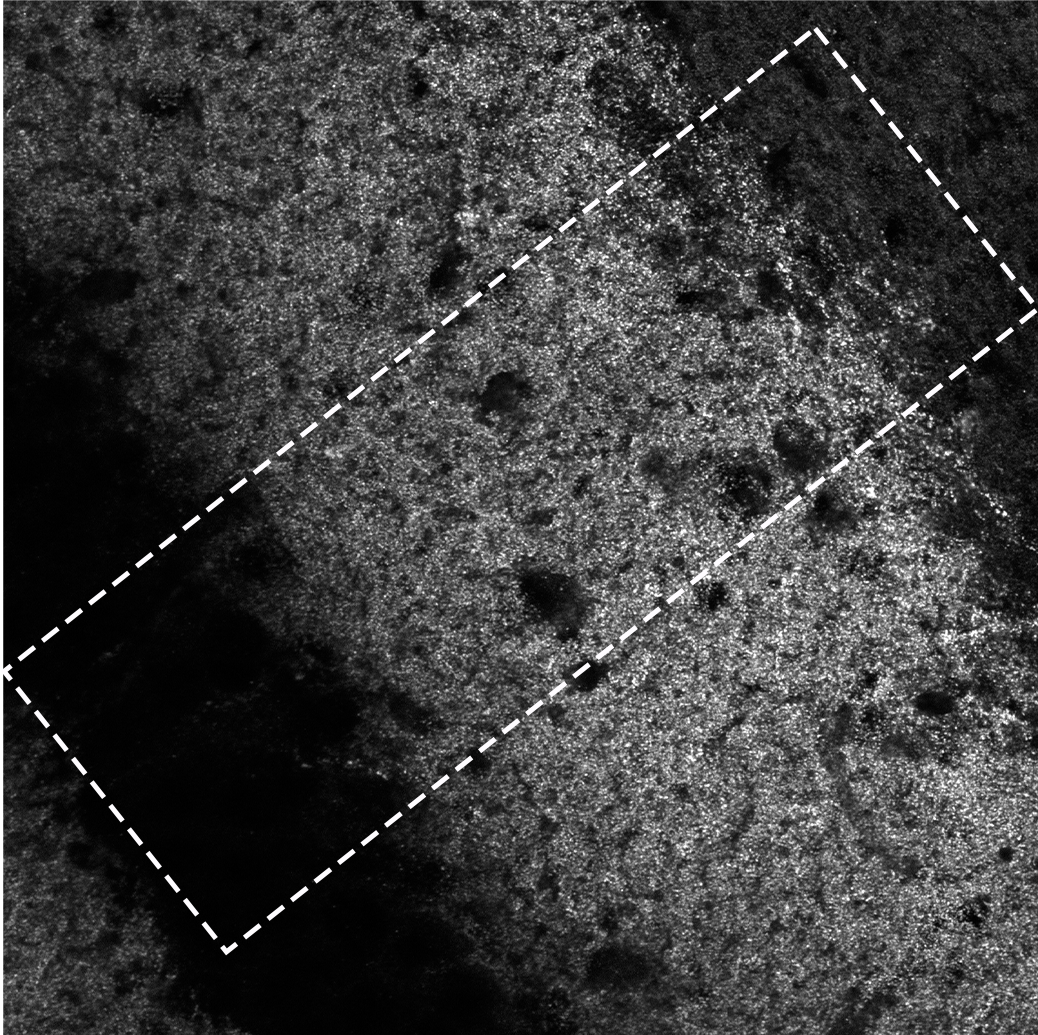

Supplement: Supplementary file 4 — Source data Fig. 2 [file 44318_2024_252_MOESM4_ESM.zip › Figure 2/2C/Figure2C_S.O_KI_PSD95 annotated.png]

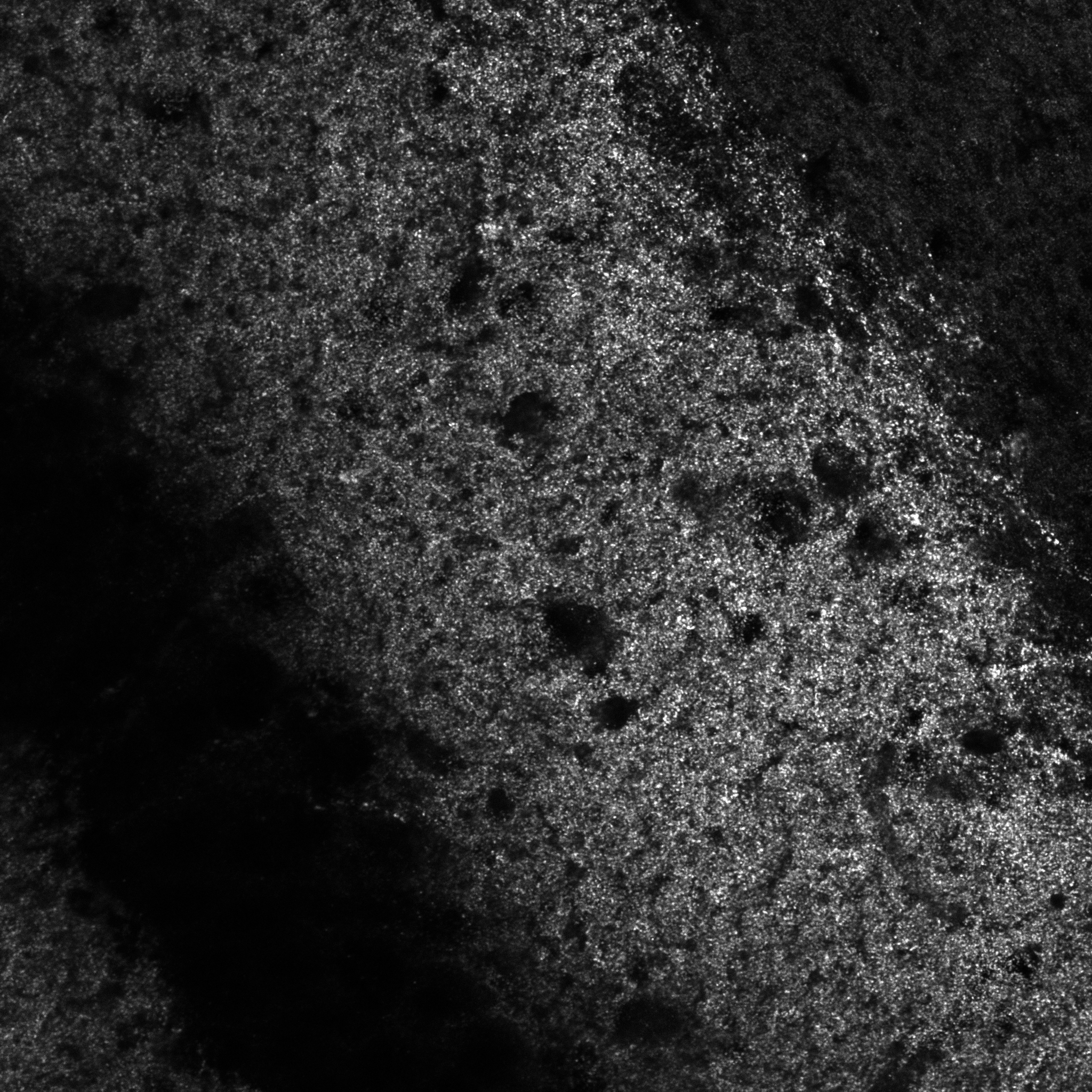

Supplement: Supplementary file 4 — Source data Fig. 2 [file 44318_2024_252_MOESM4_ESM.zip › Figure 2/2C/Figure2C_S.O_KI_PSD95.tif]

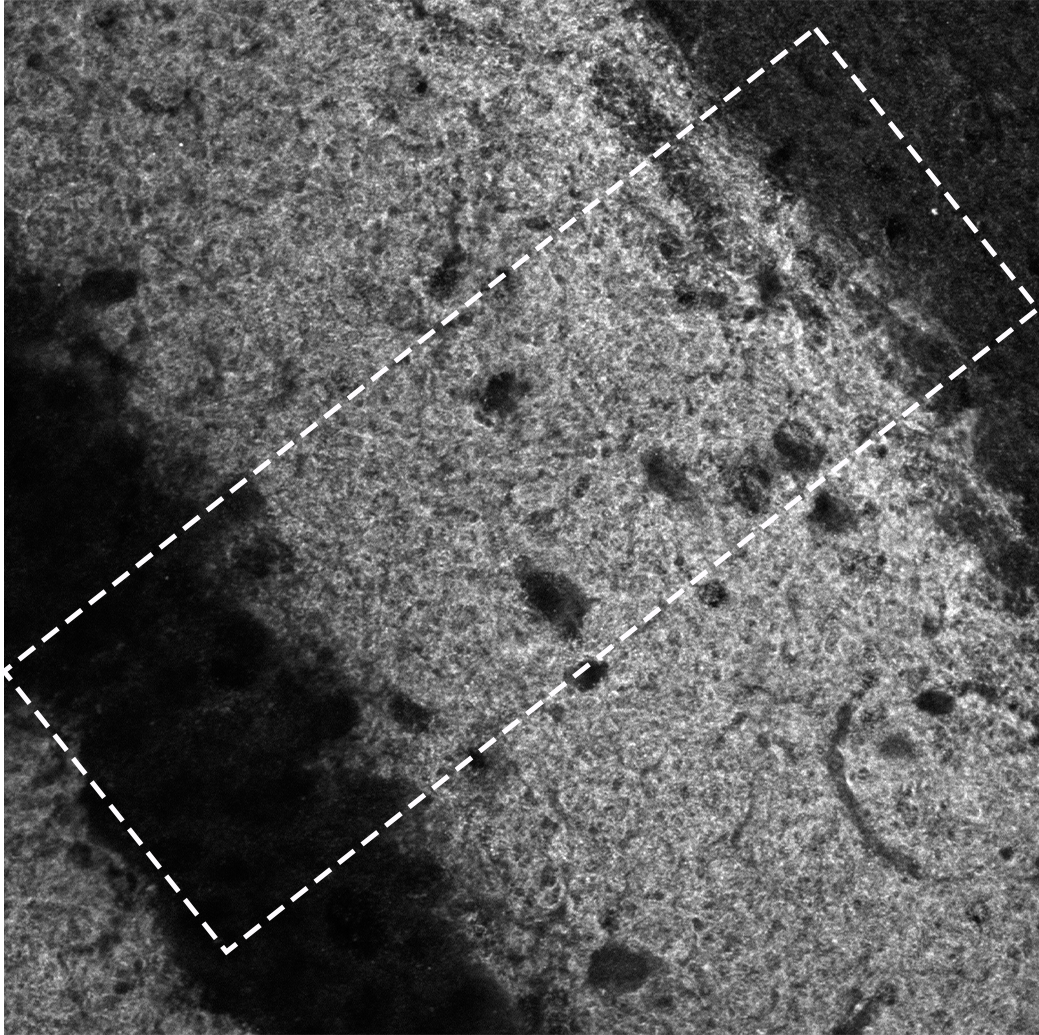

Supplement: Supplementary file 4 — Source data Fig. 2 [file 44318_2024_252_MOESM4_ESM.zip › Figure 2/2C/Figure2C_S.O_KI_VGLUT1 annotated.png]

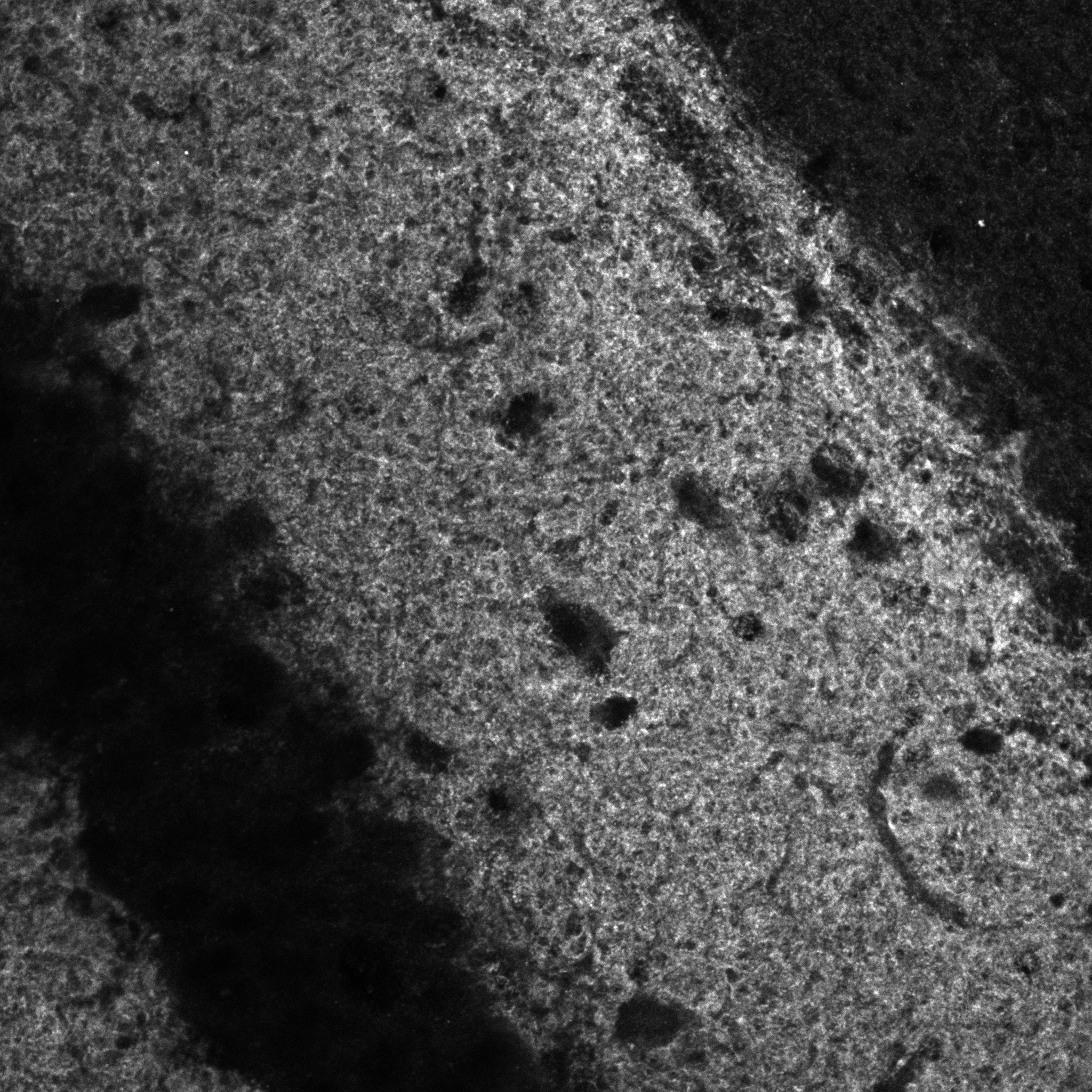

Supplement: Supplementary file 4 — Source data Fig. 2 [file 44318_2024_252_MOESM4_ESM.zip › Figure 2/2C/Figure2C_S.O_KI_VGLUT1.tif]

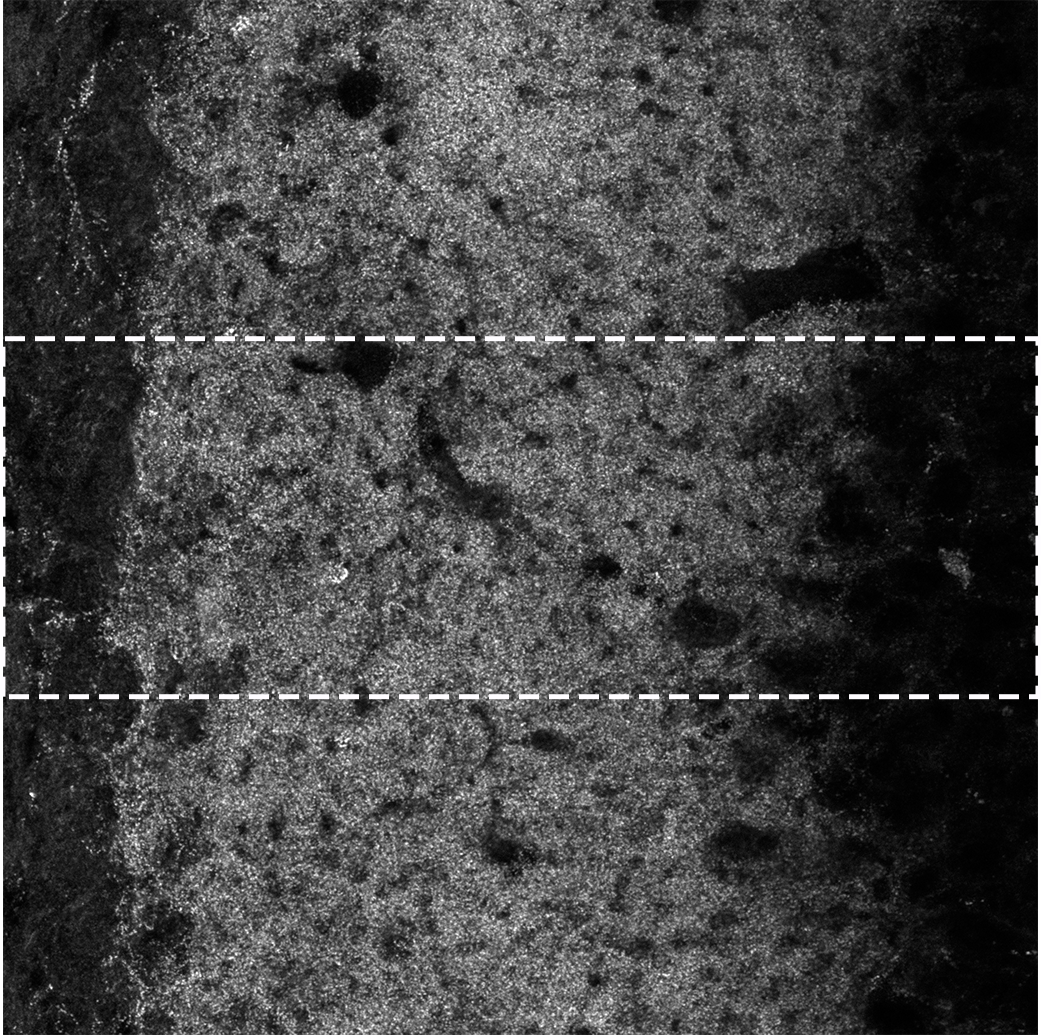

Supplement: Supplementary file 4 — Source data Fig. 2 [file 44318_2024_252_MOESM4_ESM.zip › Figure 2/2C/Figure2C_S.O_WT_PSD95 annotated.png]

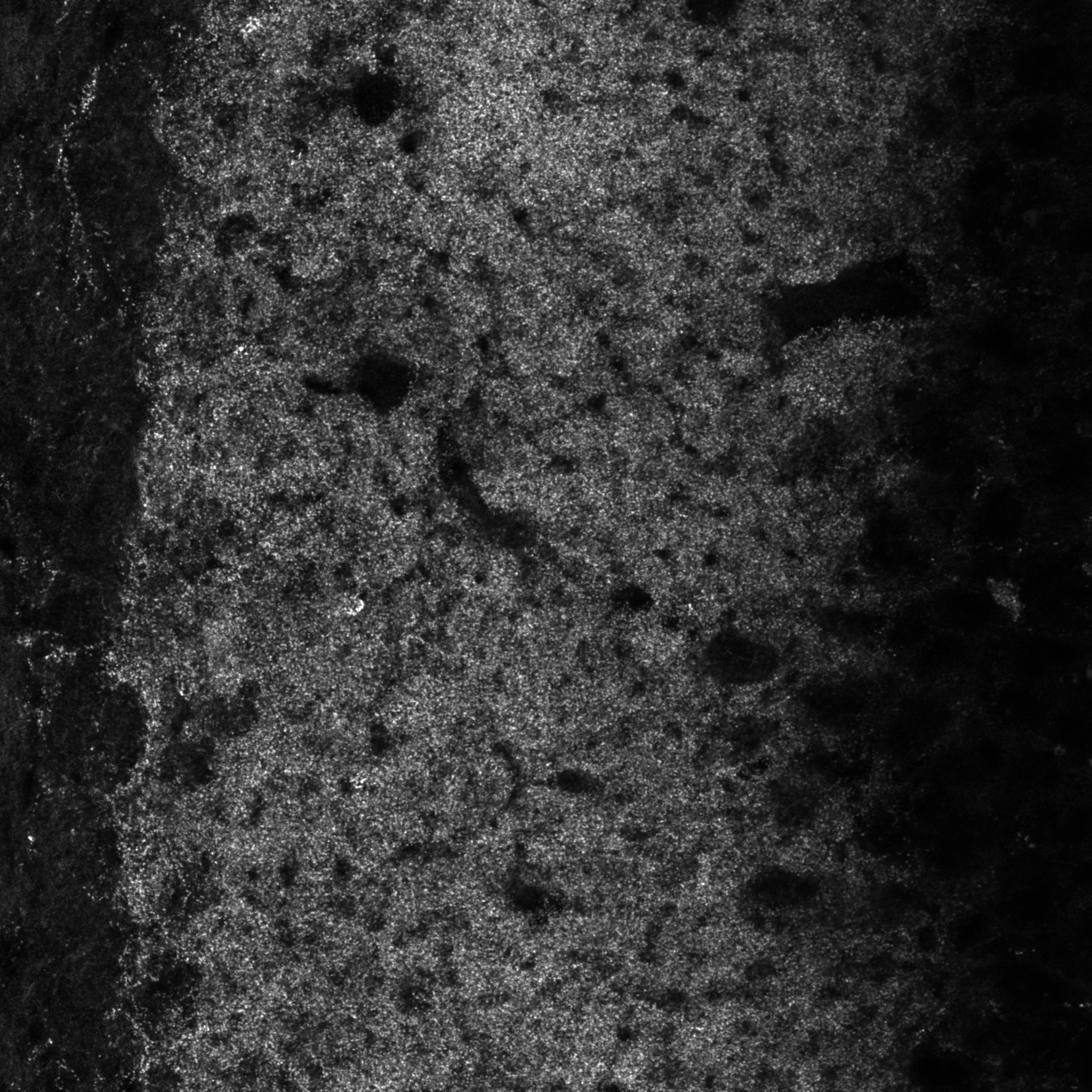

Supplement: Supplementary file 4 — Source data Fig. 2 [file 44318_2024_252_MOESM4_ESM.zip › Figure 2/2C/Figure2C_S.O_WT_PSD95.tif]

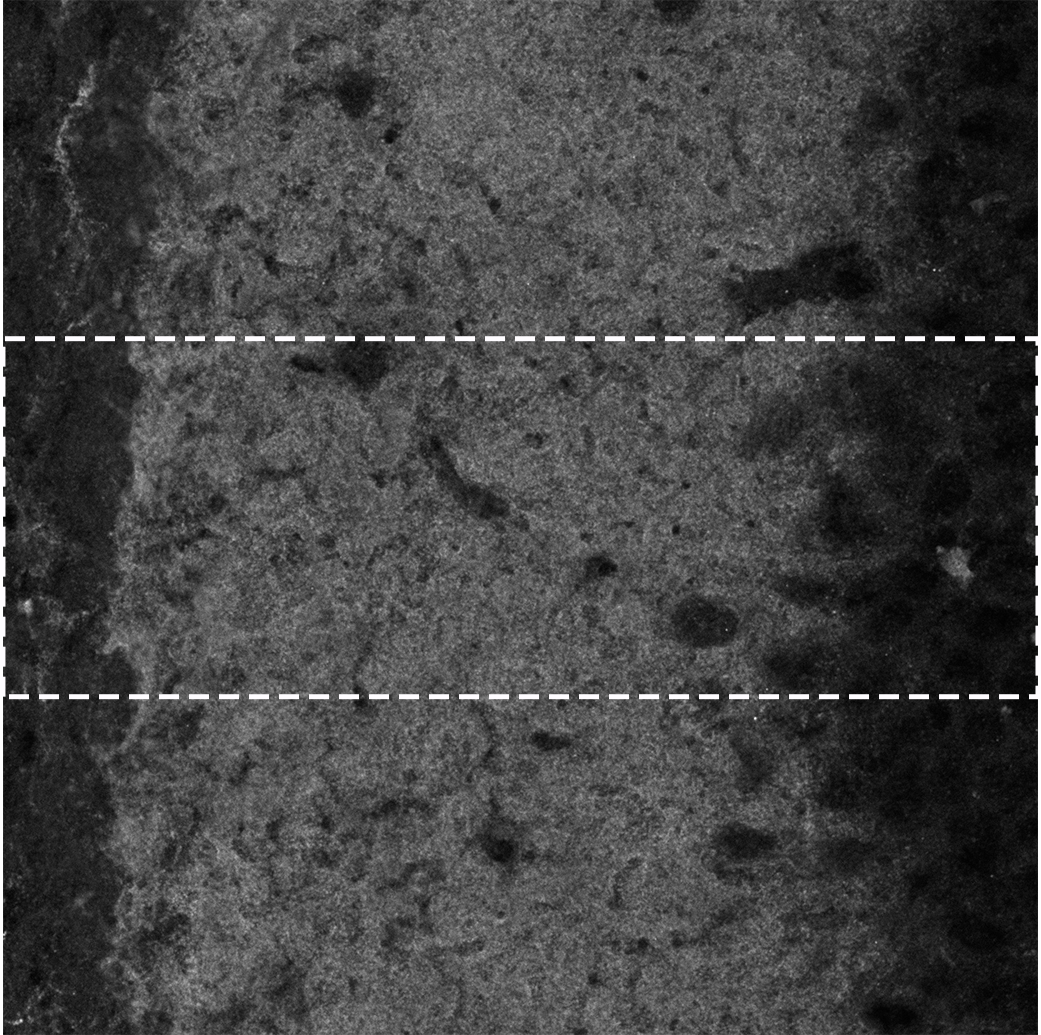

Supplement: Supplementary file 4 — Source data Fig. 2 [file 44318_2024_252_MOESM4_ESM.zip › Figure 2/2C/Figure2C_S.O_WT_VGLUT1 annotated.png]

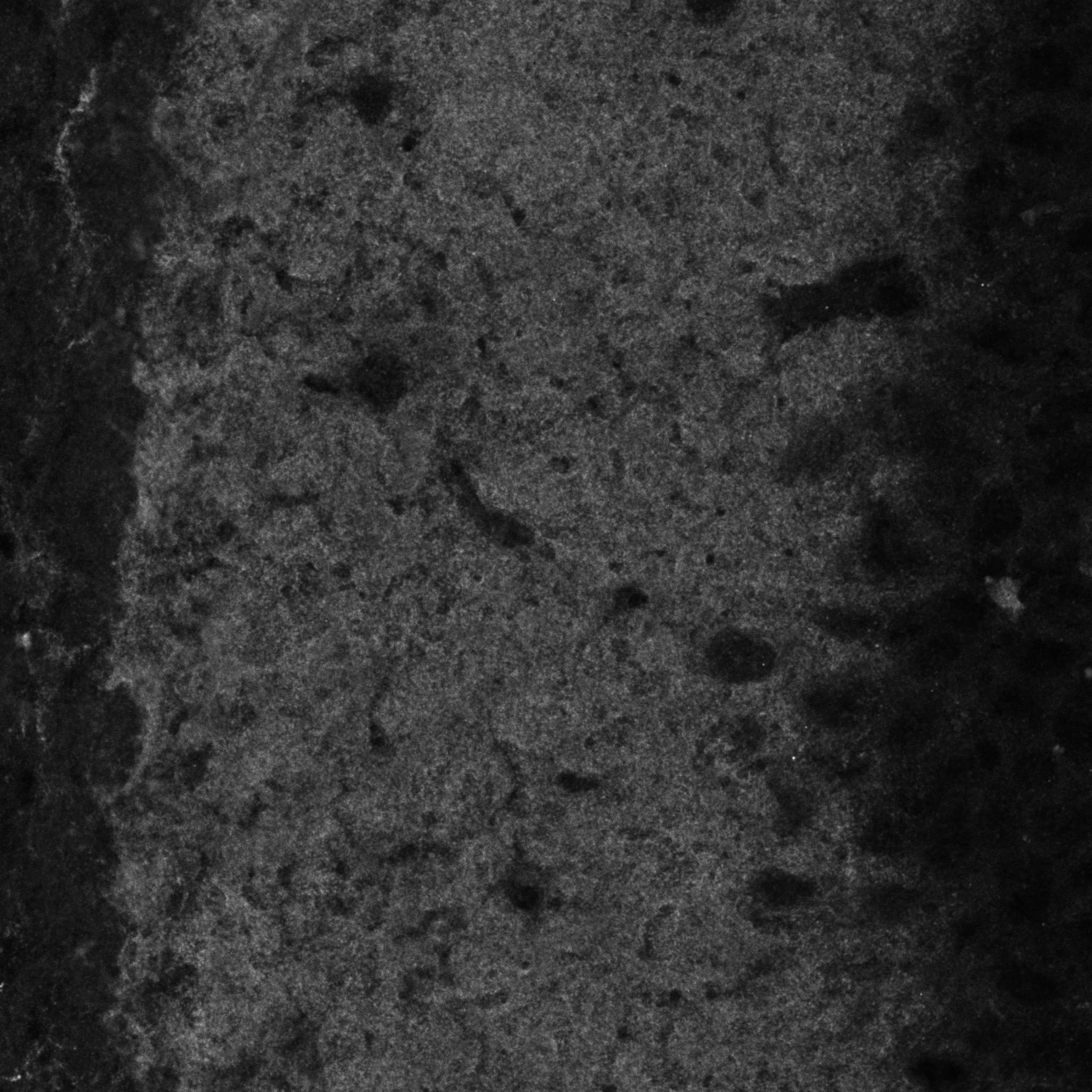

Supplement: Supplementary file 4 — Source data Fig. 2 [file 44318_2024_252_MOESM4_ESM.zip › Figure 2/2C/Figure2C_S.O_WT_VGLUT1.tif]

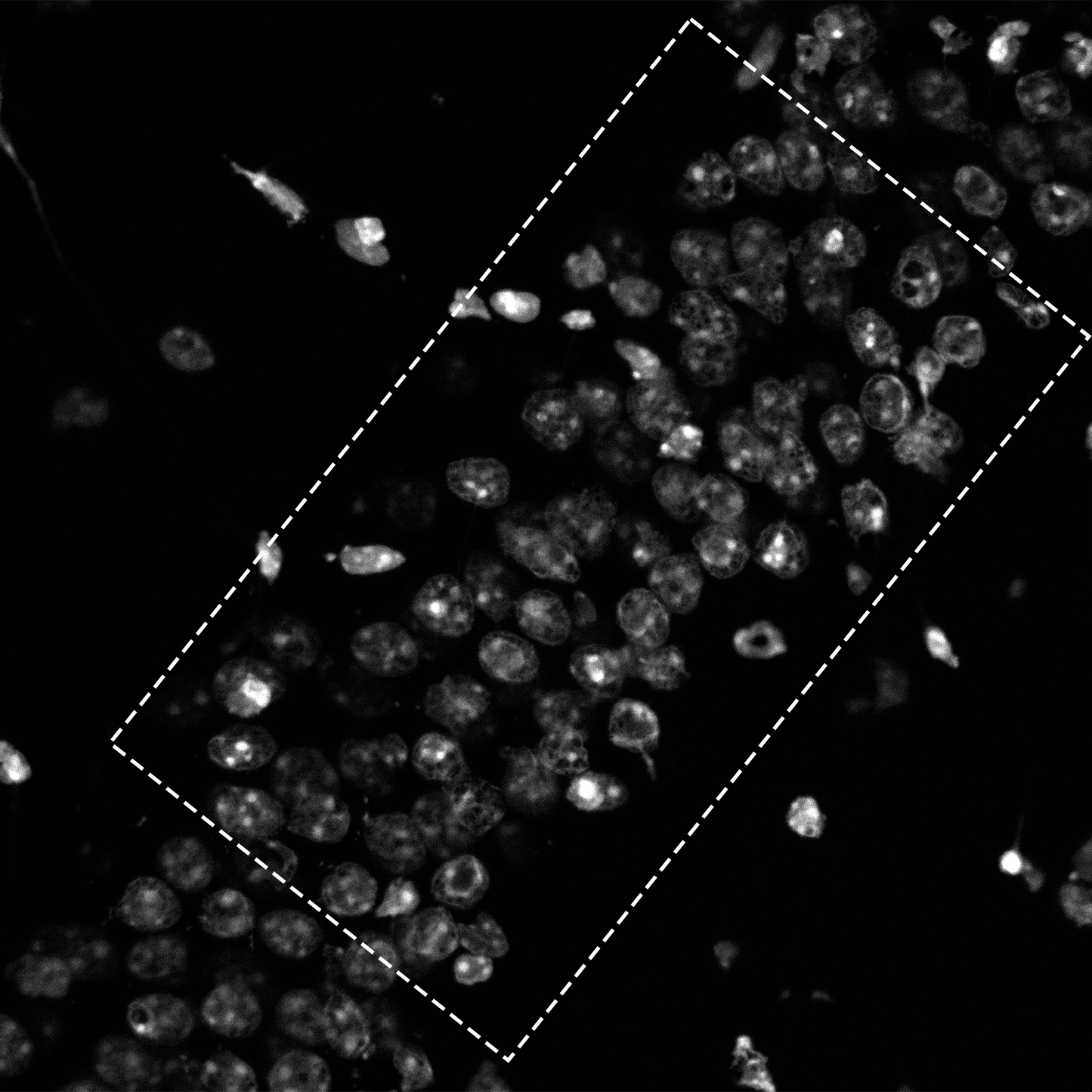

Supplement: Supplementary file 4 — Source data Fig. 2 [file 44318_2024_252_MOESM4_ESM.zip › Figure 2/2D/Figure2D_Dapi_KI annotated.png]

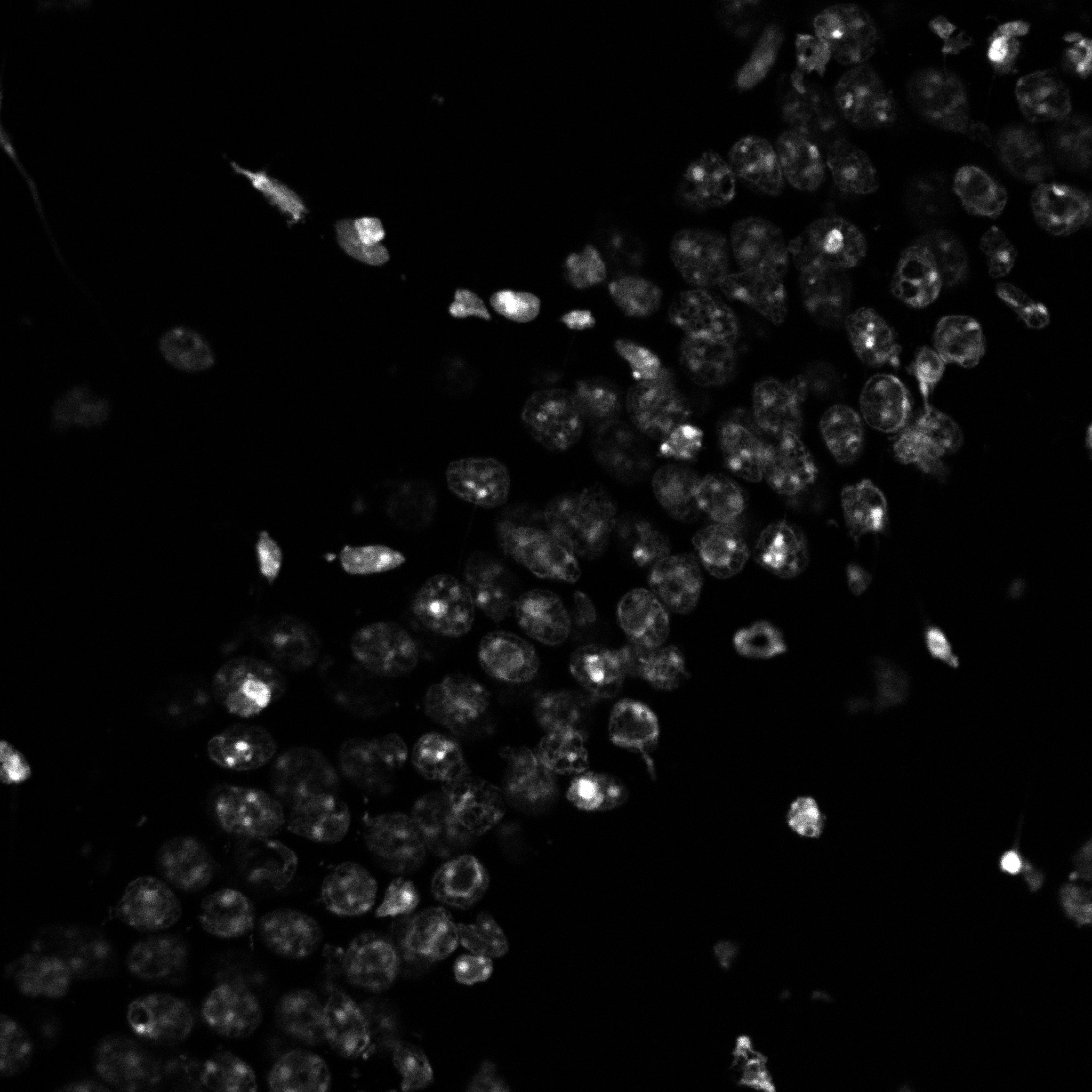

Supplement: Supplementary file 4 — Source data Fig. 2 [file 44318_2024_252_MOESM4_ESM.zip › Figure 2/2D/Figure2D_Dapi_KI.tif]

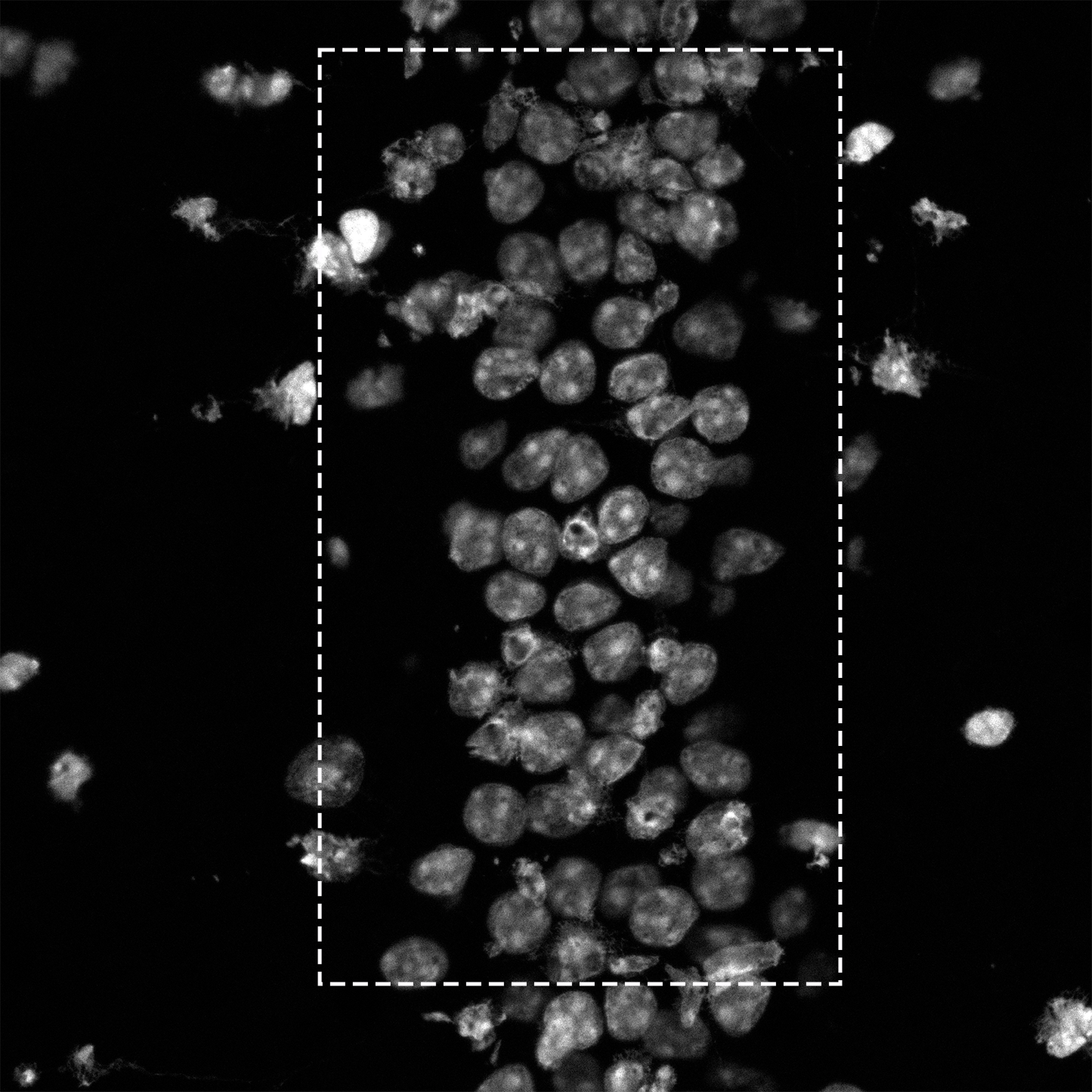

Supplement: Supplementary file 4 — Source data Fig. 2 [file 44318_2024_252_MOESM4_ESM.zip › Figure 2/2D/Figure2D_Dapi_WT annotated.png]

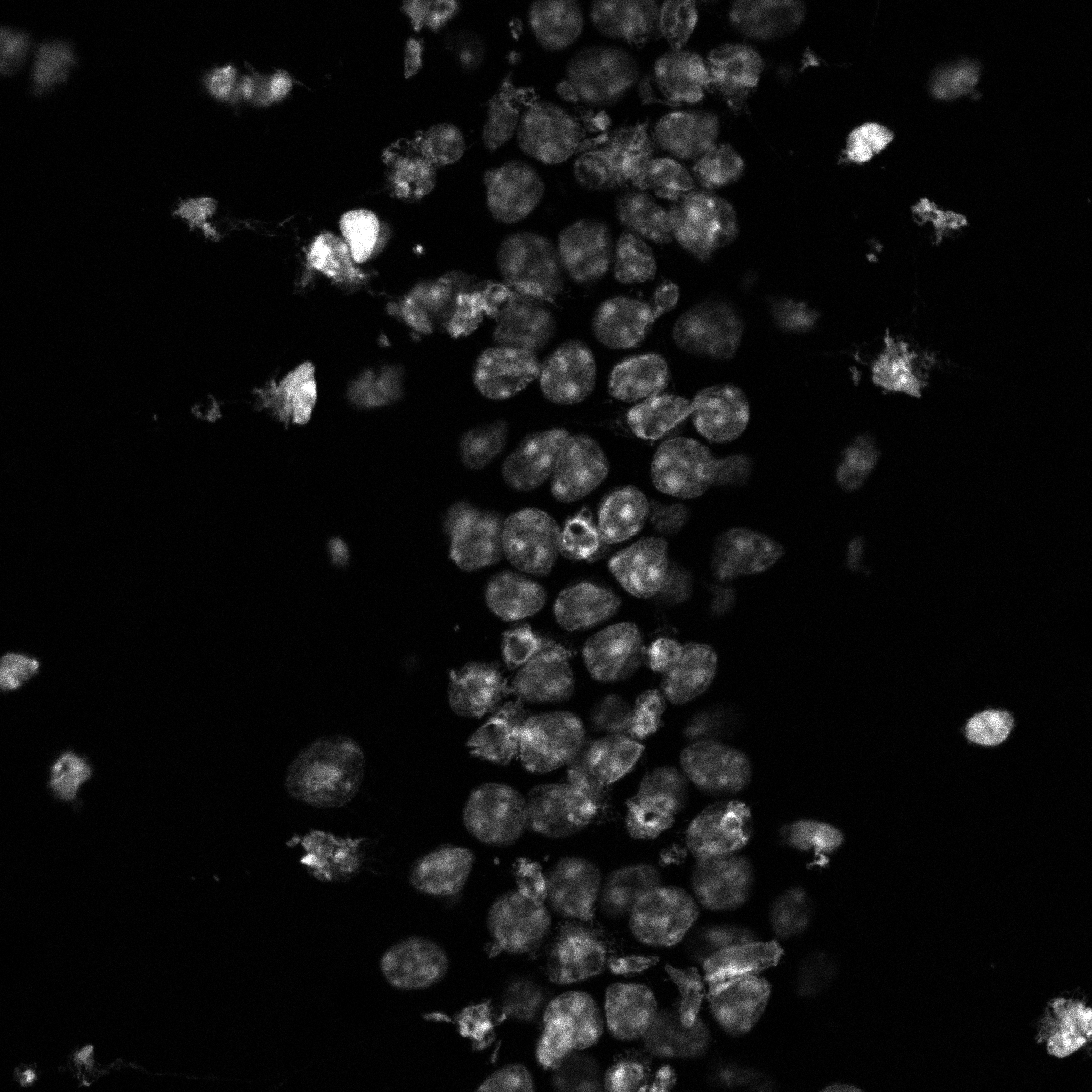

Supplement: Supplementary file 4 — Source data Fig. 2 [file 44318_2024_252_MOESM4_ESM.zip › Figure 2/2D/Figure2D_Dapi_WT.tif]

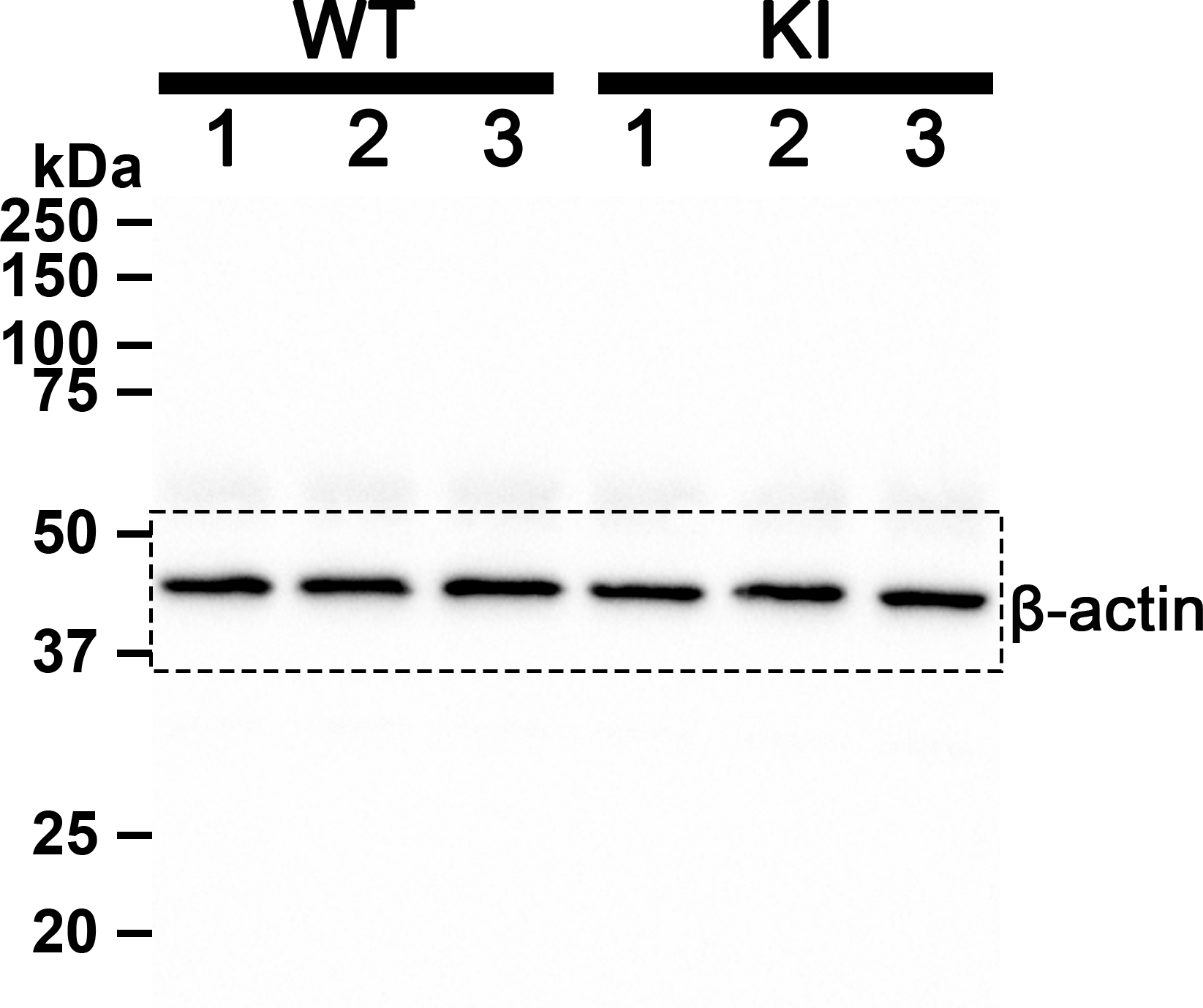

Supplement: Supplementary file 4 — Source data Fig. 2 [file 44318_2024_252_MOESM4_ESM.zip › Figure 2/2E/Figure2E_b-actin annotated.png]

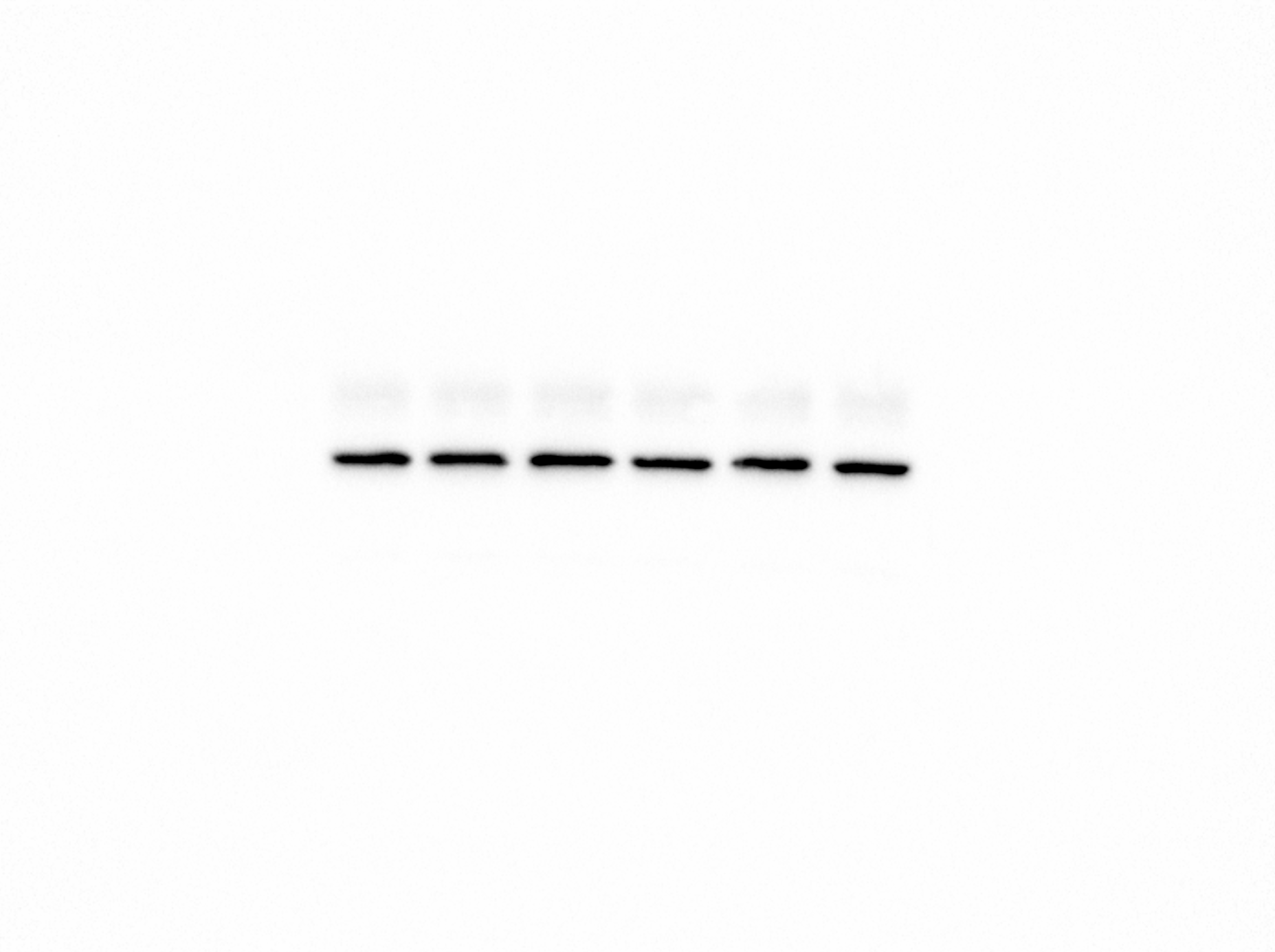

Supplement: Supplementary file 4 — Source data Fig. 2 [file 44318_2024_252_MOESM4_ESM.zip › Figure 2/2E/Figure2E_b-actin.tif]

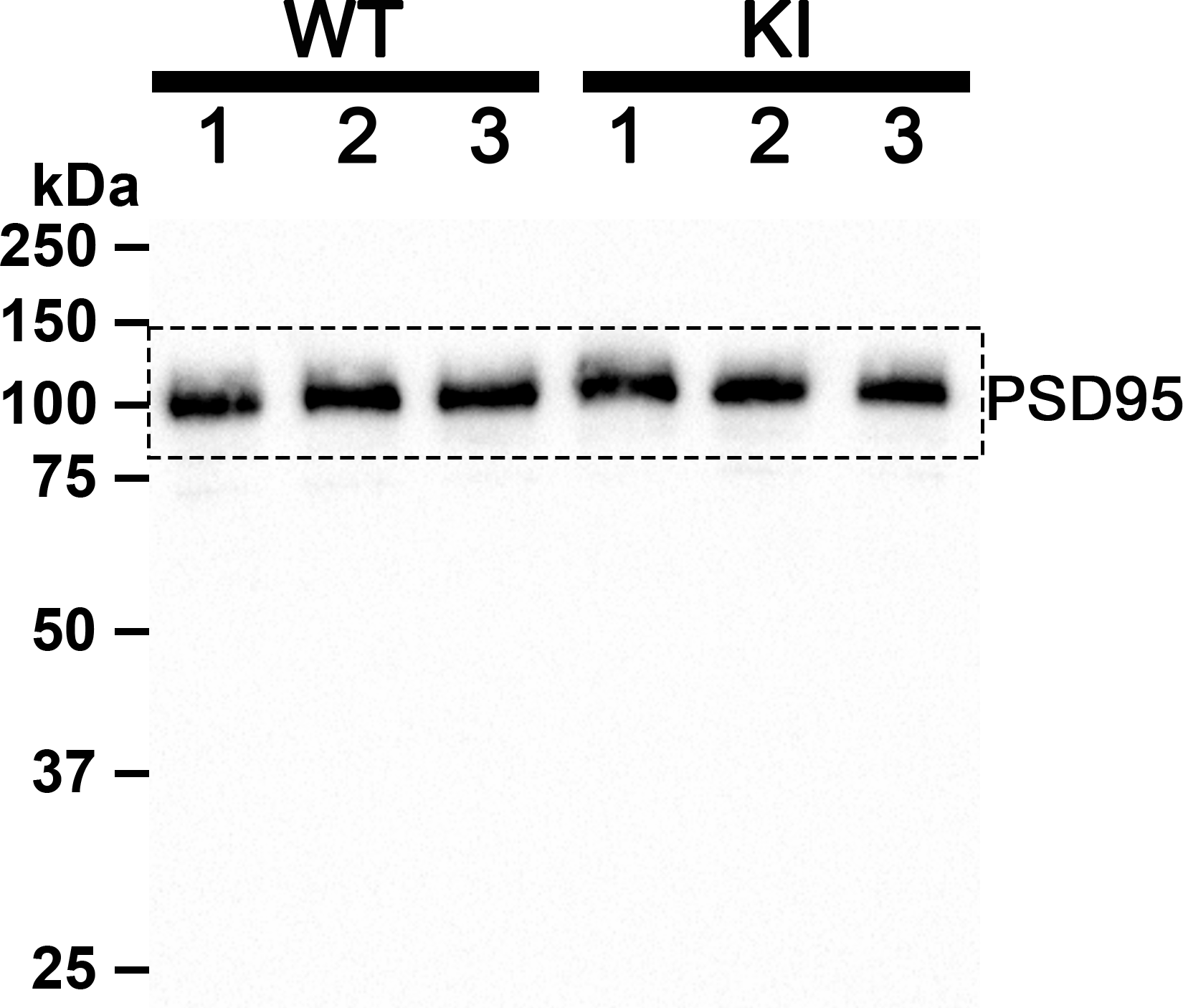

Supplement: Supplementary file 4 — Source data Fig. 2 [file 44318_2024_252_MOESM4_ESM.zip › Figure 2/2E/Figure2E_PSD95 annotated.png]

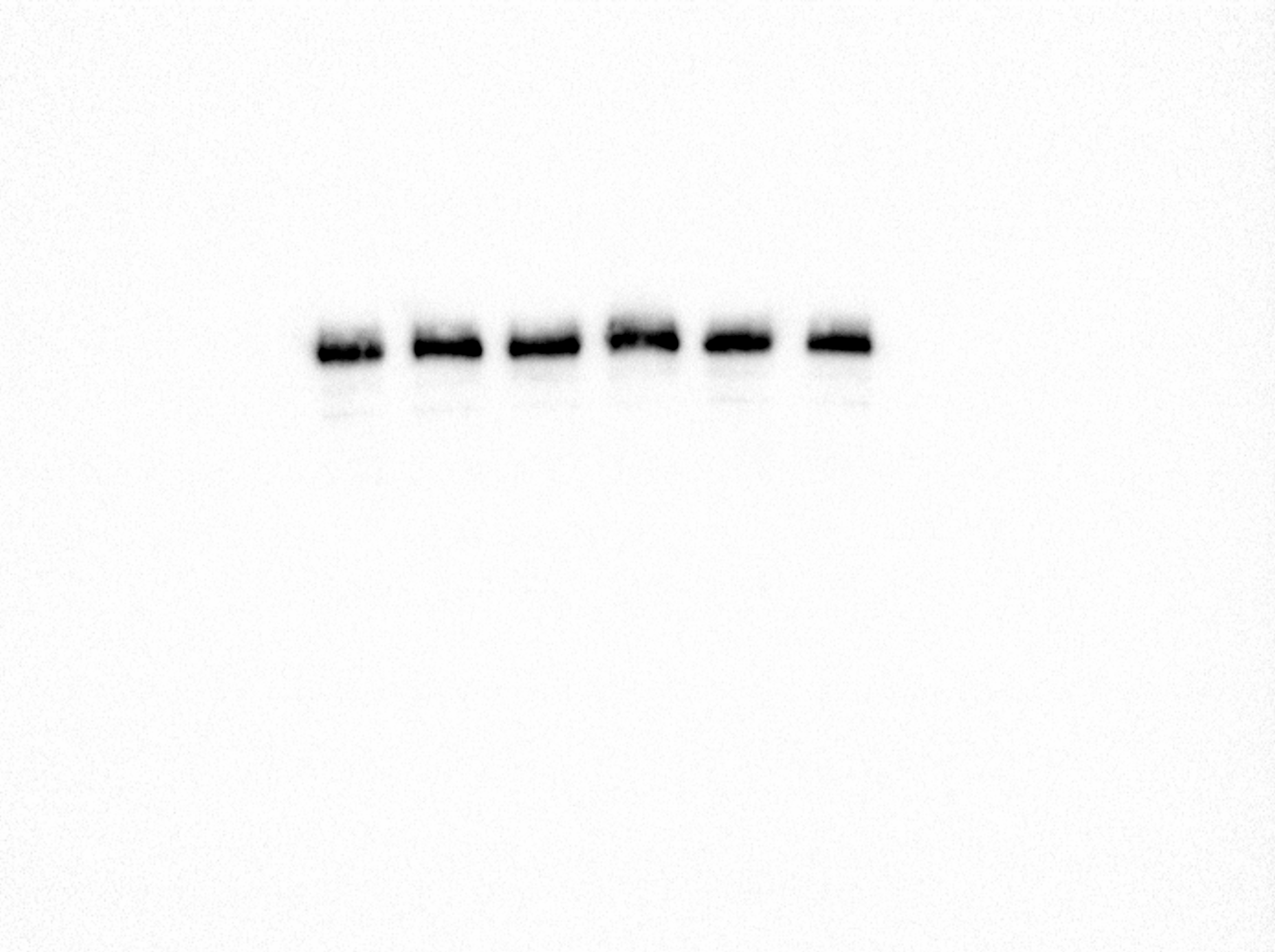

Supplement: Supplementary file 4 — Source data Fig. 2 [file 44318_2024_252_MOESM4_ESM.zip › Figure 2/2E/Figure2E_PSD95.tif]

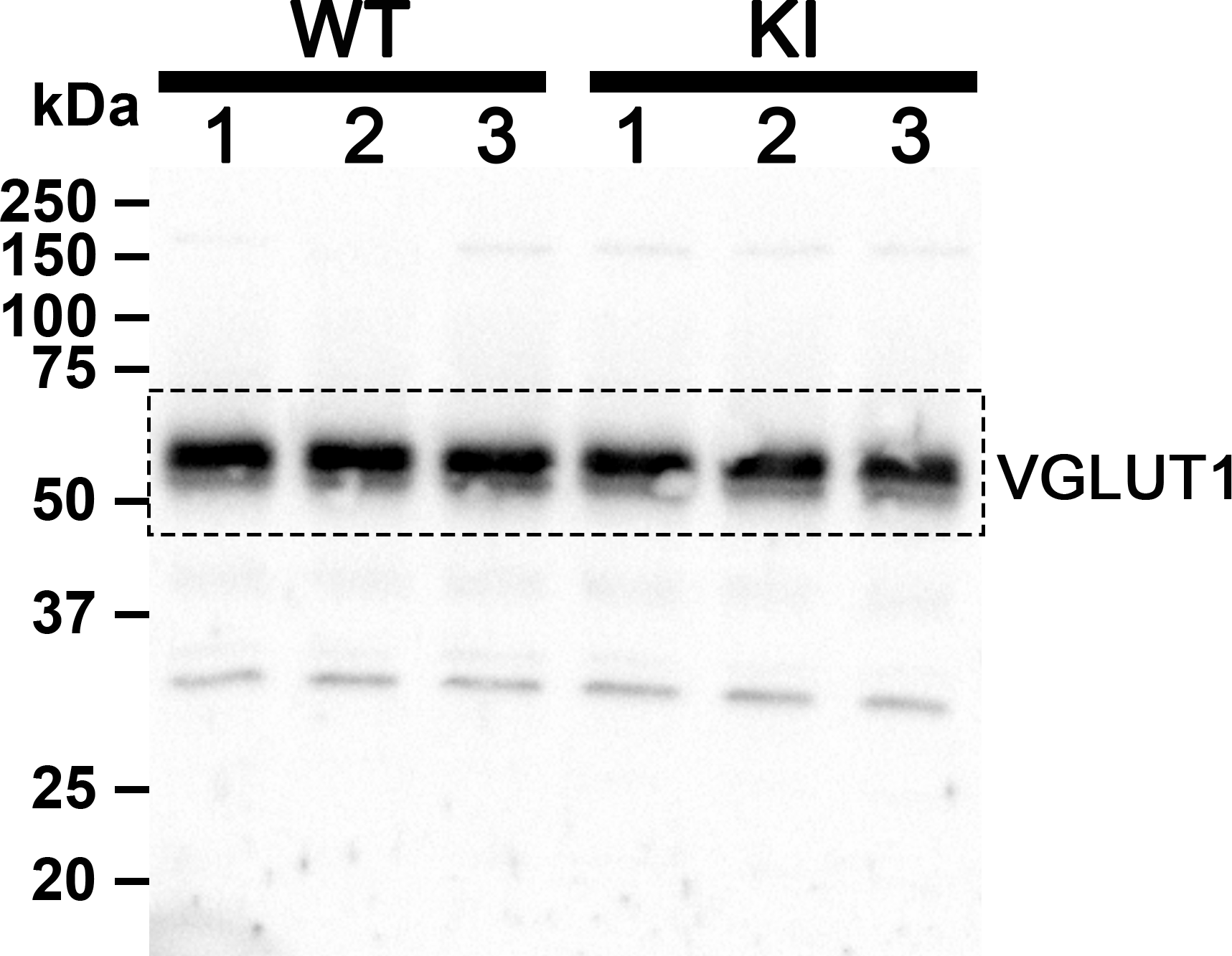

Supplement: Supplementary file 4 — Source data Fig. 2 [file 44318_2024_252_MOESM4_ESM.zip › Figure 2/2E/Figure2E_VGLUT1 annotated.png]

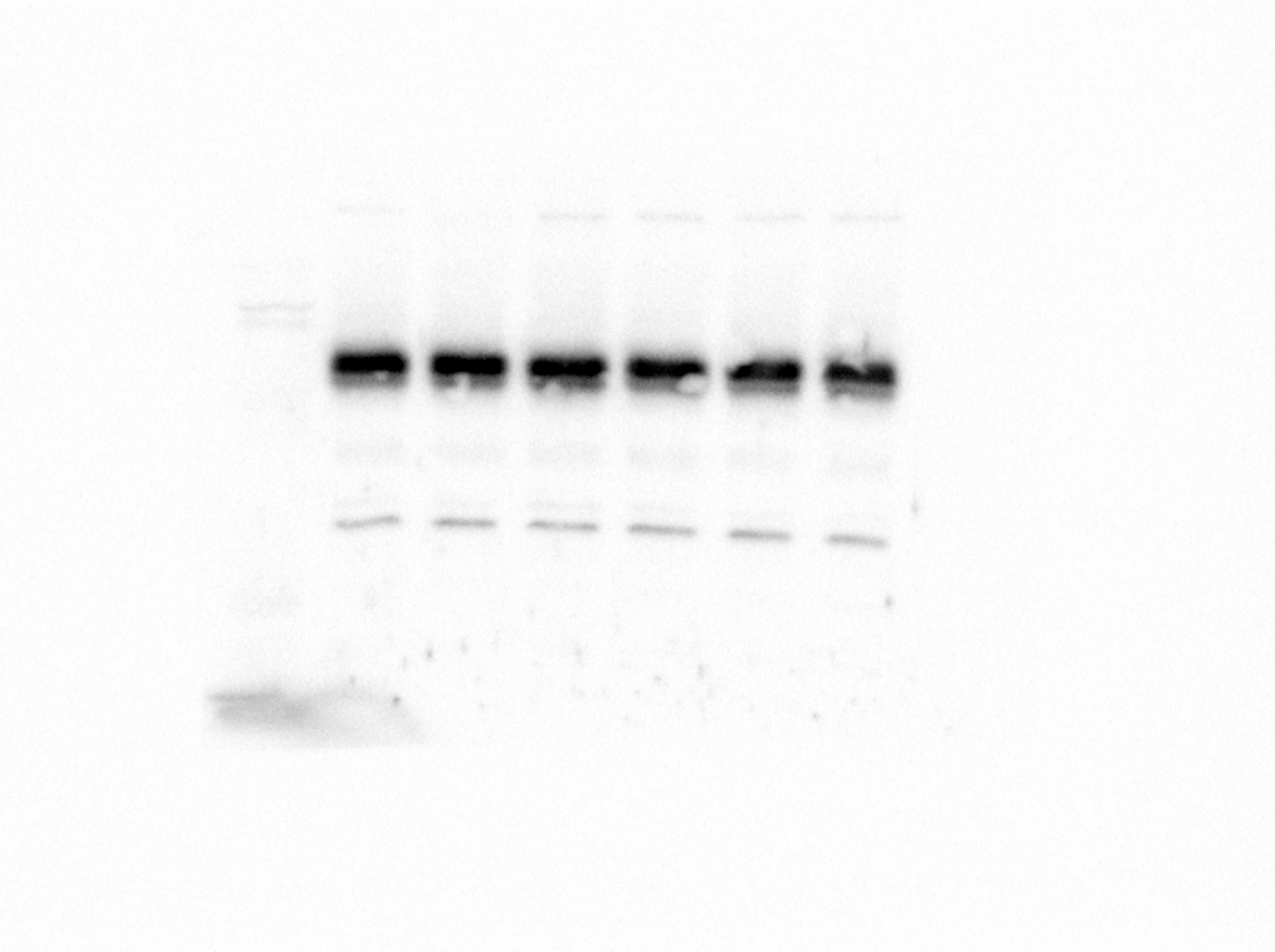

Supplement: Supplementary file 4 — Source data Fig. 2 [file 44318_2024_252_MOESM4_ESM.zip › Figure 2/2E/Figure2E_VGLUT1.tif]

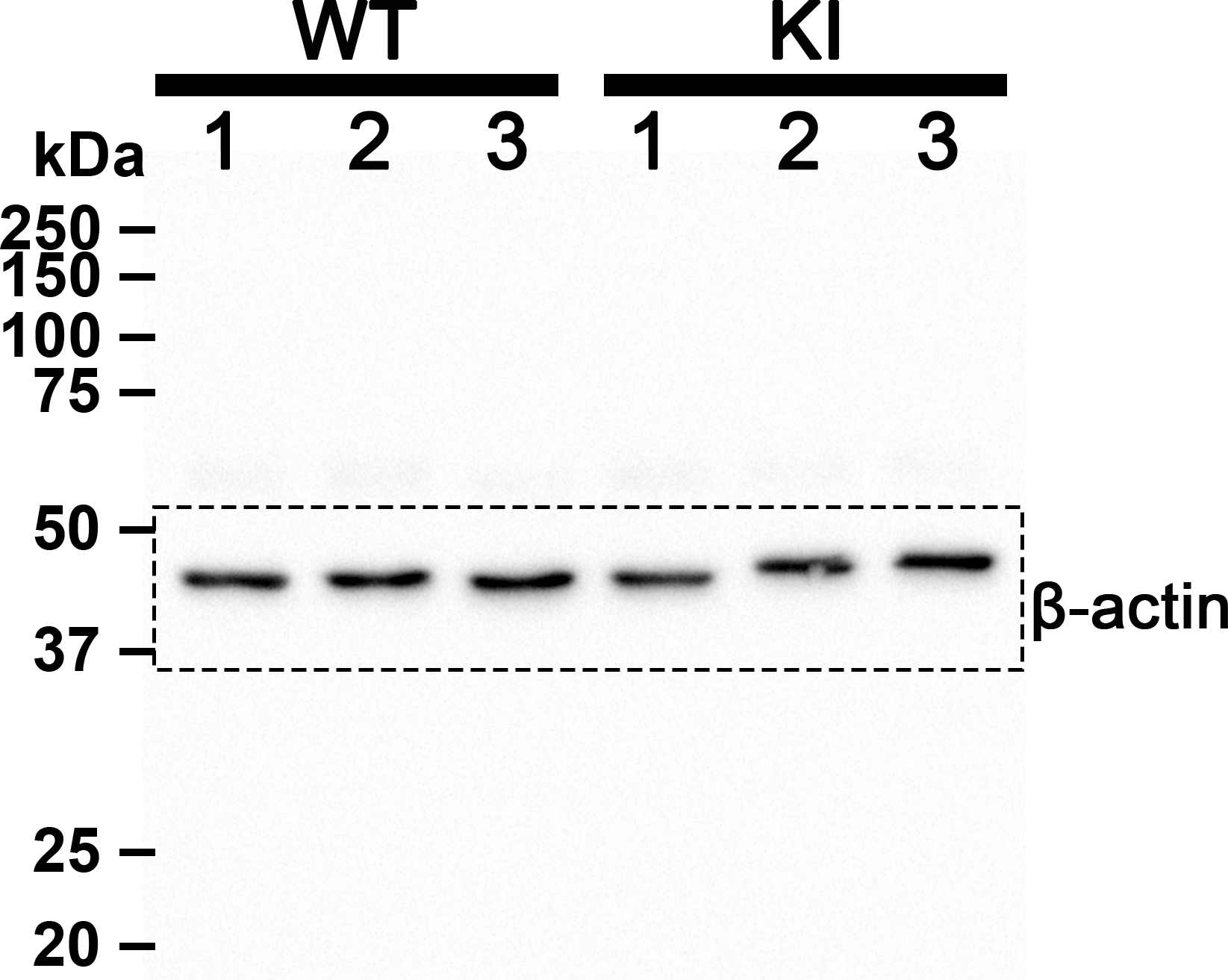

Supplement: Supplementary file 4 — Source data Fig. 2 [file 44318_2024_252_MOESM4_ESM.zip › Figure 2/2F/Figure2F_b-actin annotated.png]

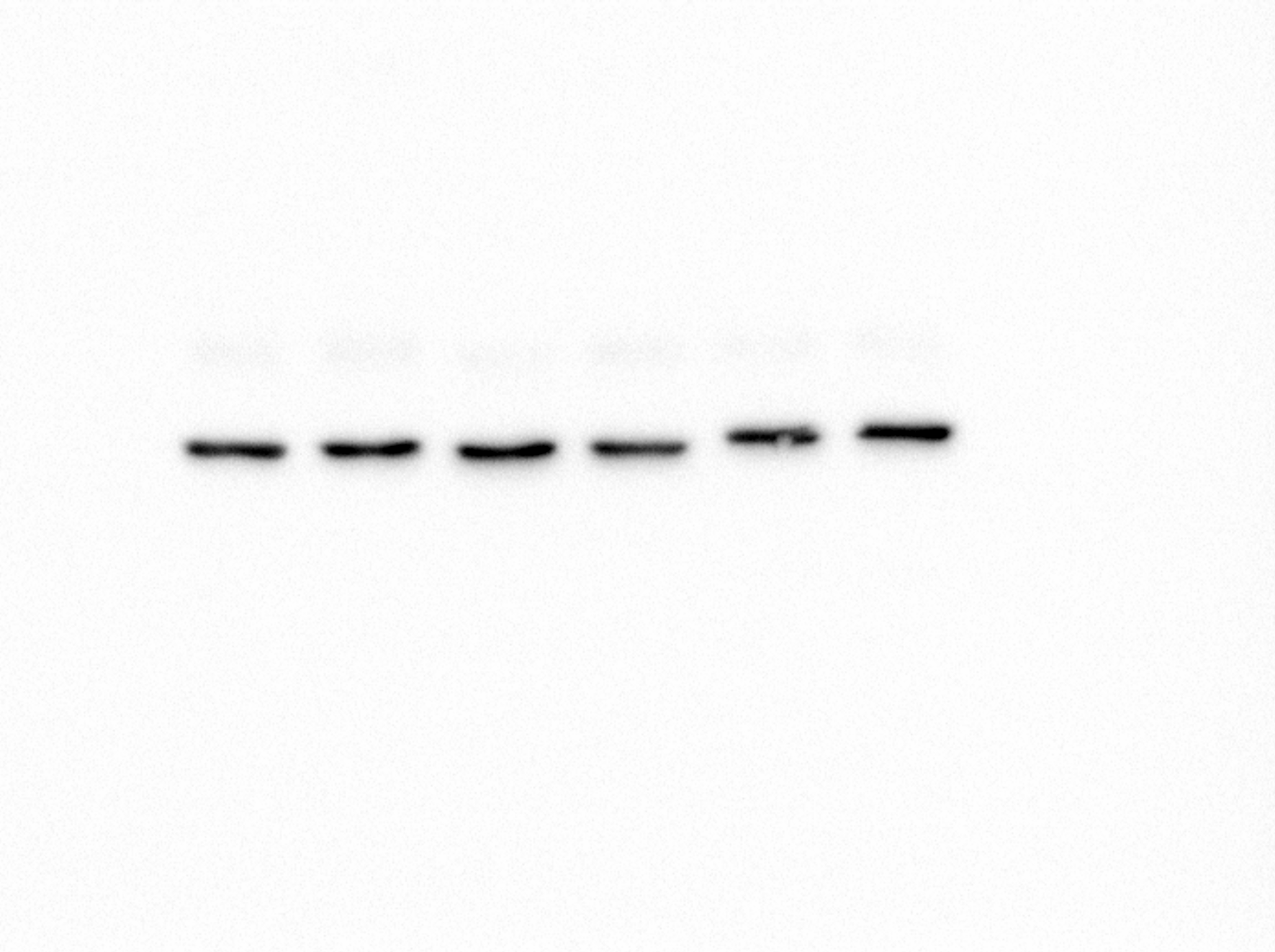

Supplement: Supplementary file 4 — Source data Fig. 2 [file 44318_2024_252_MOESM4_ESM.zip › Figure 2/2F/Figure2F_b-actin.tif]

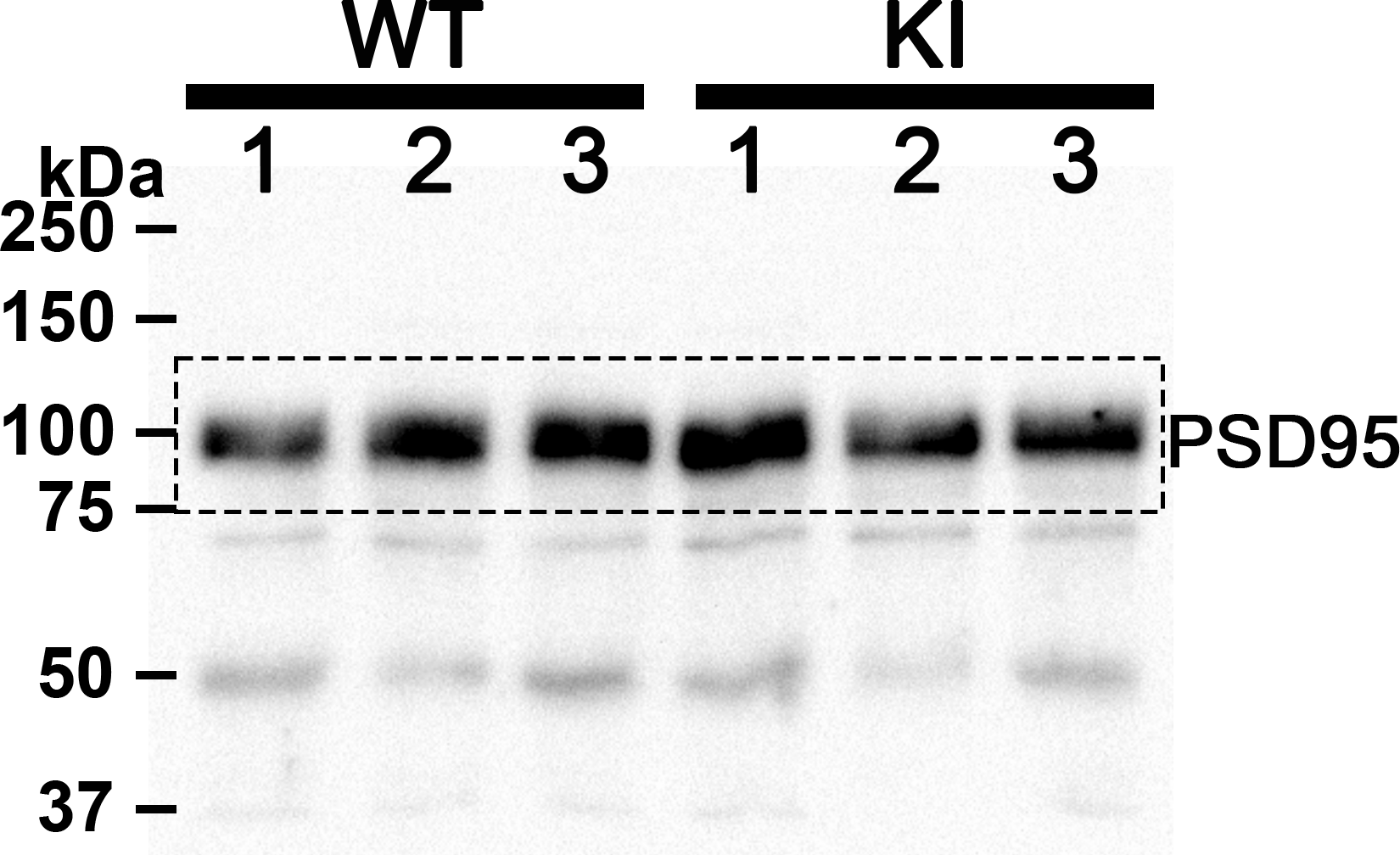

Supplement: Supplementary file 4 — Source data Fig. 2 [file 44318_2024_252_MOESM4_ESM.zip › Figure 2/2F/Figure2F_PSD95 annotated.png]

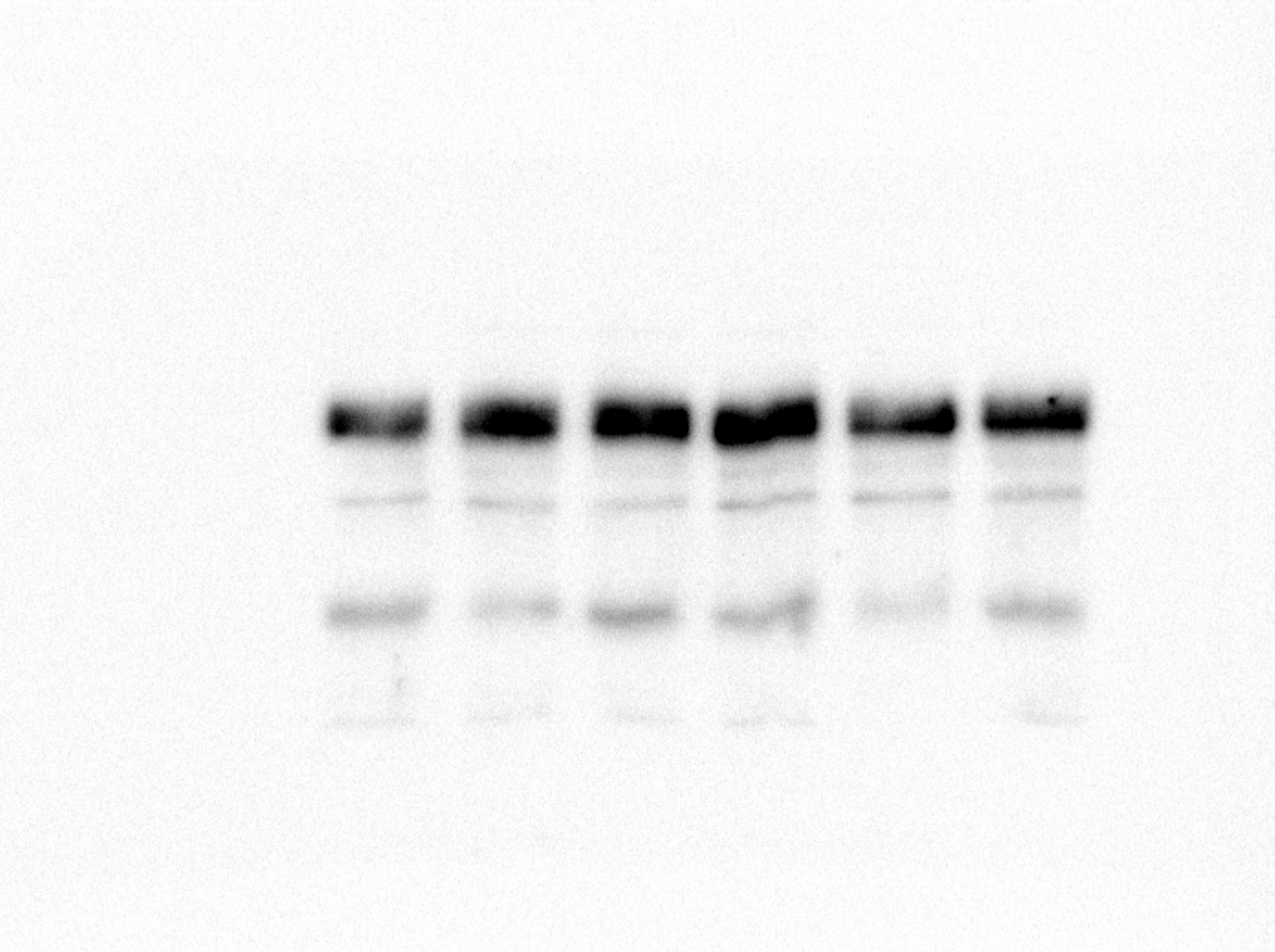

Supplement: Supplementary file 4 — Source data Fig. 2 [file 44318_2024_252_MOESM4_ESM.zip › Figure 2/2F/Figure2F_PSD95.tif]

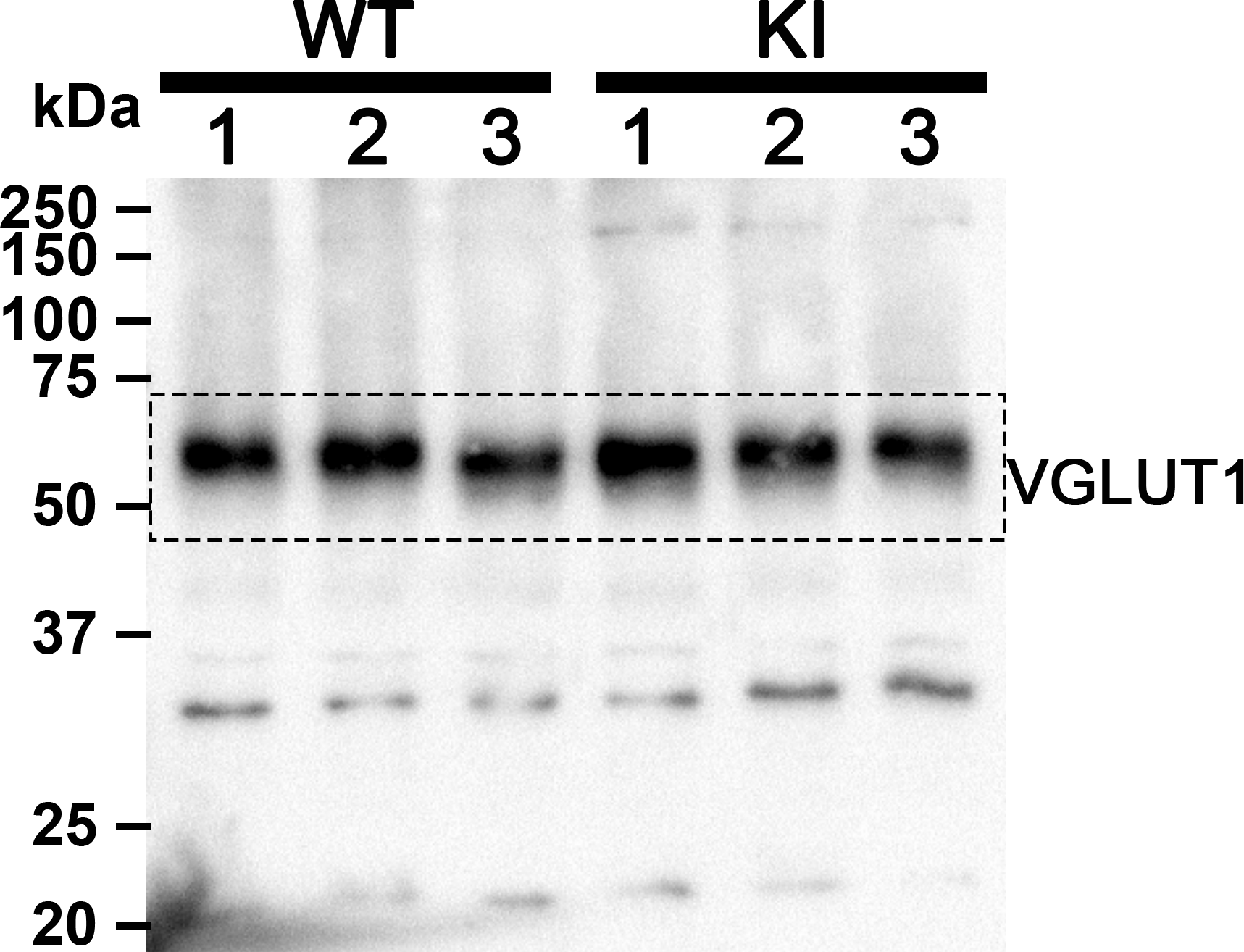

Supplement: Supplementary file 4 — Source data Fig. 2 [file 44318_2024_252_MOESM4_ESM.zip › Figure 2/2F/Figure2F_VGLUT1 annotated.png]

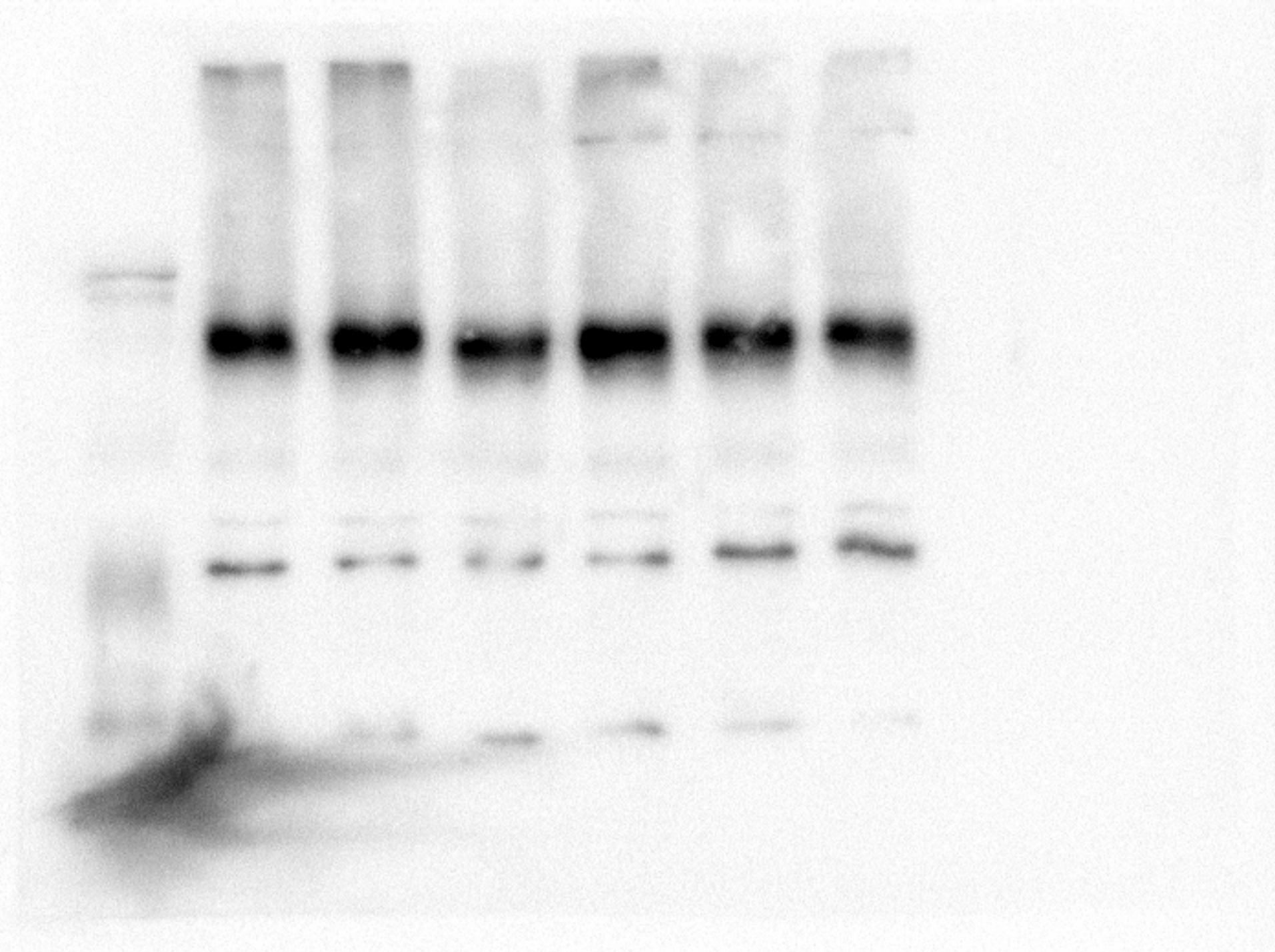

Supplement: Supplementary file 4 — Source data Fig. 2 [file 44318_2024_252_MOESM4_ESM.zip › Figure 2/2F/Figure2F_VGLUT1.tif]

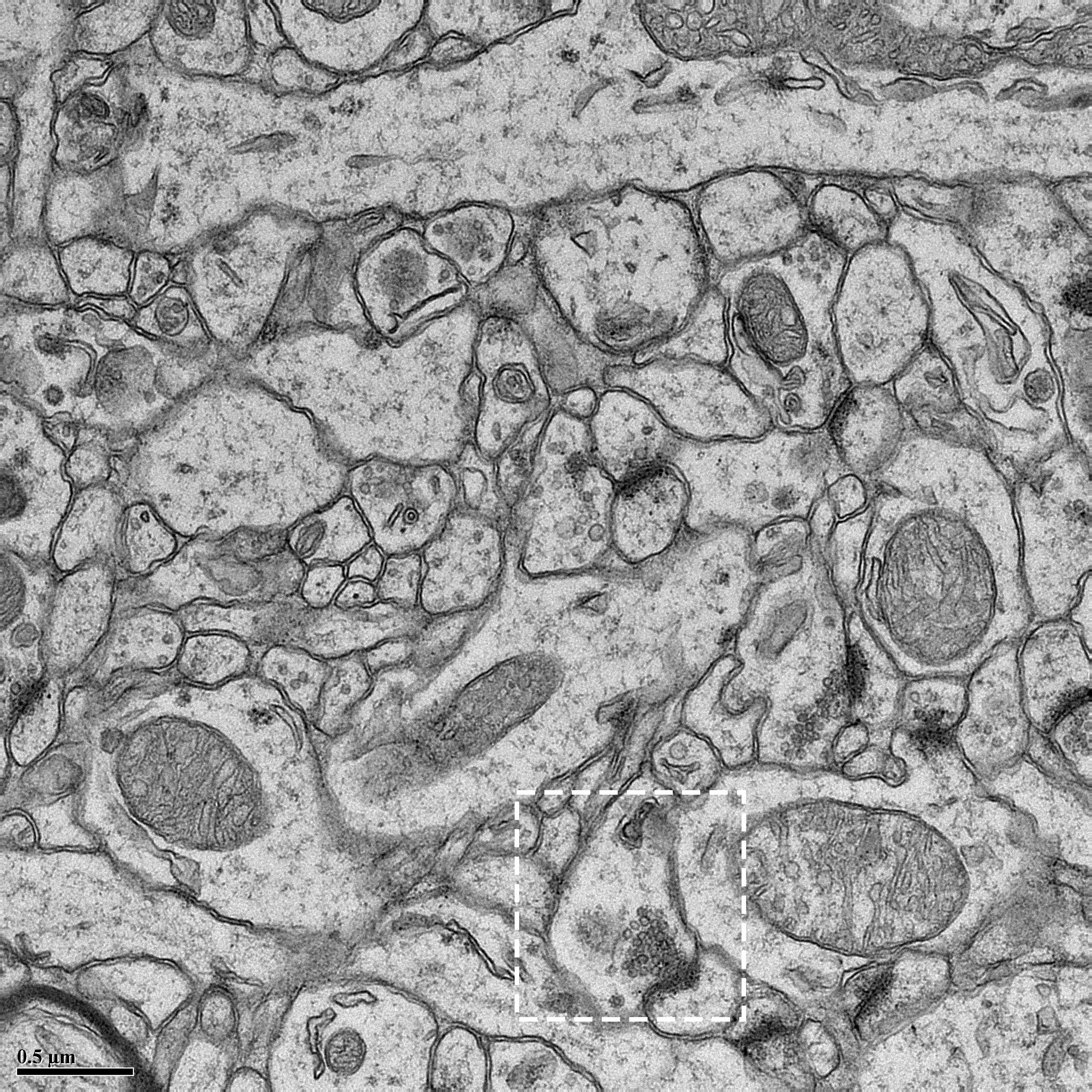

Supplement: Supplementary file 5 — Source data Fig. 3 [file 44318_2024_252_MOESM5_ESM.zip › Figure 3/3D/Figure3D_KI_s.o annotated.png]

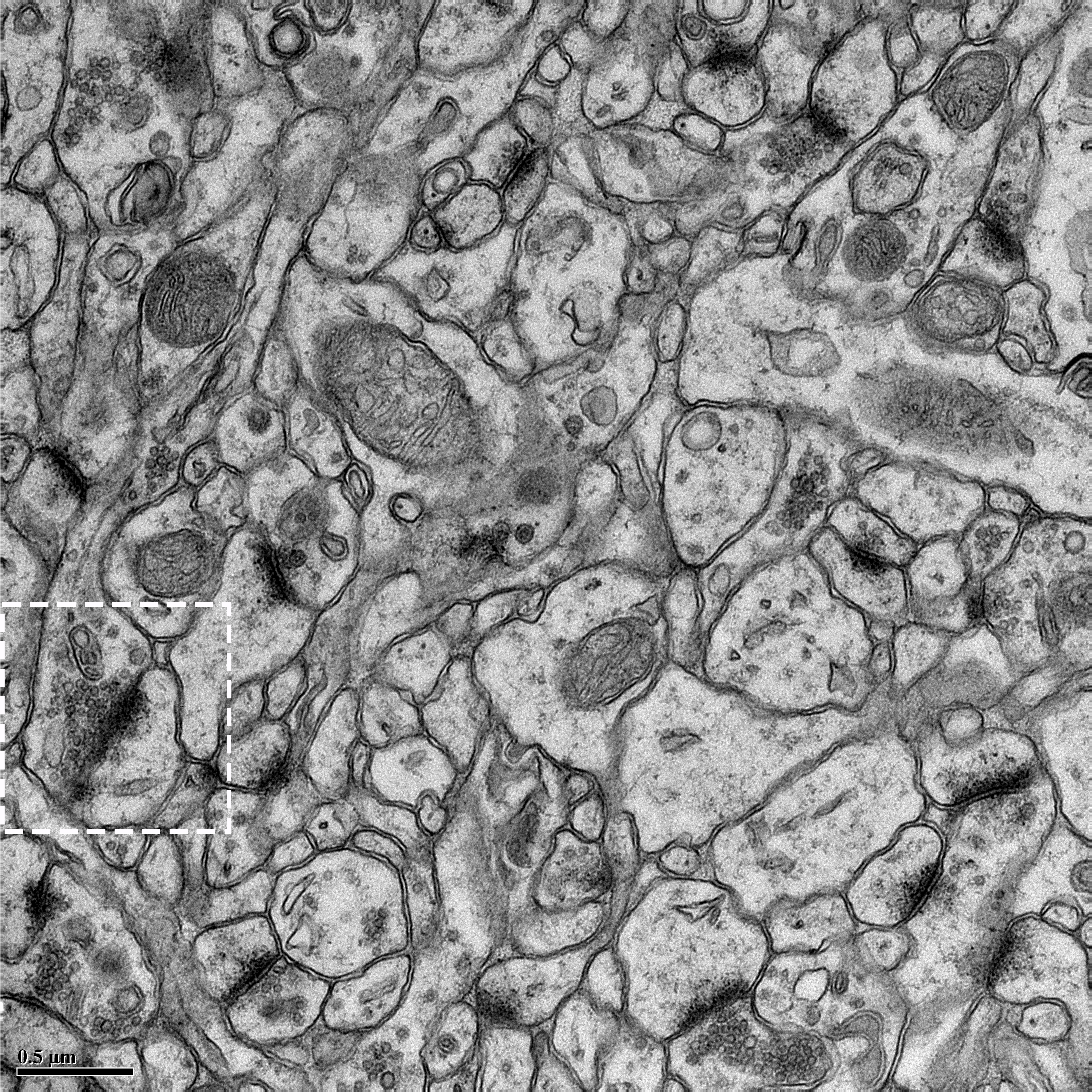

Supplement: Supplementary file 5 — Source data Fig. 3 [file 44318_2024_252_MOESM5_ESM.zip › Figure 3/3D/Figure3D_KI_s.r annotated.png]

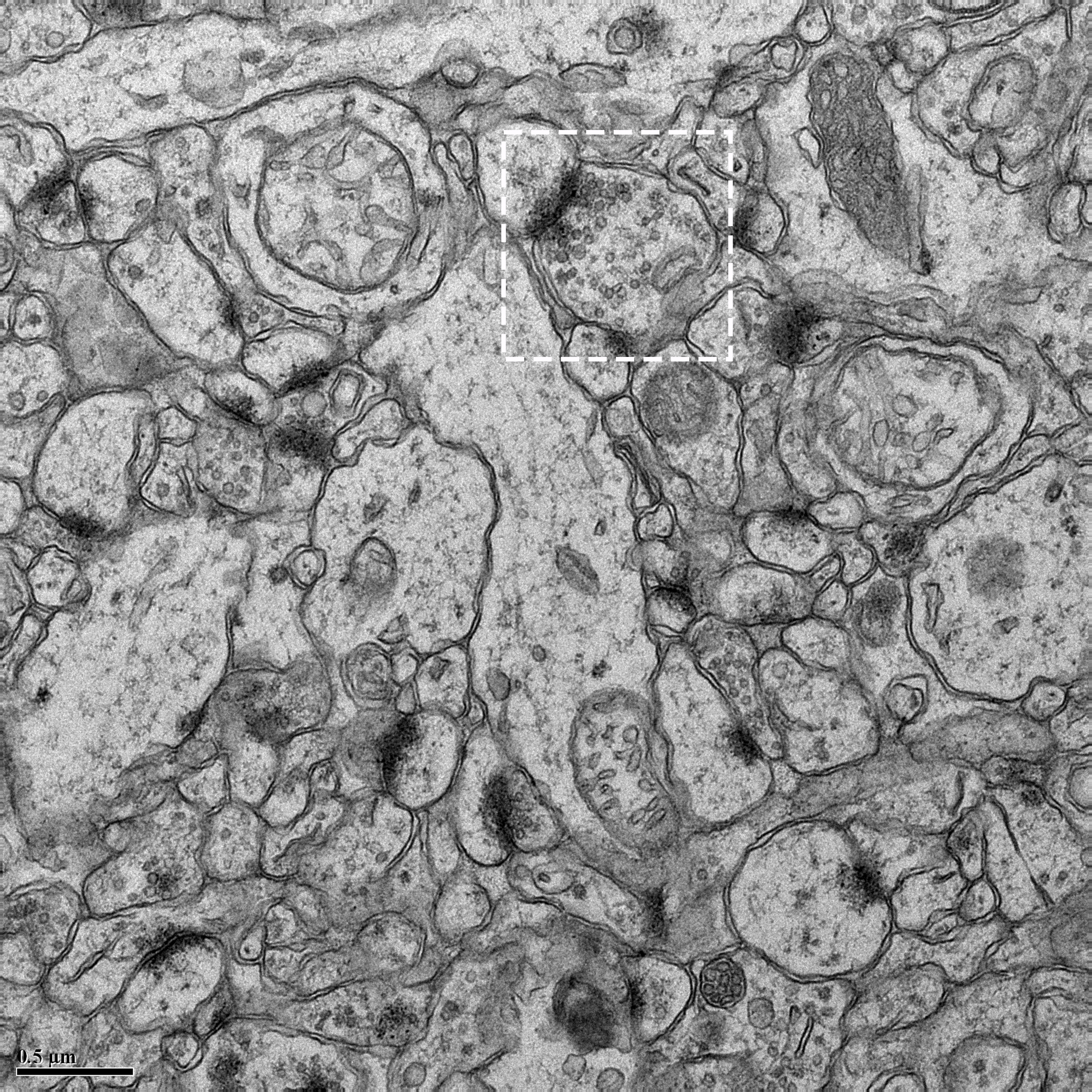

Supplement: Supplementary file 5 — Source data Fig. 3 [file 44318_2024_252_MOESM5_ESM.zip › Figure 3/3D/Figure3D_WT_s.o annotated.png]

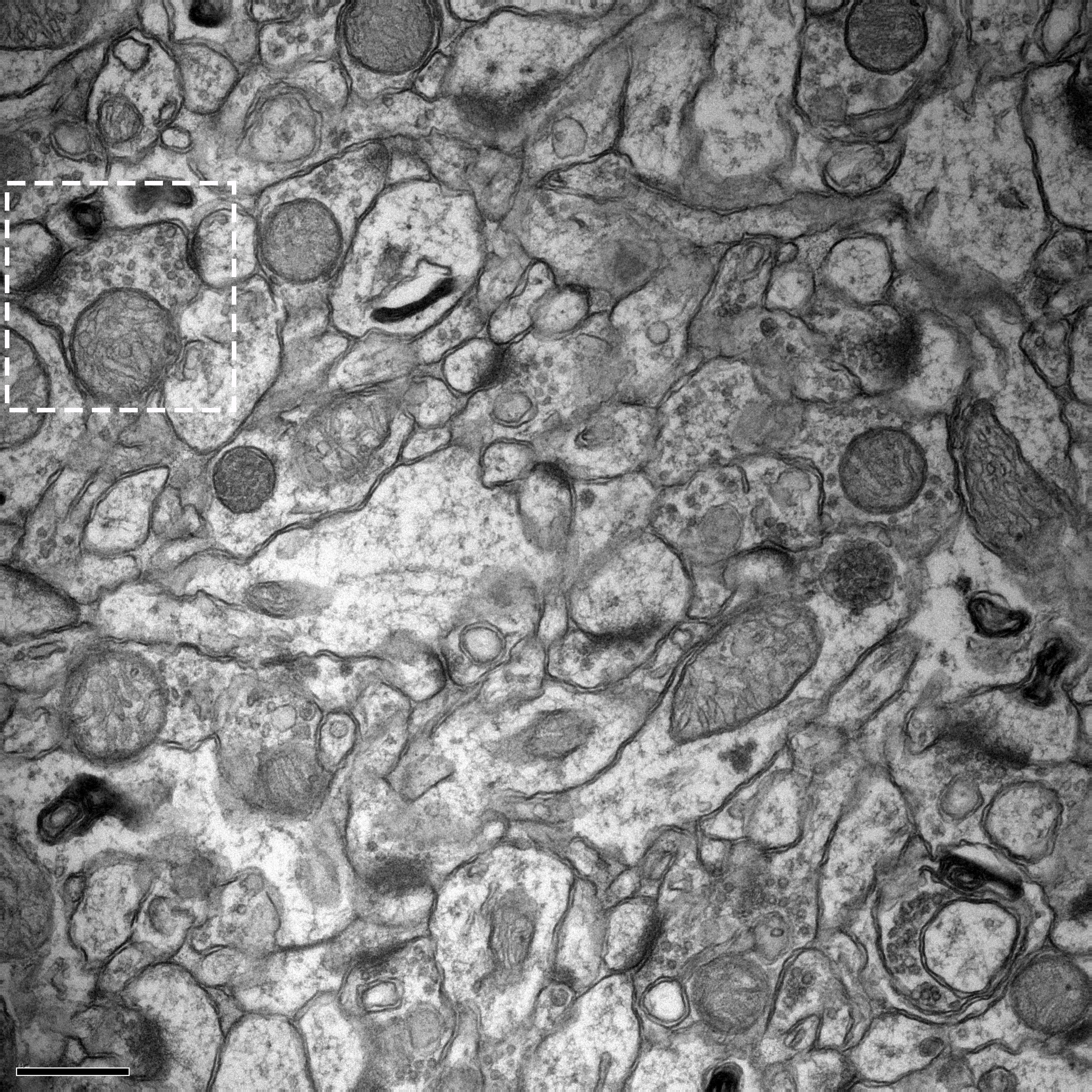

Supplement: Supplementary file 5 — Source data Fig. 3 [file 44318_2024_252_MOESM5_ESM.zip › Figure 3/3D/Figure3D_WT_s.r annotated.png]

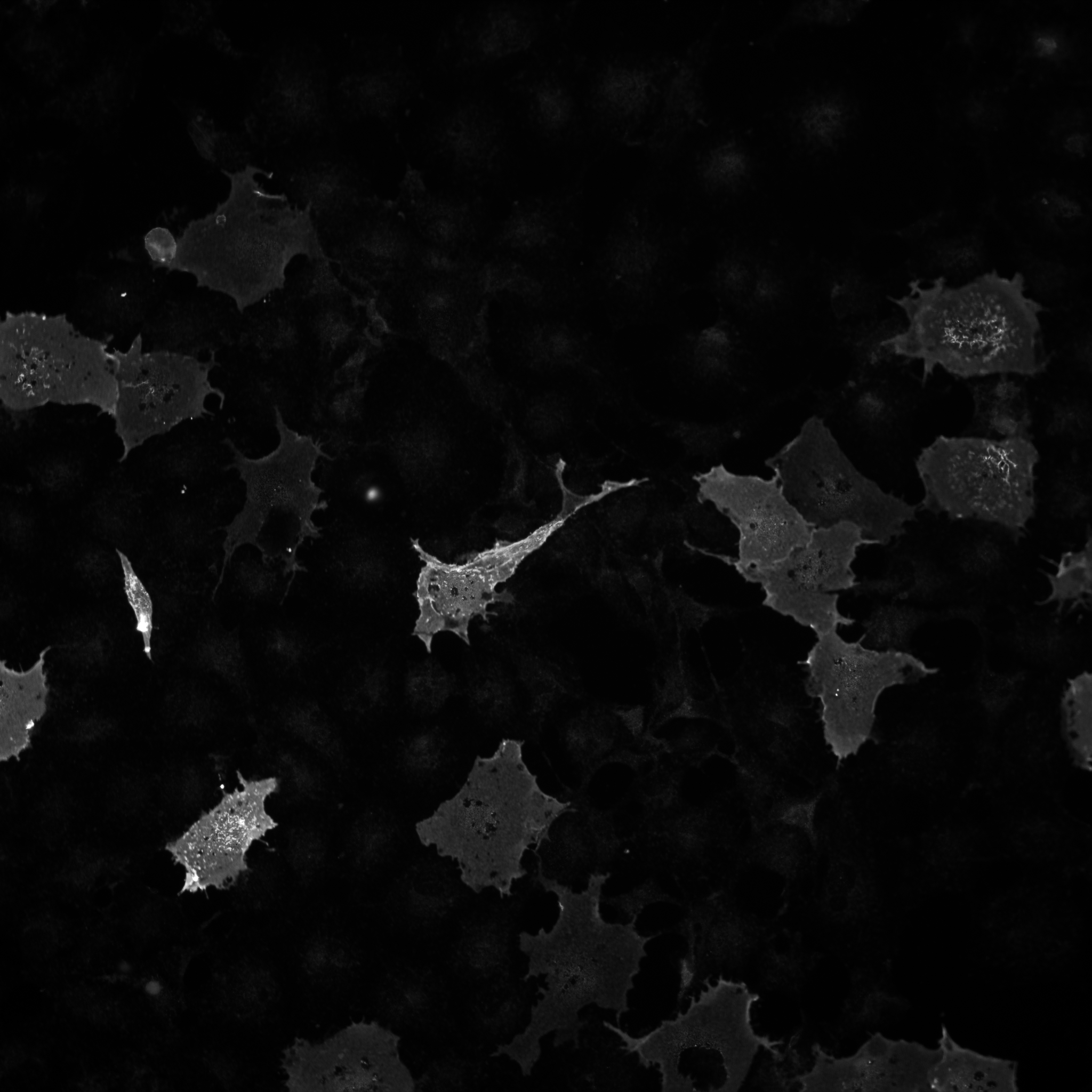

Supplement: Supplementary file 9 — Appendix. Fig. S1-10. [file 44318_2024_252_MOESM9_ESM.zip › Appendix. Fig. S1-10/Appendix. Fig. S1/S1 A/CD4 HA.tif]

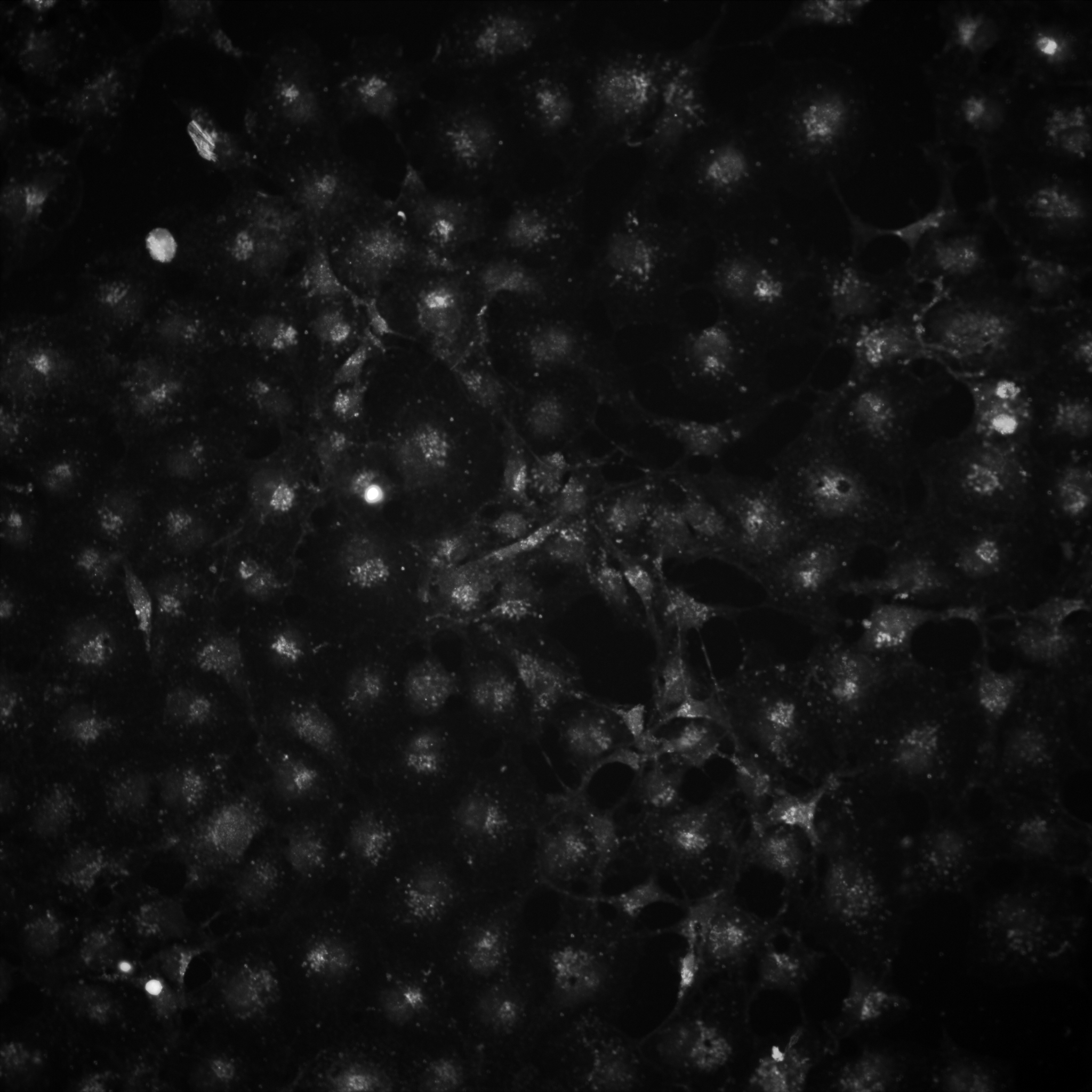

Supplement: Supplementary file 9 — Appendix. Fig. S1-10. [file 44318_2024_252_MOESM9_ESM.zip › Appendix. Fig. S1-10/Appendix. Fig. S1/S1 A/CD4 myc.tif]

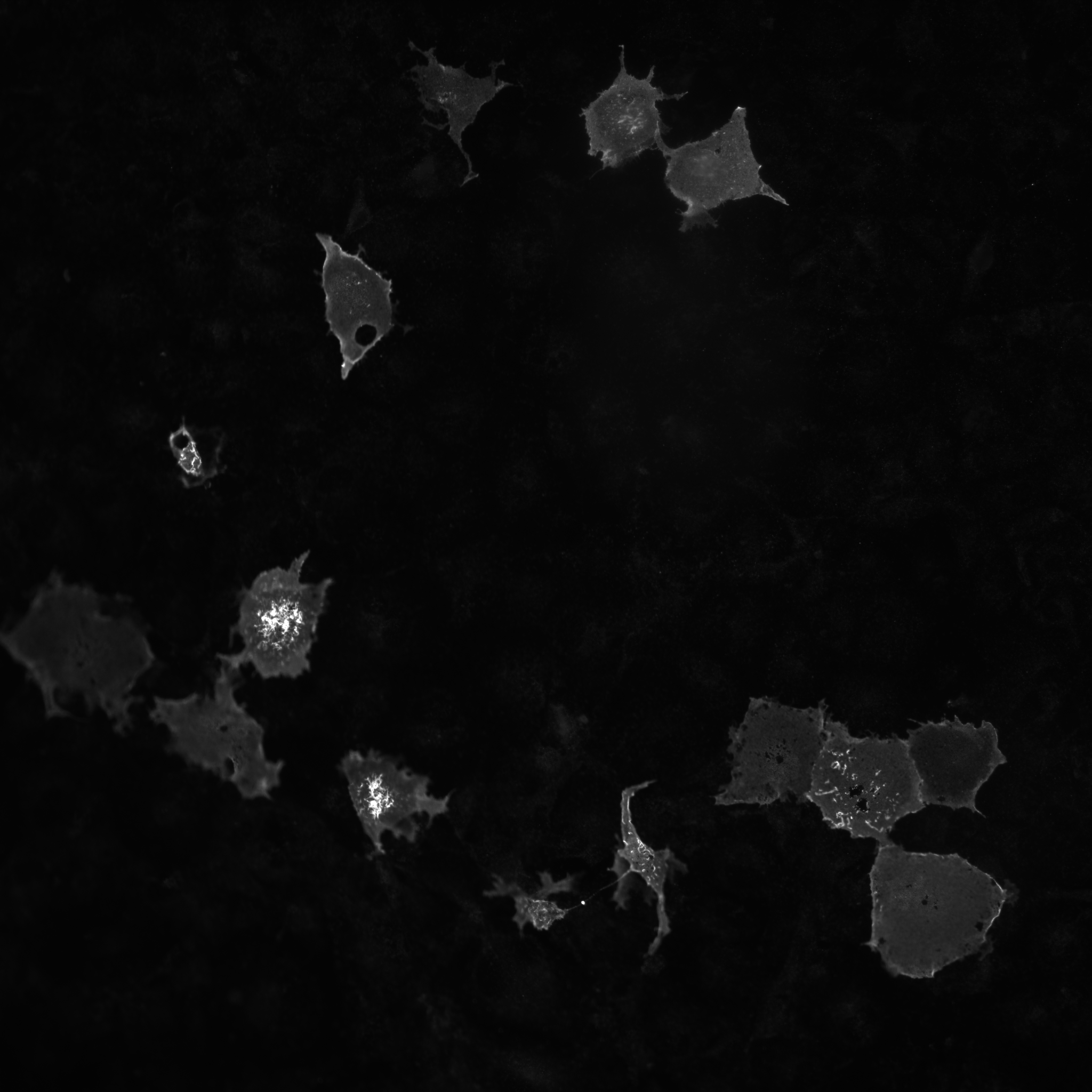

Supplement: Supplementary file 9 — Appendix. Fig. S1-10. [file 44318_2024_252_MOESM9_ESM.zip › Appendix. Fig. S1-10/Appendix. Fig. S1/S1 A/TrkC D240A D242A HA.tif]

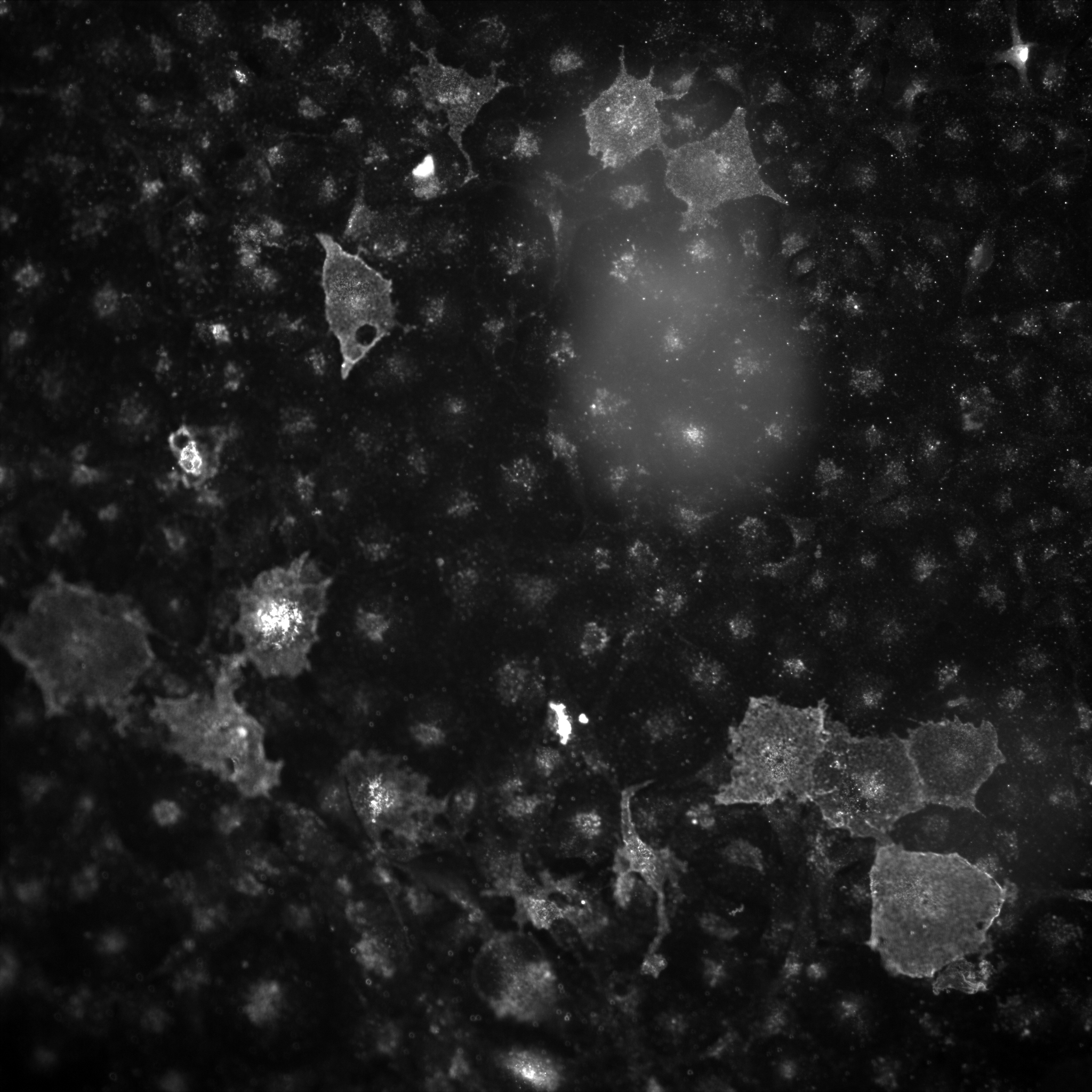

Supplement: Supplementary file 9 — Appendix. Fig. S1-10. [file 44318_2024_252_MOESM9_ESM.zip › Appendix. Fig. S1-10/Appendix. Fig. S1/S1 A/TrkC D240A D242A myc.tif]

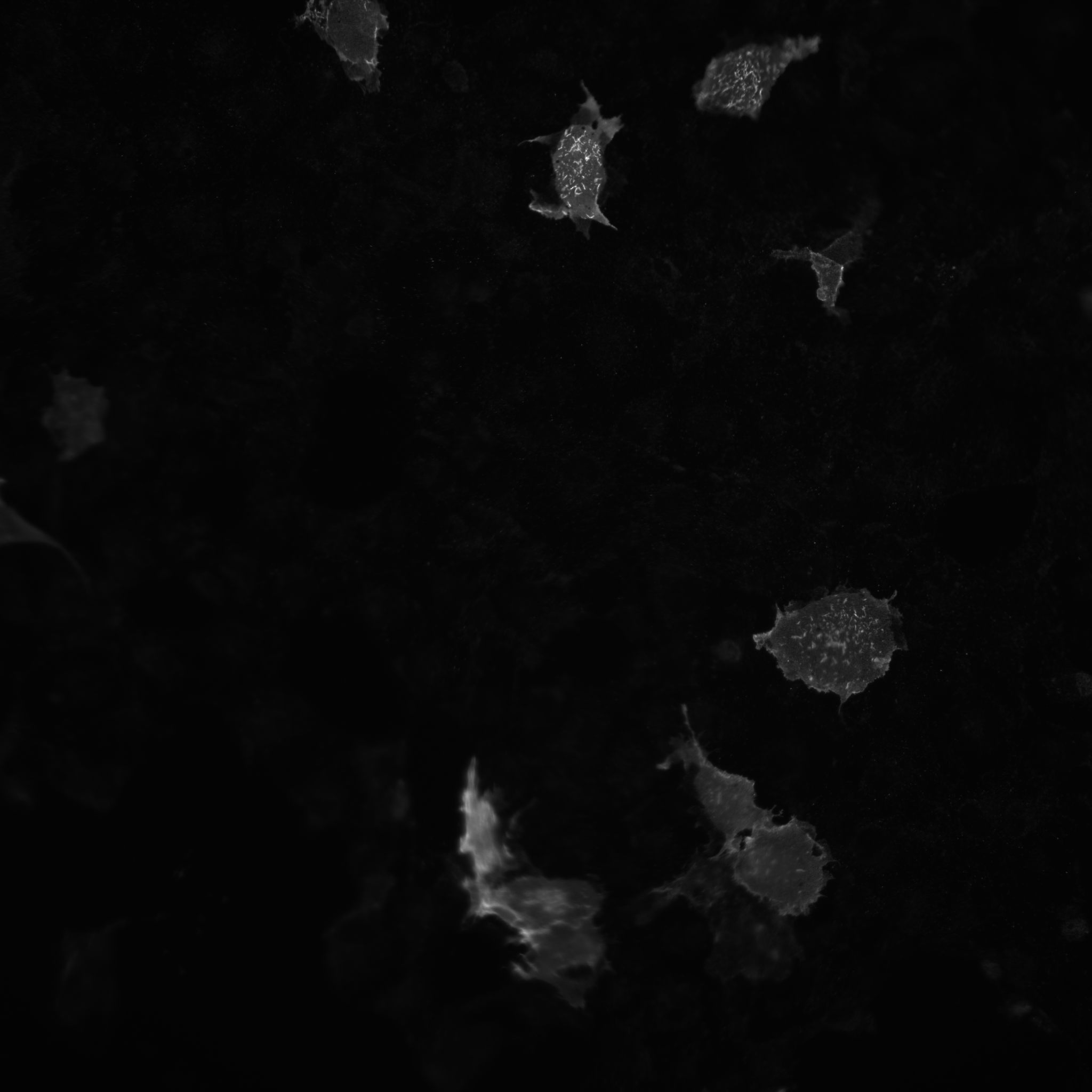

Supplement: Supplementary file 9 — Appendix. Fig. S1-10. [file 44318_2024_252_MOESM9_ESM.zip › Appendix. Fig. S1-10/Appendix. Fig. S1/S1 A/TrkC N366A N369A HA.tif]

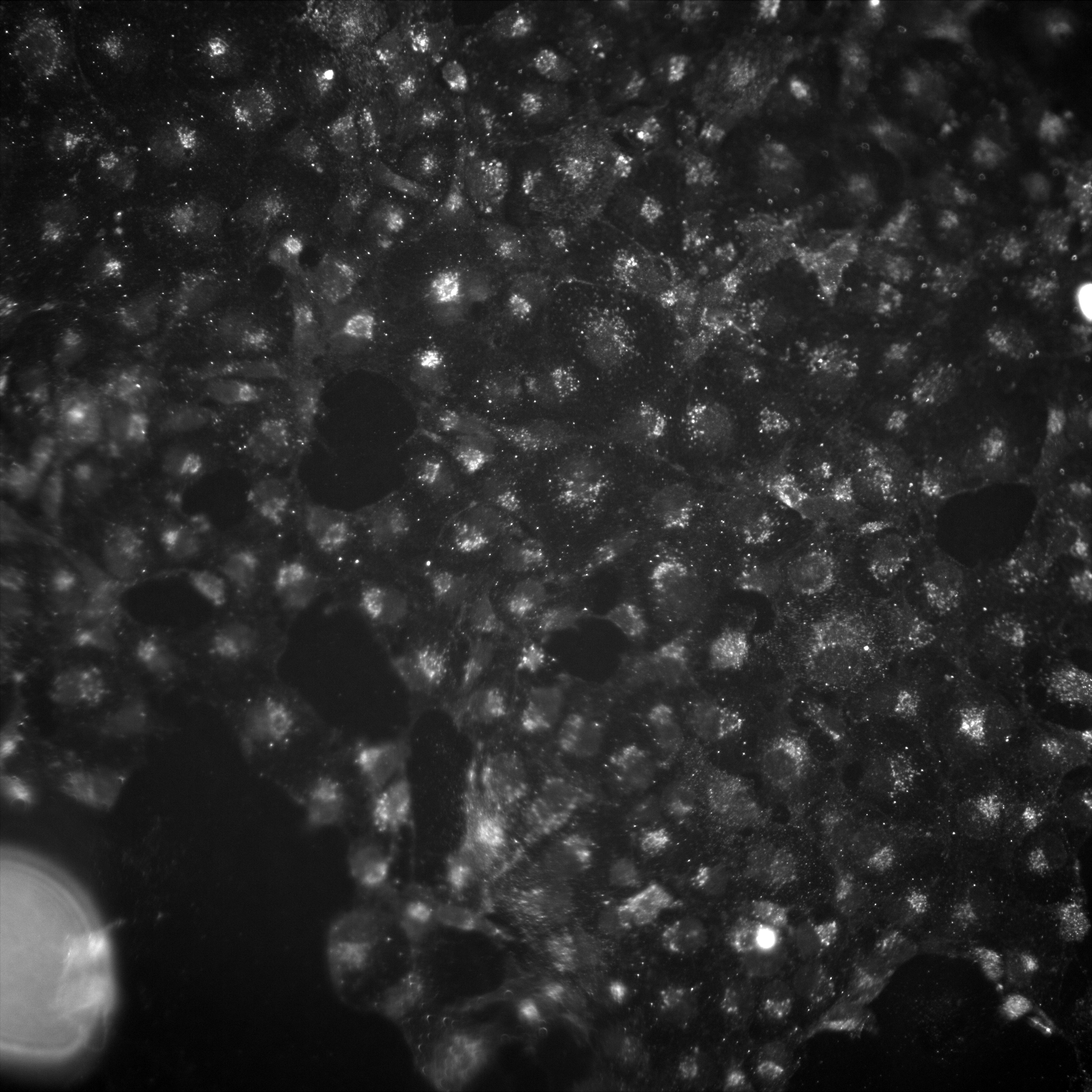

Supplement: Supplementary file 9 — Appendix. Fig. S1-10. [file 44318_2024_252_MOESM9_ESM.zip › Appendix. Fig. S1-10/Appendix. Fig. S1/S1 A/TrkC N366A N369A myc.tif]

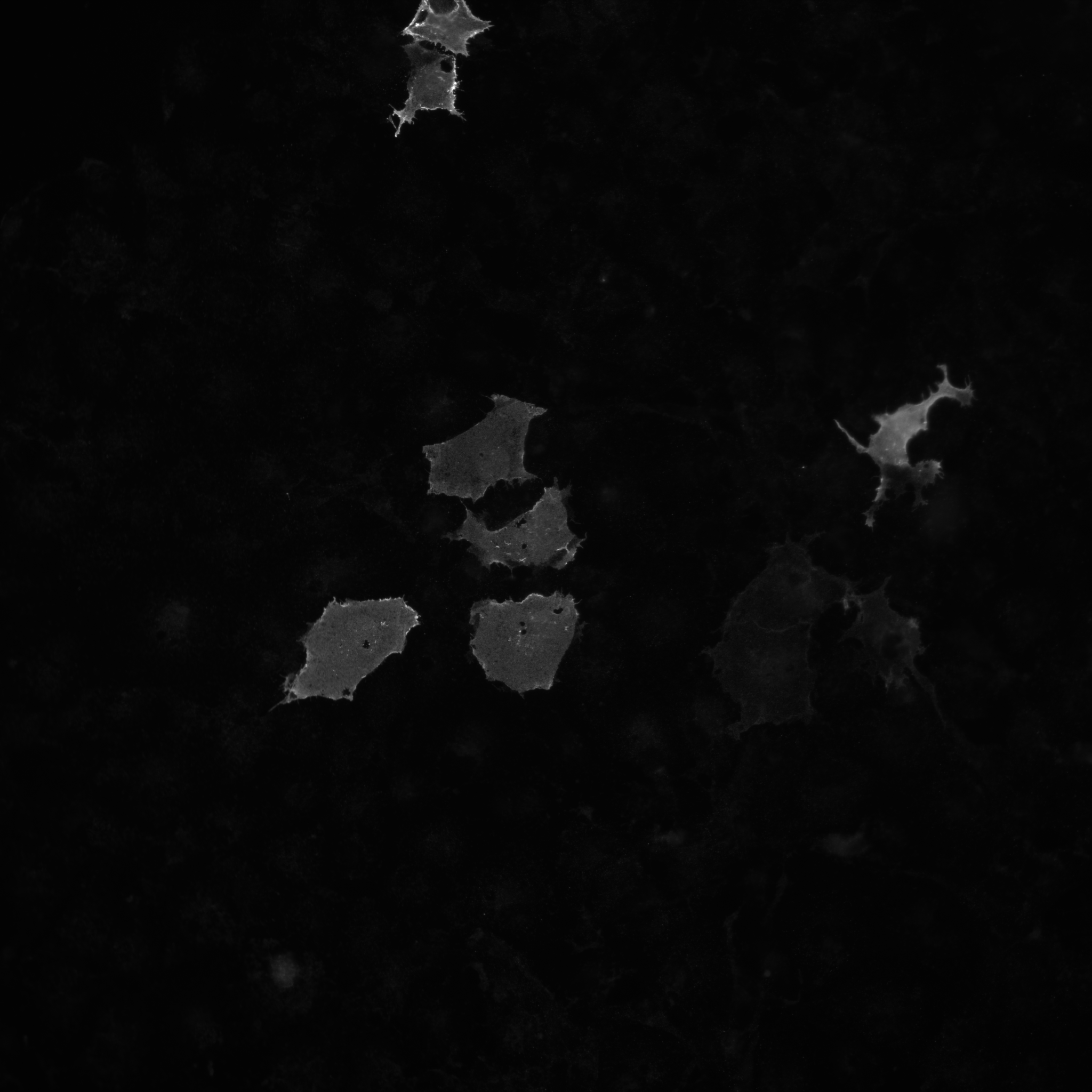

Supplement: Supplementary file 9 — Appendix. Fig. S1-10. [file 44318_2024_252_MOESM9_ESM.zip › Appendix. Fig. S1-10/Appendix. Fig. S1/S1 A/TrkC WT HA.tif]

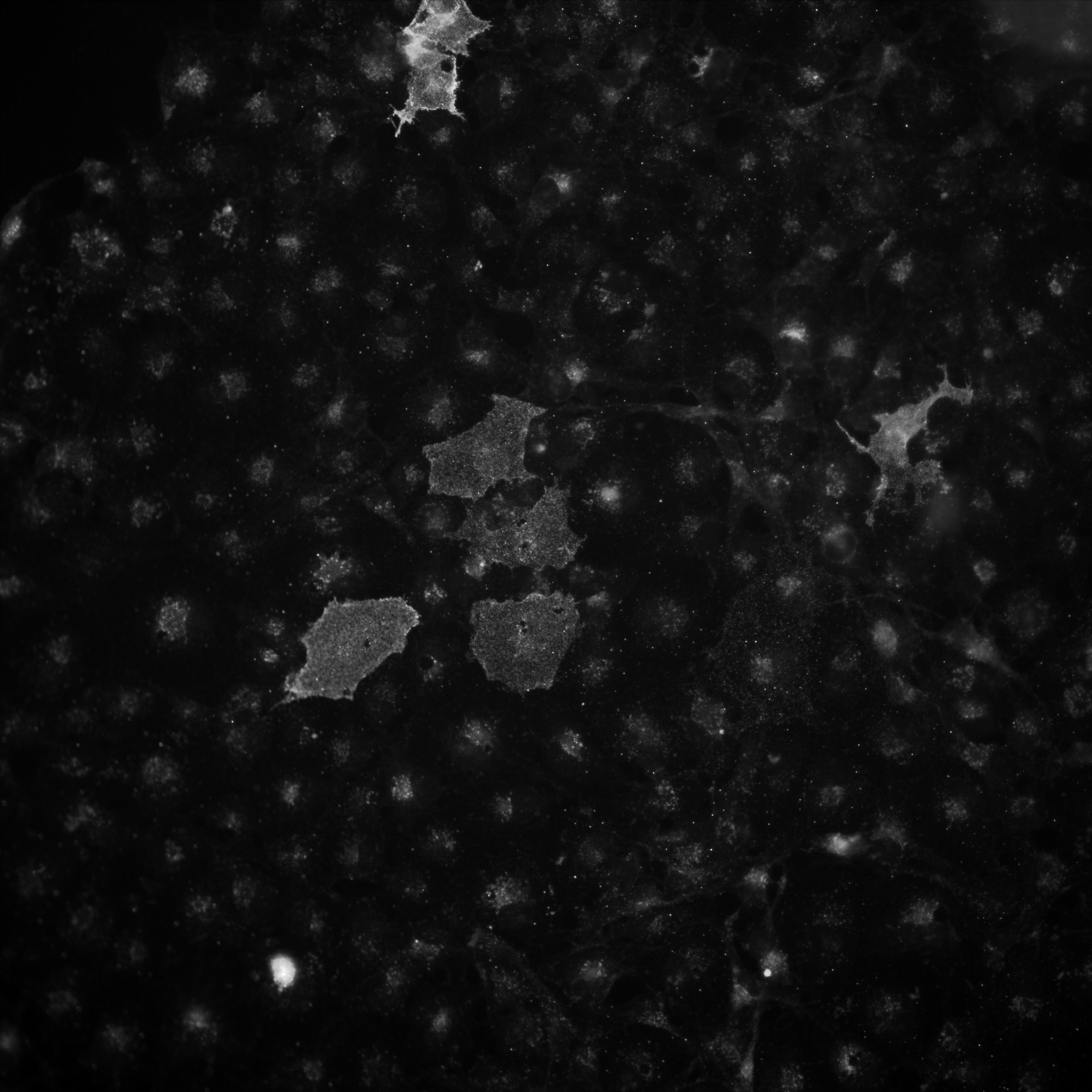

Supplement: Supplementary file 9 — Appendix. Fig. S1-10. [file 44318_2024_252_MOESM9_ESM.zip › Appendix. Fig. S1-10/Appendix. Fig. S1/S1 A/TrkC WT myc.tif]

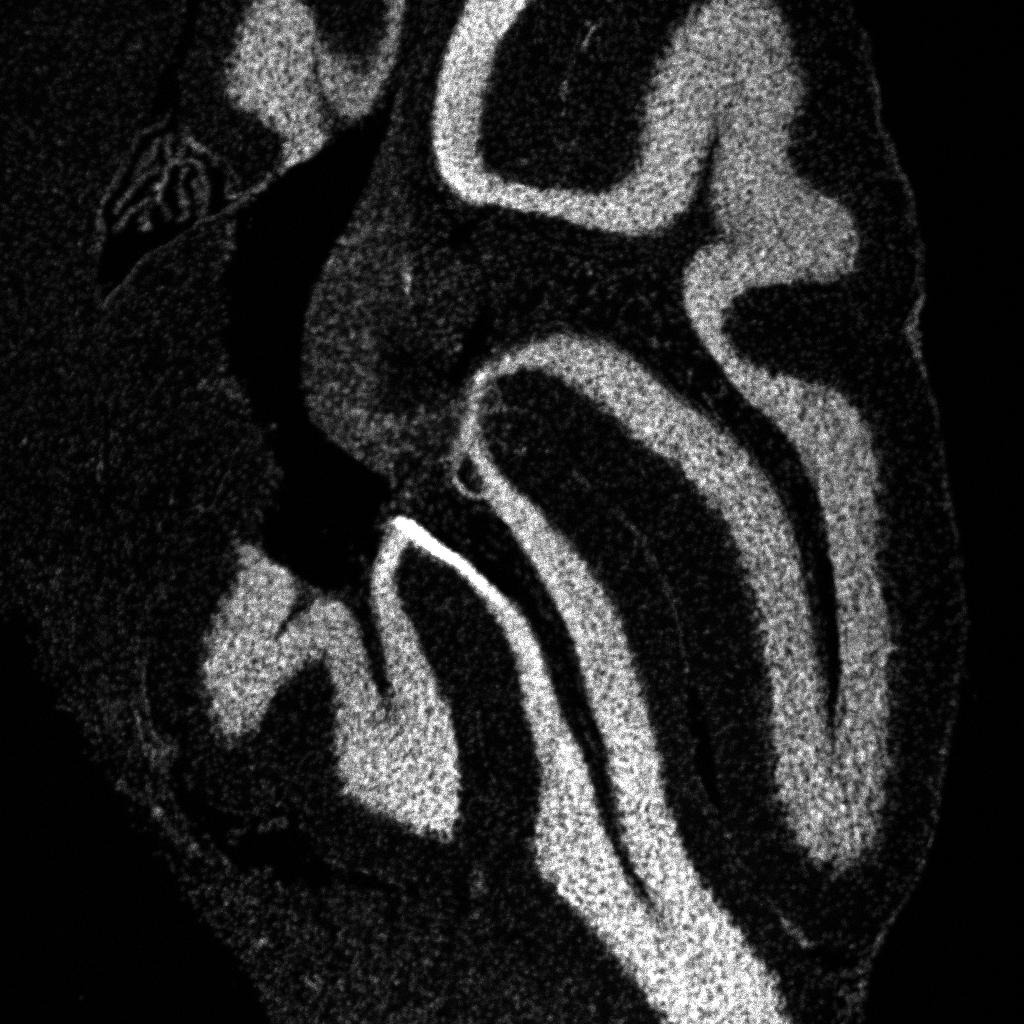

Supplement: Supplementary file 9 — Appendix. Fig. S1-10. [file 44318_2024_252_MOESM9_ESM.zip › Appendix. Fig. S1-10/Appendix. Fig. S2/S2 A/sup_fig_2A_KI_CB_DAPI.tif]

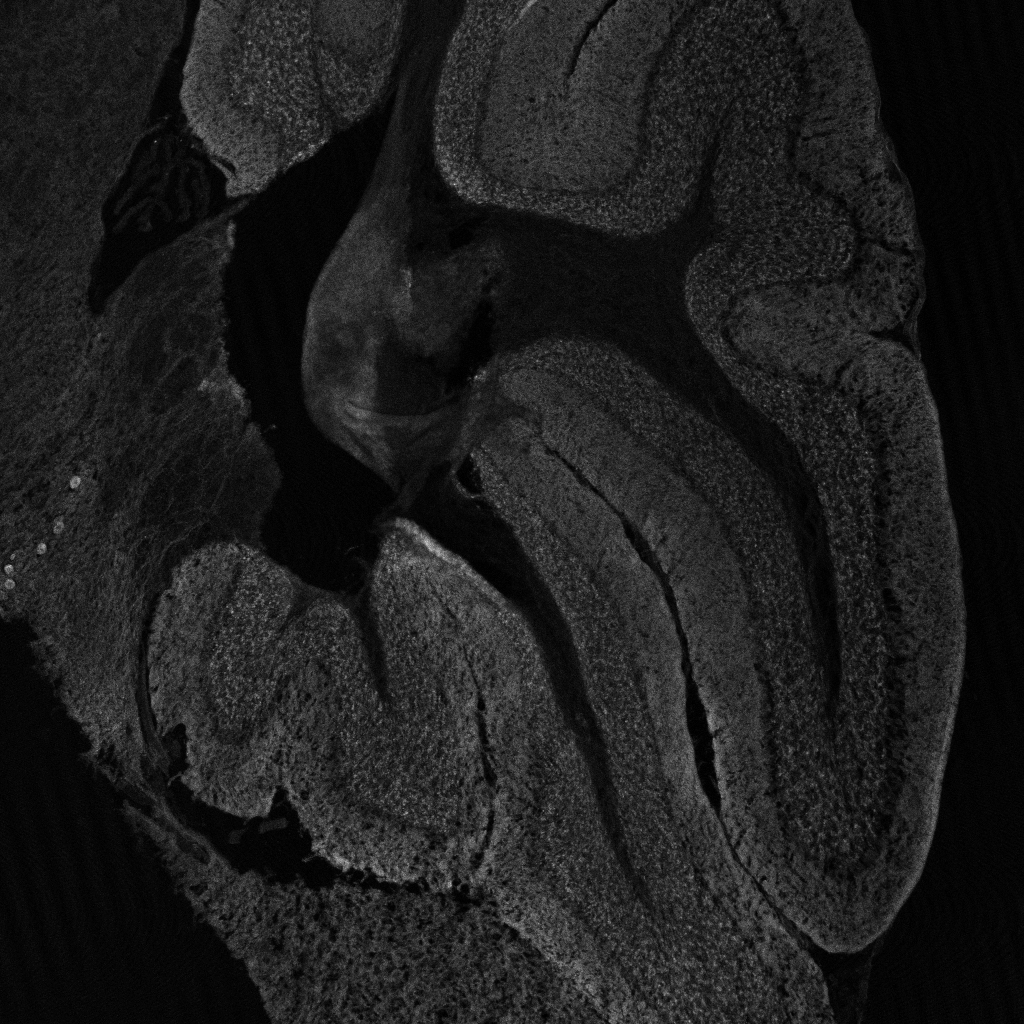

Supplement: Supplementary file 9 — Appendix. Fig. S1-10. [file 44318_2024_252_MOESM9_ESM.zip › Appendix. Fig. S1-10/Appendix. Fig. S2/S2 A/sup_fig_2A_KI_CB_TRKC.tif]

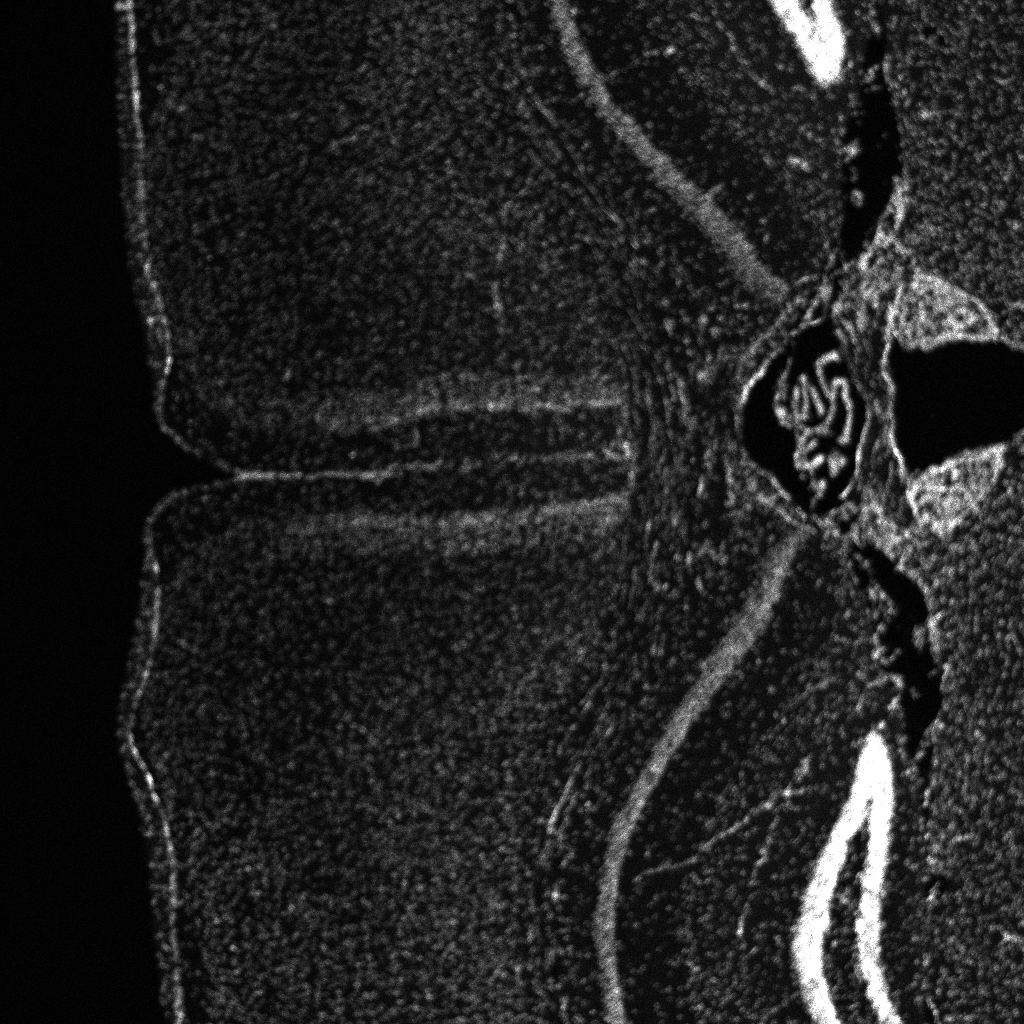

Supplement: Supplementary file 9 — Appendix. Fig. S1-10. [file 44318_2024_252_MOESM9_ESM.zip › Appendix. Fig. S1-10/Appendix. Fig. S2/S2 A/sup_fig_2A_KI_CX_DAPI.tif]

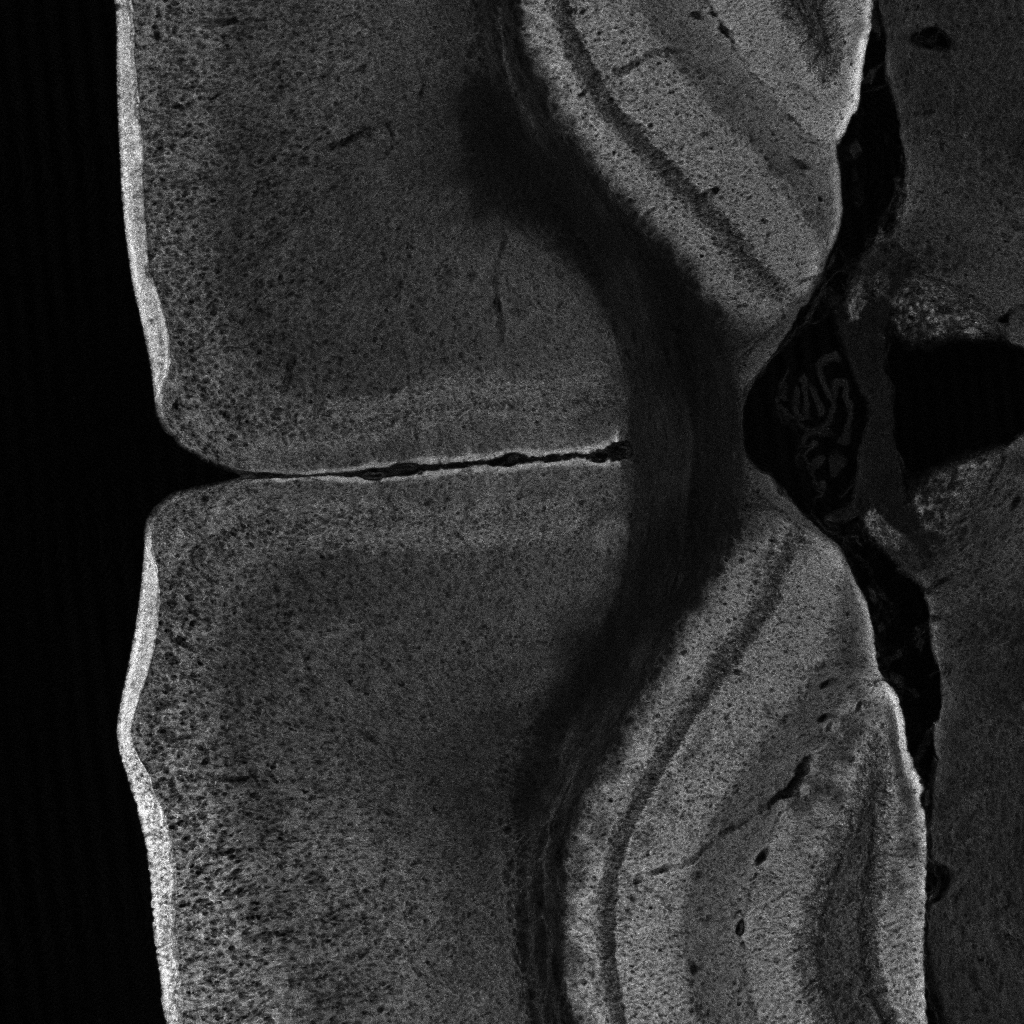

Supplement: Supplementary file 9 — Appendix. Fig. S1-10. [file 44318_2024_252_MOESM9_ESM.zip › Appendix. Fig. S1-10/Appendix. Fig. S2/S2 A/sup_fig_2A_KI_CX_TRKC.tif]

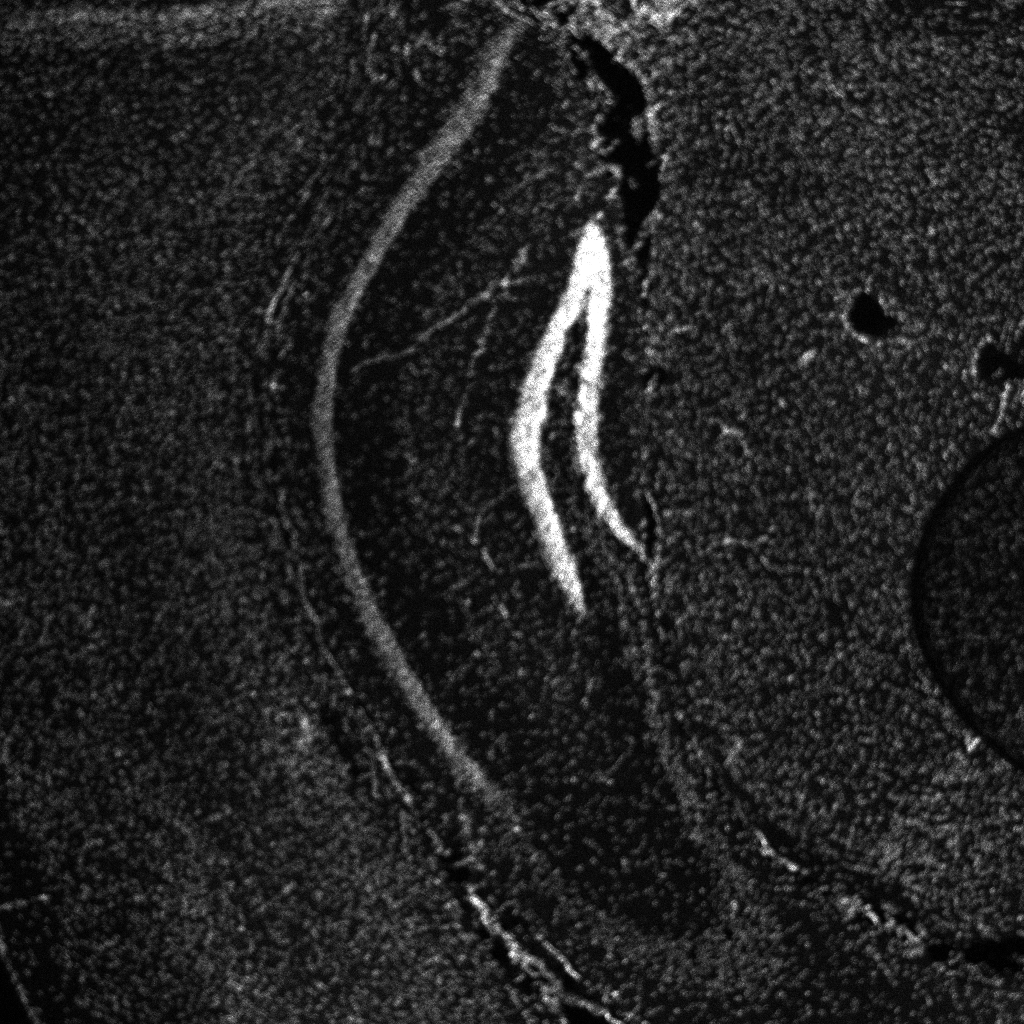

Supplement: Supplementary file 9 — Appendix. Fig. S1-10. [file 44318_2024_252_MOESM9_ESM.zip › Appendix. Fig. S1-10/Appendix. Fig. S2/S2 A/sup_fig_2A_KI_Hippo_DAPI.tif]

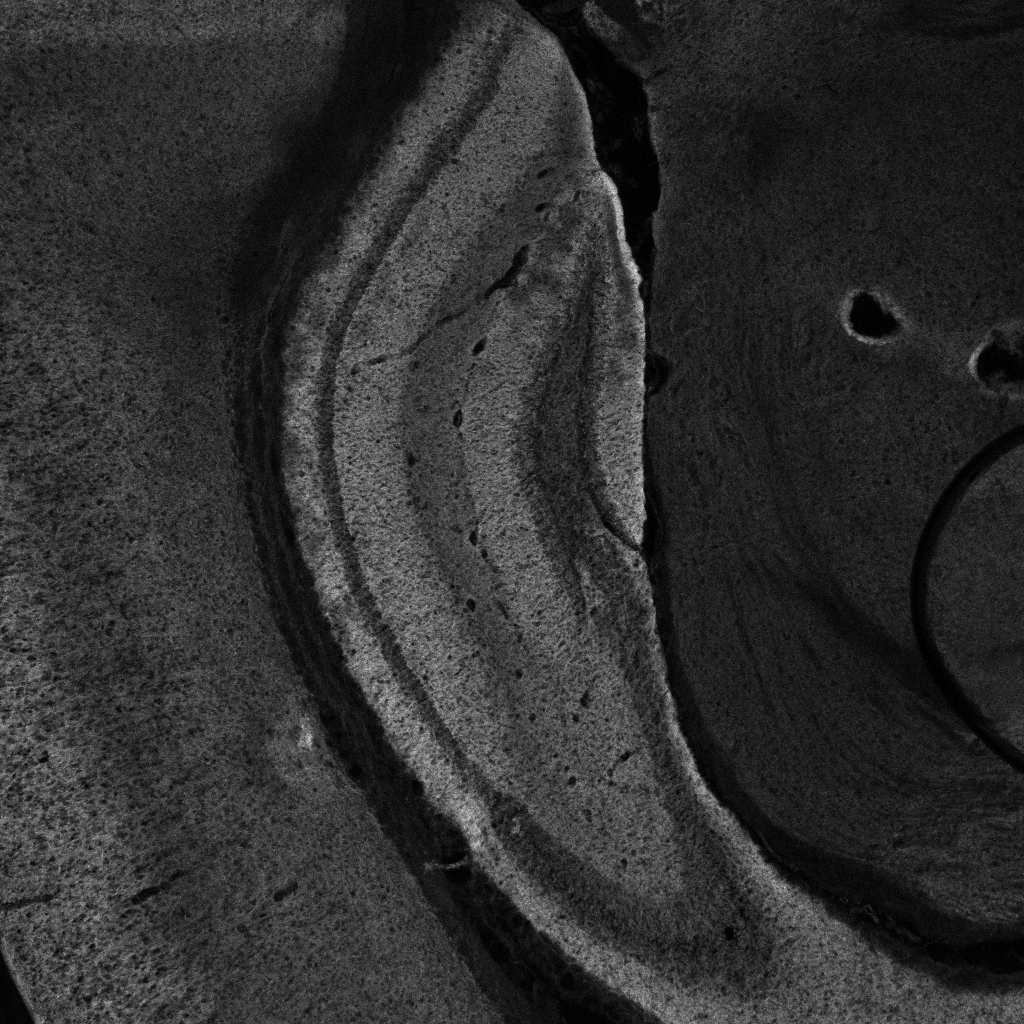

Supplement: Supplementary file 9 — Appendix. Fig. S1-10. [file 44318_2024_252_MOESM9_ESM.zip › Appendix. Fig. S1-10/Appendix. Fig. S2/S2 A/sup_fig_2A_KI_Hippo_TRKC.tif]

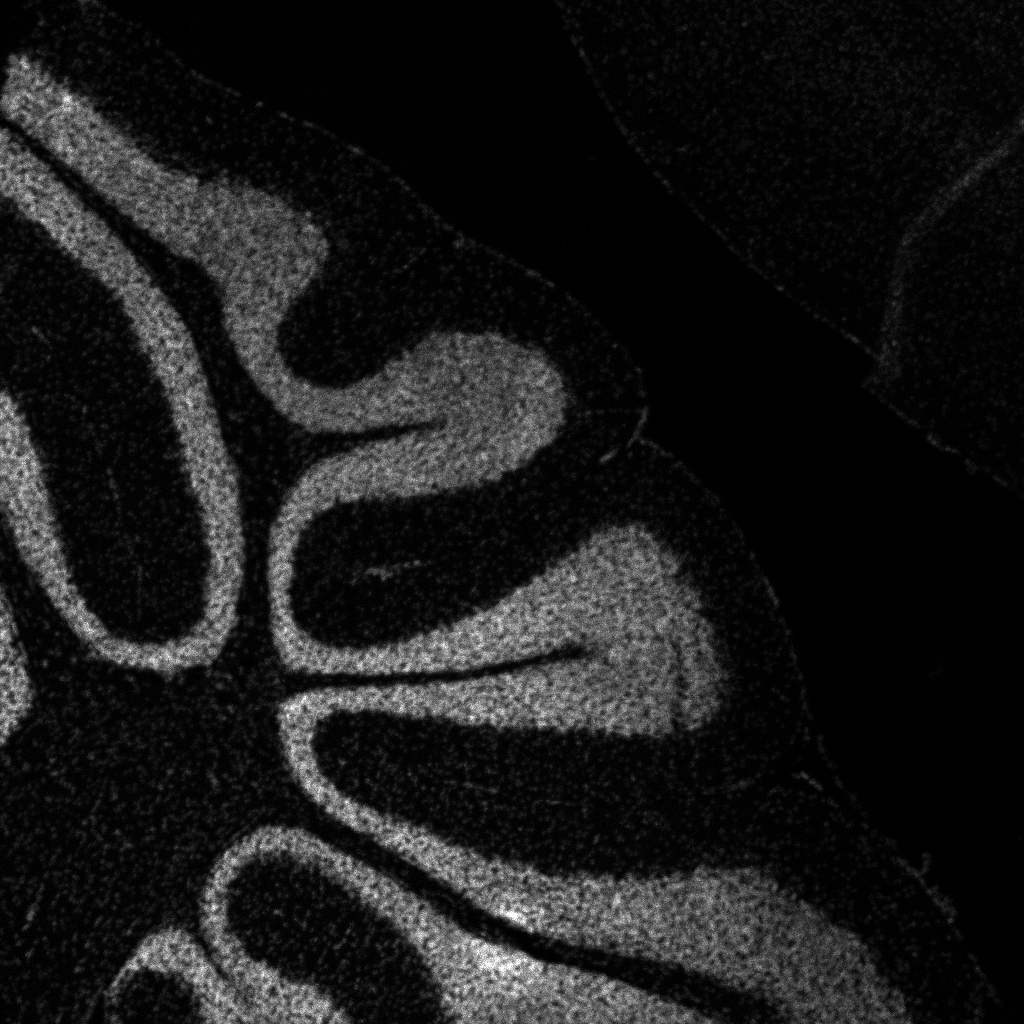

Supplement: Supplementary file 9 — Appendix. Fig. S1-10. [file 44318_2024_252_MOESM9_ESM.zip › Appendix. Fig. S1-10/Appendix. Fig. S2/S2 A/sup_fig_2A_WT_CB_DAPI.tif]

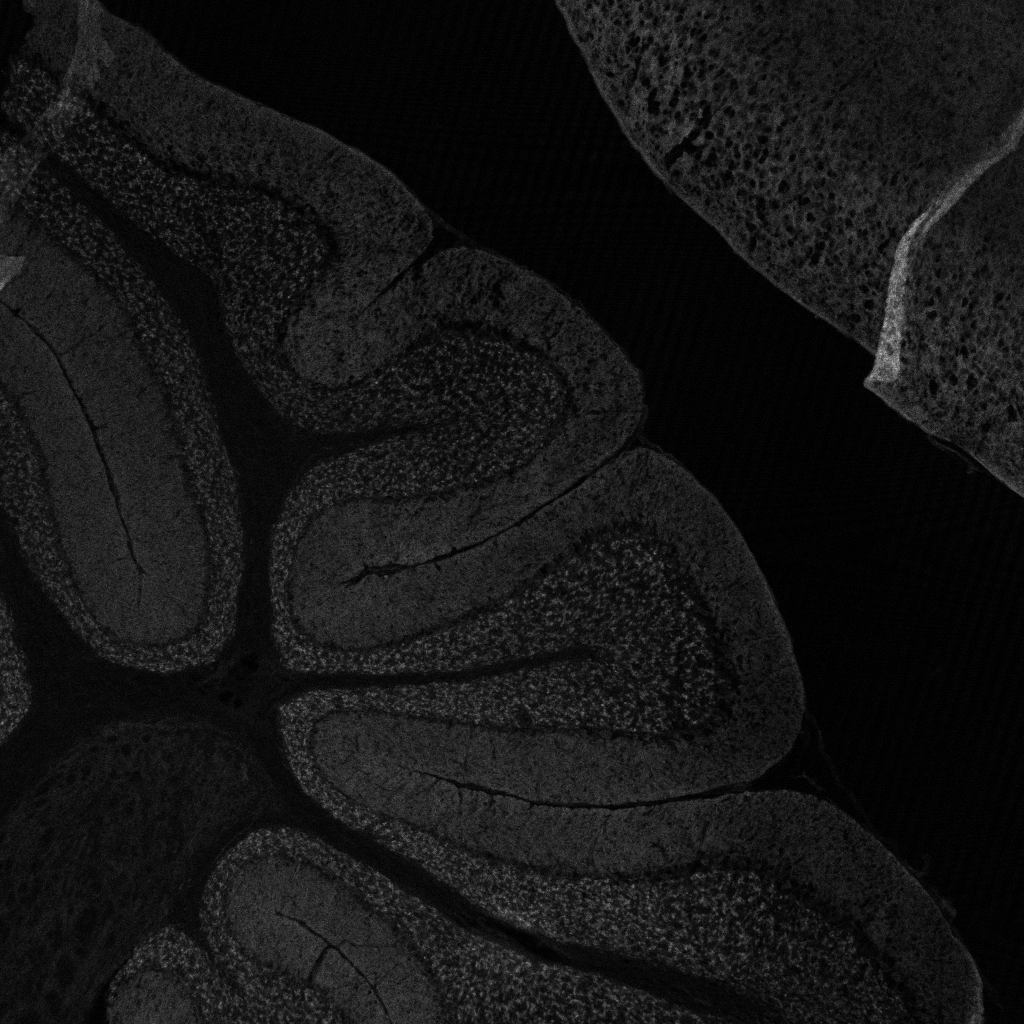

Supplement: Supplementary file 9 — Appendix. Fig. S1-10. [file 44318_2024_252_MOESM9_ESM.zip › Appendix. Fig. S1-10/Appendix. Fig. S2/S2 A/sup_fig_2A_WT_CB_TRKC.tif]

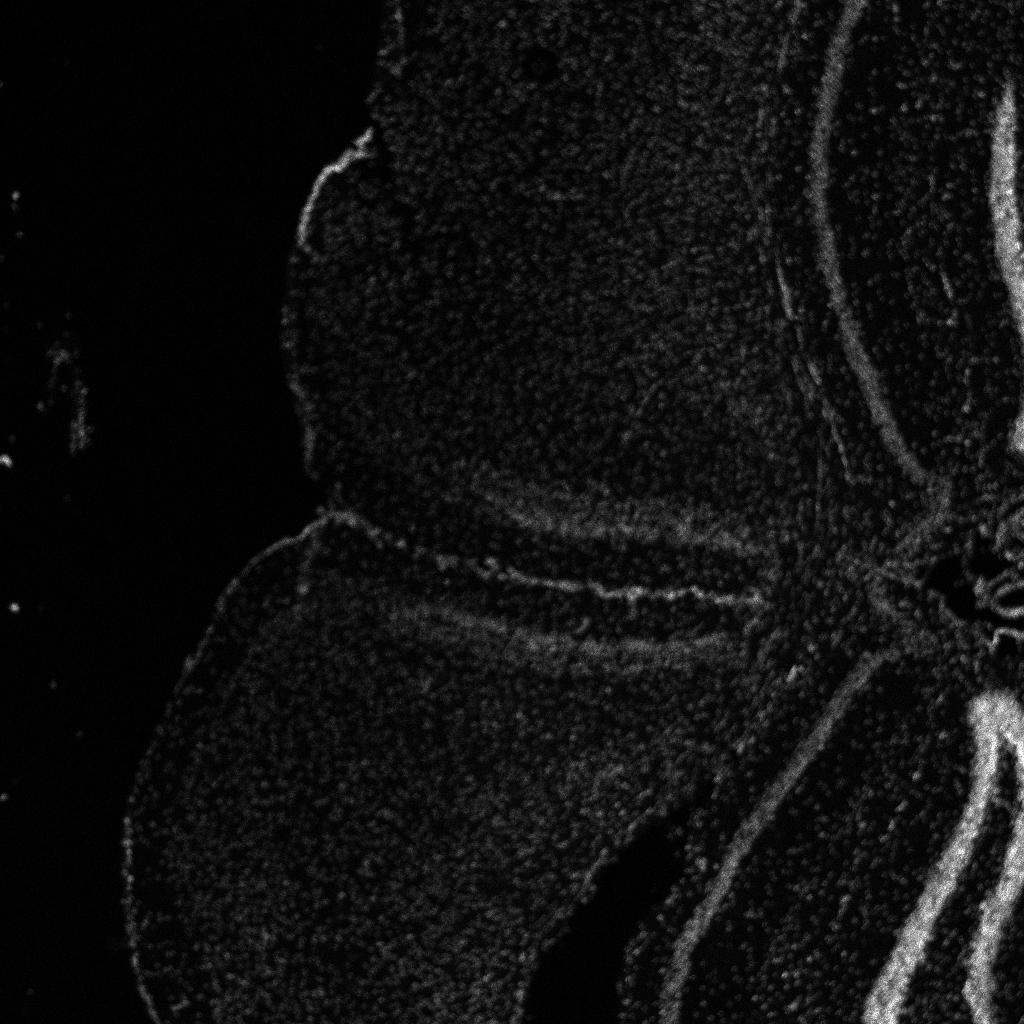

Supplement: Supplementary file 9 — Appendix. Fig. S1-10. [file 44318_2024_252_MOESM9_ESM.zip › Appendix. Fig. S1-10/Appendix. Fig. S2/S2 A/sup_fig_2A_WT_CX_DAPI.tif]

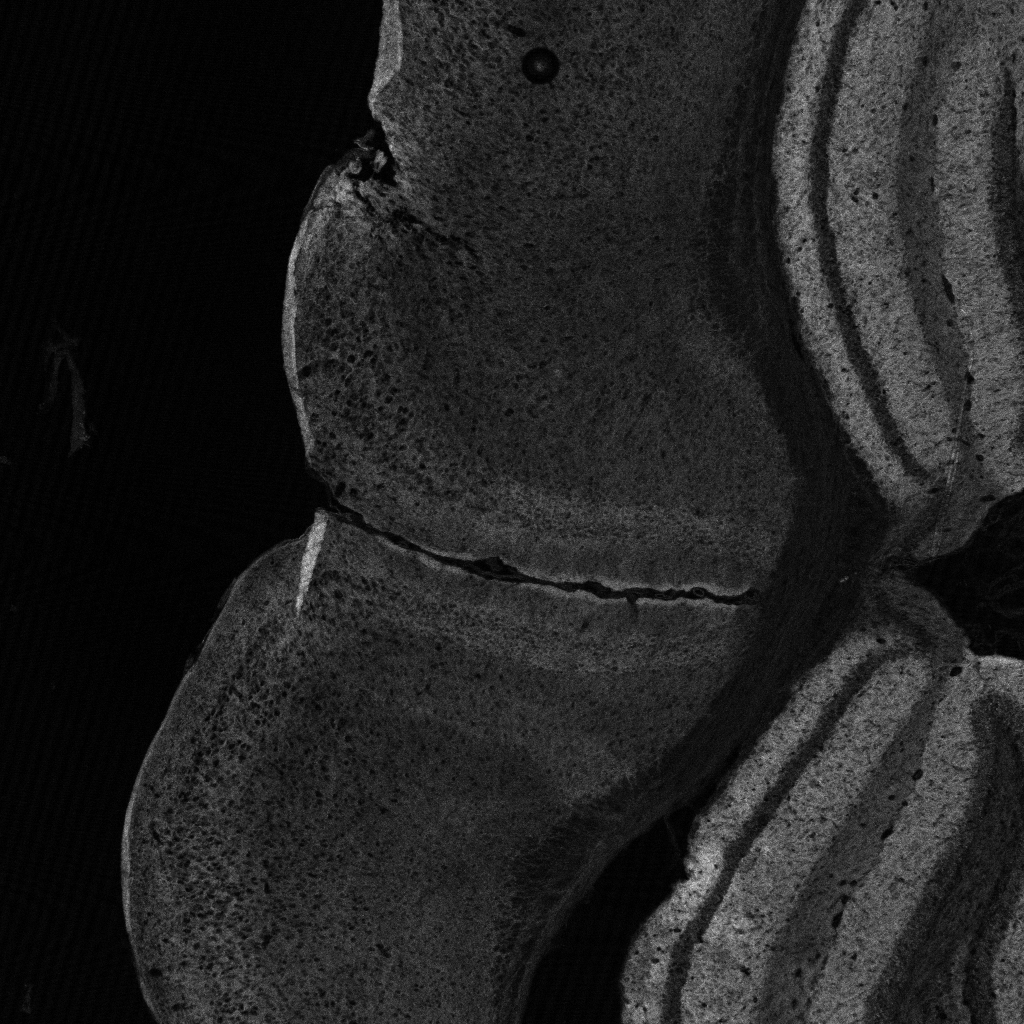

Supplement: Supplementary file 9 — Appendix. Fig. S1-10. [file 44318_2024_252_MOESM9_ESM.zip › Appendix. Fig. S1-10/Appendix. Fig. S2/S2 A/sup_fig_2A_WT_CX_TRKC.tif]

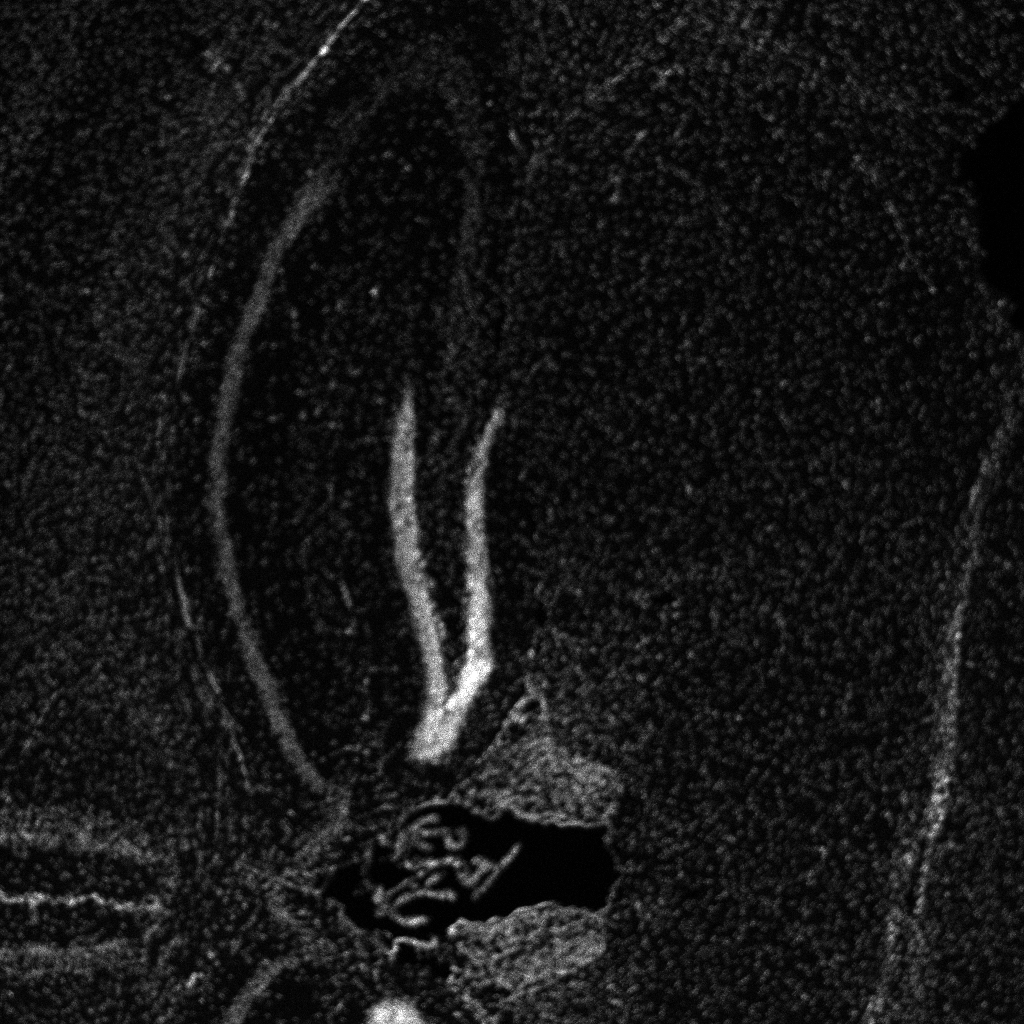

Supplement: Supplementary file 9 — Appendix. Fig. S1-10. [file 44318_2024_252_MOESM9_ESM.zip › Appendix. Fig. S1-10/Appendix. Fig. S2/S2 A/sup_fig_2A_WT_Hippo_DAPI.tif]

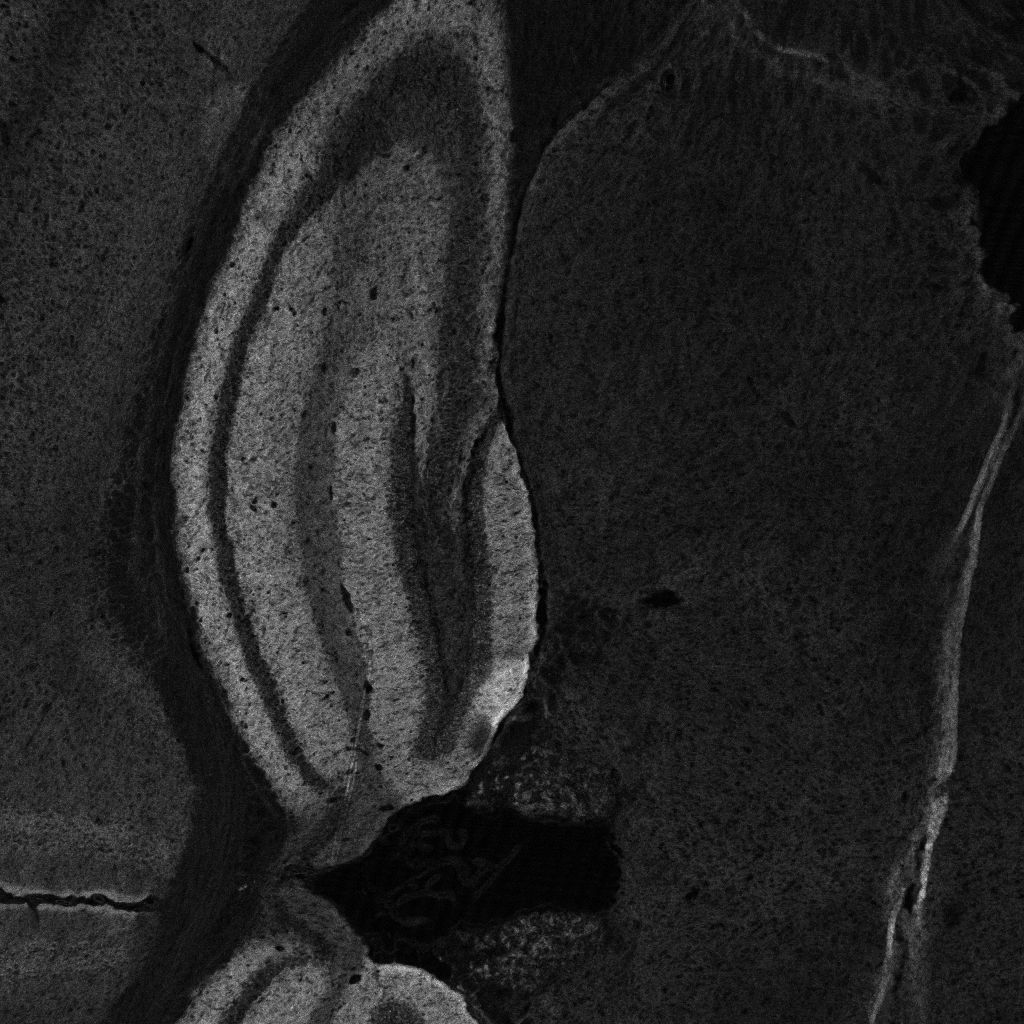

Supplement: Supplementary file 9 — Appendix. Fig. S1-10. [file 44318_2024_252_MOESM9_ESM.zip › Appendix. Fig. S1-10/Appendix. Fig. S2/S2 A/sup_fig_2A_WT_Hippo_TRKC.tif]

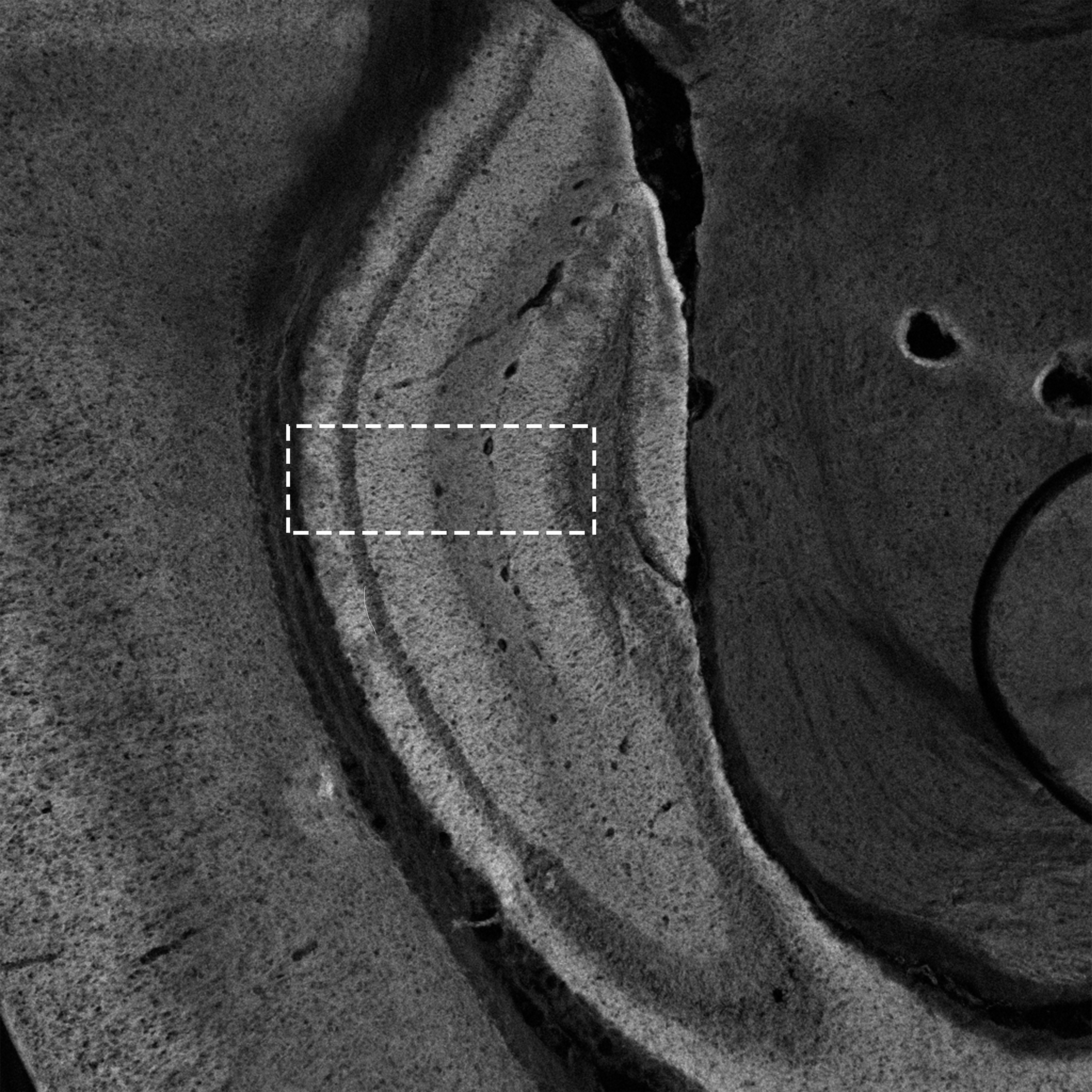

Supplement: Supplementary file 9 — Appendix. Fig. S1-10. [file 44318_2024_252_MOESM9_ESM.zip › Appendix. Fig. S1-10/Appendix. Fig. S2/S2 B/sup_fig_2B_KI annotated.png]

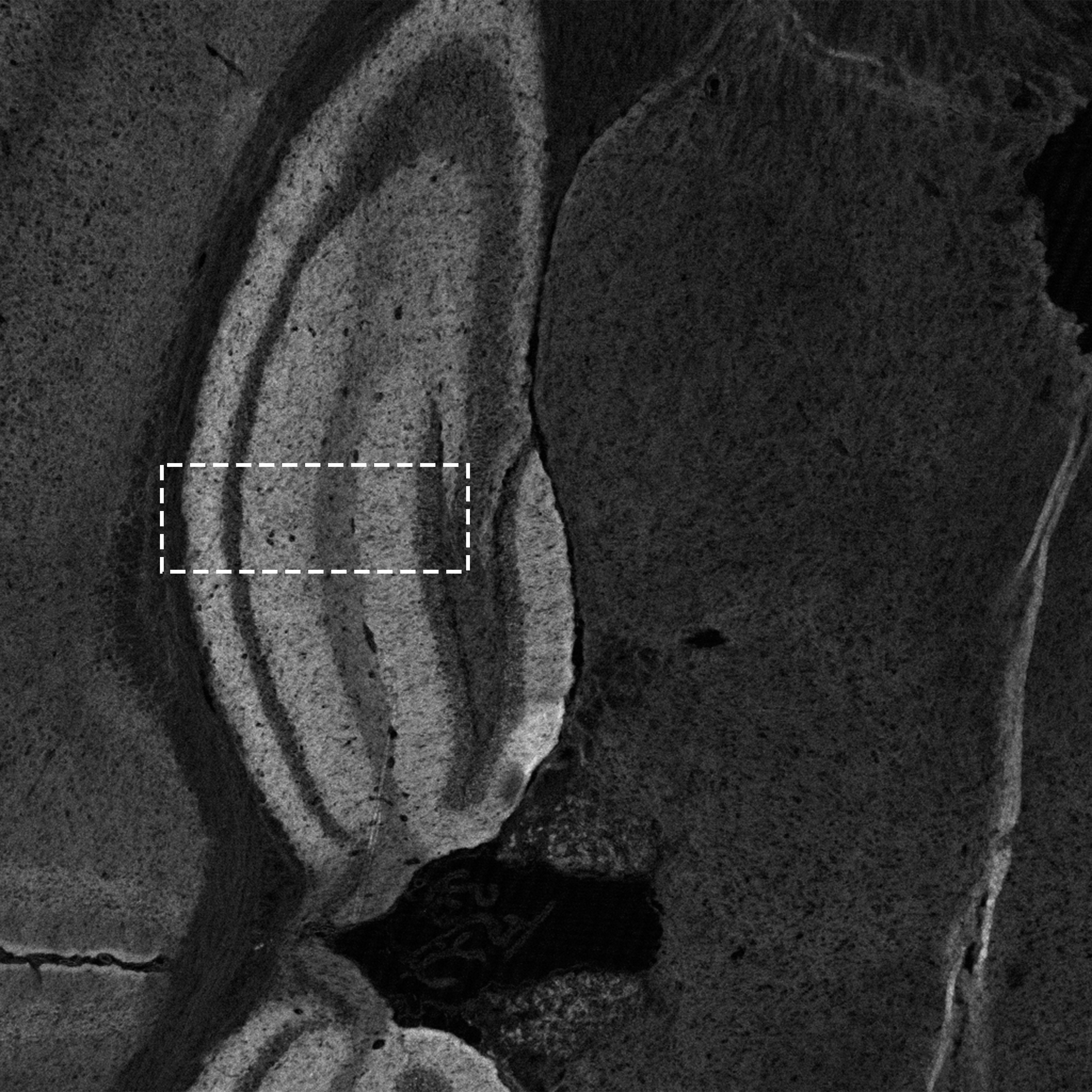

Supplement: Supplementary file 9 — Appendix. Fig. S1-10. [file 44318_2024_252_MOESM9_ESM.zip › Appendix. Fig. S1-10/Appendix. Fig. S2/S2 B/sup_fig_2B_WT annotated.png]

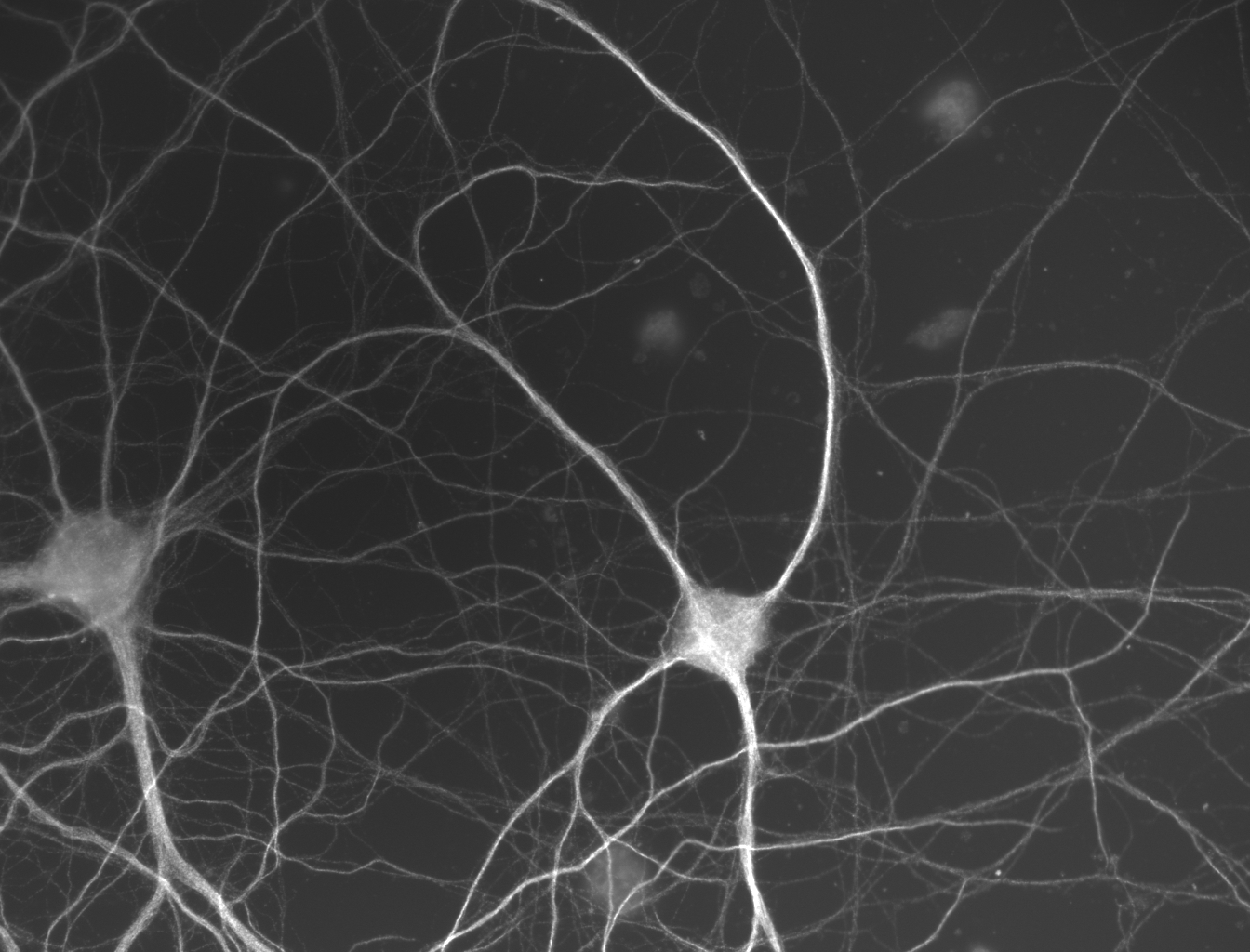

Supplement: Supplementary file 9 — Appendix. Fig. S1-10. [file 44318_2024_252_MOESM9_ESM.zip › Appendix. Fig. S1-10/Appendix. Fig. S4/S4 A/TrkC KI MAP2.tif]

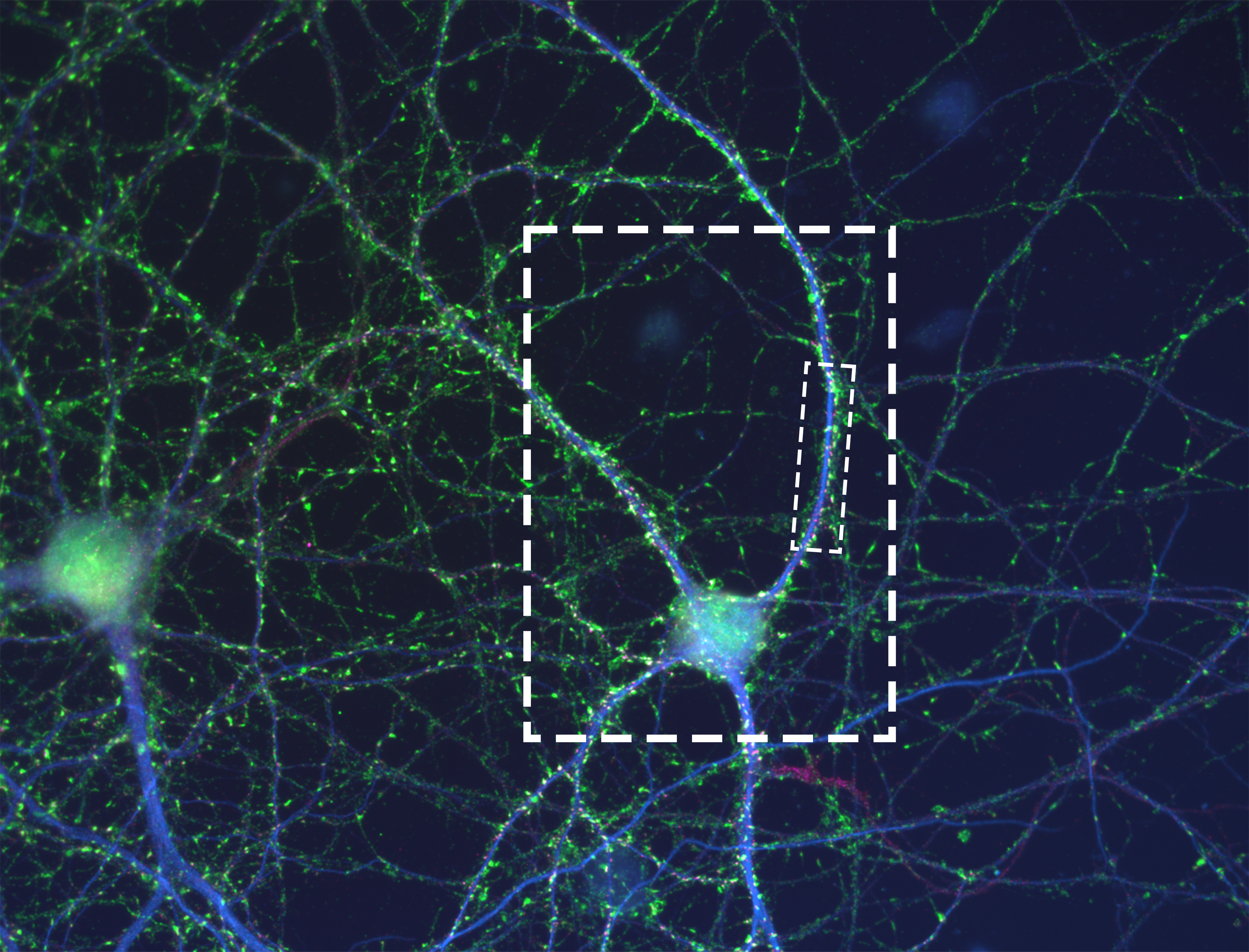

Supplement: Supplementary file 9 — Appendix. Fig. S1-10. [file 44318_2024_252_MOESM9_ESM.zip › Appendix. Fig. S1-10/Appendix. Fig. S4/S4 A/TrkC KI merged and annotated.png]

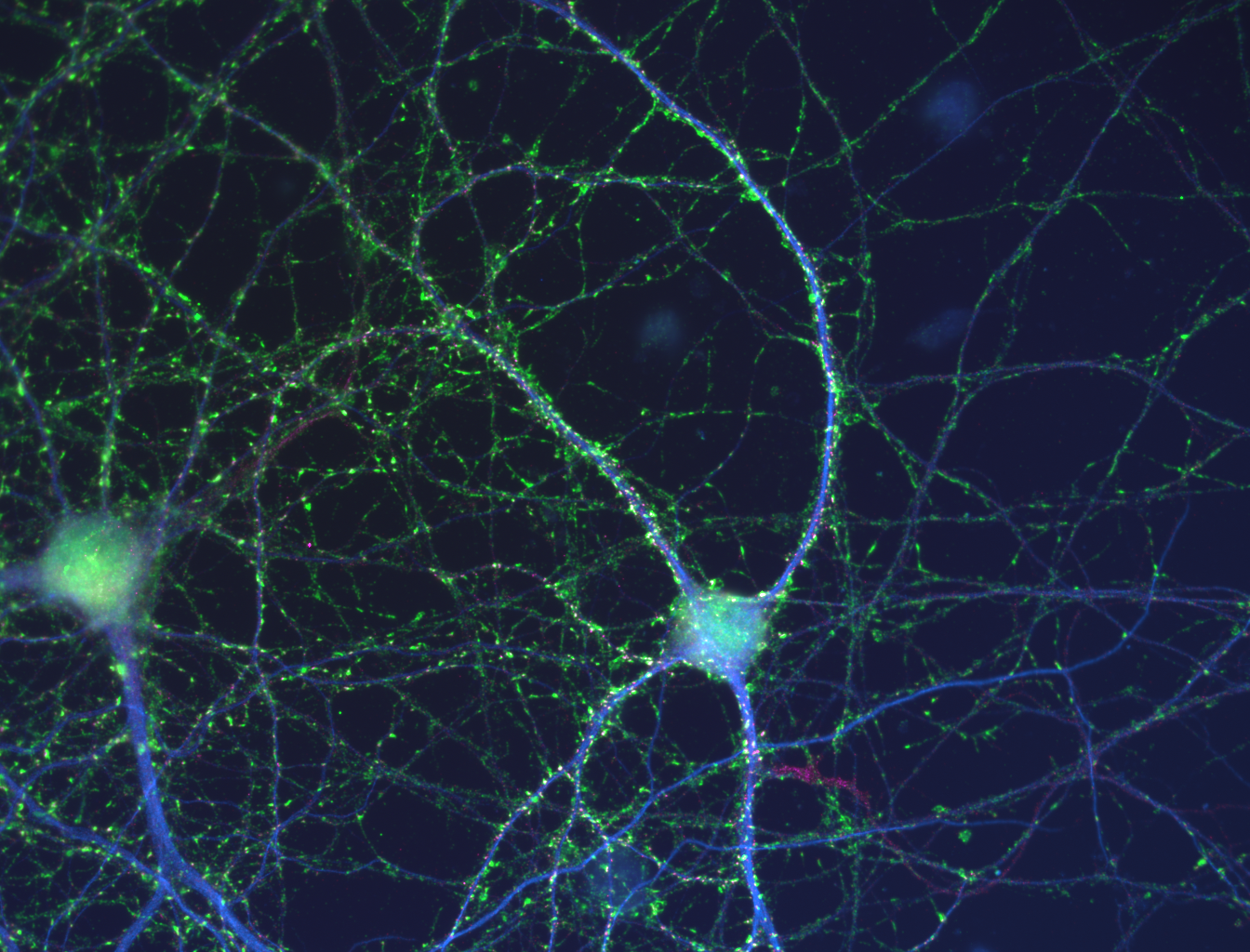

Supplement: Supplementary file 9 — Appendix. Fig. S1-10. [file 44318_2024_252_MOESM9_ESM.zip › Appendix. Fig. S1-10/Appendix. Fig. S4/S4 A/TrkC KI Merged.tif]

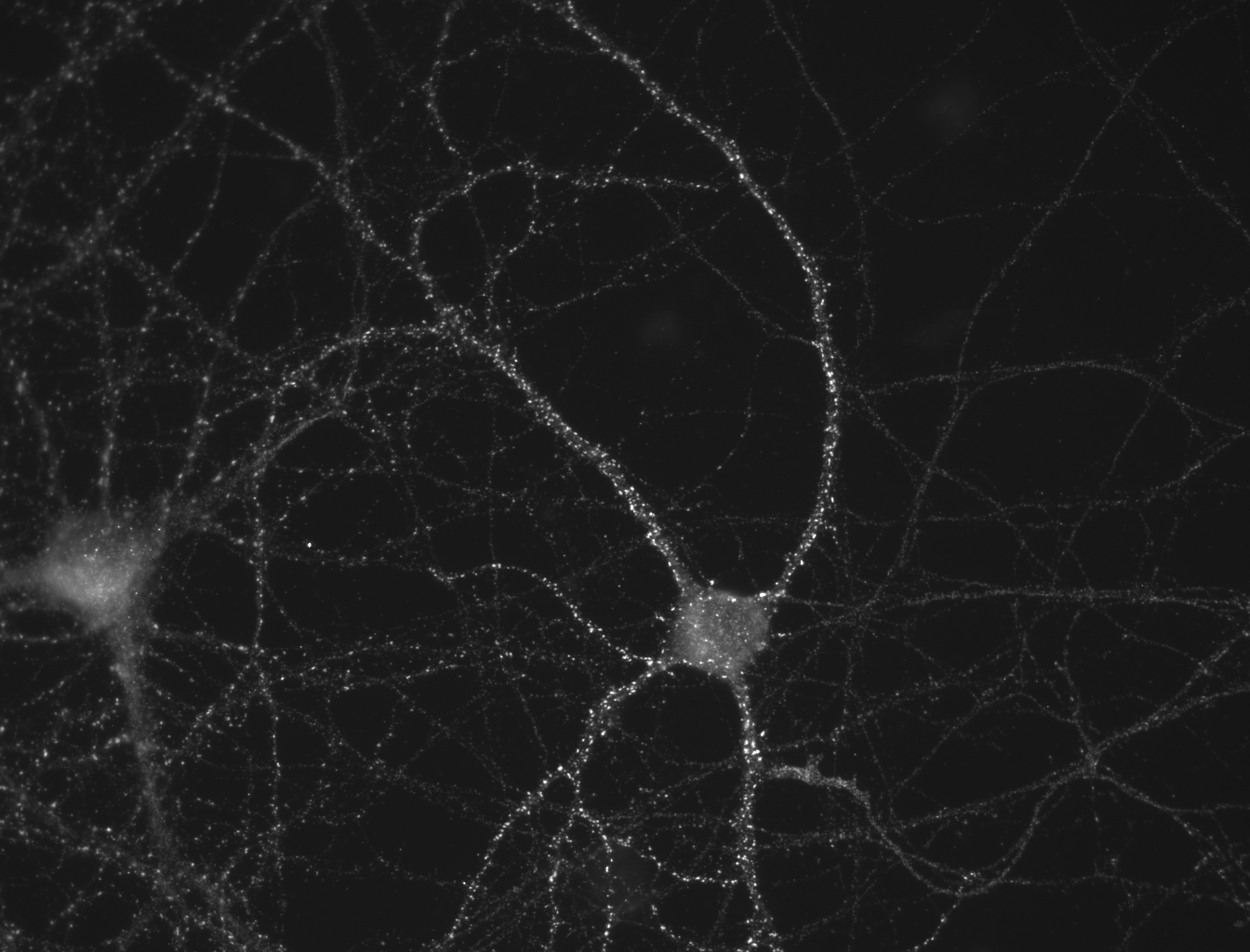

Supplement: Supplementary file 9 — Appendix. Fig. S1-10. [file 44318_2024_252_MOESM9_ESM.zip › Appendix. Fig. S1-10/Appendix. Fig. S4/S4 A/TrkC KI PSD95.tif]

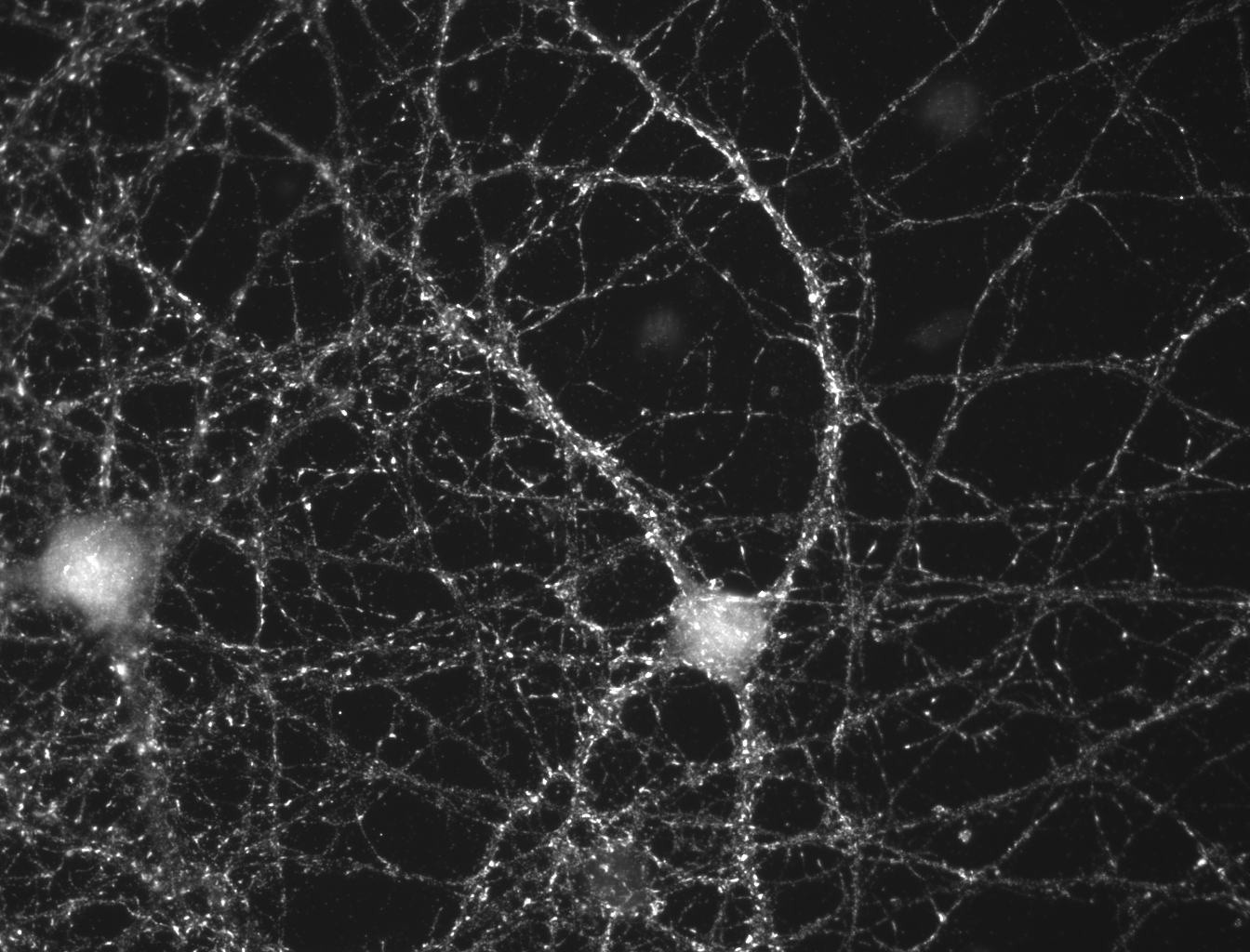

Supplement: Supplementary file 9 — Appendix. Fig. S1-10. [file 44318_2024_252_MOESM9_ESM.zip › Appendix. Fig. S1-10/Appendix. Fig. S4/S4 A/TrkC KI VGLUT1.tif]

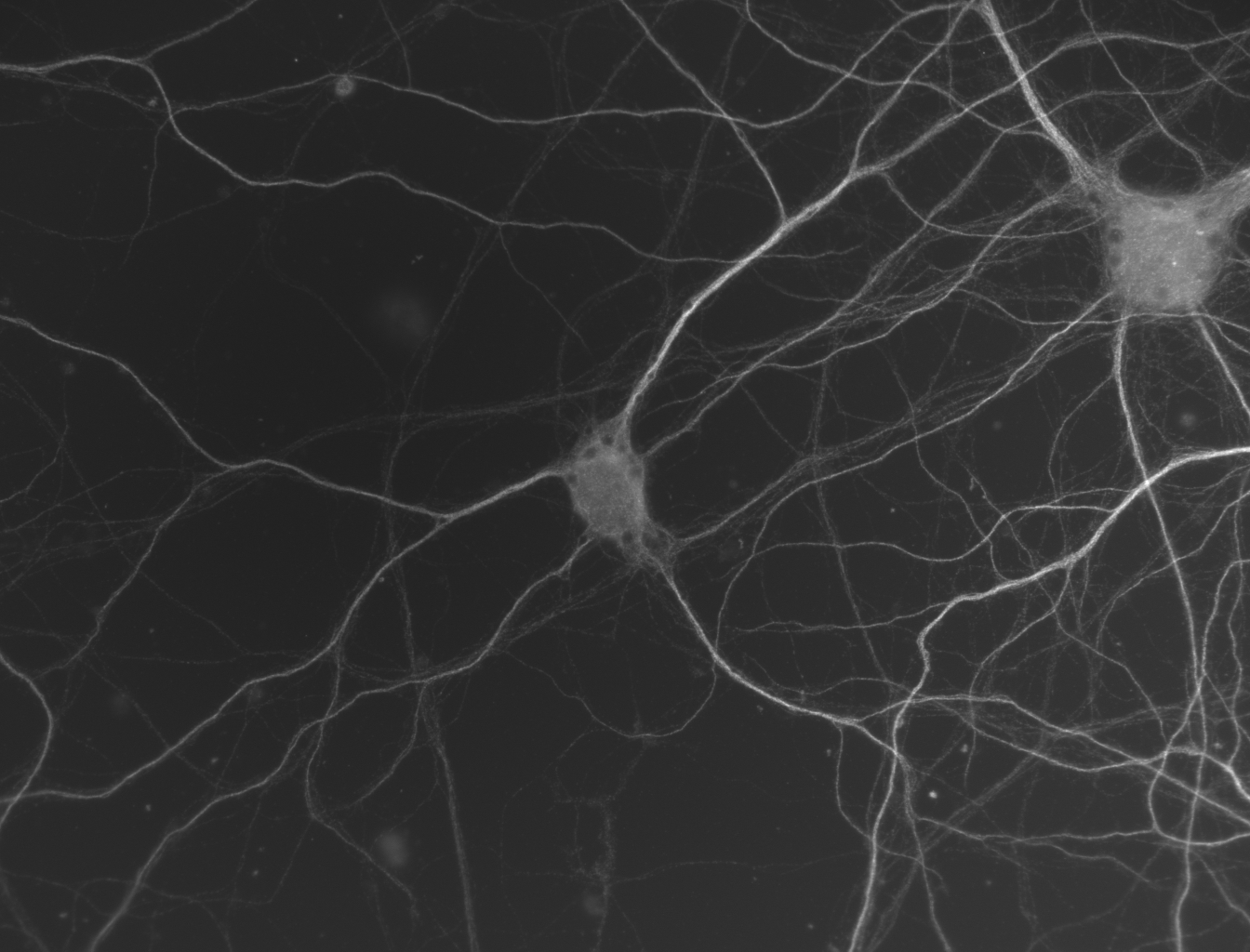

Supplement: Supplementary file 9 — Appendix. Fig. S1-10. [file 44318_2024_252_MOESM9_ESM.zip › Appendix. Fig. S1-10/Appendix. Fig. S4/S4 A/TrkC WT MAP2.tif]

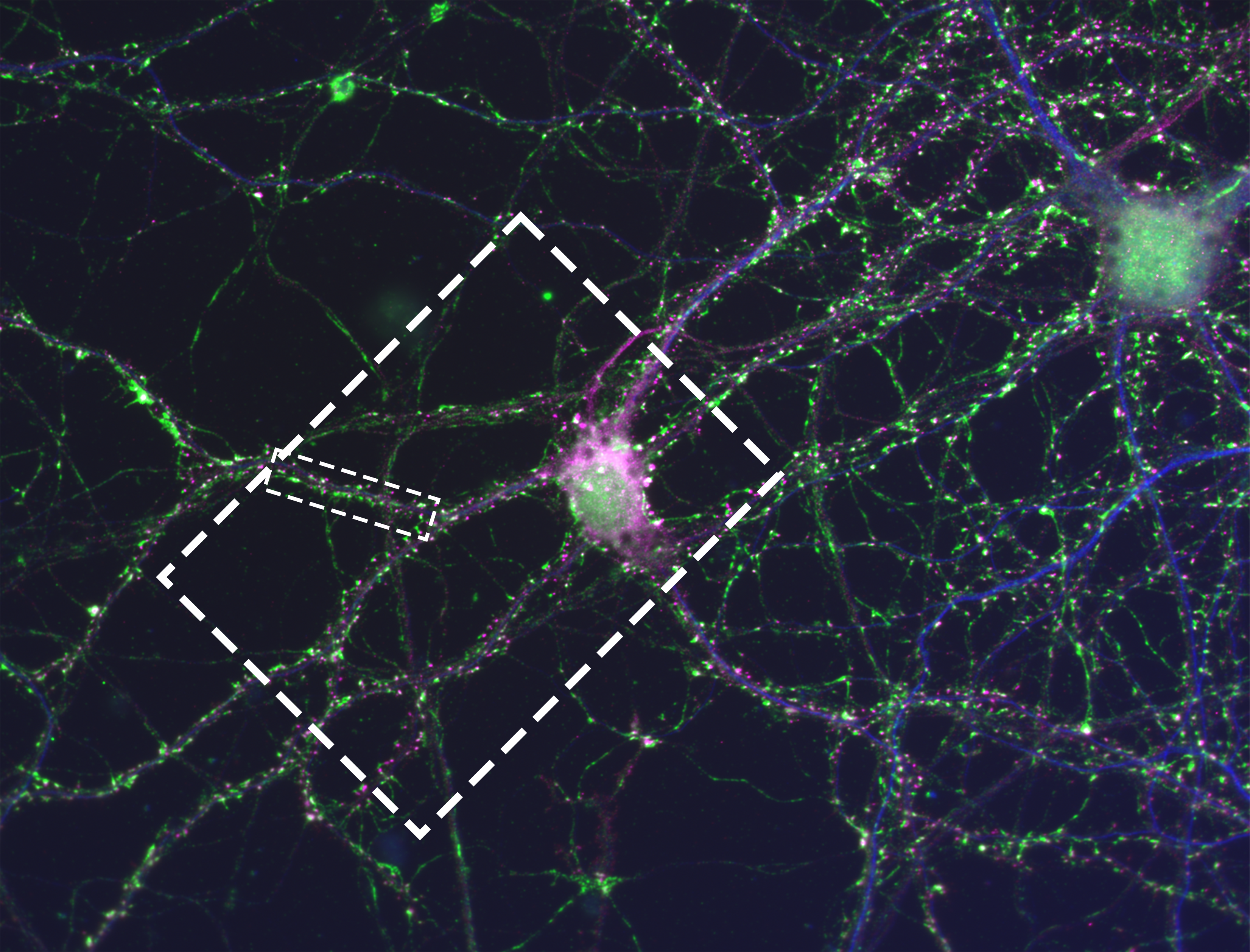

Supplement: Supplementary file 9 — Appendix. Fig. S1-10. [file 44318_2024_252_MOESM9_ESM.zip › Appendix. Fig. S1-10/Appendix. Fig. S4/S4 A/TrkC WT merged and annotated.png]

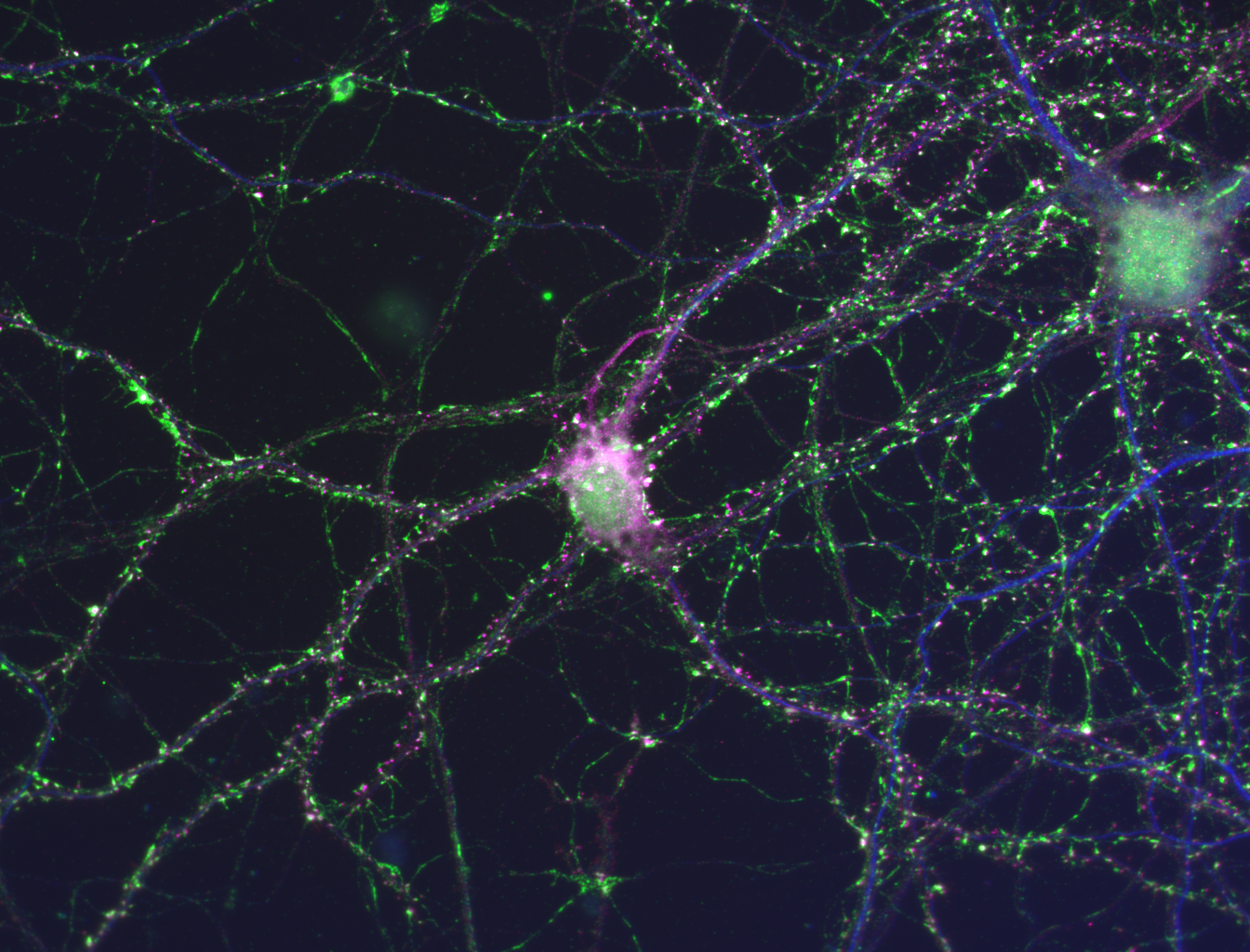

Supplement: Supplementary file 9 — Appendix. Fig. S1-10. [file 44318_2024_252_MOESM9_ESM.zip › Appendix. Fig. S1-10/Appendix. Fig. S4/S4 A/TrkC WT Merged.tif]

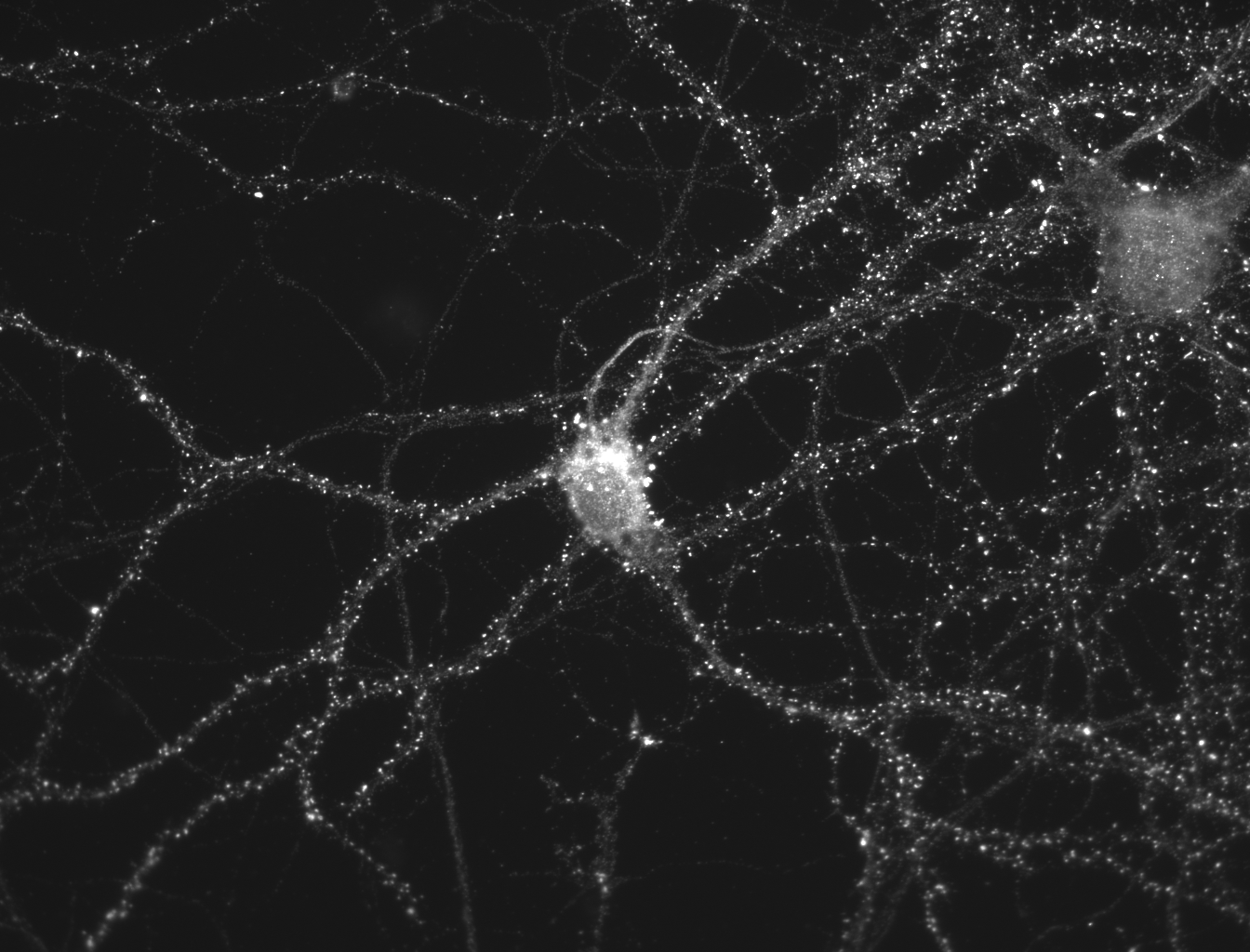

Supplement: Supplementary file 9 — Appendix. Fig. S1-10. [file 44318_2024_252_MOESM9_ESM.zip › Appendix. Fig. S1-10/Appendix. Fig. S4/S4 A/TrkC WT PSD95.tif]

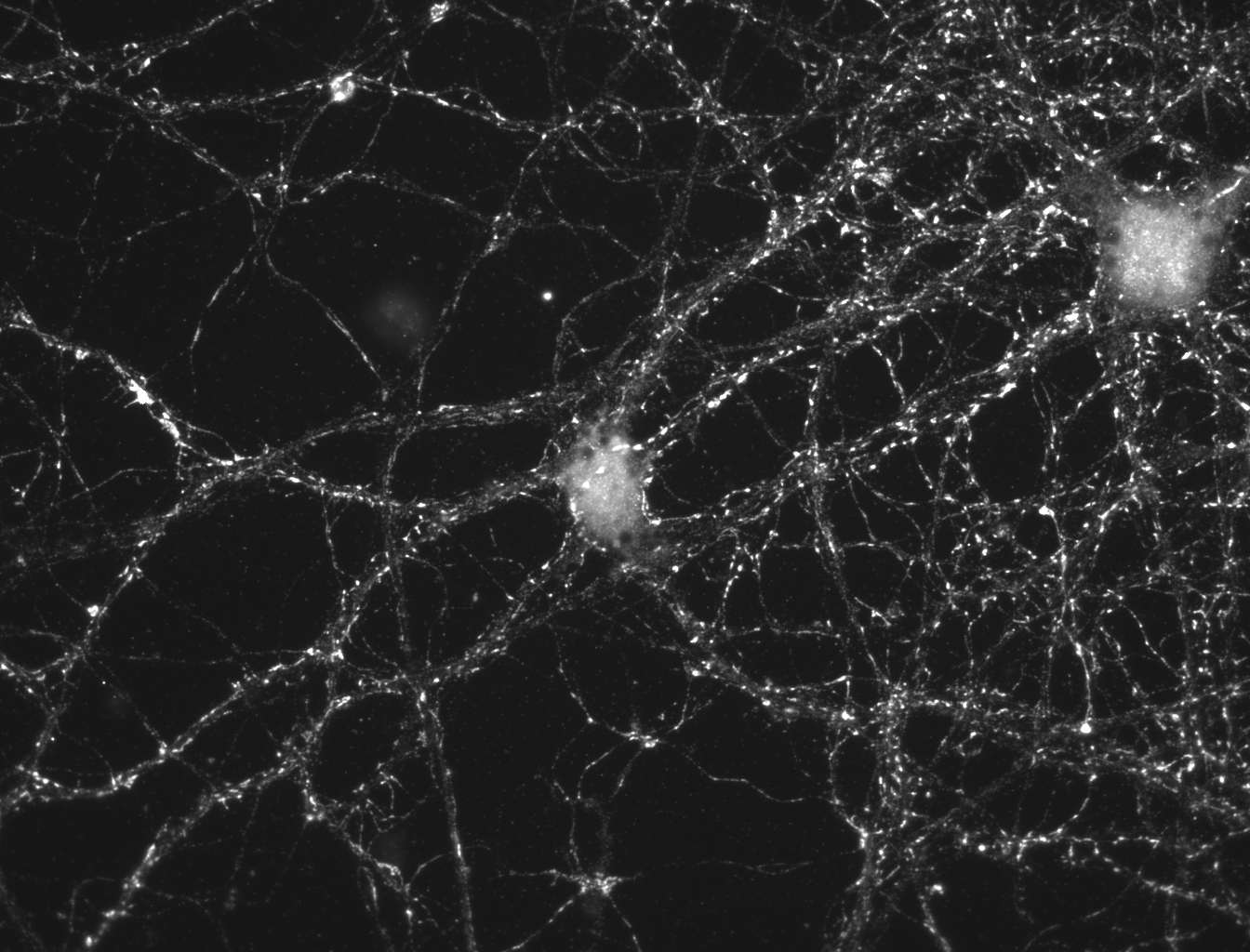

Supplement: Supplementary file 9 — Appendix. Fig. S1-10. [file 44318_2024_252_MOESM9_ESM.zip › Appendix. Fig. S1-10/Appendix. Fig. S4/S4 A/TrkC WT VGLUT1.tif]

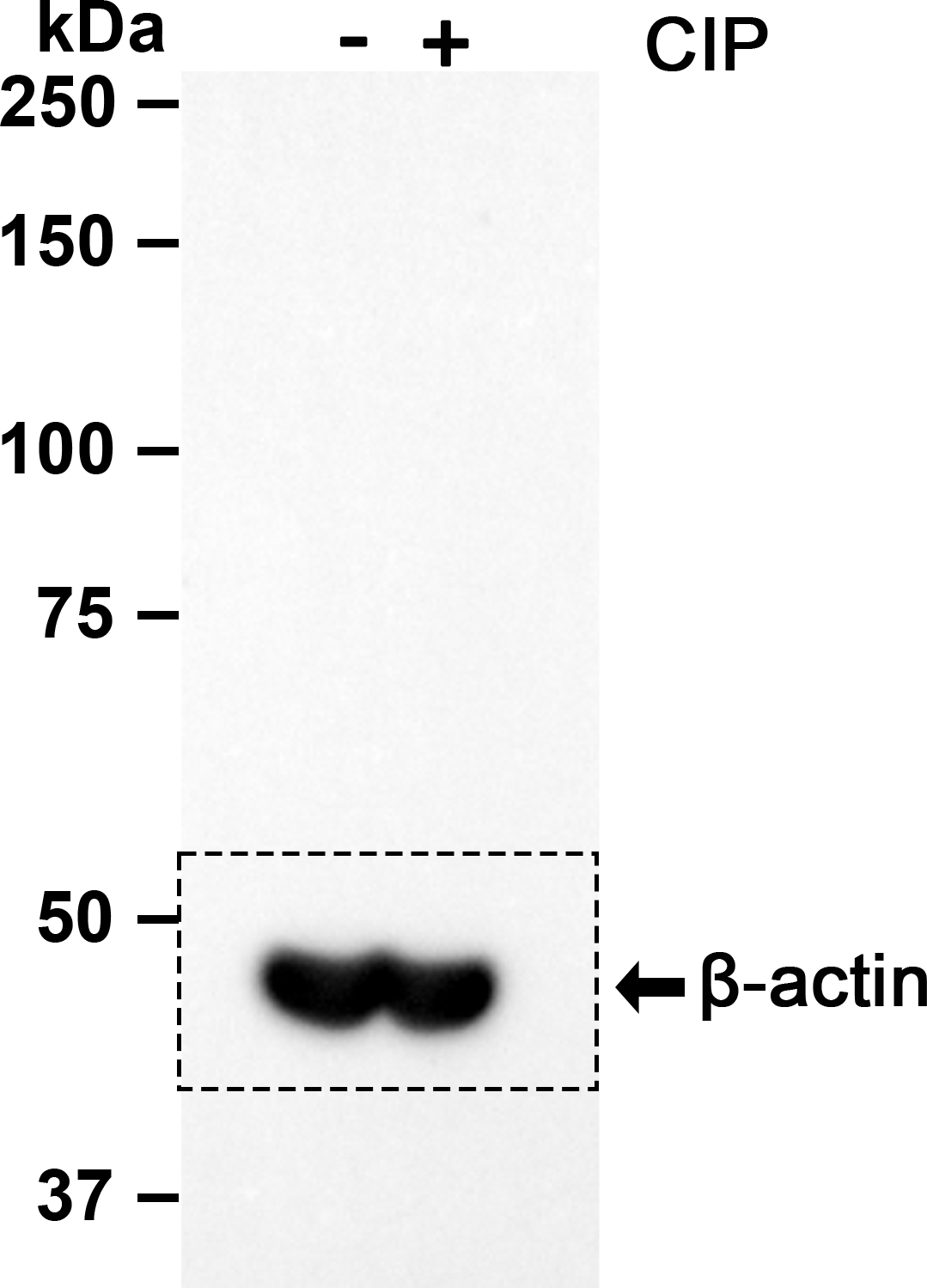

Supplement: Supplementary file 9 — Appendix. Fig. S1-10. [file 44318_2024_252_MOESM9_ESM.zip › Appendix. Fig. S1-10/Appendix. Fig. S6/S6 B/beta-actin Phos-Tag +CIP annotated.png]

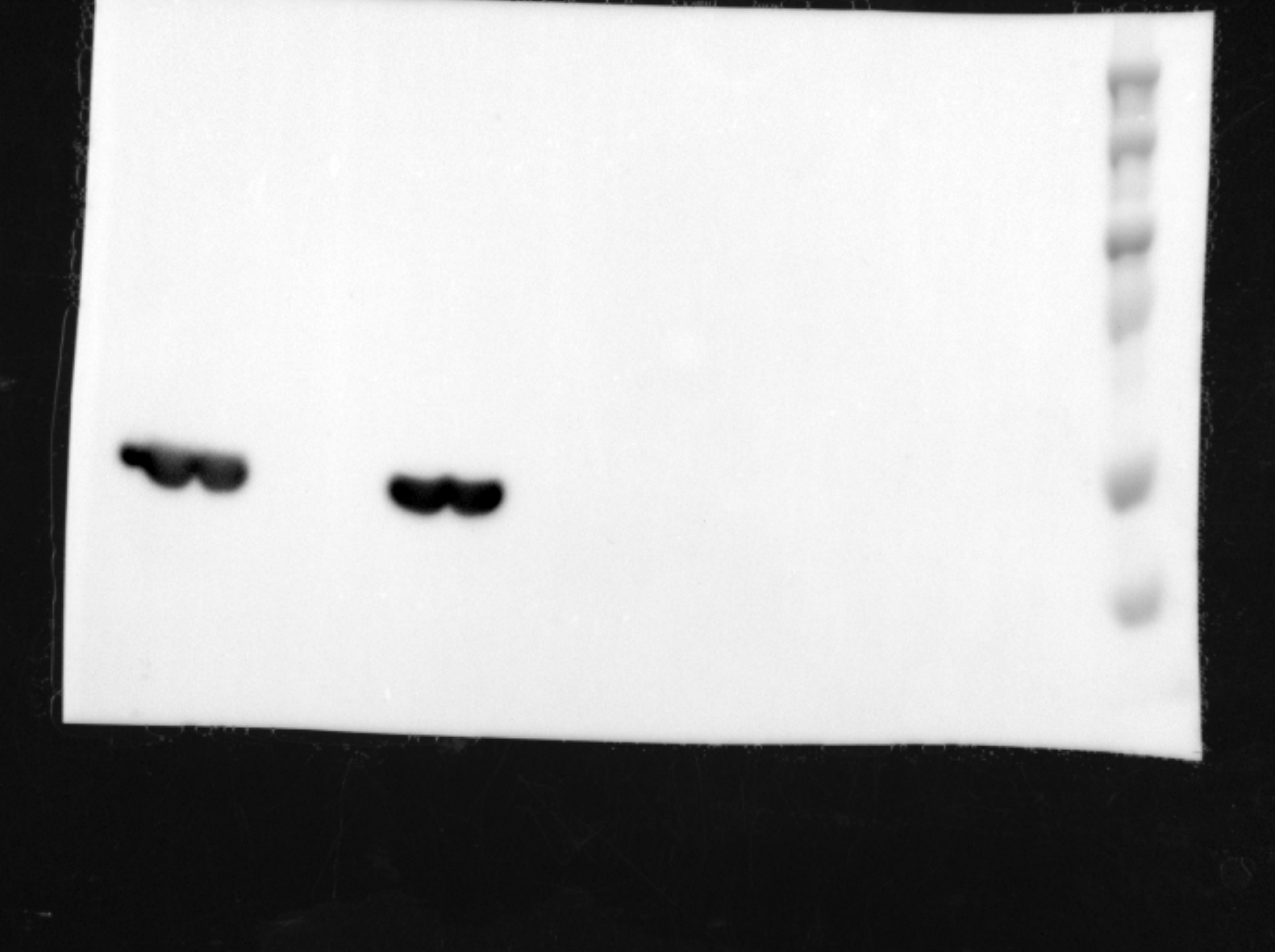

Supplement: Supplementary file 9 — Appendix. Fig. S1-10. [file 44318_2024_252_MOESM9_ESM.zip › Appendix. Fig. S1-10/Appendix. Fig. S6/S6 B/beta-actin Phos-Tag +CIP.tif]

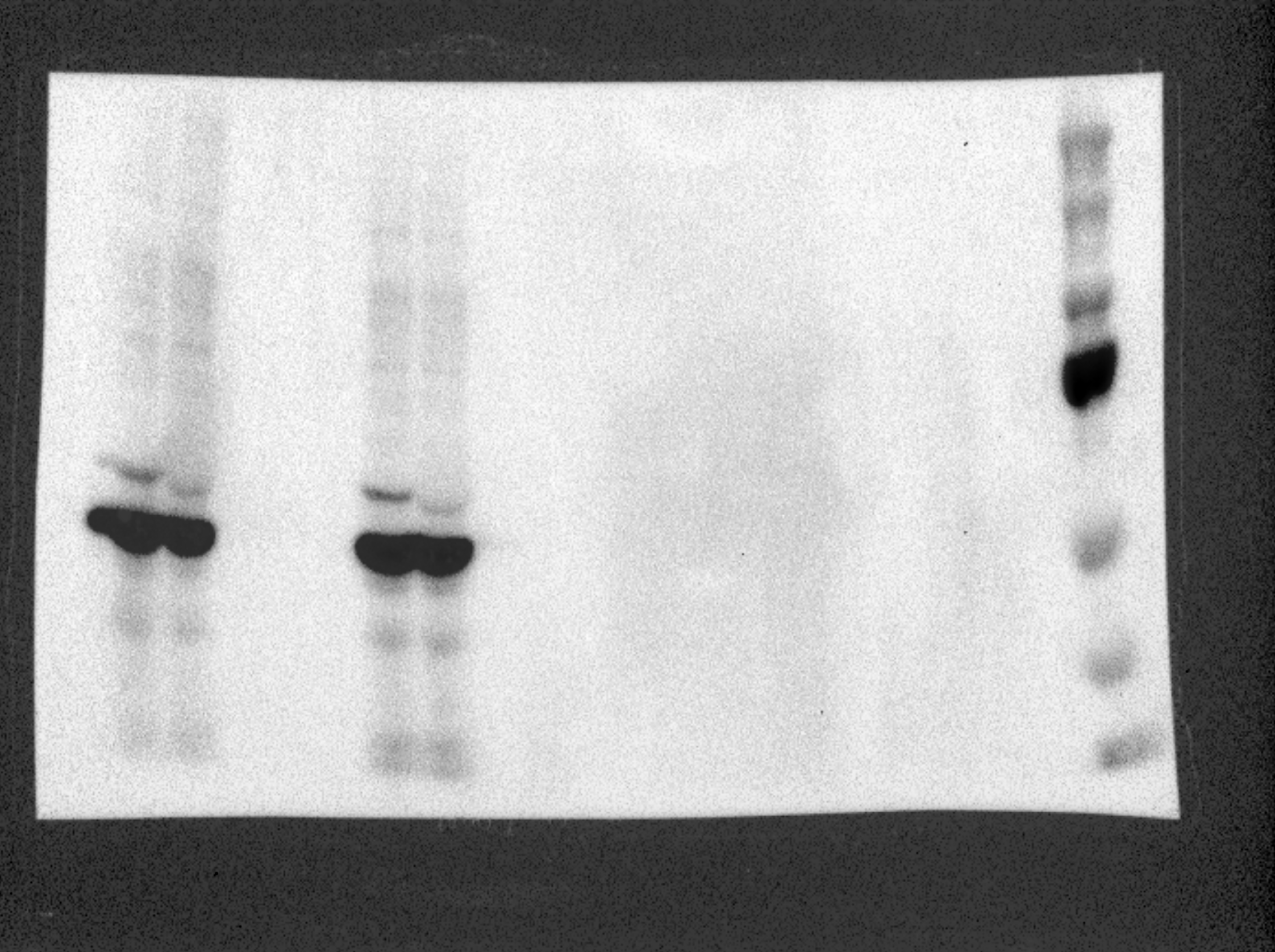

Supplement: Supplementary file 9 — Appendix. Fig. S1-10. [file 44318_2024_252_MOESM9_ESM.zip › Appendix. Fig. S1-10/Appendix. Fig. S6/S6 B/ITPKA Phos-Tag SDS-PAGE +CIP.tif]

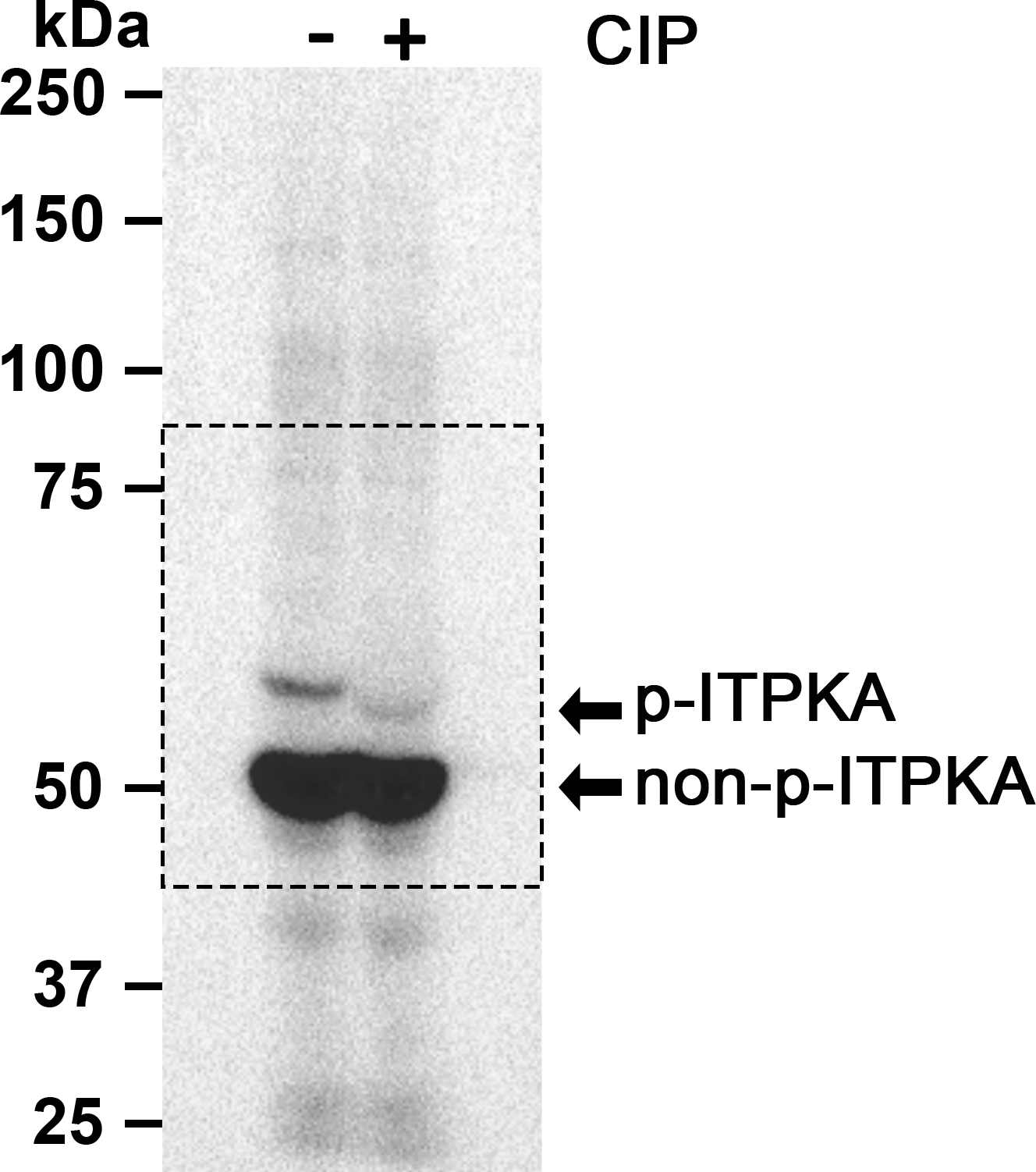

Supplement: Supplementary file 9 — Appendix. Fig. S1-10. [file 44318_2024_252_MOESM9_ESM.zip › Appendix. Fig. S1-10/Appendix. Fig. S6/S6 B/ITPKA_Phos Tag SDS-PAGE +CIP annotated.png]
