# Supplementary figures and images for: The TrkC-PTPσ complex governs synapse maturation and anxiogenic avoidance via synaptic protein phosphorylation (part 2 of 2)
Source: EMBO J. 2024 Sep 27;43(22):5690–717. doi: 10.1038/s44318-024-00252-9 (PMC11574141; doi:10.1038/s44318-024-00252-9)

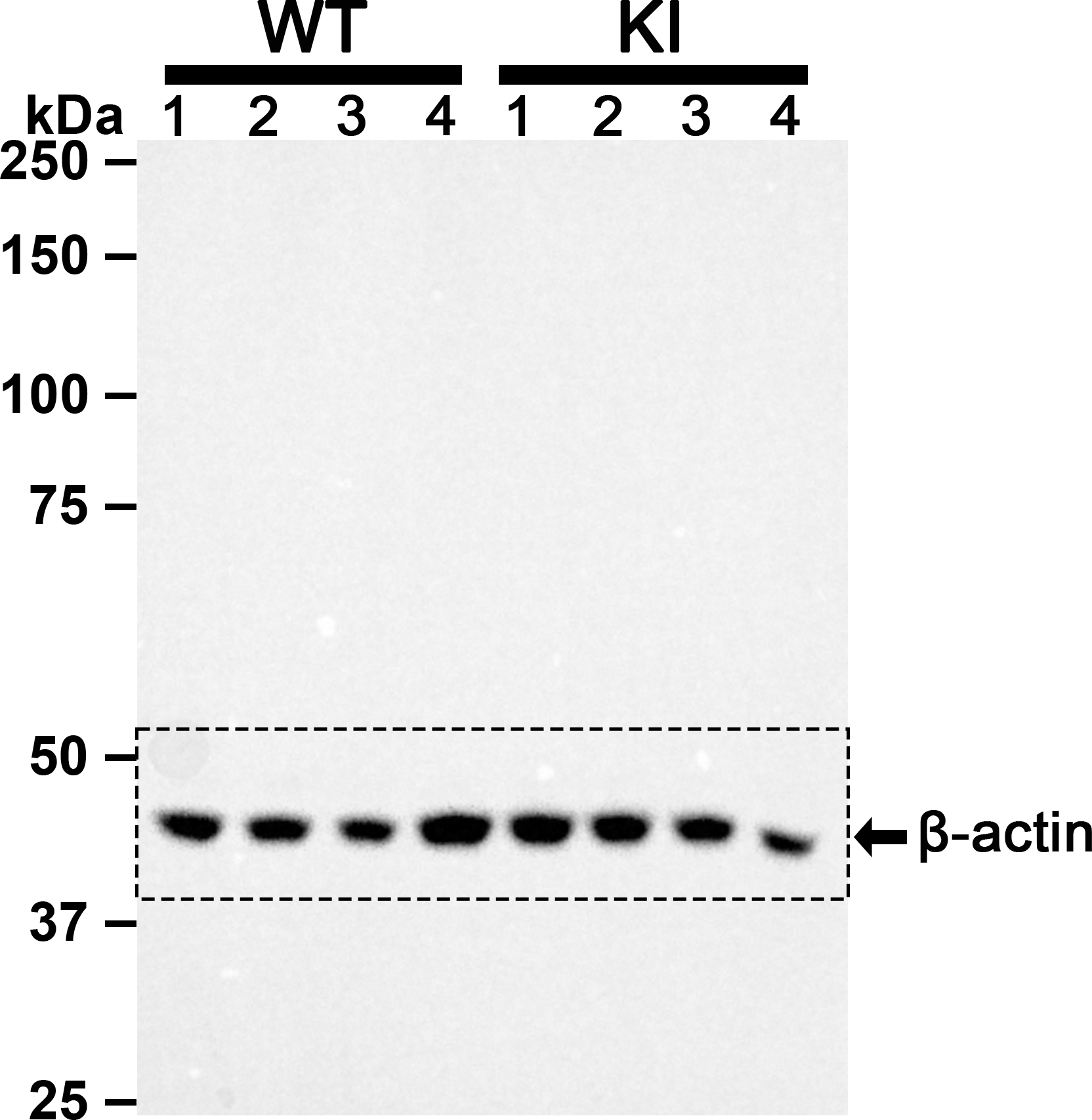

Supplement: Supplementary file 9 — Appendix. Fig. S1-10. [file 44318_2024_252_MOESM9_ESM.zip › Appendix. Fig. S1-10/Appendix. Fig. S6/S6 C/beta-actin Phos-Tag annotated.png]

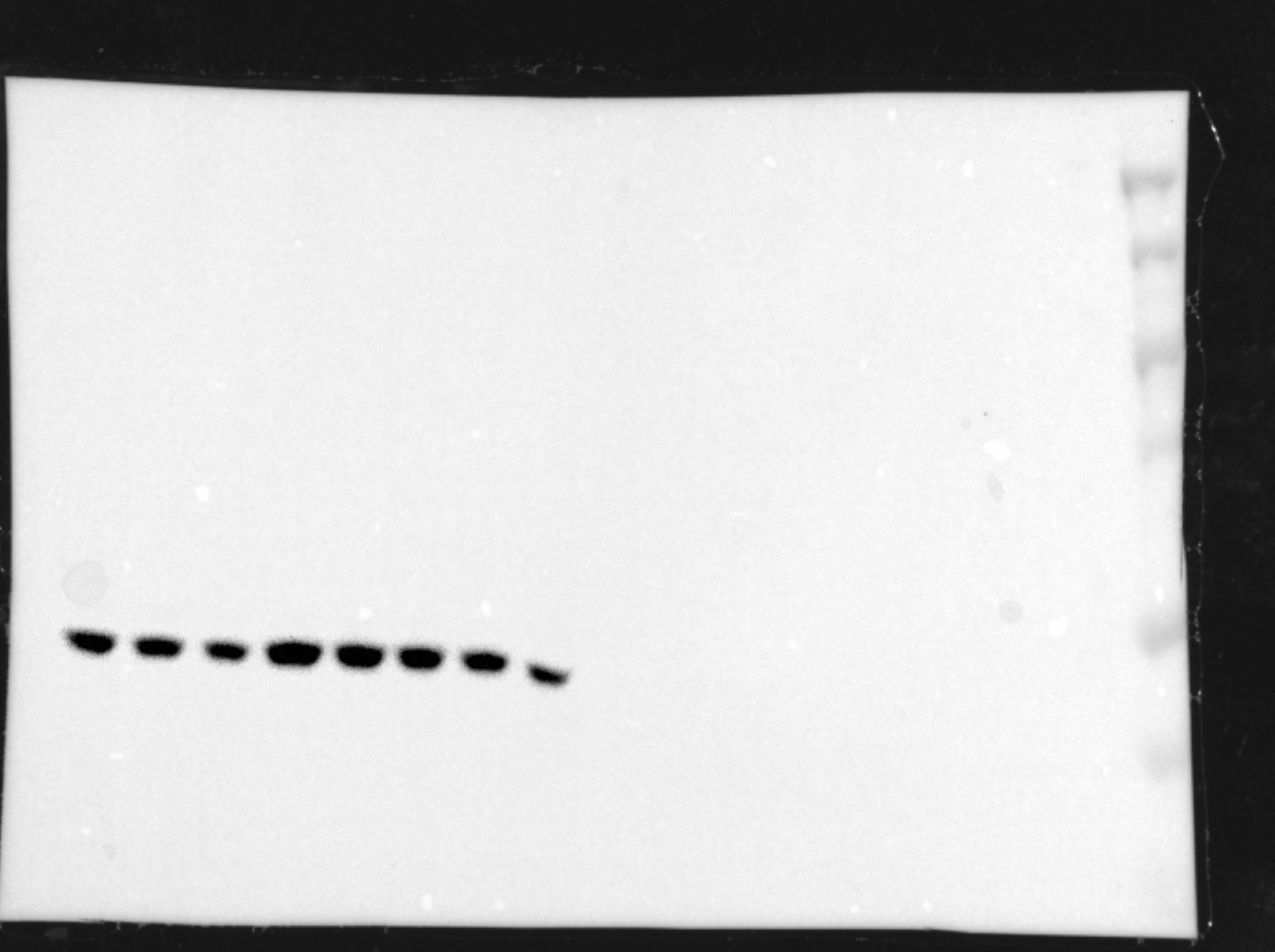

Supplement: Supplementary file 9 — Appendix. Fig. S1-10. [file 44318_2024_252_MOESM9_ESM.zip › Appendix. Fig. S1-10/Appendix. Fig. S6/S6 C/beta-actin Phos-Tag.tif]

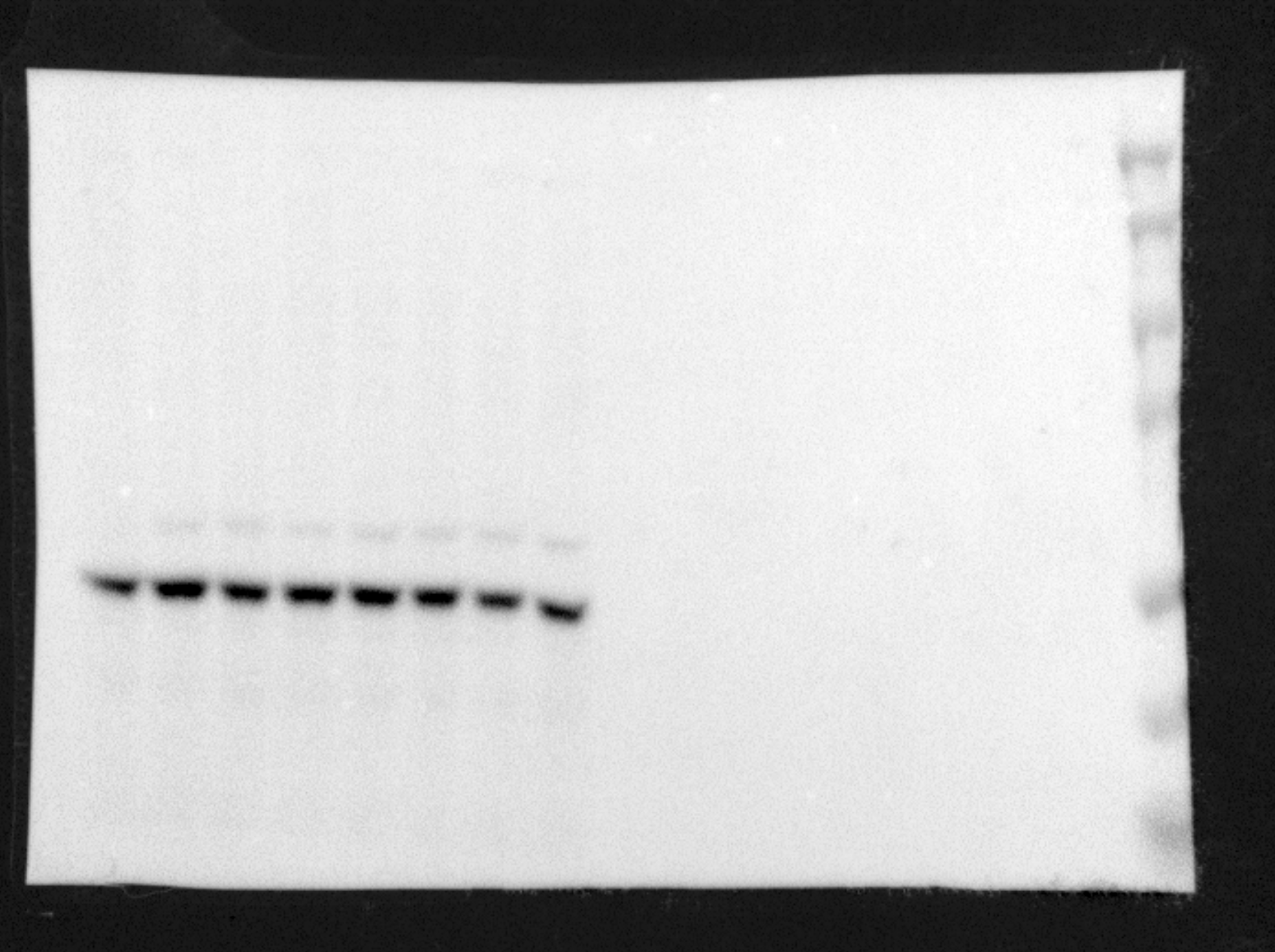

Supplement: Supplementary file 9 — Appendix. Fig. S1-10. [file 44318_2024_252_MOESM9_ESM.zip › Appendix. Fig. S1-10/Appendix. Fig. S6/S6 C/ITPKA Phos-Tag SDS-PAGE (short exposure).tif]

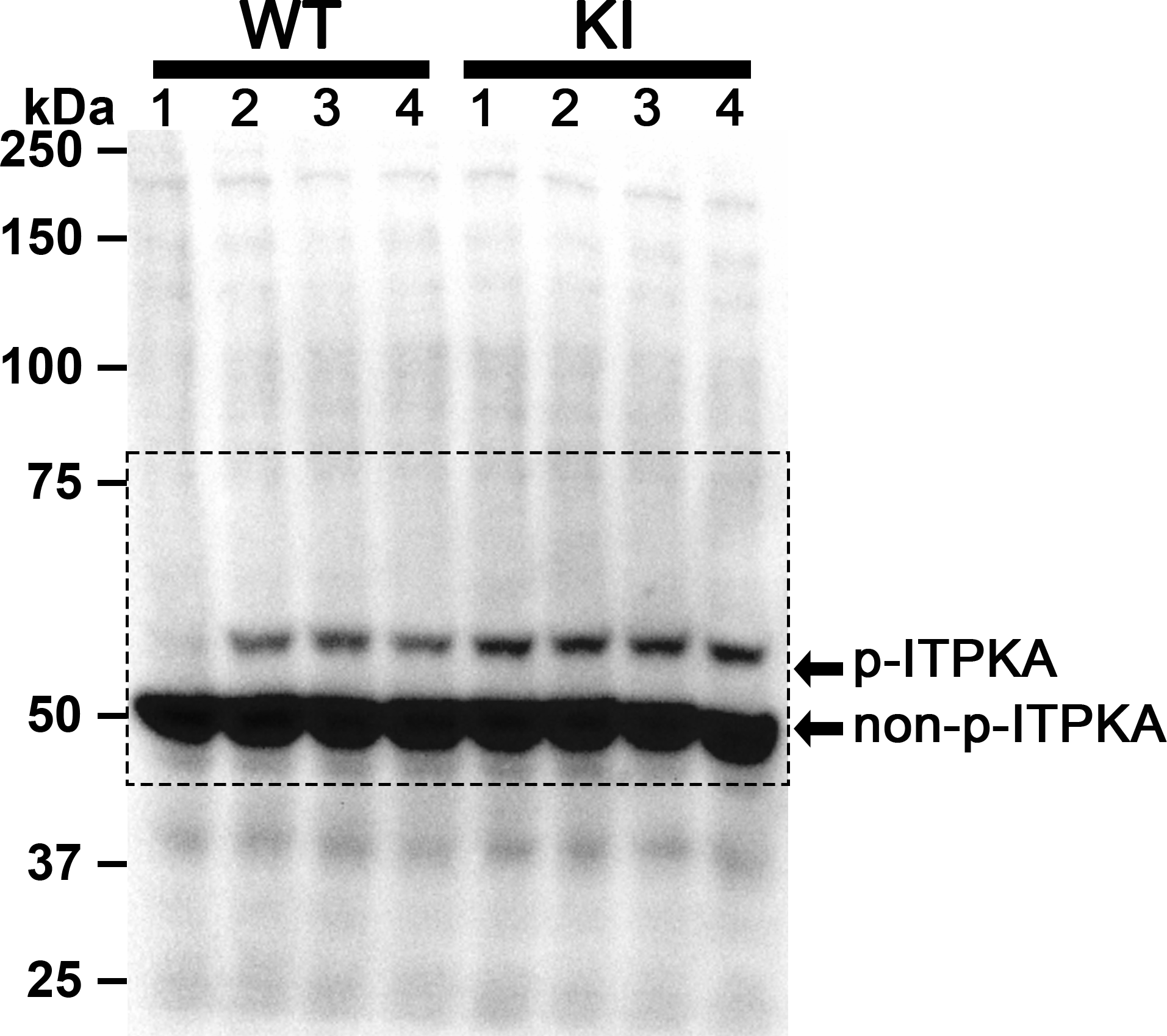

Supplement: Supplementary file 9 — Appendix. Fig. S1-10. [file 44318_2024_252_MOESM9_ESM.zip › Appendix. Fig. S1-10/Appendix. Fig. S6/S6 C/ITPKA Phos-Tag SDS-PAGE annotated.png]

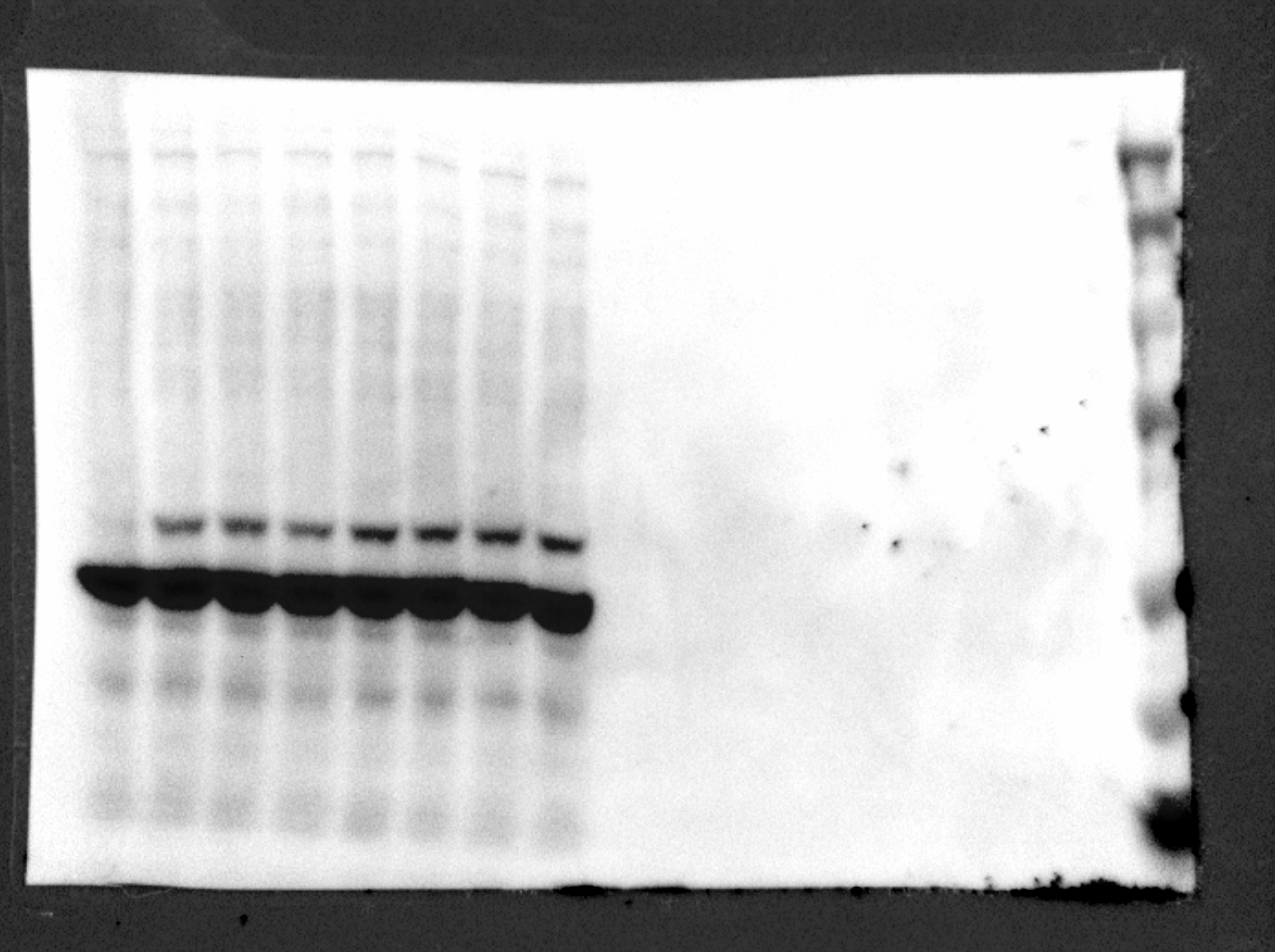

Supplement: Supplementary file 9 — Appendix. Fig. S1-10. [file 44318_2024_252_MOESM9_ESM.zip › Appendix. Fig. S1-10/Appendix. Fig. S6/S6 C/ITPKA Phos-Tag SDS-PAGE.tif]

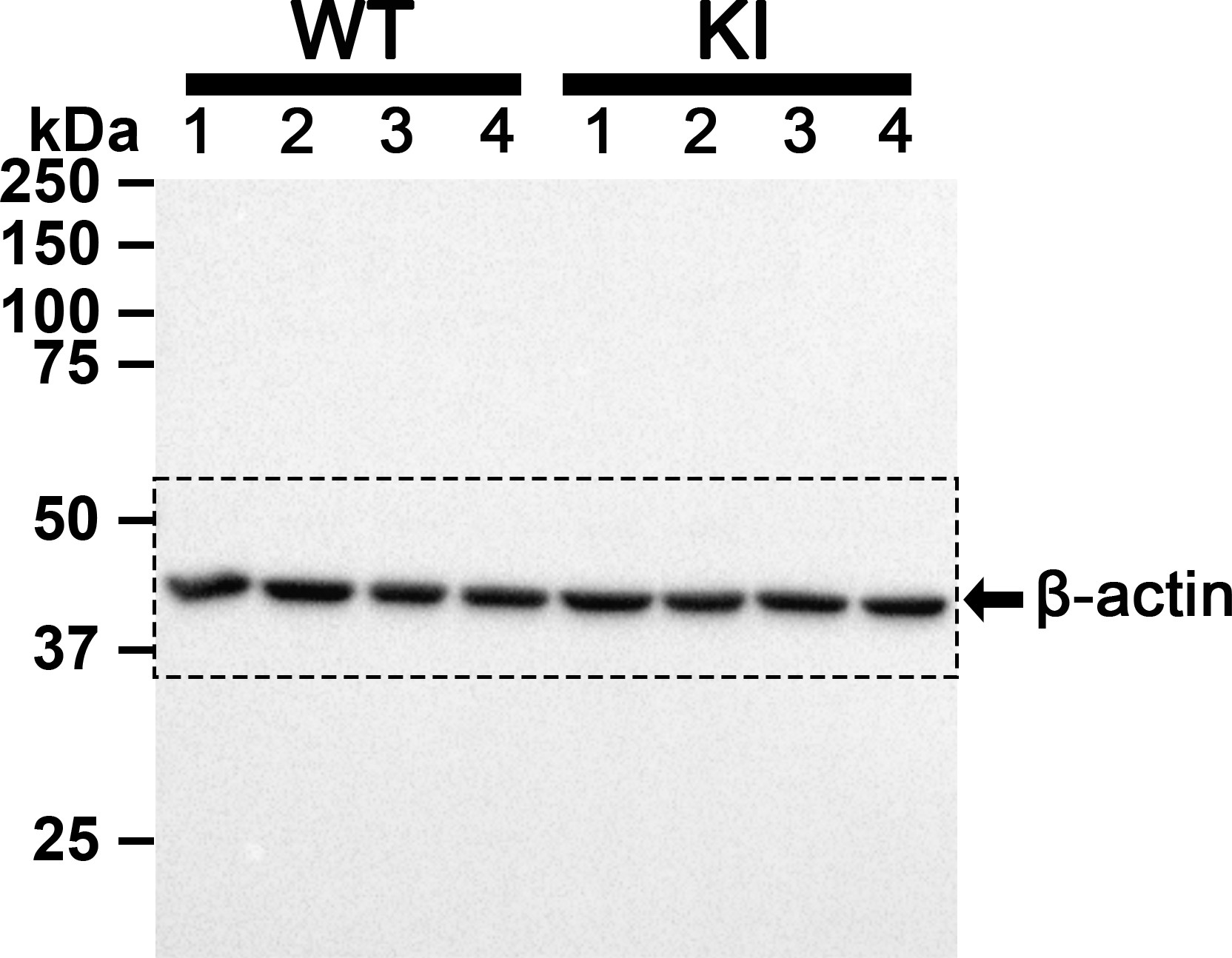

Supplement: Supplementary file 9 — Appendix. Fig. S1-10. [file 44318_2024_252_MOESM9_ESM.zip › Appendix. Fig. S1-10/Appendix. Fig. S6/S6 D/beta-actin SDS-PAGE (no-Phos-Tag) annotated.png]

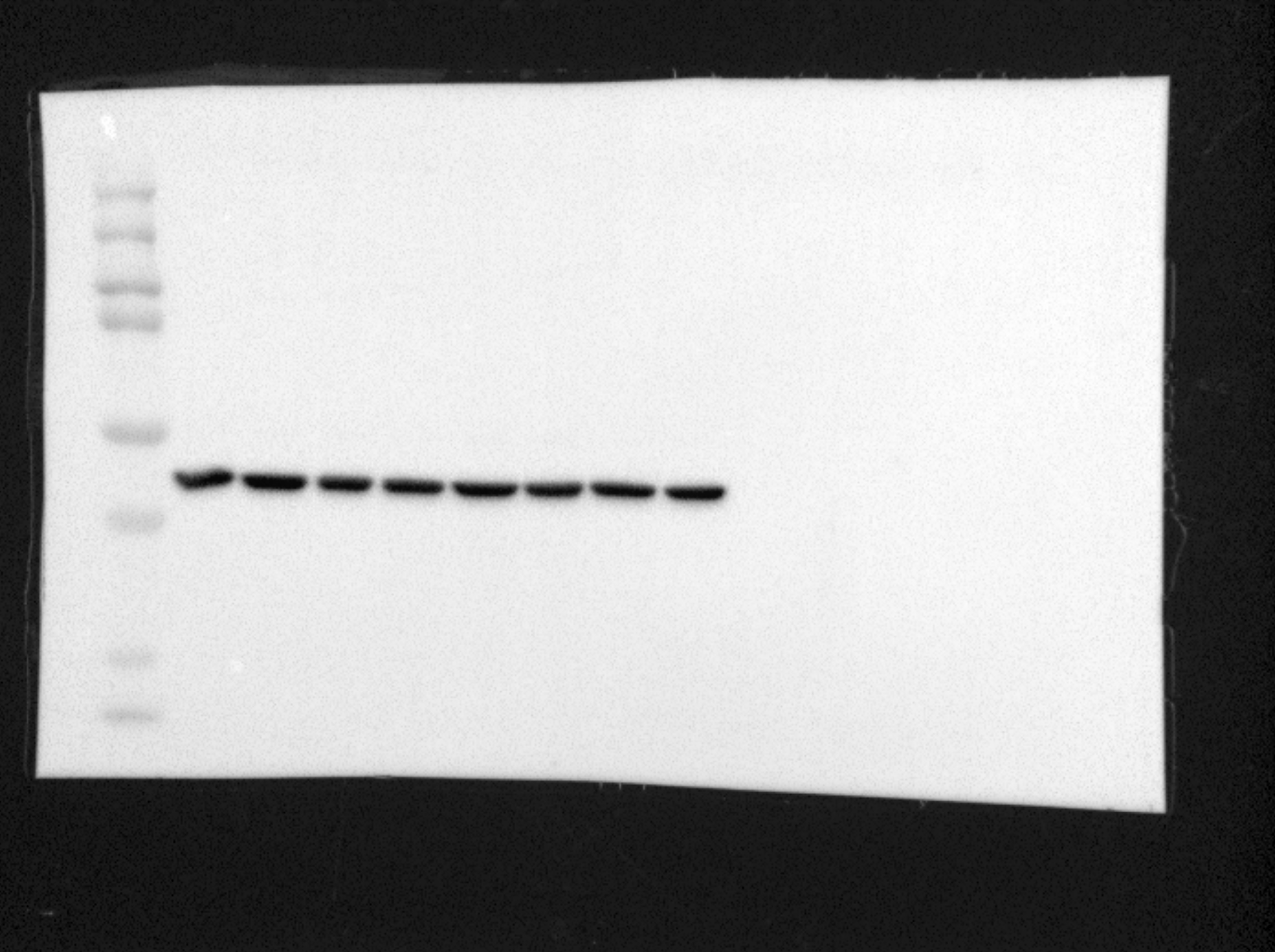

Supplement: Supplementary file 9 — Appendix. Fig. S1-10. [file 44318_2024_252_MOESM9_ESM.zip › Appendix. Fig. S1-10/Appendix. Fig. S6/S6 D/beta-actin SDS-PAGE (no-Phos-Tag).tif]

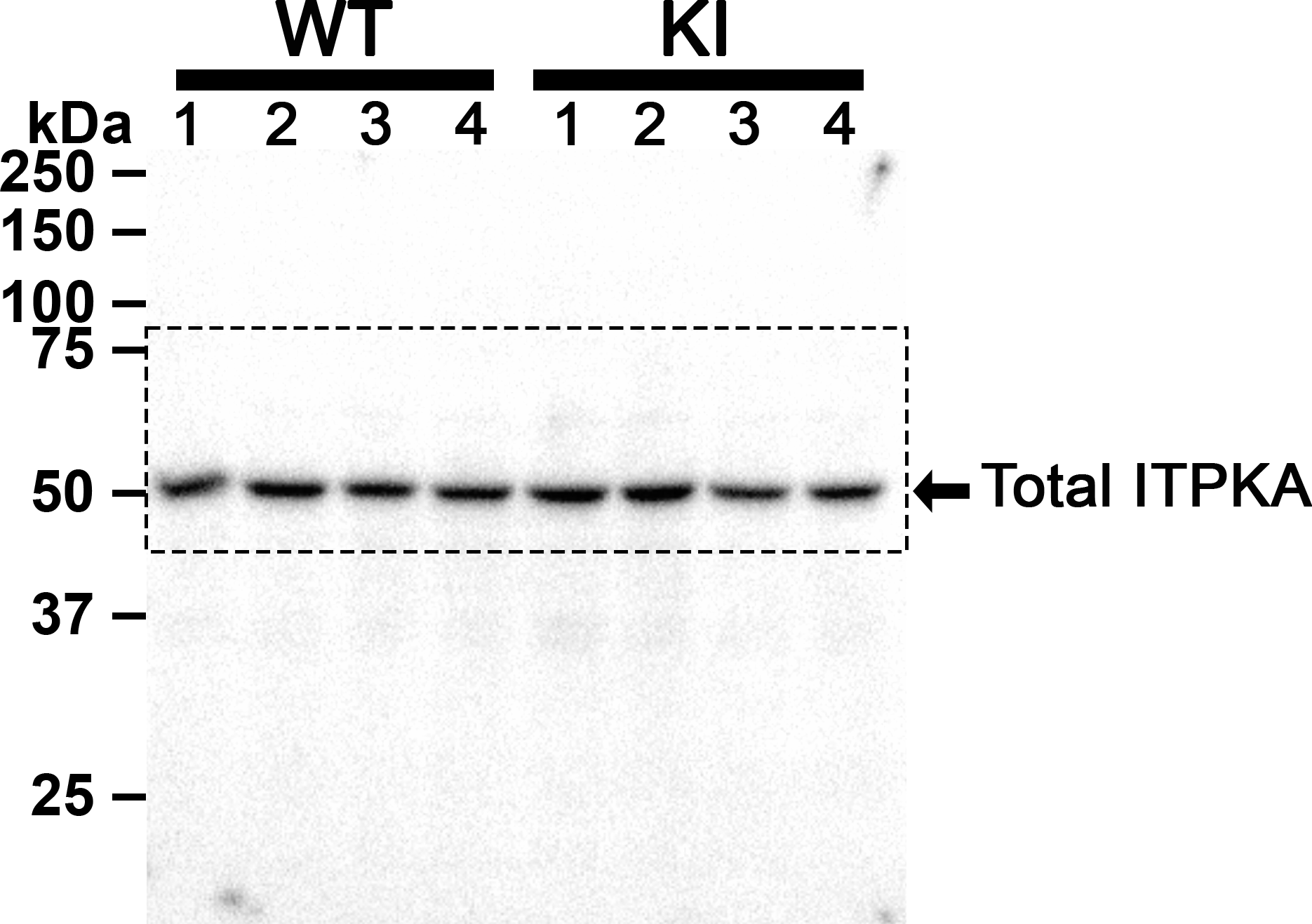

Supplement: Supplementary file 9 — Appendix. Fig. S1-10. [file 44318_2024_252_MOESM9_ESM.zip › Appendix. Fig. S1-10/Appendix. Fig. S6/S6 D/ITPKA SDS-PAGE (no Phos-Tag) annotated.png]

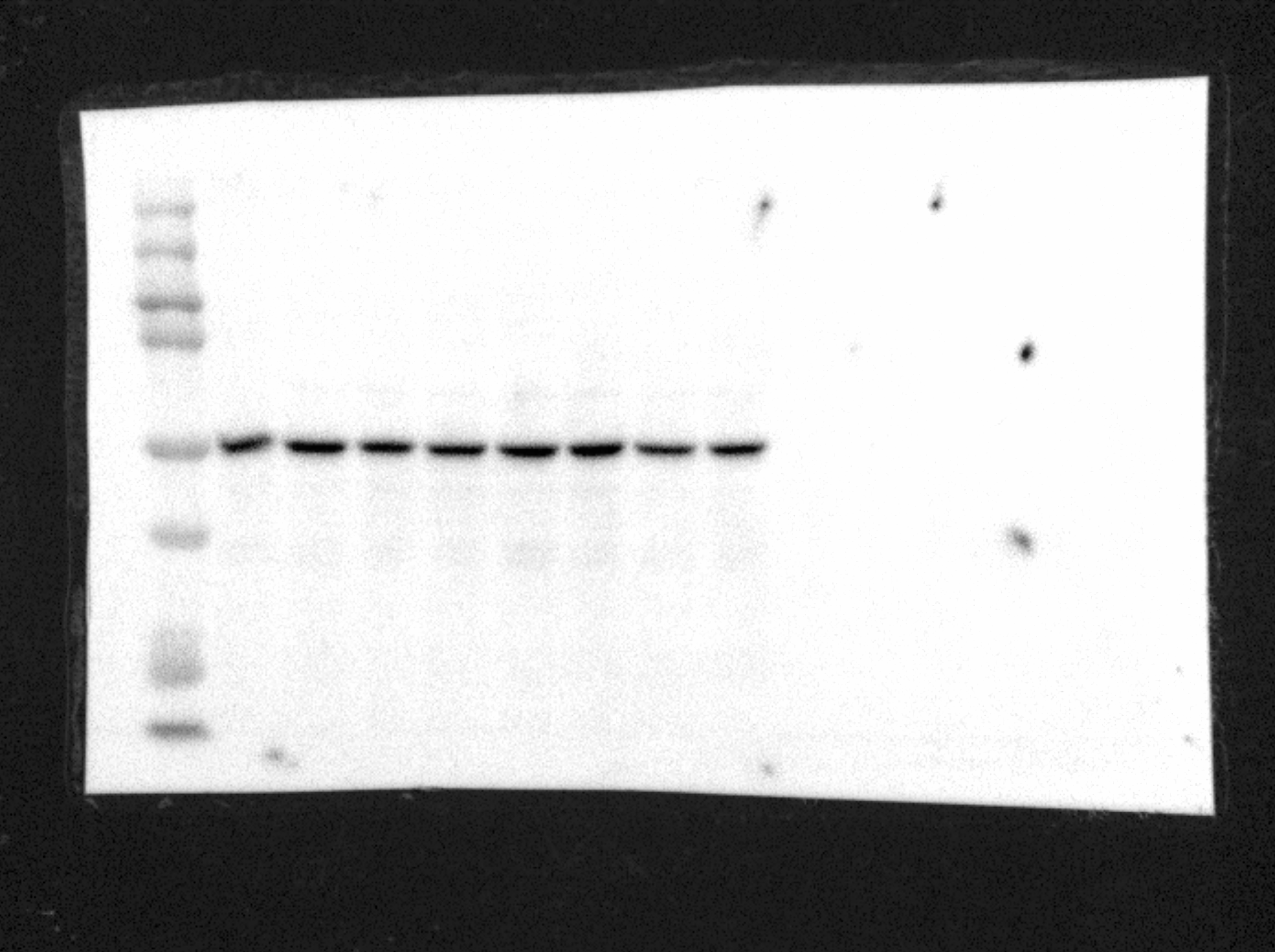

Supplement: Supplementary file 9 — Appendix. Fig. S1-10. [file 44318_2024_252_MOESM9_ESM.zip › Appendix. Fig. S1-10/Appendix. Fig. S6/S6 D/ITPKA SDS-PAGE (no Phos-Tag).tif]

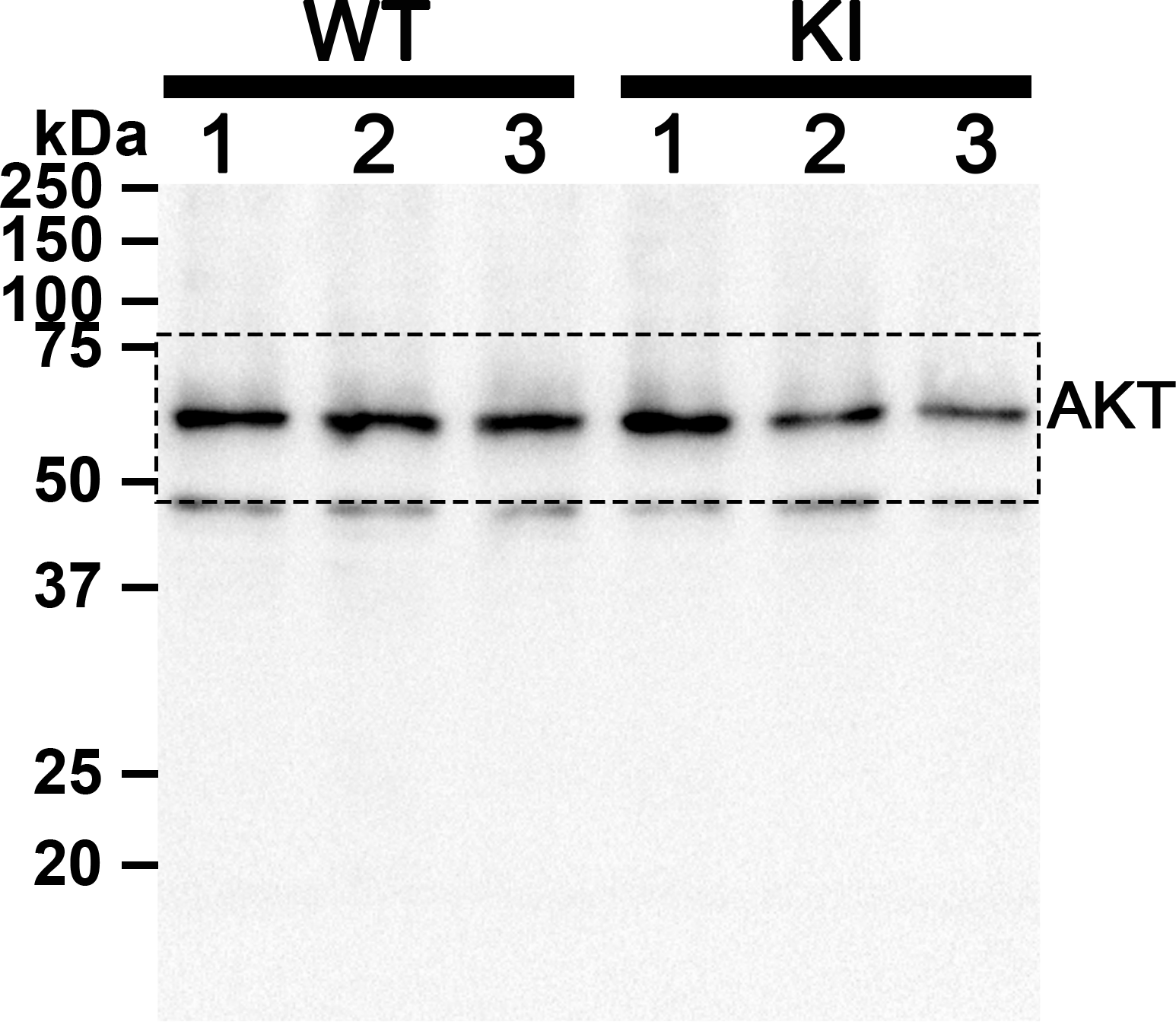

Supplement: Supplementary file 9 — Appendix. Fig. S1-10. [file 44318_2024_252_MOESM9_ESM.zip › Appendix. Fig. S1-10/Appendix. Fig. S8/S8 C/AKT annotated.png]

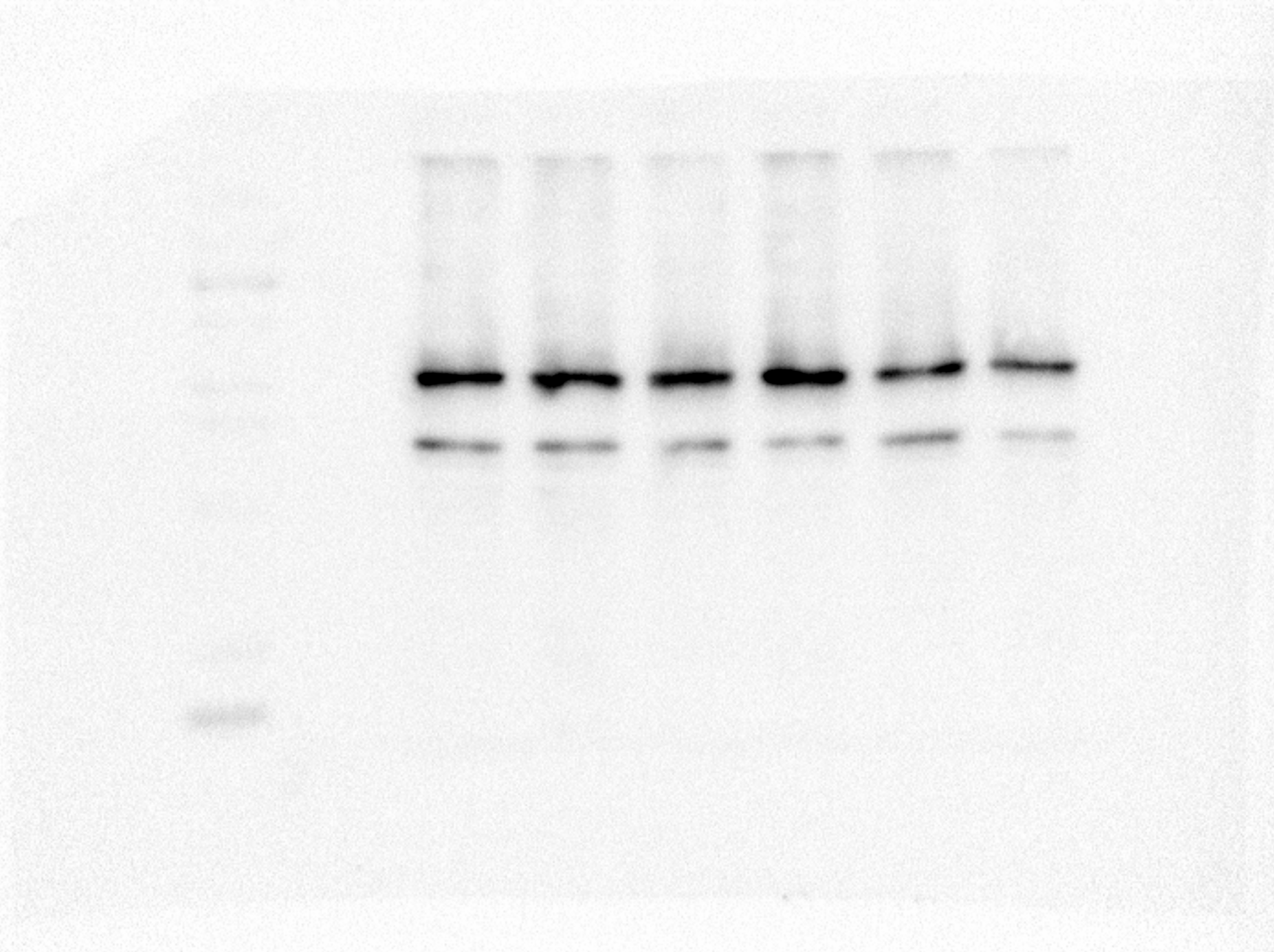

Supplement: Supplementary file 9 — Appendix. Fig. S1-10. [file 44318_2024_252_MOESM9_ESM.zip › Appendix. Fig. S1-10/Appendix. Fig. S8/S8 C/AKT.tif]

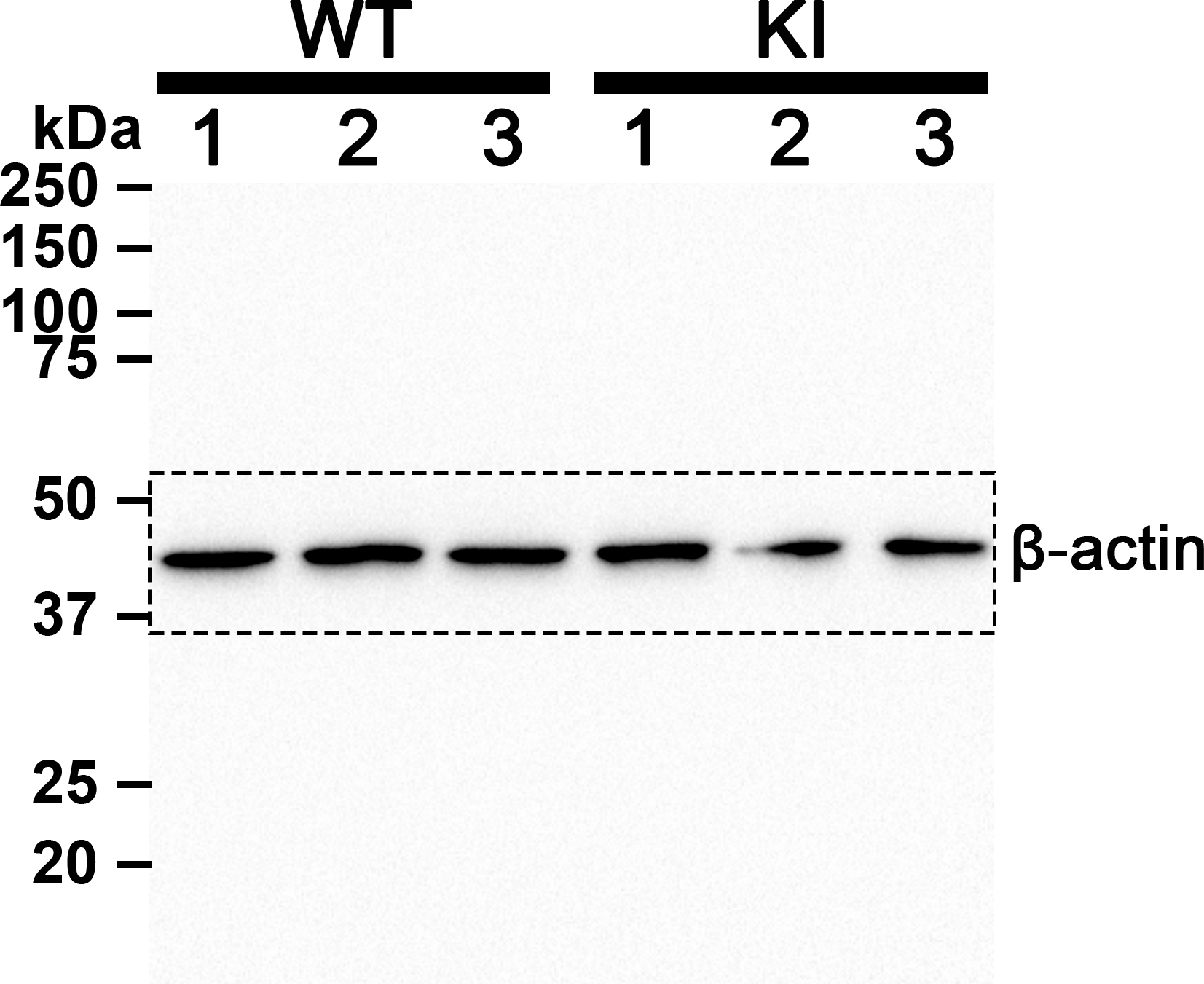

Supplement: Supplementary file 9 — Appendix. Fig. S1-10. [file 44318_2024_252_MOESM9_ESM.zip › Appendix. Fig. S1-10/Appendix. Fig. S8/S8 C/beta-actin annotated.png]

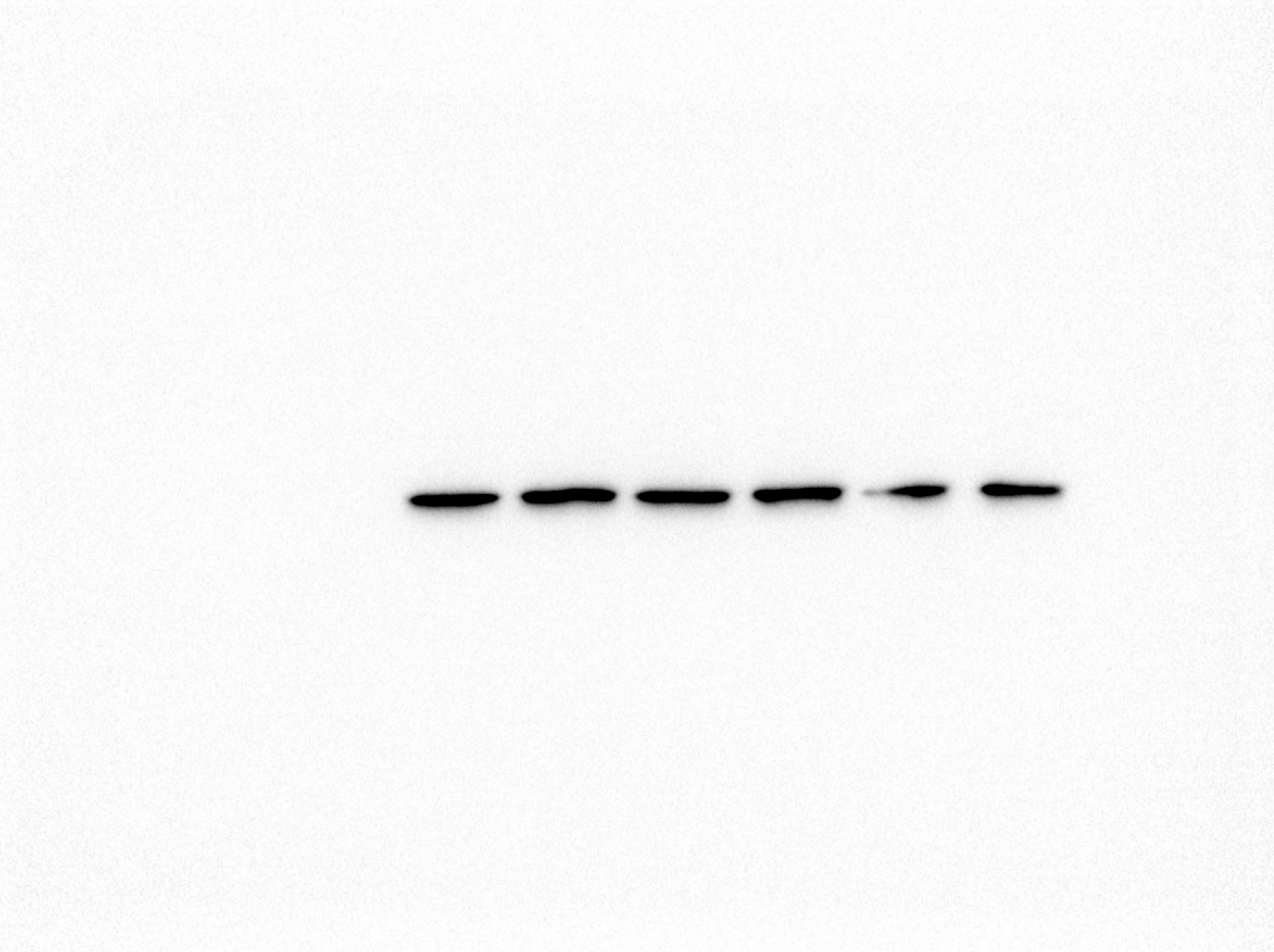

Supplement: Supplementary file 9 — Appendix. Fig. S1-10. [file 44318_2024_252_MOESM9_ESM.zip › Appendix. Fig. S1-10/Appendix. Fig. S8/S8 C/beta-actin.tif]

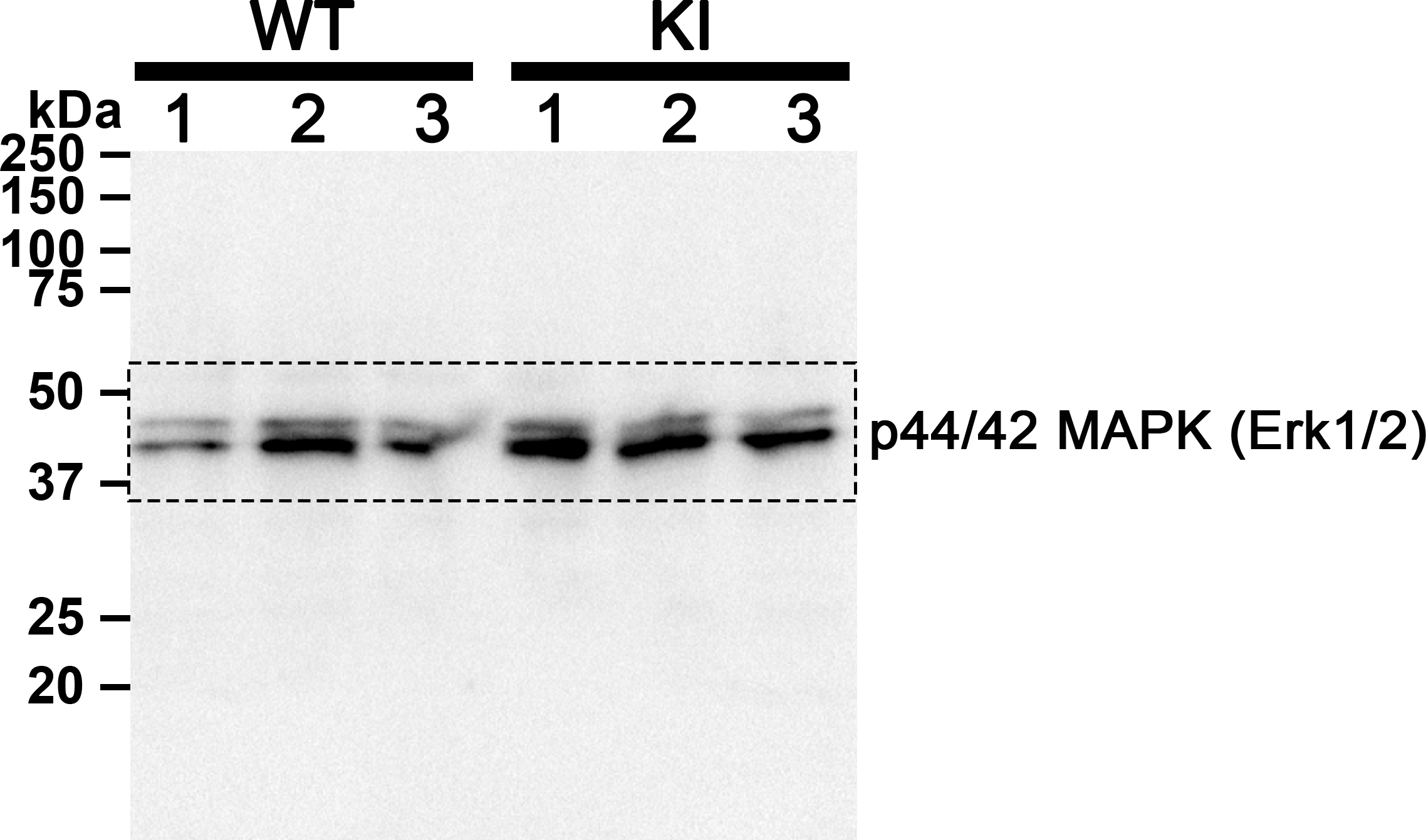

Supplement: Supplementary file 9 — Appendix. Fig. S1-10. [file 44318_2024_252_MOESM9_ESM.zip › Appendix. Fig. S1-10/Appendix. Fig. S8/S8 C/MAPK annotated.png]

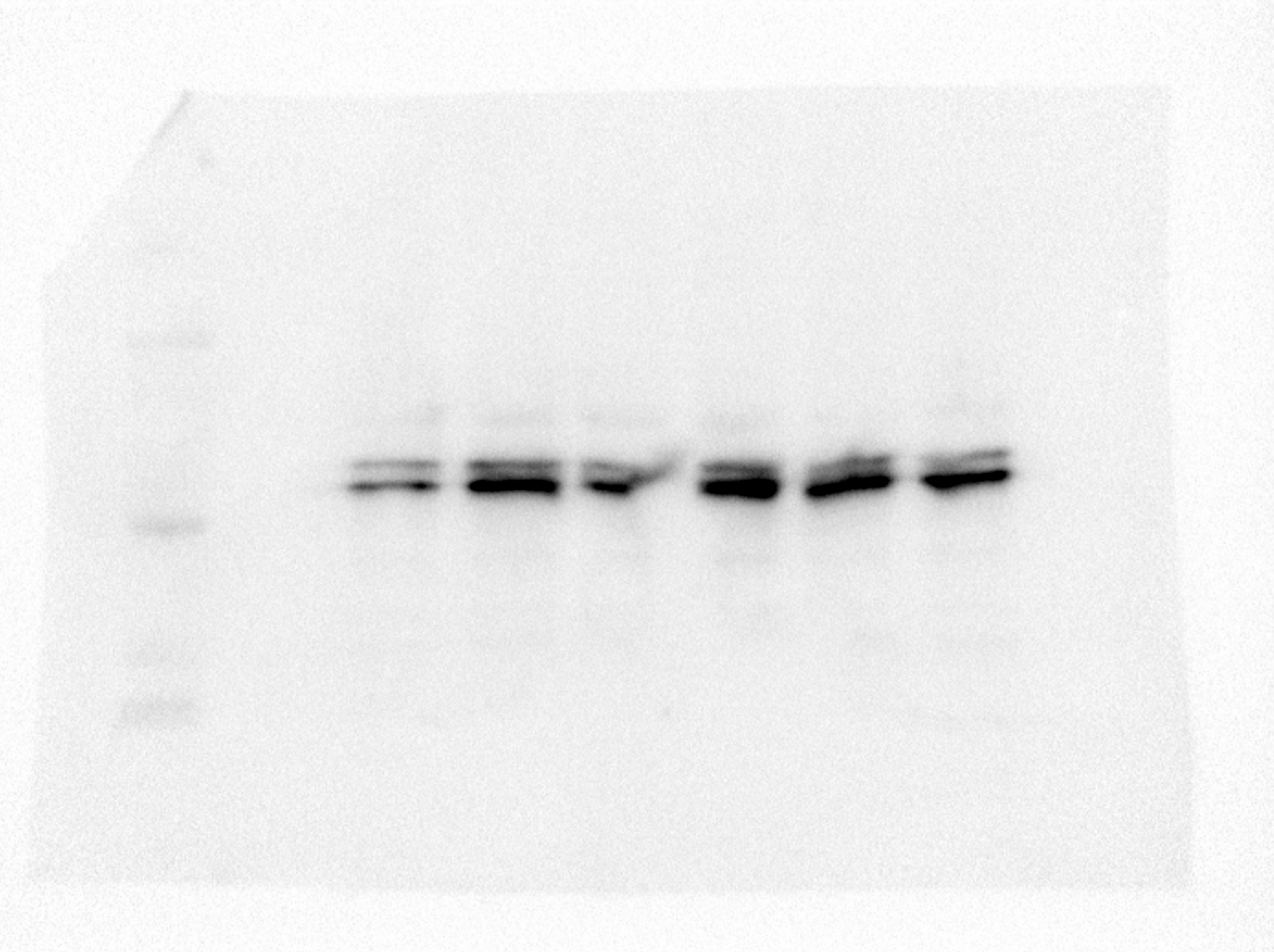

Supplement: Supplementary file 9 — Appendix. Fig. S1-10. [file 44318_2024_252_MOESM9_ESM.zip › Appendix. Fig. S1-10/Appendix. Fig. S8/S8 C/MAPK.tif]

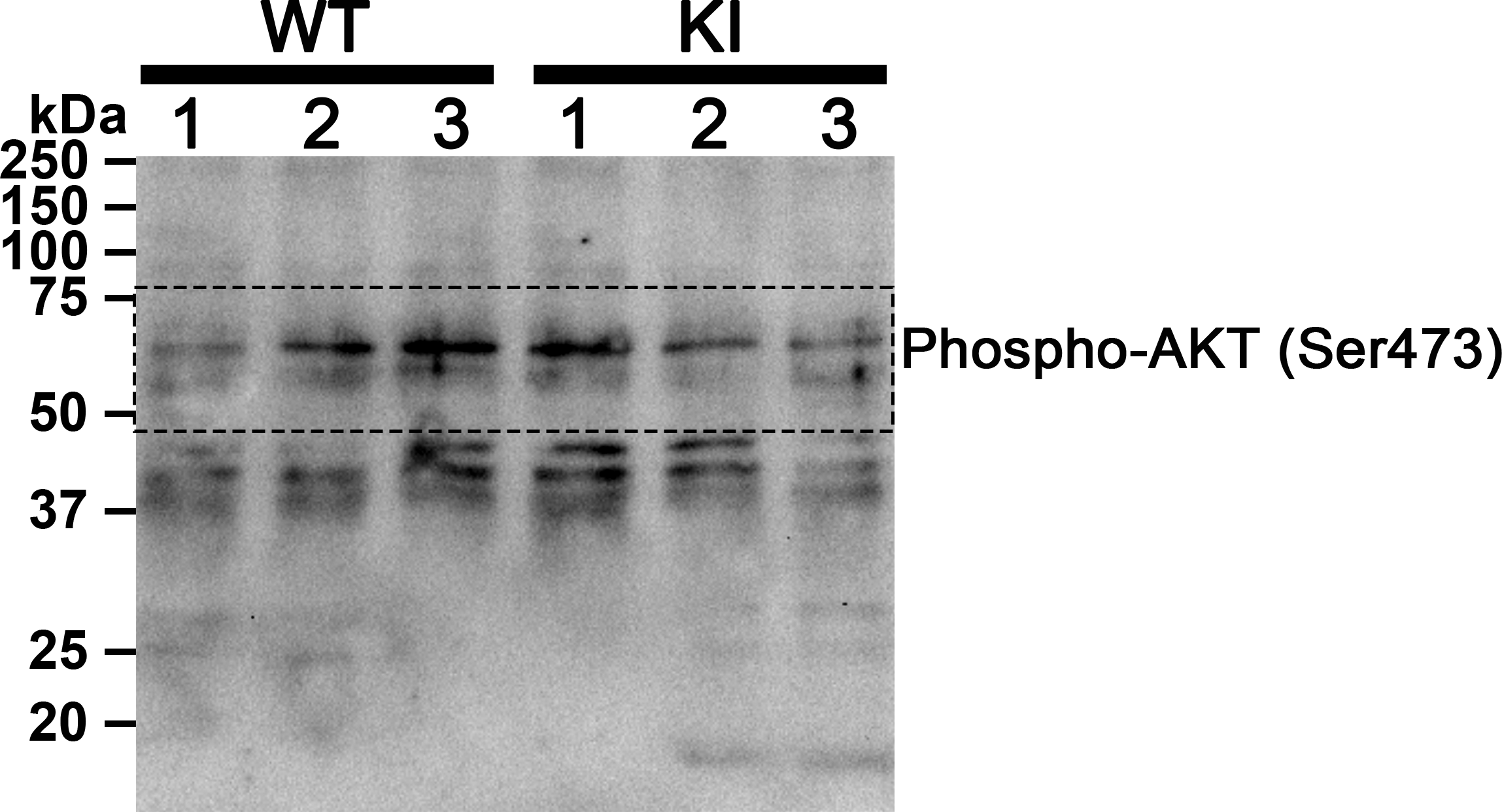

Supplement: Supplementary file 9 — Appendix. Fig. S1-10. [file 44318_2024_252_MOESM9_ESM.zip › Appendix. Fig. S1-10/Appendix. Fig. S8/S8 C/Phospho-AKT annotated.png]

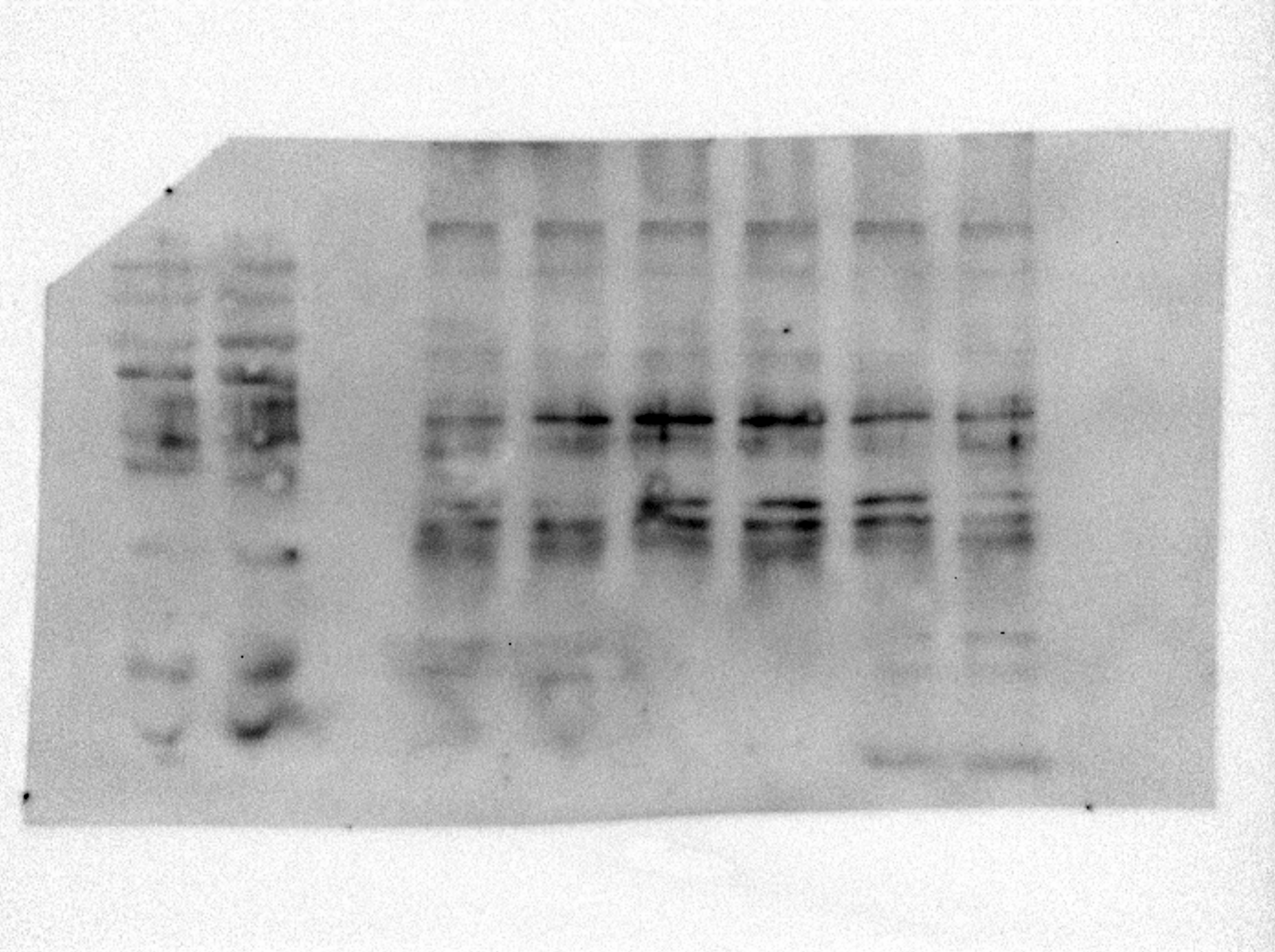

Supplement: Supplementary file 9 — Appendix. Fig. S1-10. [file 44318_2024_252_MOESM9_ESM.zip › Appendix. Fig. S1-10/Appendix. Fig. S8/S8 C/Phospho-AKT.tif]

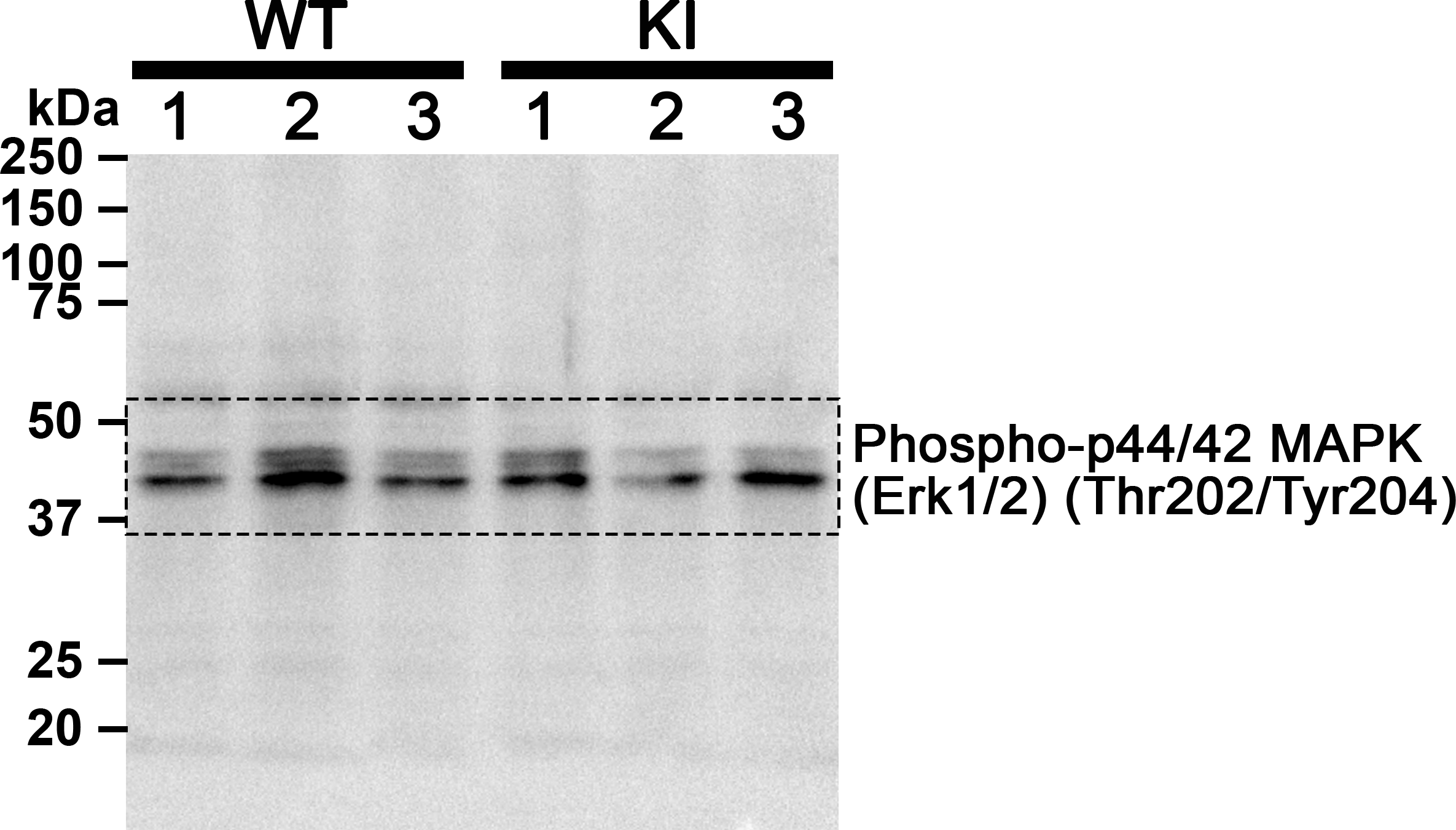

Supplement: Supplementary file 9 — Appendix. Fig. S1-10. [file 44318_2024_252_MOESM9_ESM.zip › Appendix. Fig. S1-10/Appendix. Fig. S8/S8 C/Phospho-MAPK annotated.png]

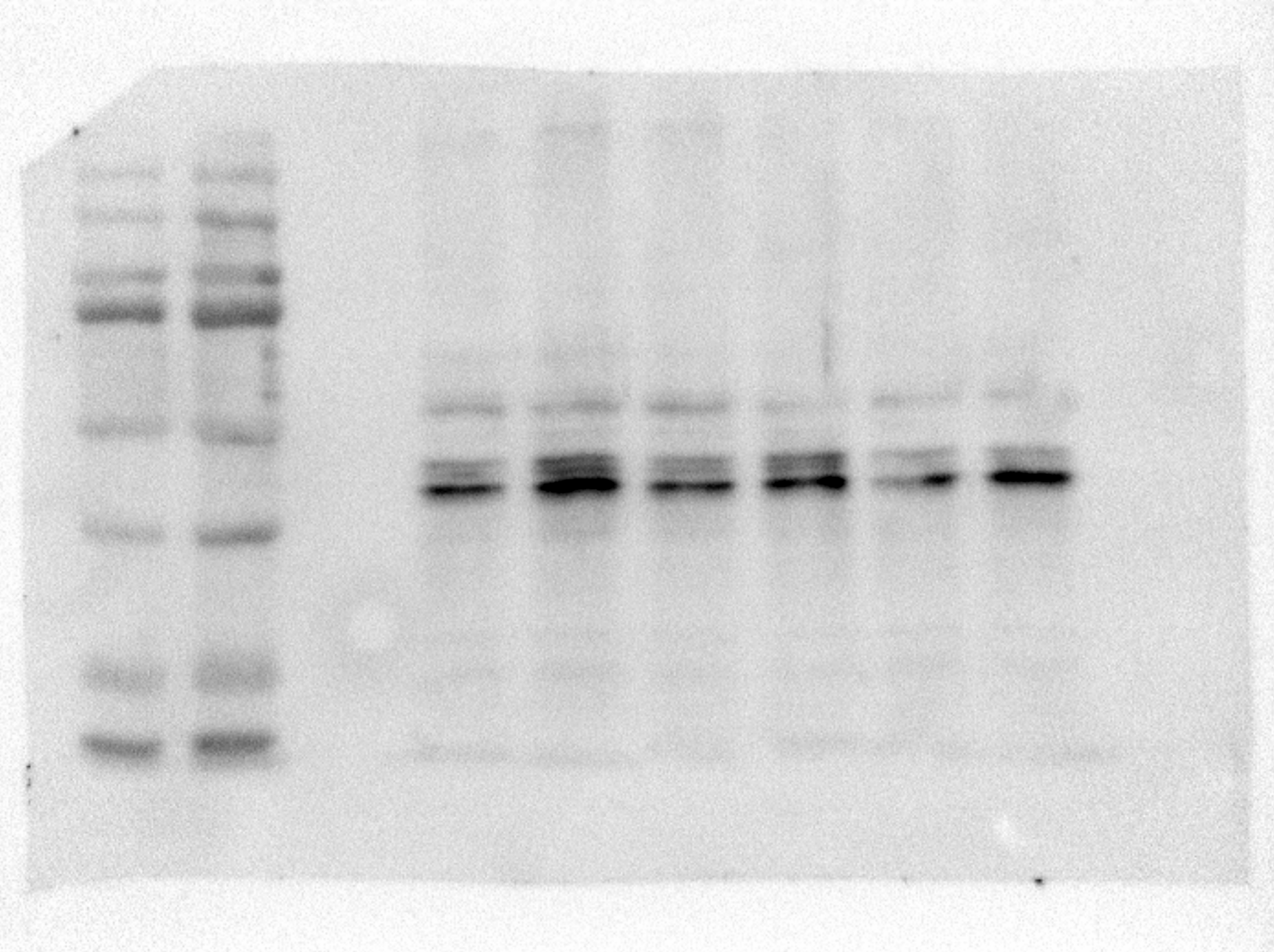

Supplement: Supplementary file 9 — Appendix. Fig. S1-10. [file 44318_2024_252_MOESM9_ESM.zip › Appendix. Fig. S1-10/Appendix. Fig. S8/S8 C/Phospho-MAPK.tif]

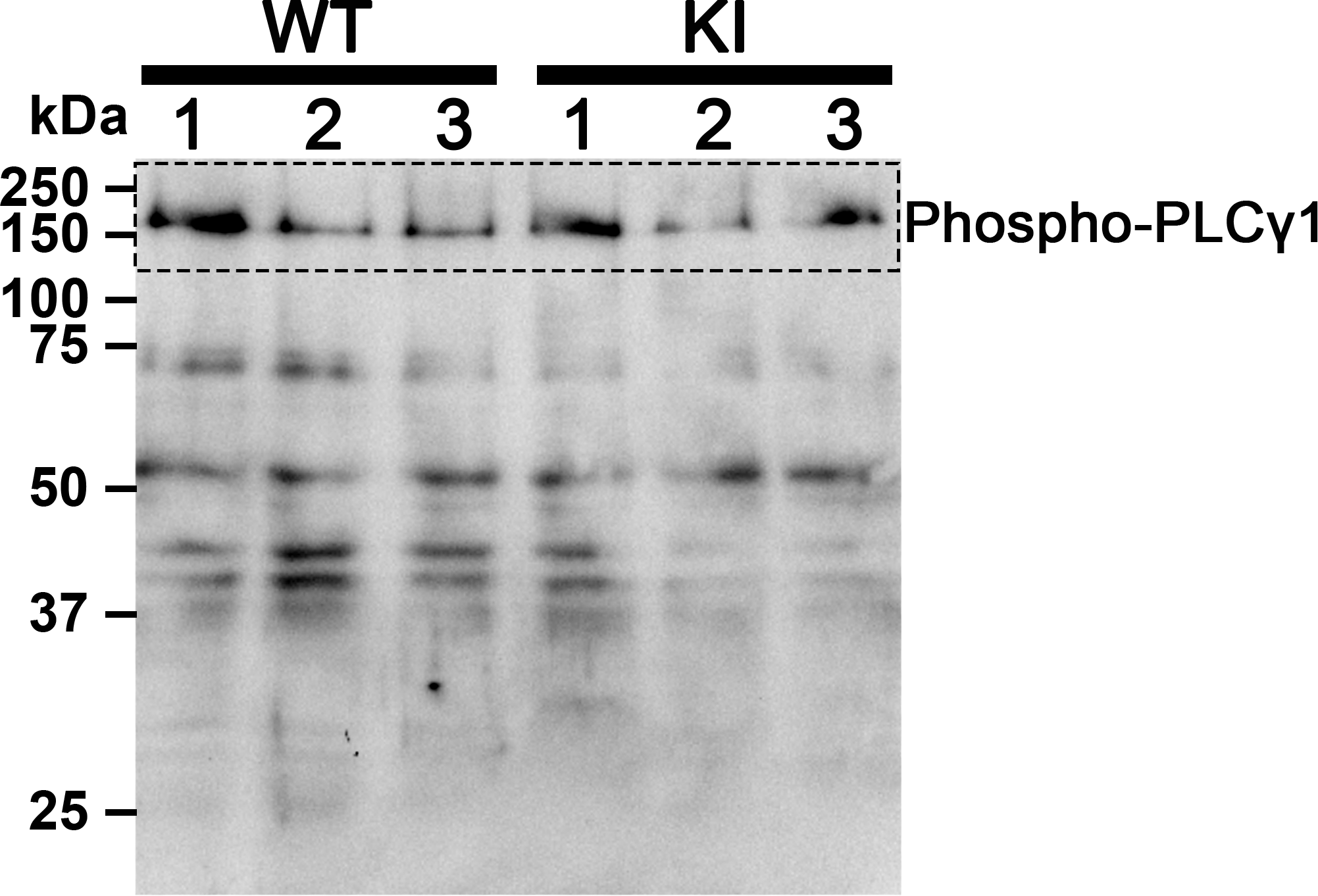

Supplement: Supplementary file 9 — Appendix. Fig. S1-10. [file 44318_2024_252_MOESM9_ESM.zip › Appendix. Fig. S1-10/Appendix. Fig. S8/S8 C/Phospho-PLCgamma annotated.png]

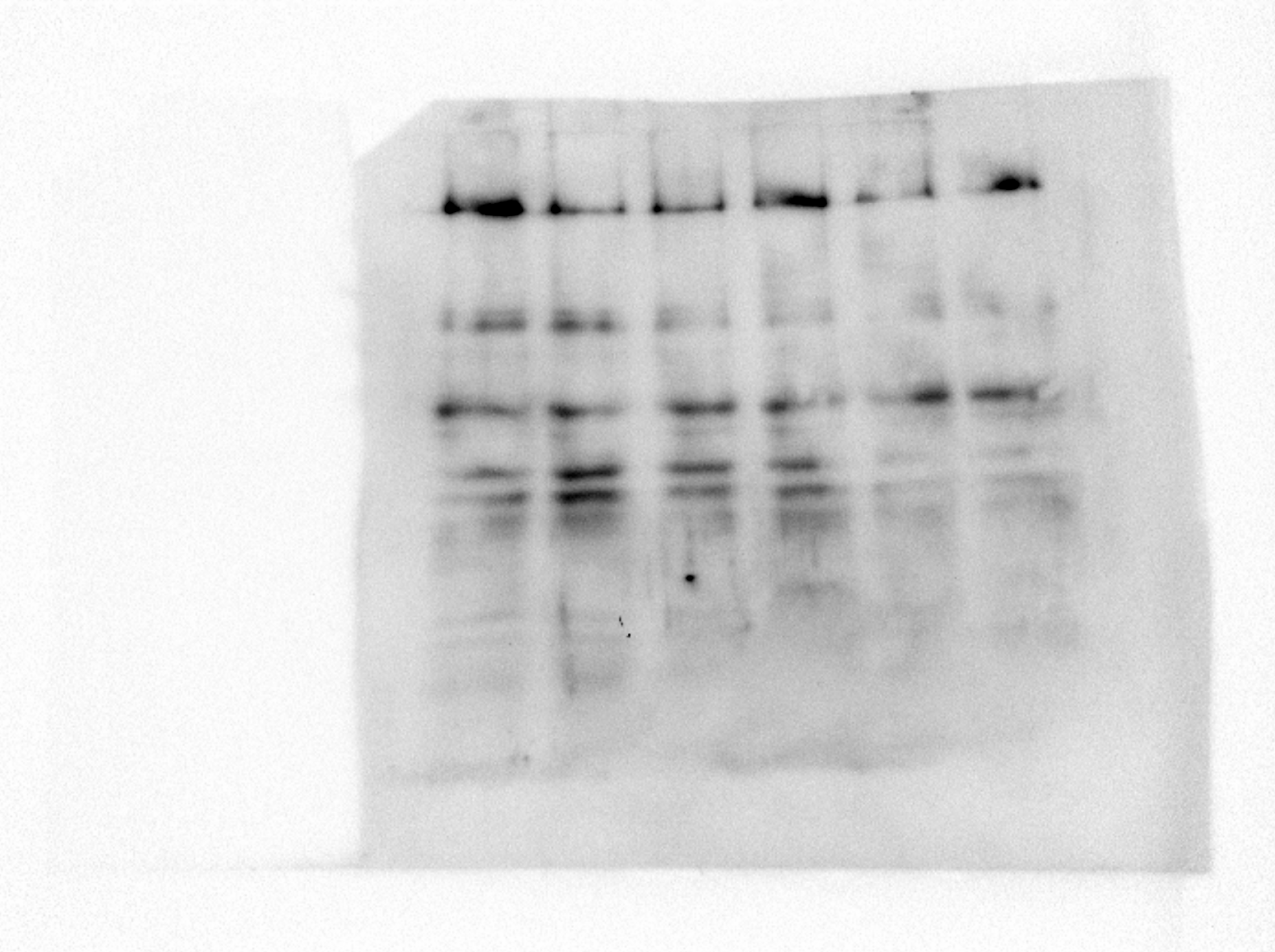

Supplement: Supplementary file 9 — Appendix. Fig. S1-10. [file 44318_2024_252_MOESM9_ESM.zip › Appendix. Fig. S1-10/Appendix. Fig. S8/S8 C/phospho-PLCgamma.tif]

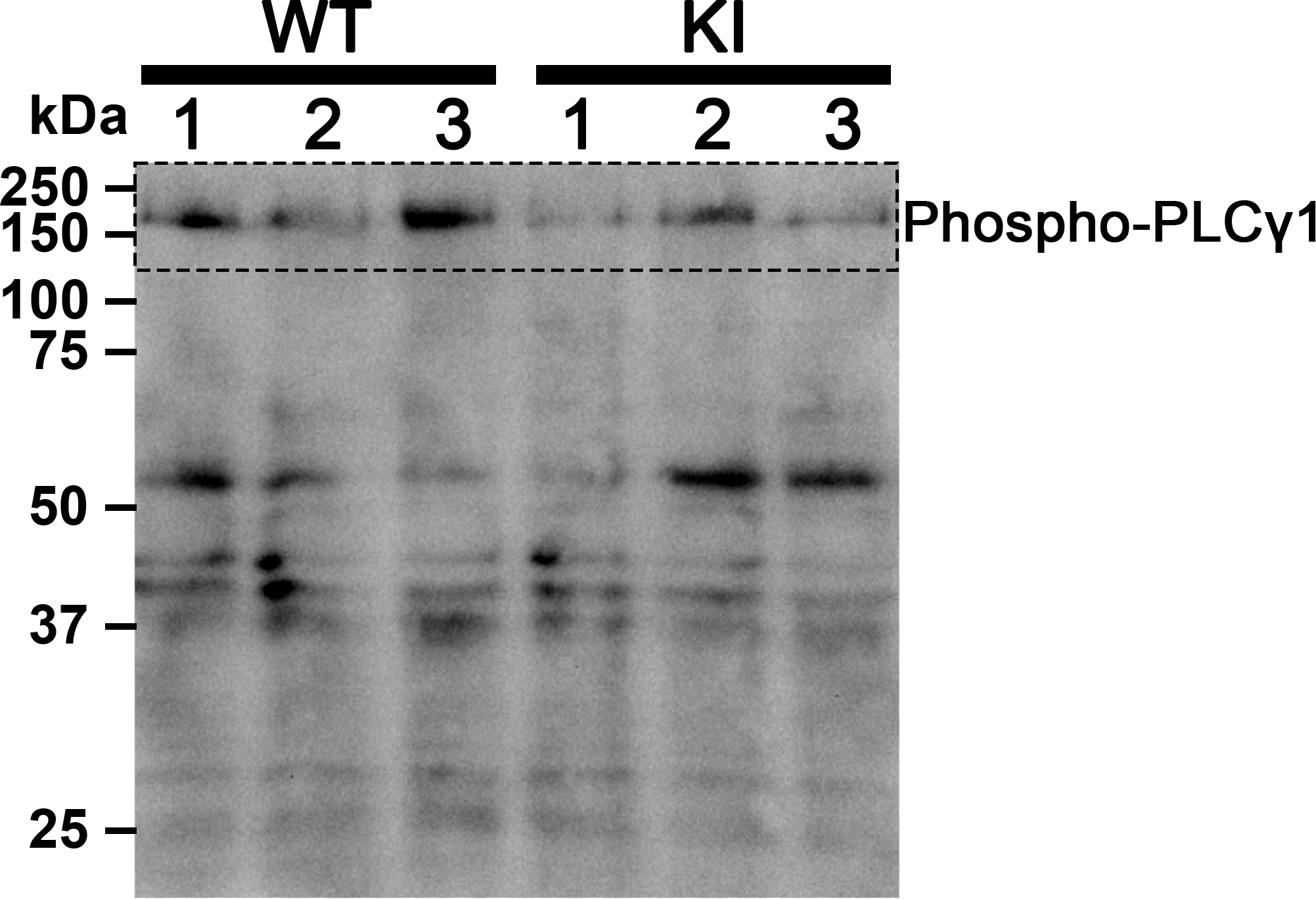

Supplement: Supplementary file 9 — Appendix. Fig. S1-10. [file 44318_2024_252_MOESM9_ESM.zip › Appendix. Fig. S1-10/Appendix. Fig. S8/S8 C/PLCgamma annotated.png]

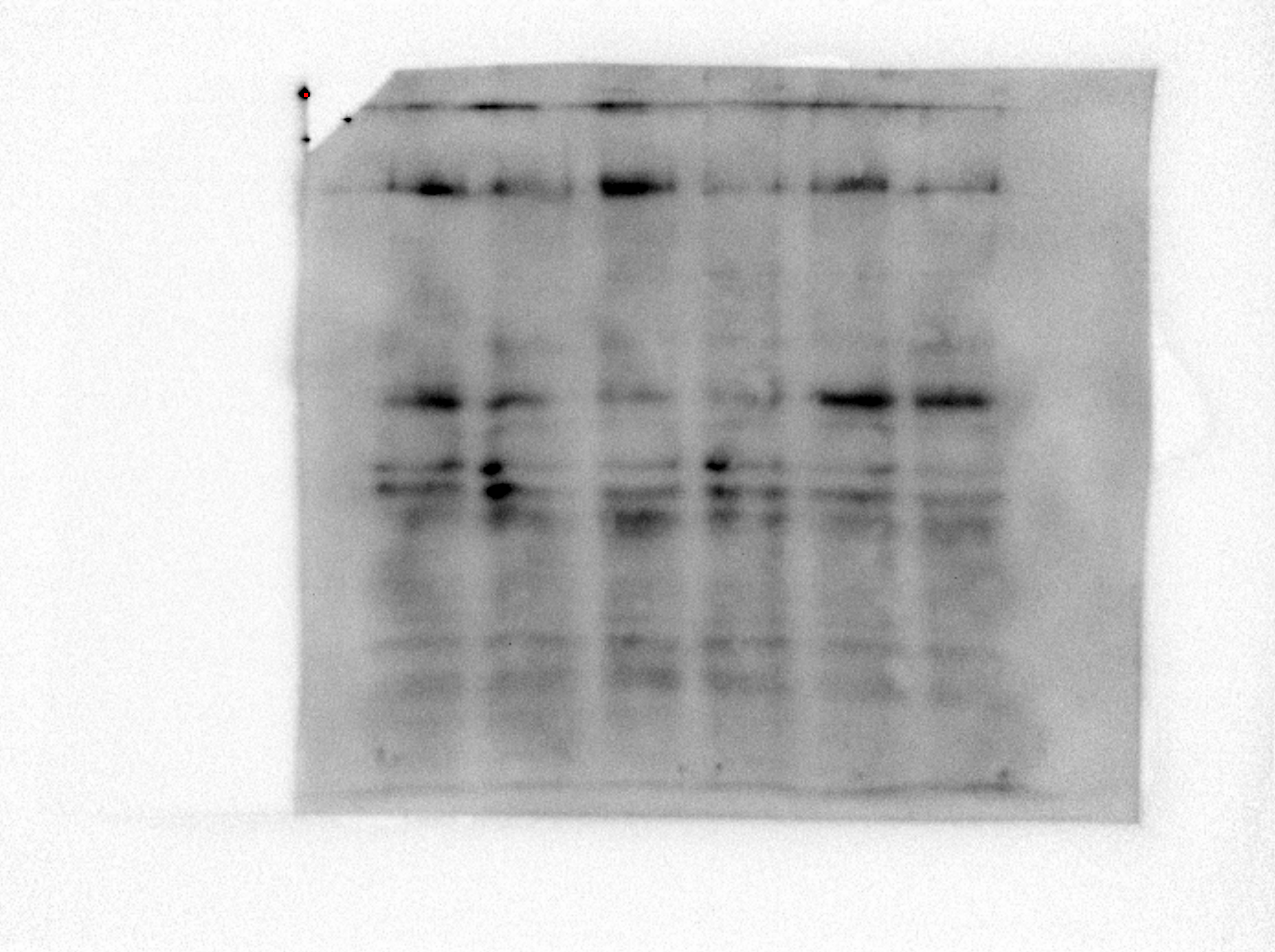

Supplement: Supplementary file 9 — Appendix. Fig. S1-10. [file 44318_2024_252_MOESM9_ESM.zip › Appendix. Fig. S1-10/Appendix. Fig. S8/S8 C/PLCgamma.tif]

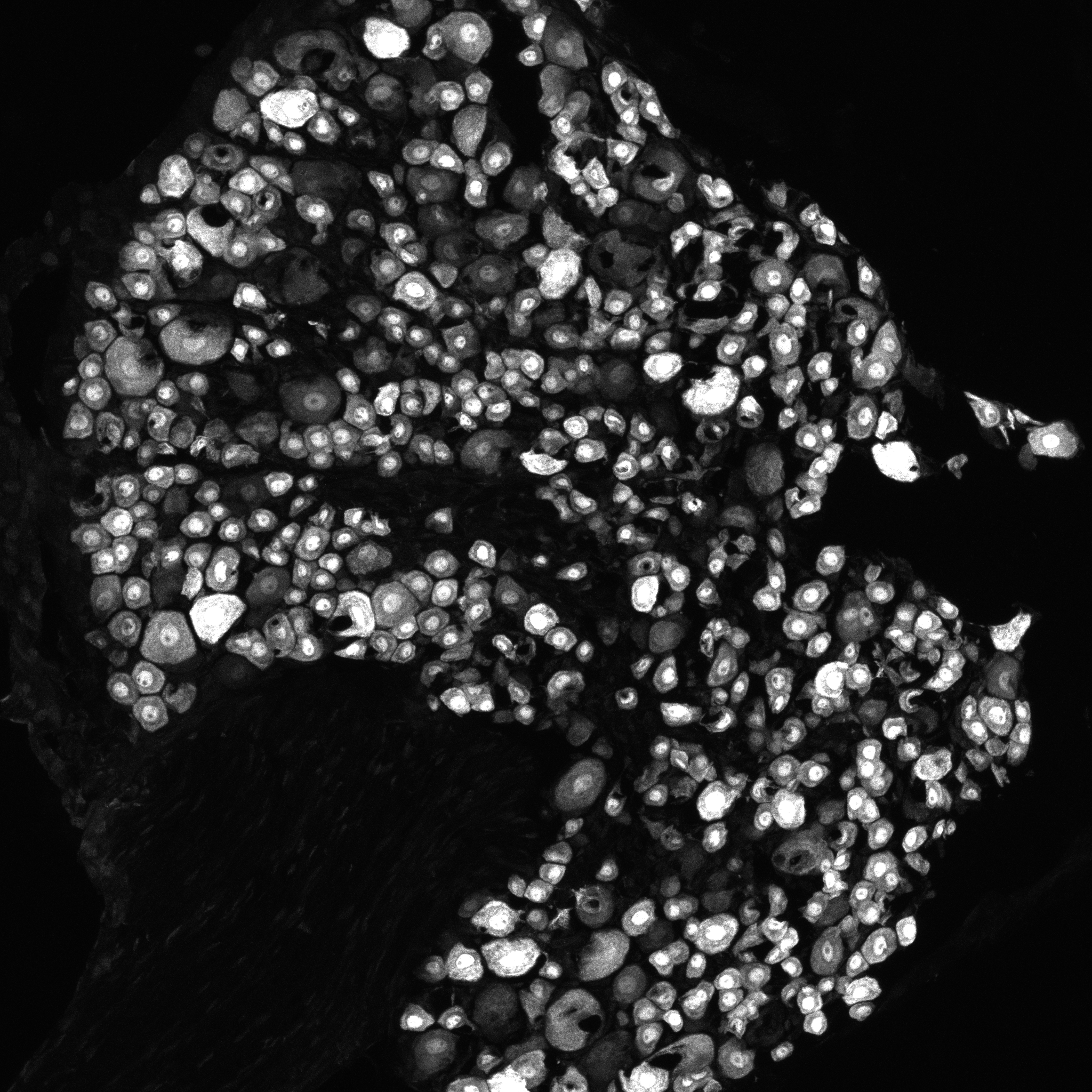

Supplement: Supplementary file 9 — Appendix. Fig. S1-10. [file 44318_2024_252_MOESM9_ESM.zip › Appendix. Fig. S1-10/Appendix. Fig. S9/S9 A/NeuN KI.tif]

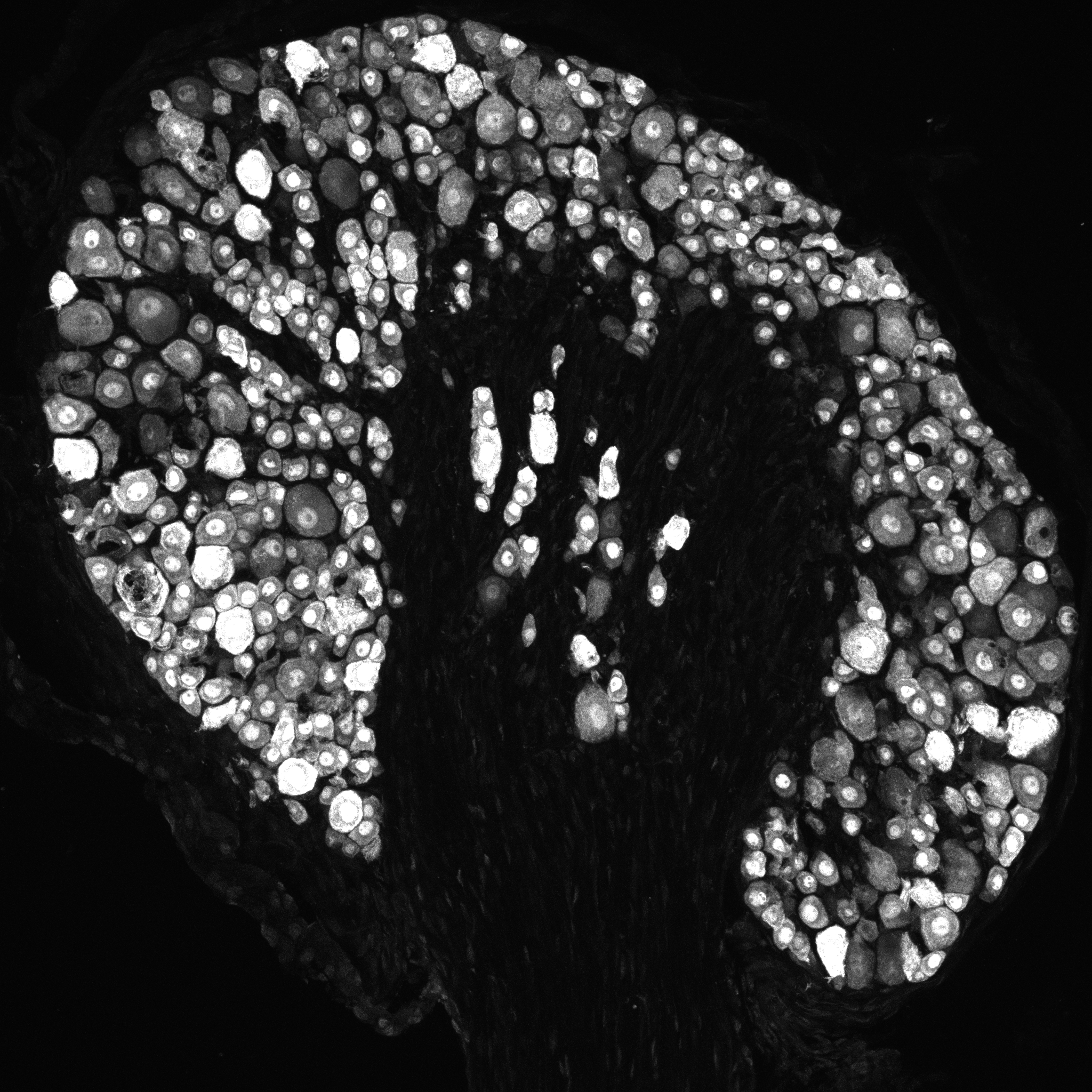

Supplement: Supplementary file 9 — Appendix. Fig. S1-10. [file 44318_2024_252_MOESM9_ESM.zip › Appendix. Fig. S1-10/Appendix. Fig. S9/S9 A/NeuN WT.tif]

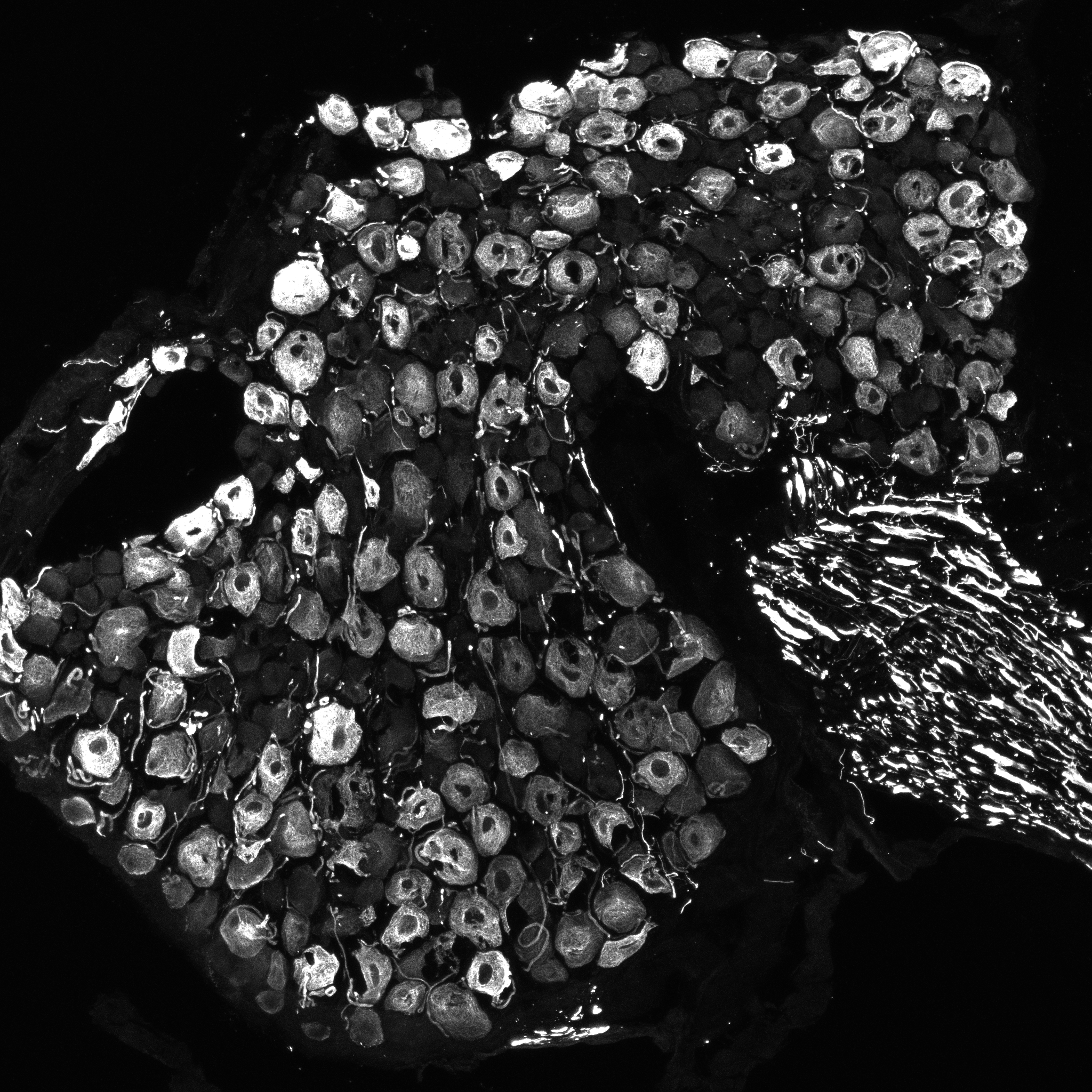

Supplement: Supplementary file 9 — Appendix. Fig. S1-10. [file 44318_2024_252_MOESM9_ESM.zip › Appendix. Fig. S1-10/Appendix. Fig. S9/S9 A/NF200 KI.tif]

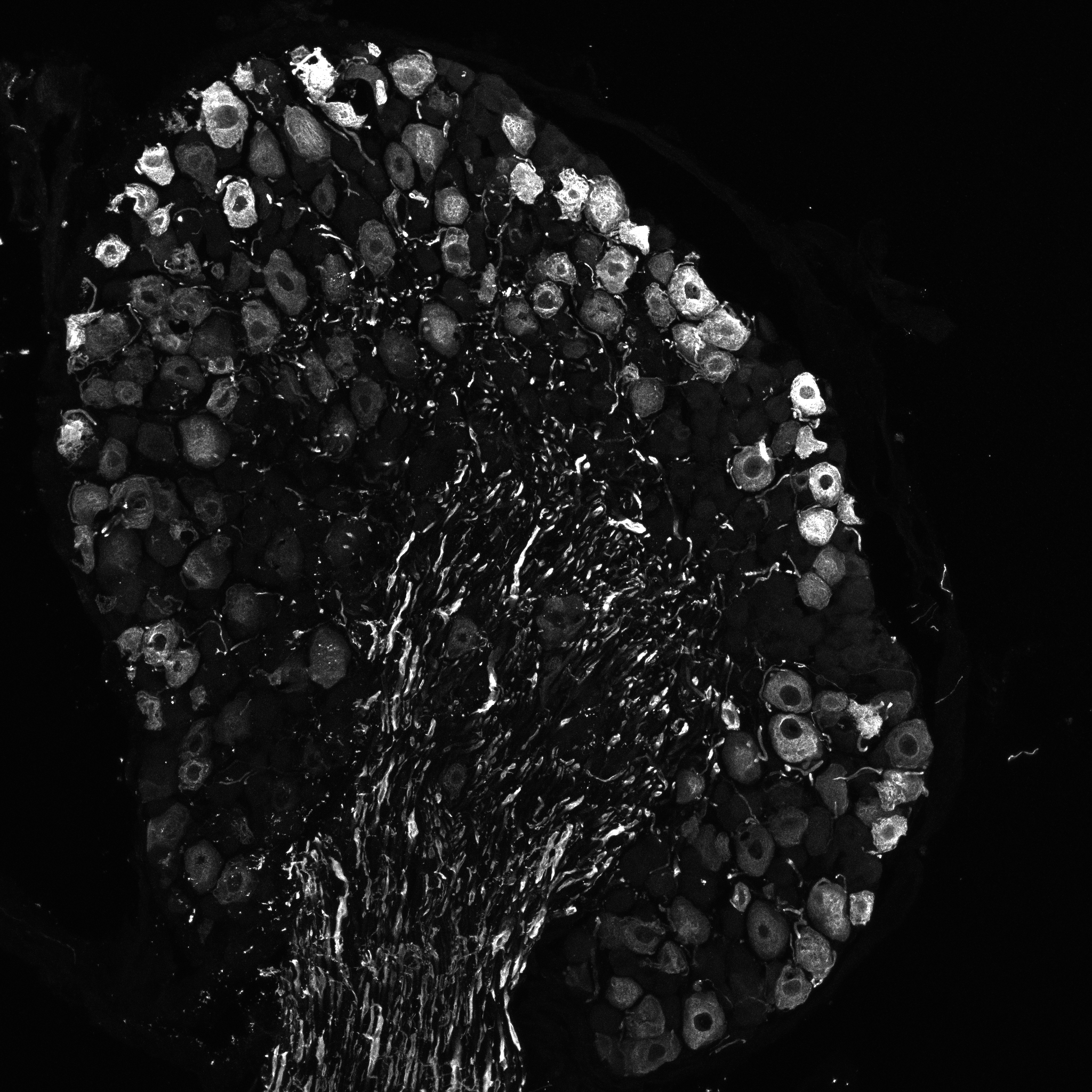

Supplement: Supplementary file 9 — Appendix. Fig. S1-10. [file 44318_2024_252_MOESM9_ESM.zip › Appendix. Fig. S1-10/Appendix. Fig. S9/S9 A/NF200 WT.tif]

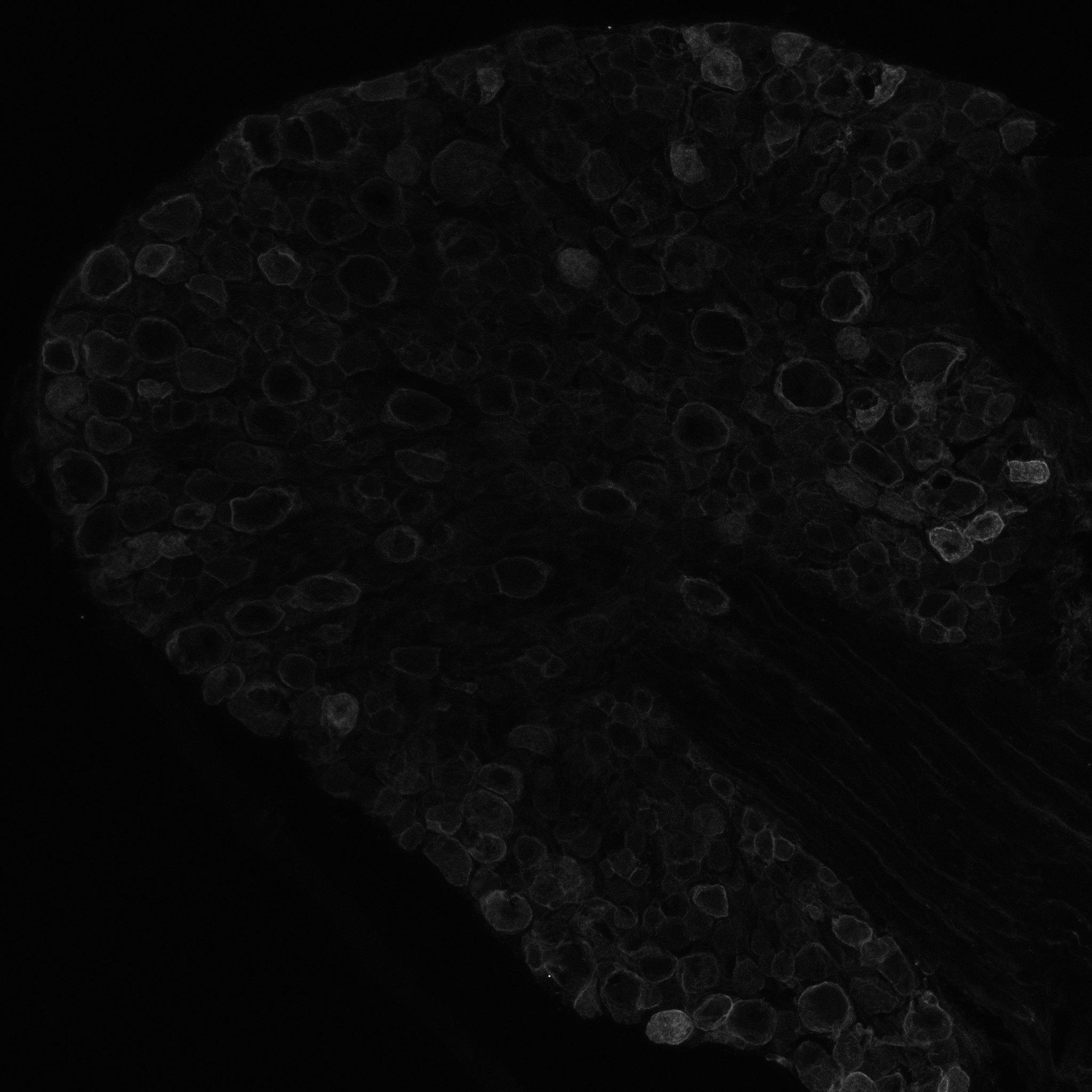

Supplement: Supplementary file 9 — Appendix. Fig. S1-10. [file 44318_2024_252_MOESM9_ESM.zip › Appendix. Fig. S1-10/Appendix. Fig. S9/S9 A/TrkB KI.tif]

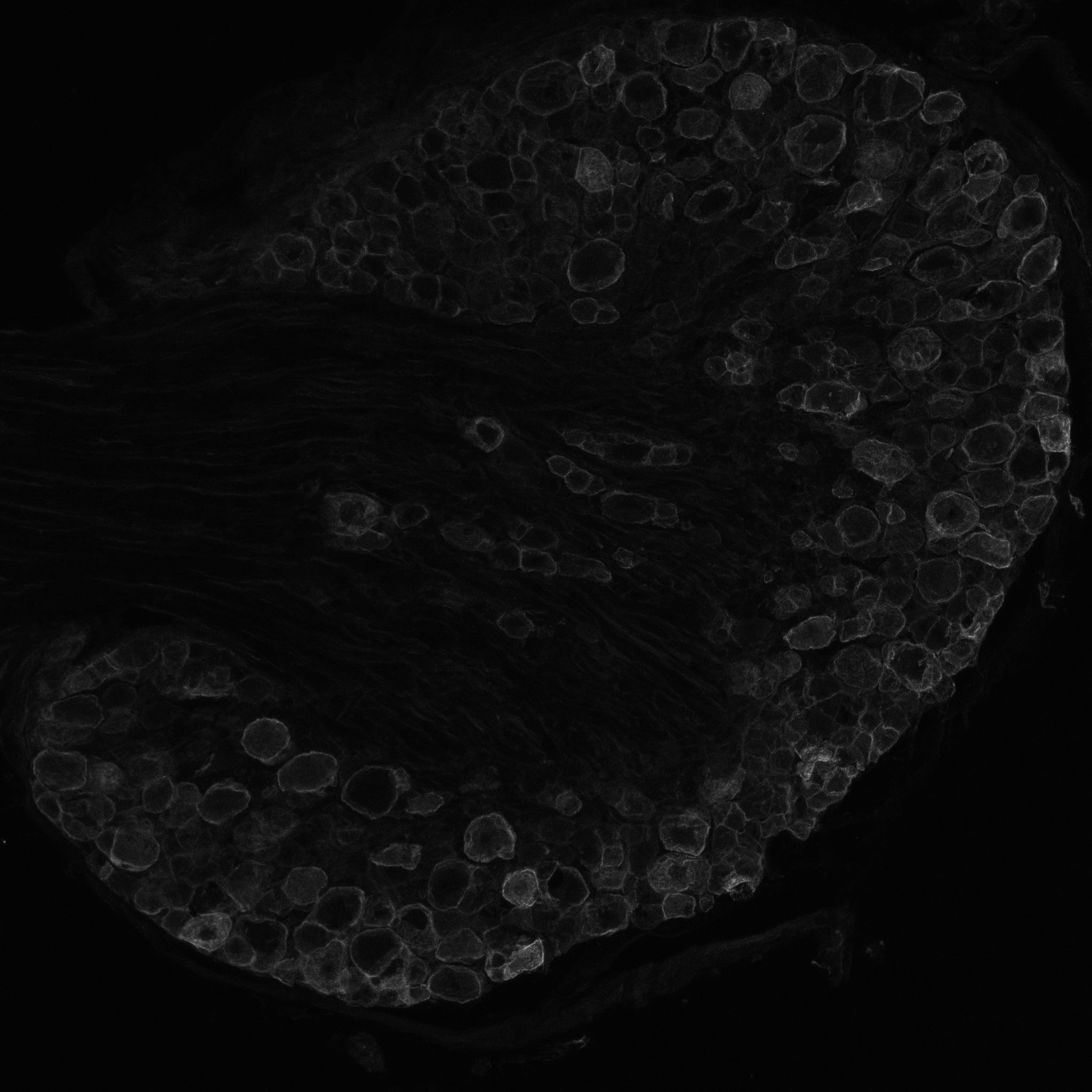

Supplement: Supplementary file 9 — Appendix. Fig. S1-10. [file 44318_2024_252_MOESM9_ESM.zip › Appendix. Fig. S1-10/Appendix. Fig. S9/S9 A/TrkB WT.tif]

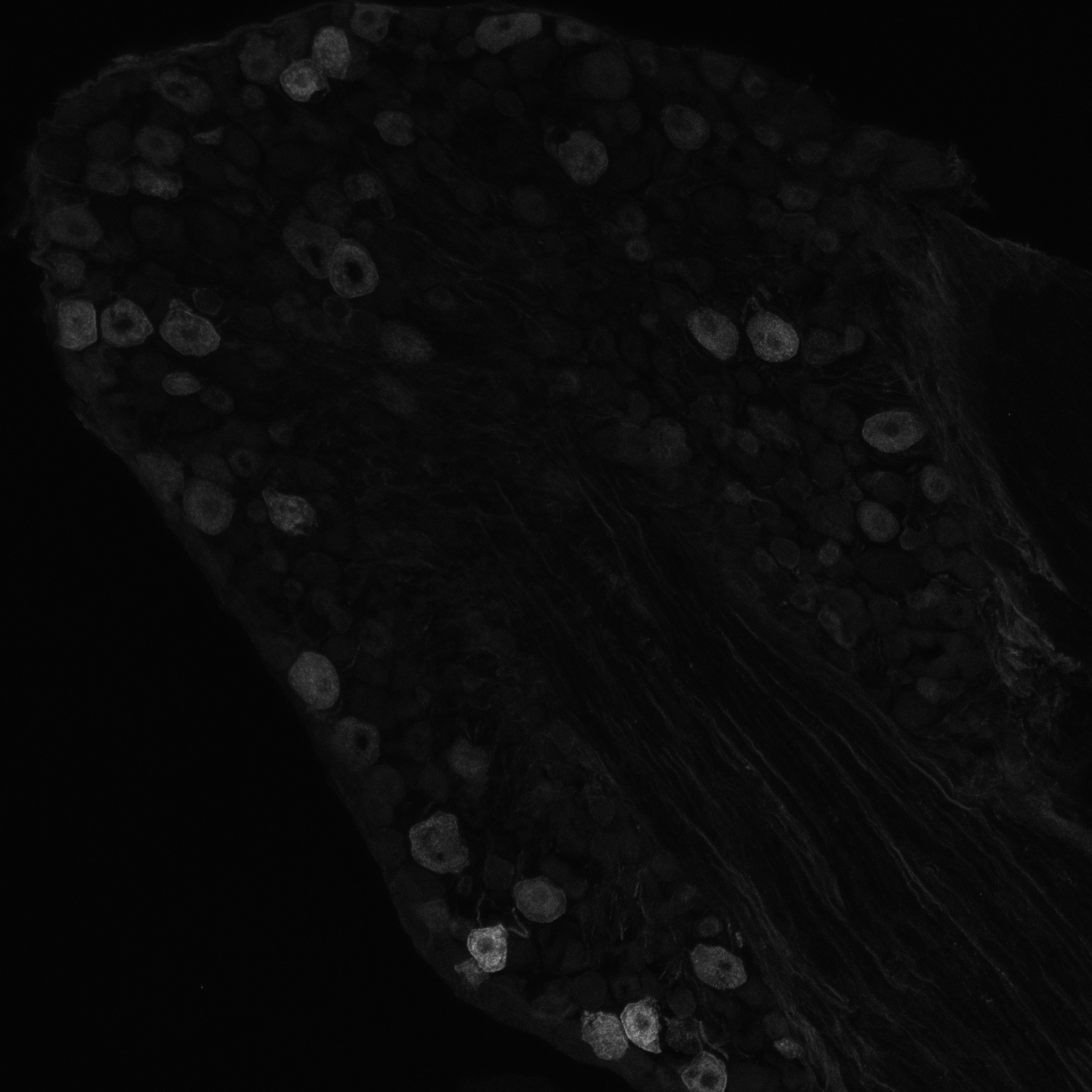

Supplement: Supplementary file 9 — Appendix. Fig. S1-10. [file 44318_2024_252_MOESM9_ESM.zip › Appendix. Fig. S1-10/Appendix. Fig. S9/S9 A/TrkC KI.tif]

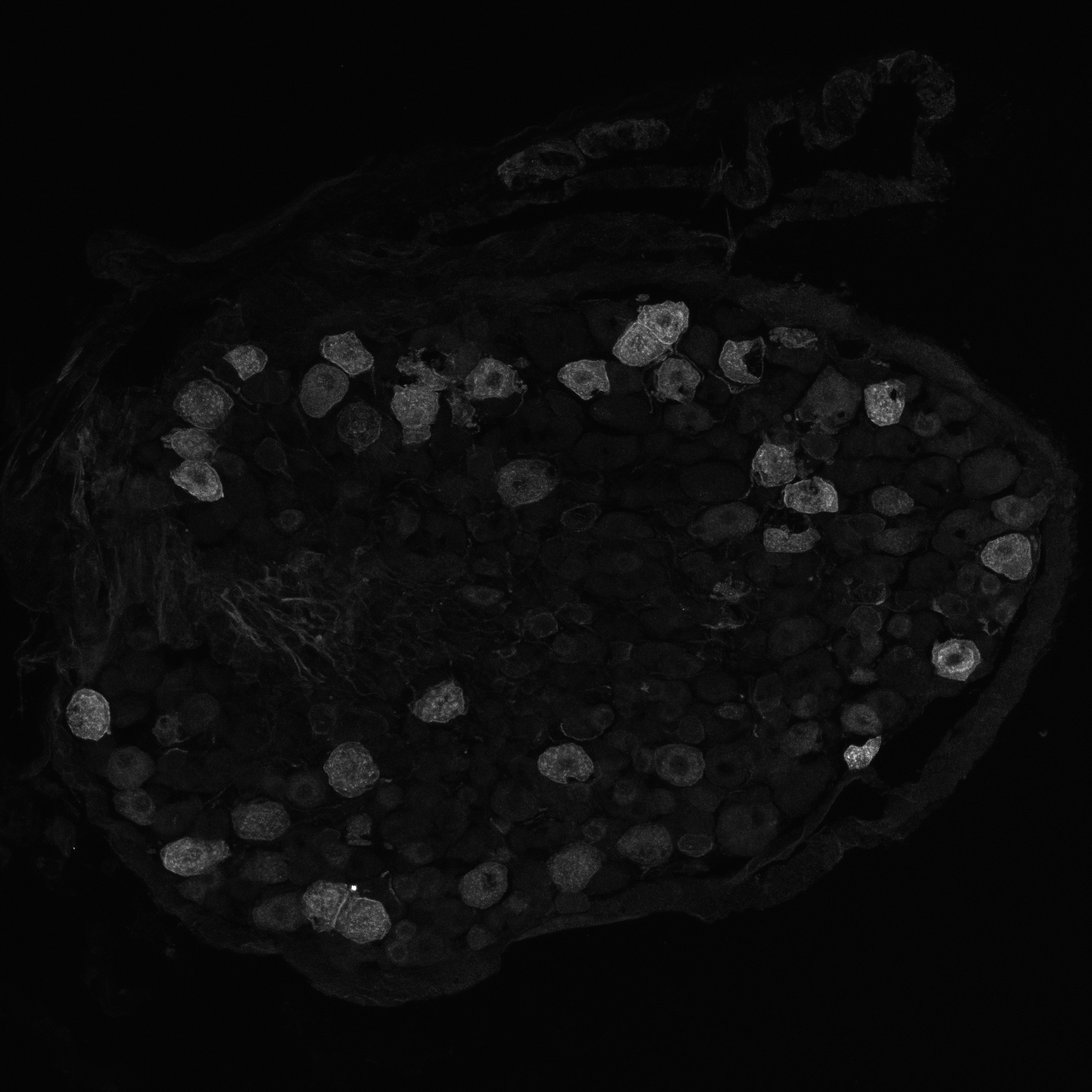

Supplement: Supplementary file 9 — Appendix. Fig. S1-10. [file 44318_2024_252_MOESM9_ESM.zip › Appendix. Fig. S1-10/Appendix. Fig. S9/S9 A/TrkC WT.tif]

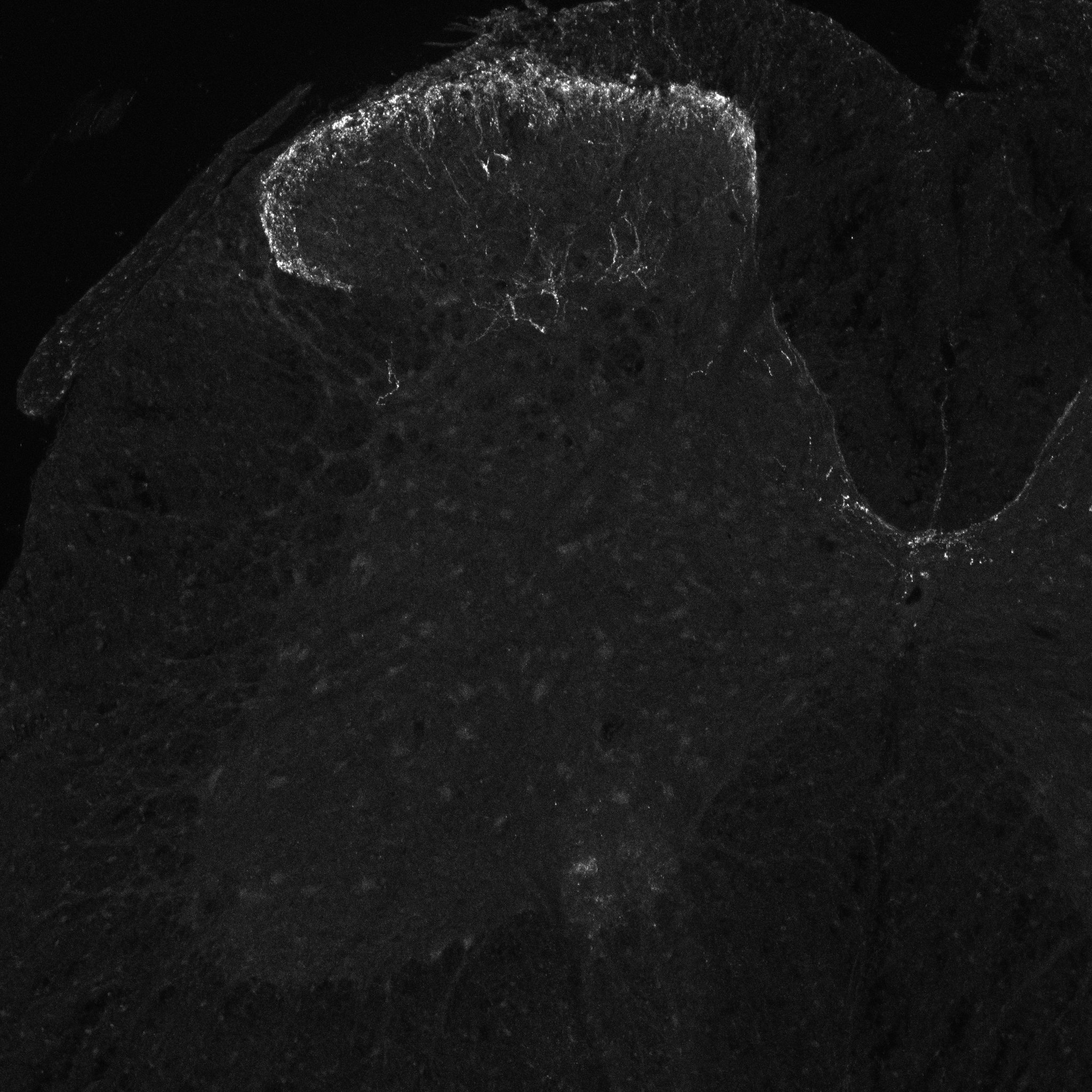

Supplement: Supplementary file 9 — Appendix. Fig. S1-10. [file 44318_2024_252_MOESM9_ESM.zip › Appendix. Fig. S1-10/Appendix. Fig. S9/S9 C/sup. Figure C KI CGRP.tif]

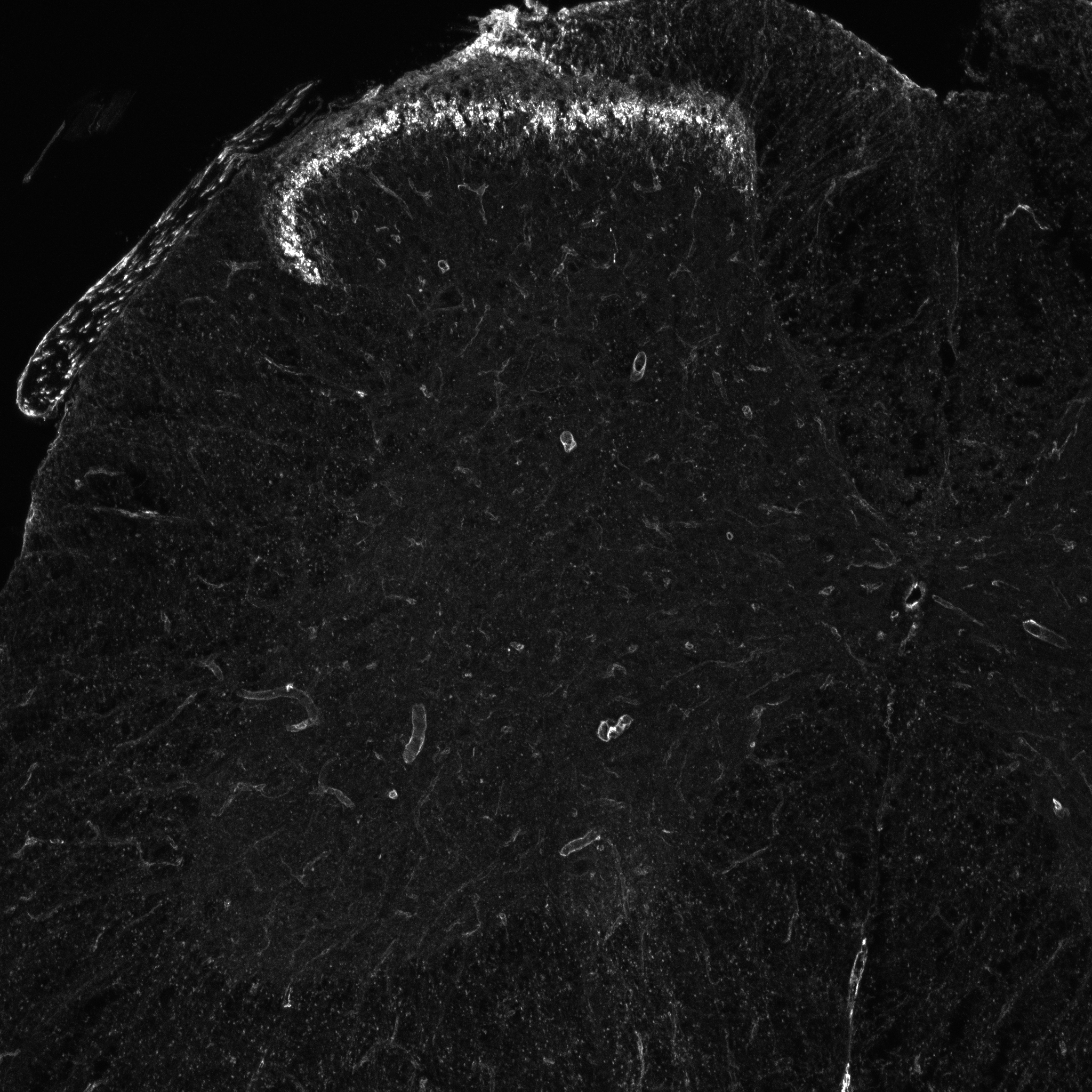

Supplement: Supplementary file 9 — Appendix. Fig. S1-10. [file 44318_2024_252_MOESM9_ESM.zip › Appendix. Fig. S1-10/Appendix. Fig. S9/S9 C/sup. Figure C KI IB4.tif]

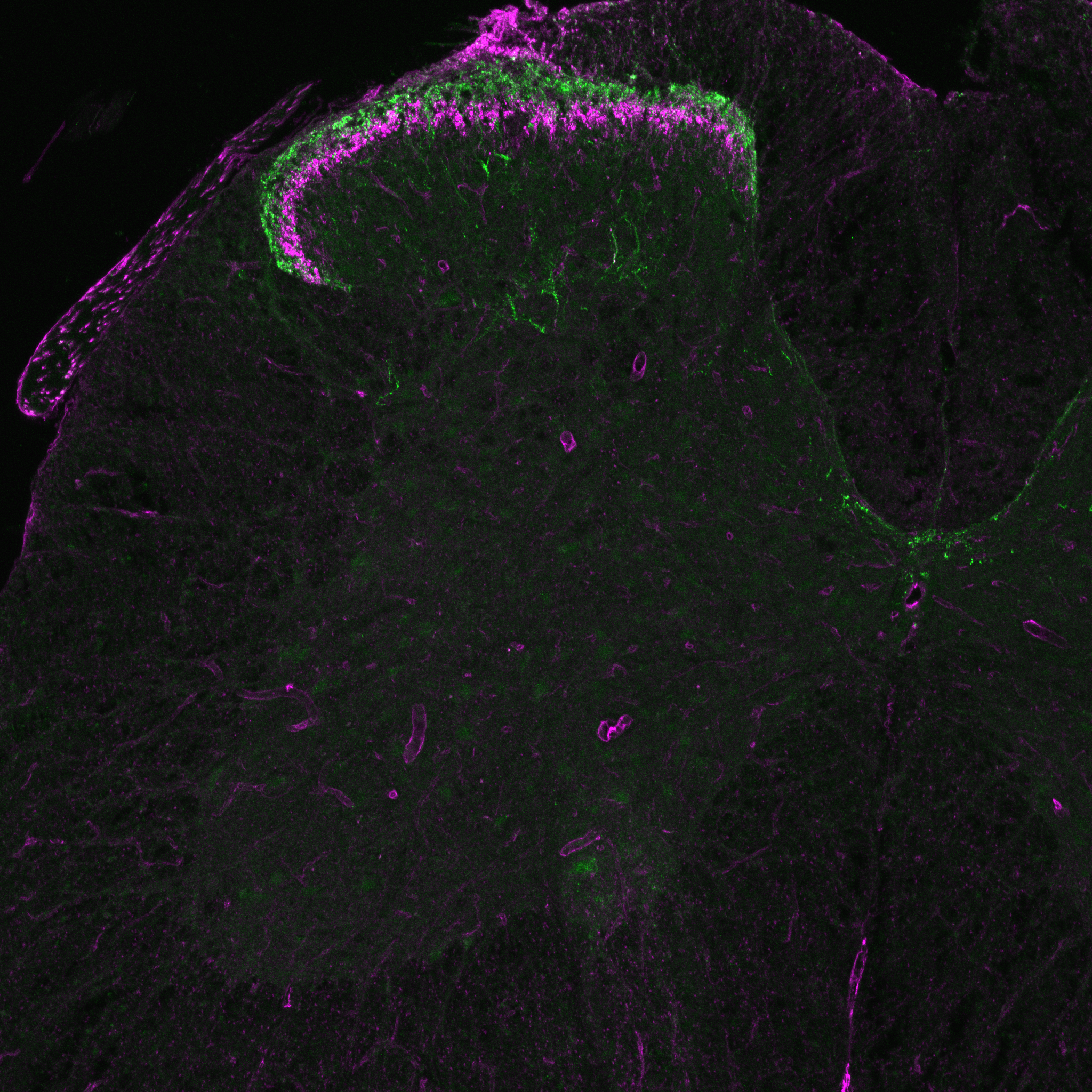

Supplement: Supplementary file 9 — Appendix. Fig. S1-10. [file 44318_2024_252_MOESM9_ESM.zip › Appendix. Fig. S1-10/Appendix. Fig. S9/S9 C/sup. Figure C KI IB4_CGRP merge.tif]

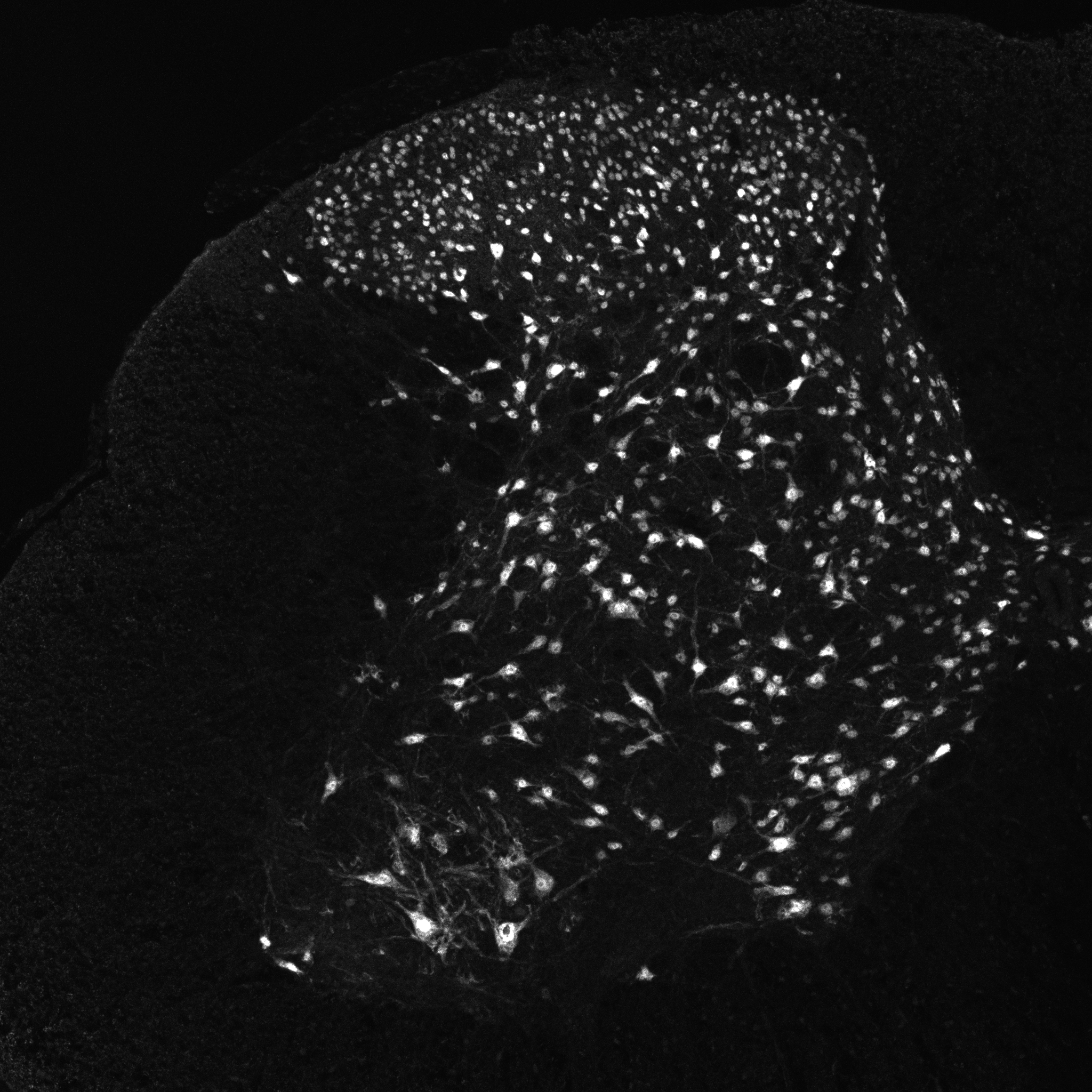

Supplement: Supplementary file 9 — Appendix. Fig. S1-10. [file 44318_2024_252_MOESM9_ESM.zip › Appendix. Fig. S1-10/Appendix. Fig. S9/S9 C/sup. Figure C KI NeuN.tif]

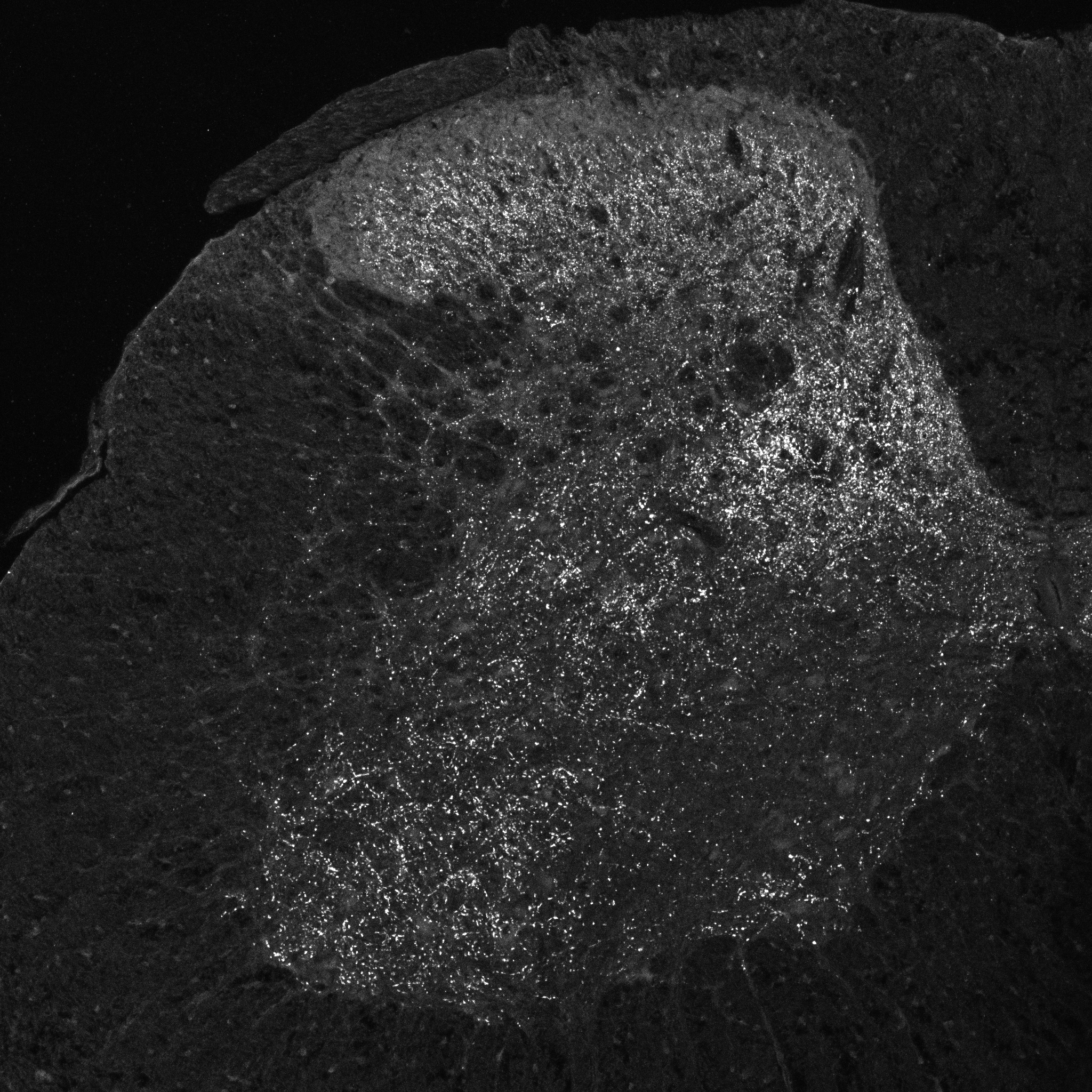

Supplement: Supplementary file 9 — Appendix. Fig. S1-10. [file 44318_2024_252_MOESM9_ESM.zip › Appendix. Fig. S1-10/Appendix. Fig. S9/S9 C/sup. Figure C KI VGLUT1.tif]

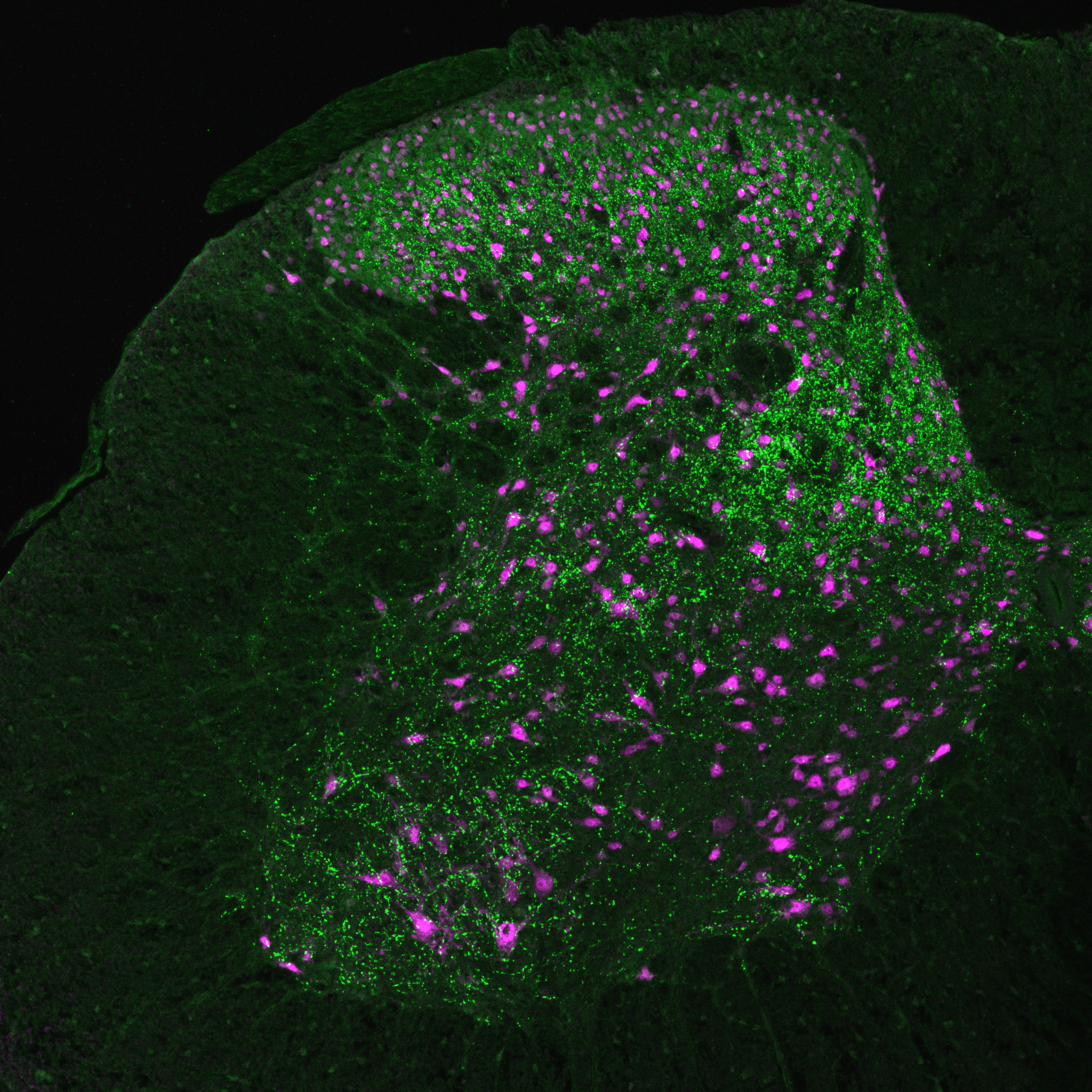

Supplement: Supplementary file 9 — Appendix. Fig. S1-10. [file 44318_2024_252_MOESM9_ESM.zip › Appendix. Fig. S1-10/Appendix. Fig. S9/S9 C/sup. Figure C KI VGLUT1_NeuN merge.tif]

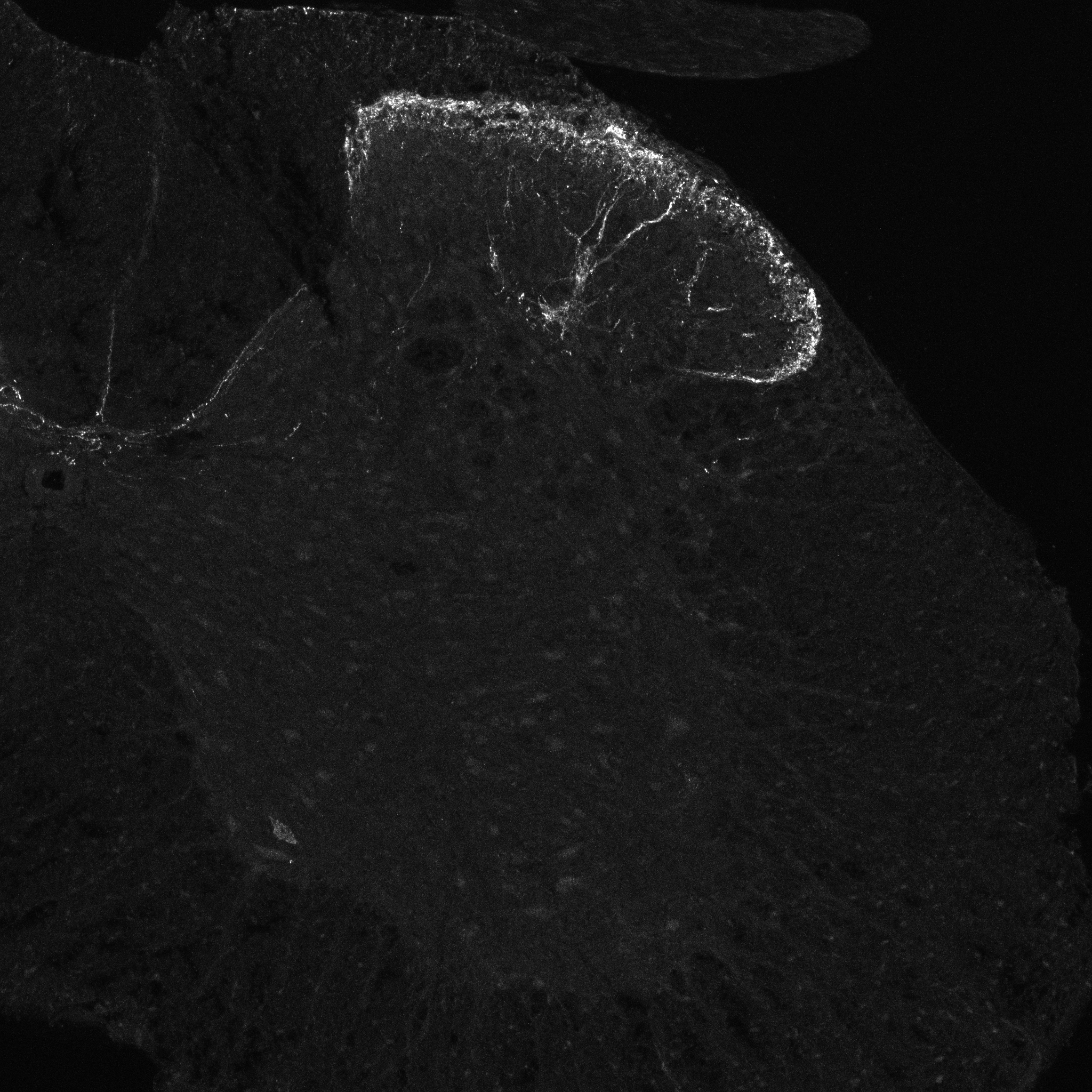

Supplement: Supplementary file 9 — Appendix. Fig. S1-10. [file 44318_2024_252_MOESM9_ESM.zip › Appendix. Fig. S1-10/Appendix. Fig. S9/S9 C/sup. Figure C WT CGRP.tif]

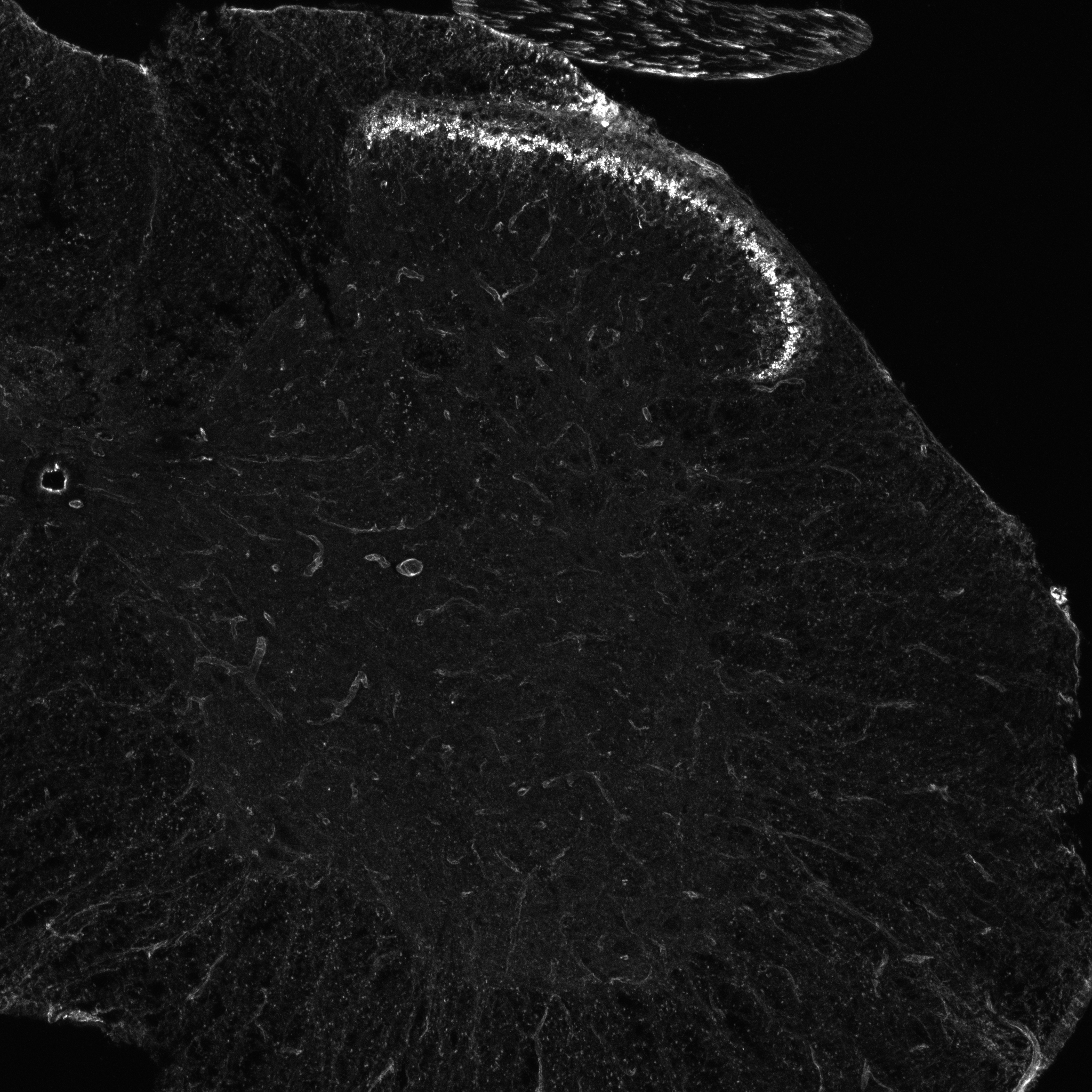

Supplement: Supplementary file 9 — Appendix. Fig. S1-10. [file 44318_2024_252_MOESM9_ESM.zip › Appendix. Fig. S1-10/Appendix. Fig. S9/S9 C/sup. Figure C WT IB4.tif]

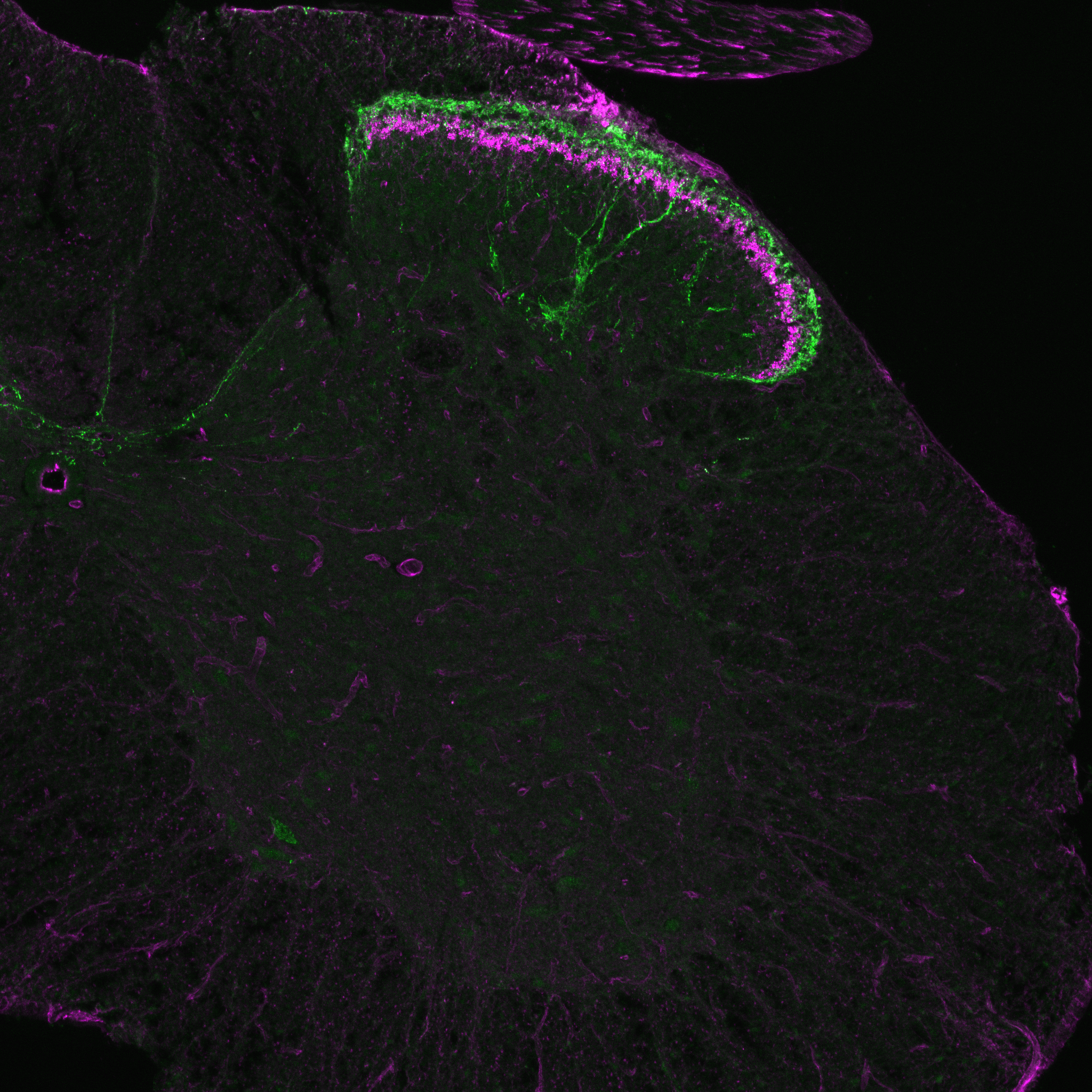

Supplement: Supplementary file 9 — Appendix. Fig. S1-10. [file 44318_2024_252_MOESM9_ESM.zip › Appendix. Fig. S1-10/Appendix. Fig. S9/S9 C/sup. Figure C WT IB4_CGRP merge.tif]

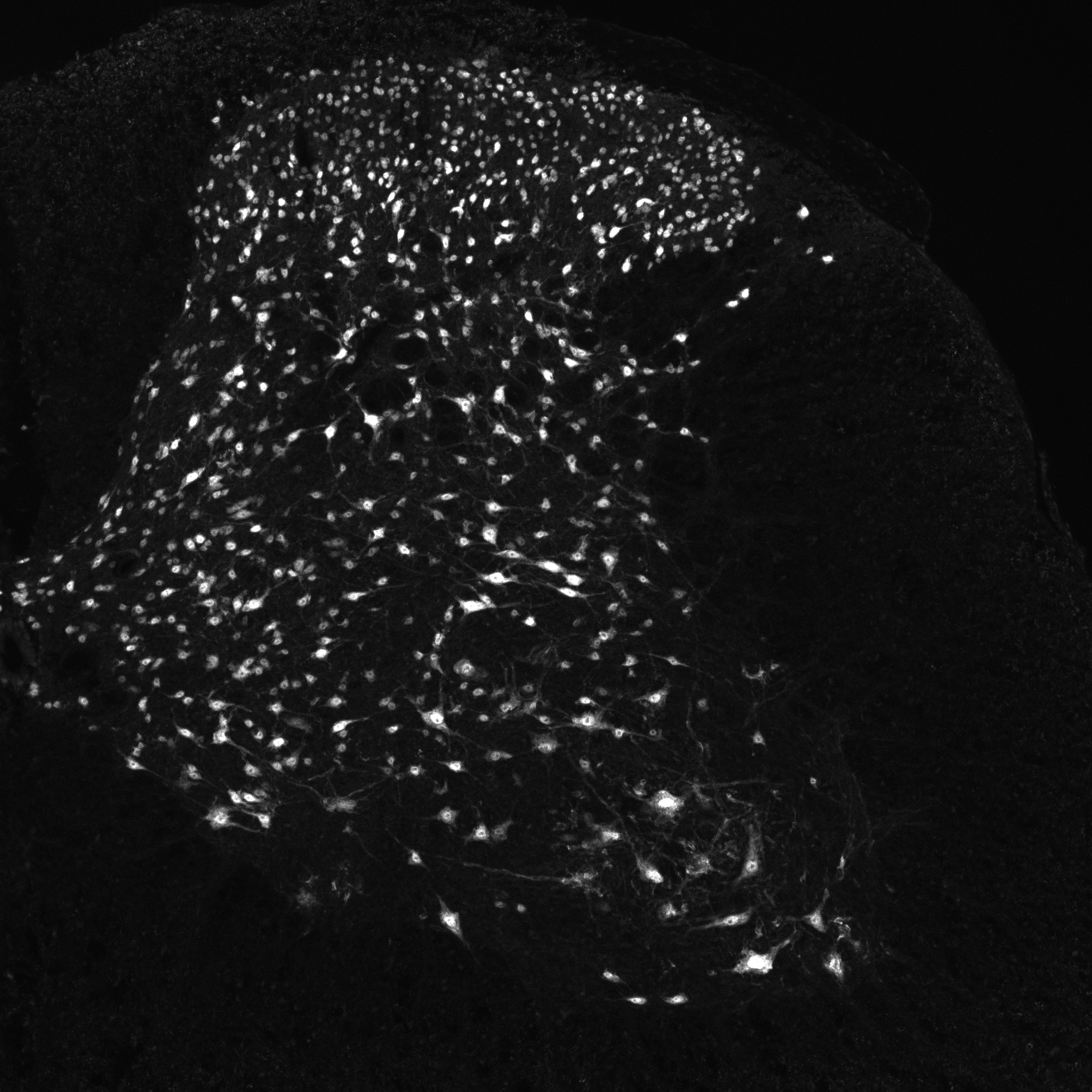

Supplement: Supplementary file 9 — Appendix. Fig. S1-10. [file 44318_2024_252_MOESM9_ESM.zip › Appendix. Fig. S1-10/Appendix. Fig. S9/S9 C/sup. Figure C WT NeuN.tif]

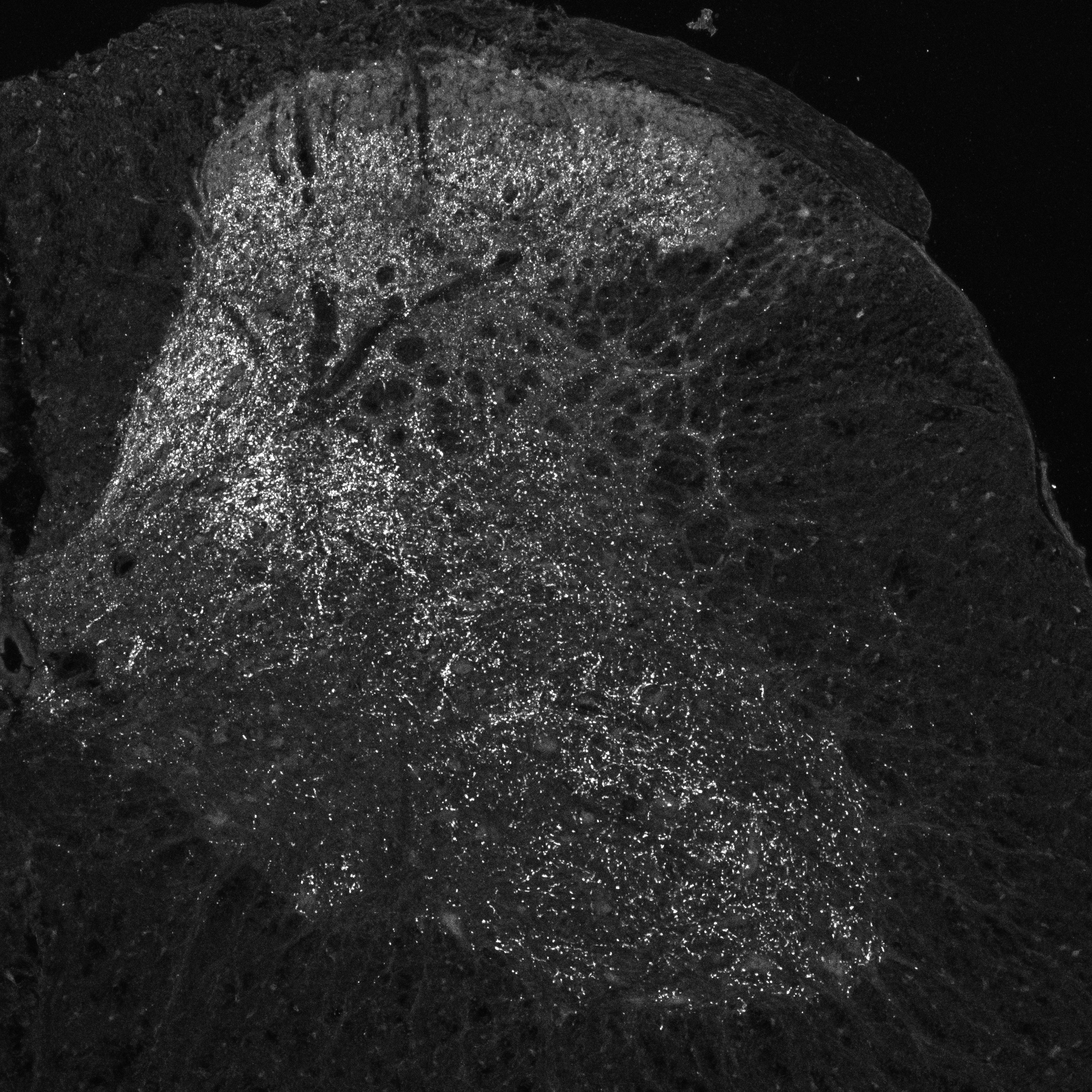

Supplement: Supplementary file 9 — Appendix. Fig. S1-10. [file 44318_2024_252_MOESM9_ESM.zip › Appendix. Fig. S1-10/Appendix. Fig. S9/S9 C/sup. Figure C WT VGLUT1.tif]

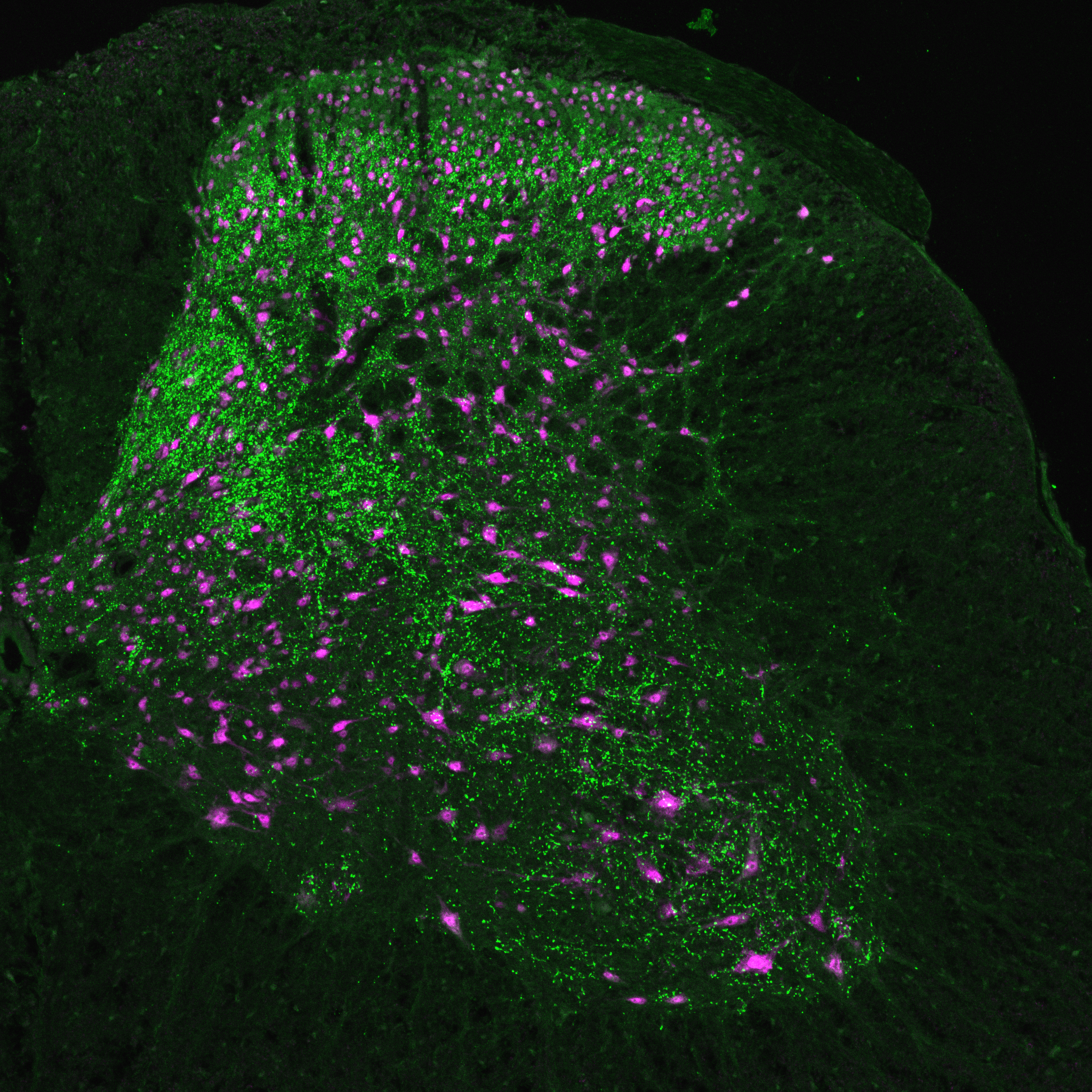

Supplement: Supplementary file 9 — Appendix. Fig. S1-10. [file 44318_2024_252_MOESM9_ESM.zip › Appendix. Fig. S1-10/Appendix. Fig. S9/S9 C/sup. Figure C WT VGLUT1_NeuN merge.tif]
